# A coalescent sampler successfully detects biologically meaningful population structure overlooked by *F*‐statistics

**DOI:** 10.1111/eva.12712

**Published:** 2018-10-15

**Authors:** Eric D. Crandall, Robert J. Toonen, ToBo Laboratory, Kimberly A. Selkoe

**Affiliations:** ^1^ School of Natural Sciences California State University, Monterey Bay Seaside California; ^2^ School of Ocean and Earth Science and Technology, Hawai‘i Institute of Marine Biology University of Hawai‘i at Manoa Kane‘ohe Hawaii; ^3^ National Center for Ecological Analysis and Synthesis Santa Barbara California

**Keywords:** coalescent sampler, gene flow, isolation by distance, mtDNA, population structure, simulation, stepping‐stones

## Abstract

Assessing the geographic structure of populations has relied heavily on Sewell Wright's *F*‐statistics and their numerous analogues for many decades. However, it is well appreciated that, due to their nonlinear relationship with gene flow, *F*‐statistics frequently fail to reject the null model of panmixia in species with relatively high levels of gene flow and large population sizes. Coalescent genealogy samplers instead allow a model‐selection approach to the characterization of population structure, thereby providing the opportunity for stronger inference. Here, we validate the use of coalescent samplers in a high gene flow context using simulations of a stepping‐stone model. In an example case study, we then re‐analyze genetic datasets from 41 marine species sampled from throughout the Hawaiian archipelago using coalescent model selection. Due to the archipelago's linear nature, it is expected that most species will conform to some sort of stepping‐stone model (leading to an expected pattern of isolation by distance), but *F*‐statistics have only supported this inference in ~10% of these datasets. Our simulation analysis shows that a coalescent sampler can make a correct inference of stepping‐stone gene flow in nearly 100% of cases where gene flow is ≤100 migrants per generation (equivalent to *F*_ST_ = 0.002), while *F*‐statistics had mixed results. Our re‐analysis of empirical datasets found that nearly 70% of datasets with an unambiguous result fit a stepping‐stone model with varying population sizes and rates of gene flow, although 37% of datasets yielded ambiguous results. Together, our results demonstrate that coalescent samplers hold great promise for detecting weak but meaningful population structure, and defining appropriate management units.

## 1 INTRODUCTION

The delineation of population genetic structure is a long‐standing problem in ecology and conservation of natural populations (Funk, McKay, Hohenlohe, & Allendorf, 2012; Hellberg, 2009; Palsbøll, Bérubé, & Allendorf, 2007; Selkoe, D'Aloia, et al., 2016; Waples, 1998). Particularly in marine systems, large population sizes and relatively high rates of gene flow (via a planktonic larval stage) coincide to create high‐diversity datasets with low or nonexistent genetic structure as measured by traditional *F*‐statistics (Gagnaire et al., 2015; Riginos, Crandall, Liggins, Bongaerts, & Treml, 2016). This is chiefly because *F*_ST_ has a nonlinear relationship with gene flow such that flows greater than about 10 migrants per generation cannot be statistically distinguished from *F*_ST_ = 0 using realistic sample sizes (Waples, 1998). As a result, studies of species with large and variable population sizes and moderate gene flow are often unable to reject the null hypothesis that all sampled individuals are part of a single, randomly mating population (panmixia), even when population samples are separated by hundreds of kilometers.

This problem is especially acute in the face of growing evidence that mean larval dispersal distances are typically <100 km (Almany et al., 2017; Cowen & Sponaugle, 2009; D'Aloia et al., 2015; Kinlan & Gaines, 2003; Schunter, Pascual, Garza, Raventos, & Macpherson, 2014). We would thus expect population structure for marine species with larval dispersal to be governed by a model of isolation by distance (IBD; Wright, 1943), wherein nearby individuals are more likely to mate than distant individuals, or, more specifically, by a stepping‐stone model, a special case of IBD wherein individuals are lumped into spatially discrete demes and dispersal occurs only between neighboring demes (Kimura & Weiss, 1964), such as would be expected in an island archipelago system. However, less than one third of marine population genetic studies to date have found a significant correlation between geographic distance and *F*_ST_ that is diagnostic of IBD (Selkoe & Toonen, 2011), probably due to (a) lack of sensitivity to weak structure in species with high gene flow and large population sizes discussed above, and (b) a lack of equilibrium between genetic drift and gene flow caused by population growth and range expansions, especially those that followed the Last Glacial Maximum (LGM) (Crandall, Sbrocco, DeBoer, Barber, & Carpenter, 2012; Slatkin, 1993).

Coalescent genealogy samplers provide a promising alternative to methods based on *F*‐statistics (reviewed by Kuhner, 2009; Marko & Hart, 2011). When viewed backward in time, a metapopulation's genealogy will coalesce to nodes of common ancestry. By repeatedly evaluating genealogies and favoring those with high likelihood of describing the data in a Bayesian Markov chain Monte Carlo framework, coalescent samplers can obtain estimates of population genetic parameters, such as effective population size (*N*_e_) and the proportion of migrants (*m*). By adding additional Markov chains with higher acceptance ratios that search most of parameter space (path sampling), these programs are also able to evaluate the marginal likelihood of alternative models of population structure (Beerli & Palczewski, 2010). In comparison with *F*_ST_ methods, coalescent methods use information from genealogy in addition to information about allele frequency, and should be much better able to characterize gene flows higher than 10 migrants per generation so long as N_e_ is large and *m* is relatively small (the structured coalescent; Wakeley, 2004; Crandall, Treml, & Barber, 2012).

**Figure 1:**
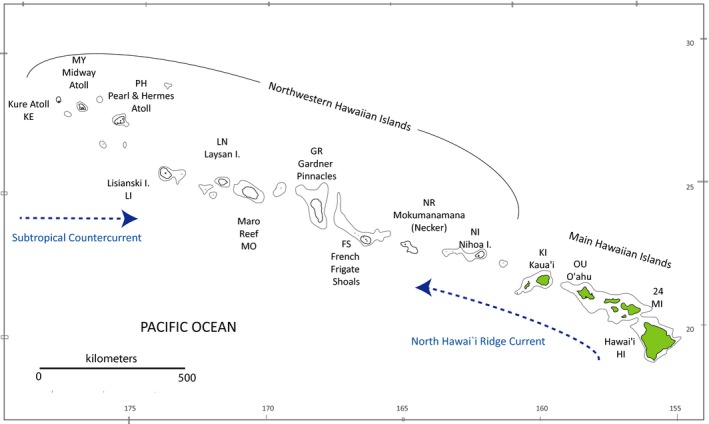
Map of the Hawaiian archipelago, with names and abbreviations of sample sites

Extending almost linearly more than 2,500 km from the hotspot in the southeast to Kure Atoll in the northwest, the Hawaiian archipelago provides an excellent test of our ability to characterize population genetic structure in a linear stepping‐stone array of populations (Figure 1). The archipelago supports coral reef habitat on every island and atoll and is isolated from the rest of the Indo‐Pacific region by more than 800 km of open ocean. Biophysical modeling demonstrates a clear expectation for IBD, with neighboring islands exchanging many more larvae than distant islands (Wren, Kobayashi, Jia, & Toonen, 2016). However, population genetic surveys of over 40 marine species have yielded only four that show the predicted correlation between *F*_ST_ and geographic distance, with the majority showing some form of genetic structure separating large panmictic regions (regional structure), with smaller fractions showing “chaotic” population structure with no relationship to geography, or apparent panmixia across the entire archipelago (reviewed in Selkoe, Gaggiotti, Bowen, & Toonen, 2014; Toonen et al., 2011).

In this study, we re‐examine genetic datasets from 41 marine species in a model‐selection framework using a popular coalescent sampler: migrate‐n (Beerli & Felsenstein, 2001; Beerli & Palczewski, 2010). We first validate the method through simulation of stepping‐stone dispersal at the characteristically high effective population sizes and rates of gene flow that are expected for marine species. We then analyze each dataset, calculating the relative probability of a stepping‐stone model in comparison with panmixia, the *n*‐island model (equal gene flow exchanged between all populations; Wright, 1931) and various hypotheses of regional structure to compare the model‐selection approach to direct interpretation of *F*‐statistics.

## 2 METHODS

### 2.1 Simulations

Using IBDsim 2.0 (Leblois, Estoup, & Rousset, 2009), we simulated stepping‐stone dispersal among 10 equally sized demes in a one‐dimensional lattice with a fixed proportion of migrants moving between neighboring demes. We created nine simulated parameter sets that varied effective population size (*N*_e_ = {10^4^, 10^5^, 10^6^}) and proportion of migrants (*m* = {10^−1^, 10^−2^, 10^−3^, 10^−4^, 10^−5^}) for all combinations where *N*_e_ *m* was equal to 10, 100, or 1,000 migrants per generation, as well as a panmictic dataset that was simulated as a single population with *N*_e_ = 10^6^ that was then subdivided into 10 demes (Table 1). Because the Hawaiian marine populations are thought to have undergone demographic expansion following sea level rise after the LGM which ended 14–20 thousand years ago (Baums, Godwin, Franklin, Carlon, & Toonen, 2013), we simulated an order of magnitude increase in effective population size for each deme to reach the final, given value for *N*_e_. This population expansion occurred 10,000 generations ago, approximating the end of the LGM (many of the study species have a generation time of ~2 years). We sampled 20 post‐dispersal individuals from each population, with a simulated sequence of 500 bp of haploid DNA evolving under the HKY85 model (transition/transversion ratio = 9.0, base frequencies set to default), with a per‐base mutation rate of 10% per million generations (i.e., mitochondrial DNA, see Crandall, Sbrocco, et al., 2012). We simulated 100 replicate datasets of each parameter set.

**Table 1:** Parameter sets for simulations of stepping‐stone dispersal with equal levels of migration among equally sized demes using IBDsim. 100 datasets were simulated per parameter set

Parameter set	Effective population size (*N*_e_)	Proportion of migrants (*m*)	Effective number of migrants (*N*_e_ *m*)
1	10^4^	10^−^^3^	10
2	10^5^	10^−^^4^	10
3	10^6^	10^−^^5^	10
4	10^4^	10^−^^2^	100
5	10^5^	10^−^^3^	100
6	10^6^	10^−^^4^	100
7	10^4^	10^−^^1^	1,000
8	10^5^	10^−^^2^	1,000
9	10^6^	10^−^^3^	1,000

**Figure 2:**
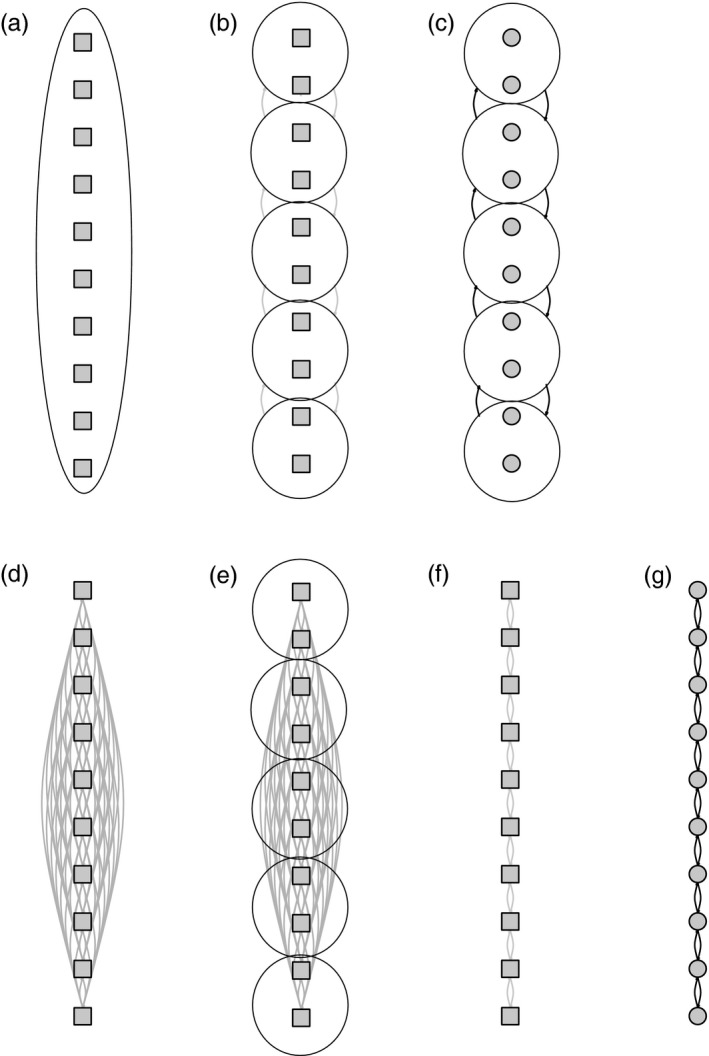
Migrate‐n models that were evaluated for each dataset simulated in Table 1. Squares represent a single Θ parameter across all populations, and circles represent distinct parameters Θ for all populations. Gray lines represent a single *m*/*μ* parameter across all populations, while black lines represent distinct *m*/*μ* across all populations. (a) Panmixia, (b) five regional groups with a shared Θ and *m*/*μ* parameters, (c) five regional groups with a distinct Θ and *m*/*μ* parameters, (d) island model, (e) island model for five regional groups, (f) stepping‐stone model with shared values for Θ and *m*/*μ* parameters (the true model), (g) stepping‐stone model with distinct parameters for Θ and *m*/*μ* parameters

For each replicate simulated dataset, we calculated pairwise Φ_ST_ (Excoffier, Smouse, & Quattro, 1992) and *θ* (Weir & Cockerham, 1984) with the StrataG package for R (Archer, Adams, & Schneiders, 2017), and then assessed the significance of the relationship between pairwise genetic distance and geographic distance along the lattice (i.e., IBD) using a Mantel test as implemented in the adegenet package for R (Jombart & Ahmed, 2011). For 10 replicate datasets from each parameter set, we also estimated the marginal likelihood of seven different metapopulation models in migrate‐n (Figure 2): (a) a stepping‐stone model with freely varying *m*/*μ* and Θ = *N*_e_μ parameters (where *N*_e_ is the effective population size, *m* is the proportion of individuals in the population that are migrants, and *μ* is mutation rate), (b) a stepping‐stone model with single estimated parameters for *m*/*μ* and Θ (the true model), (c) a stepping‐stone model between five lumped pairs of demes with freely varying parameters or (d) single estimated parameters for *m*/*μ* and Θ (models 3 and 4 representing regional structure), (e) an island model with 10 demes (migration between all possible demes pairs with a single estimated parameter for *m*/*μ* and Θ) (f) an island model with five demes, and (g) a model of panmixia. Migrate‐n was run with the same priors, and other parameter file settings as are described below for the empirical datasets.

### 2.2 Migrate‐n analysis of empirical data

Empirical datasets comprised mitochondrial data from 41 species sampled during NOAA expeditions throughout the main Hawaiian archipelago and Northwestern Hawaiian Islands from 2005 to 2012 (Figure 1, Supporting Information Table S1; Selkoe et al., 2014; Selkoe, Gaggiotti, et al., 2016; Toonen et al., 2011). Locality samples were grouped by island, and each dataset was converted from Arlequin format to Nexus and migrate‐n formats in batch via pgdspider 2.0.5.1 (version 2.0.5.1; Lischer & Excoffier, 2012). An optimal HKY model of molecular evolution for each dataset was selected with jModelTest (Darriba, Taboada, Doallo, & Posada, 2012).

Parameter input files for migrate‐n were constructed using a custom script in R. All models had identical, windowed exponential priors on Θ (lower bound: 1 × 10^−5^, upper bound: 1 × 10^−1^, mean: 0.01) and *m*/*μ* (lower bound: 1 × 10^−4^, upper bound: 1 × 10^6^, mean: 1 × 10^5^) parameters. Assuming a mutation rate of 10% per million years, these priors represent the belief that each island population's effective size is <1 million (Hare et al., 2011), and the proportion of migrants is <10% of that (i.e., <100,000 migrants/generation; Wren et al., 2016). We used four heated chains with temperatures of 1, 1.5, 3, and 1 × 10^5^ to ensure a thorough search of parameter space, thereby enabling an estimate of model marginal likelihood via path sampling (Beerli & Palczewski, 2010). Migrate‐n was set to optimize on the *m*/*μ* parameter rather than the joint parameter *N*_e_ *m*, and with an inheritance scalar that reflected the haploid, uniparental transmission of mtDNA. For each model, the coolest chain explored five million genealogies, sampling every 100 iterations, and discarding the first two million genealogies as burn‐in. Parameter files for each species and each model are available in the Github repository.

**Figure 3:**
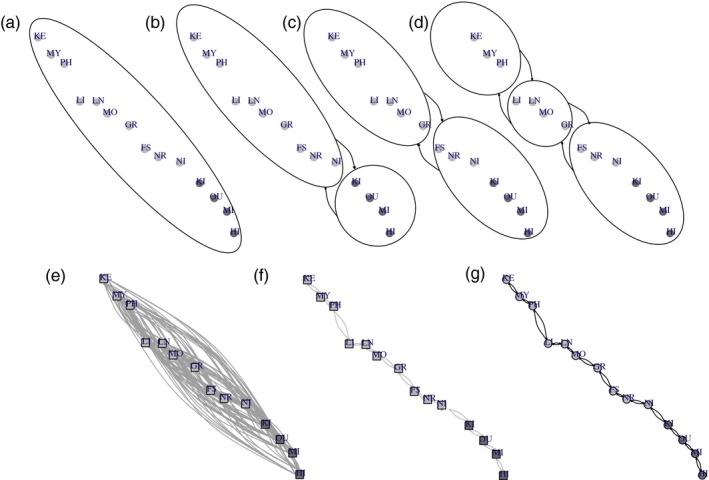
Migrate‐n models that were evaluated for each empirical dataset. Squares represent a single Θ parameter across all populations and circles represent distinct parameters Θ for all populations. Gray arrows represent a single *m*/*μ* parameter across all populations, while black arrows represent distinct *m*/*μ* across all populations. High islands are shaded dark gray, while atolls and reefs are shaded light gray. Panmictic populations are enclosed in ellipses. (a) Panmixia, (b) regional structure between the main Hawaiian islands and the Northwestern Hawaiian Islands, (c) regional structure due to a current passing between French Frigate Shoals and Gardner Pinnacles, (d) regional structure due to the current in C, and another current between Lisianski Atoll and Pearl and Hermes Atoll, (e) an island model, (f) a stepping‐stone model with shared values for Θ and *m*/*μ* parameters, (g) a stepping‐stone model with independent values for Θ and *m*/*μ*

For each species’ dataset, we created seven or eight metapopulation models to compare in a model‐selection framework (Figure 3). We first modeled panmixia as all samples belonging to a single deme (*K* = 1, 1 Θ parameter). We modeled regional structure as two panmictic demes (*K* = 2, 2 Θ parameters, 2 *m*/*μ* parameters), with a barrier to gene flow occurring (a) between the MHI and the NWHI (high–low hypothesis), (b) between French Frigate Shoals and Gardner Pinnacles due to a current that bisects the archipelago there (Wren et al., 2016), (c) regional structure as three panmictic demes with two barriers based on currents that run between French Frigate Shoals and Gardner Pinnacles, and between Lisianski Atoll and Pearl and Hermes Atoll (two‐currents hypothesis, *K* = 3, 3 Θ parameters, 6 *m*/*μ* parameters). We modeled the island model as migration at a single rate between all *n* sampled populations, which have a single shared population size (*K* = *n*, 1 Θ parameter, 1 *m*/*μ* parameter). We modeled stepping‐stone migration between neighboring islands by either fixing Θ and *m*/*μ* each to a single estimated parameter (stepping‐stone two‐parameter hypothesis, *K* = *n*, 1 Θ parameter, 1 *m*/*μ* parameter), or allowing each parameter to vary freely (stepping‐stone hypothesis, *K* = *n*,* n* Θ parameters, [2*n* − 2] *m*/*μ* parameters). Finally, for some species where Selkoe et al. (2014) had inferred regional structure that departs from the models specified above, we modeled the observed empirical structure for that species.

Three replicates of each metapopulation model were run using migrate‐n version 3.6.9 prior to estimation of the marginal likelihood via path sampling. We used the Bezier‐corrected estimate in each case as it provides a good approximation to a marginal likelihood calculated from a large number of heated chains (Beerli & Palczewski, 2010). We then reran all models for all species two more times for a total of nine replicate runs of each model, yielding three estimates of marginal likelihood. All model runs were performed on the University of Hawaii high‐performance computing (HPC) cluster.

Model runs did not always yield the same marginal likelihood, but were usually similar (within ~10 points of log‐likelihood; Supporting Information Figure S1), so we took the mean marginal likelihood values across the three replicate runs. To accommodate variance in estimated log‐likelihood across replicates, we tested for significance of the best model by comparing the mean marginal likelihood to the second‐best model for each species using a permutation *t* test executed in the R‐package perm (Fay & Shaw, 2010). Species with a permutation *t* test *p*‐value > 0.05 were considered to have significant ambiguity in their top‐ranked metapopulation model.

For species that had a non‐ambiguous inference of a full stepping‐stone model, we tested for a significant relationship between area of shallow ocean habitat <10 fathoms deep in square kilometers (Rohmann, Hayes, Newhall, Monaco, & Grigg, 2005) and the natural log of Θ = N_e_. We also tested for a significant relationship between approximate census size of each island (as estimated from densities reported in McCoy et al. (2017)) and the natural log of Θ. We did this by evaluating the slope for 10,000 linear models created by matching the area or census size of each sampled island with a random draw from the posterior distribution of Θ for that island. A significant relationship between island size and Θ was determined for any species that had a positive slope in at least 95% of the linear models.

Finally, we asked whether life history traits were predictive of metapopulation model (e.g., Does a long pelagic larval duration lead to inference of panmixia?). We used the suite of life history traits assembled by Selkoe et al. (2014, Supporting Information Table S1), which included pelagic larval duration, depth range, adult length, habitat specialist, attached eggs, herbivore, fish, and endemic, with the first three predictors being log transformed and the last five coded as logical values. We created a multinomial regression model using the nnet package in R (Venables & Ripley, 2002), and treating our expectation of a stepping‐stone model as the reference level, with the other three levels being regional structure, *n*‐island, and panmixia. The significance of each predictor was tested using a z test with the test statistic calculated as the model coefficient divided by its standard error. All R code, and infiles for IBDsim and migrate‐n are available on the GitHub repository linked in the data archiving statement.

## 3 RESULTS

### 3.1 Simulations

**Figure 4:**
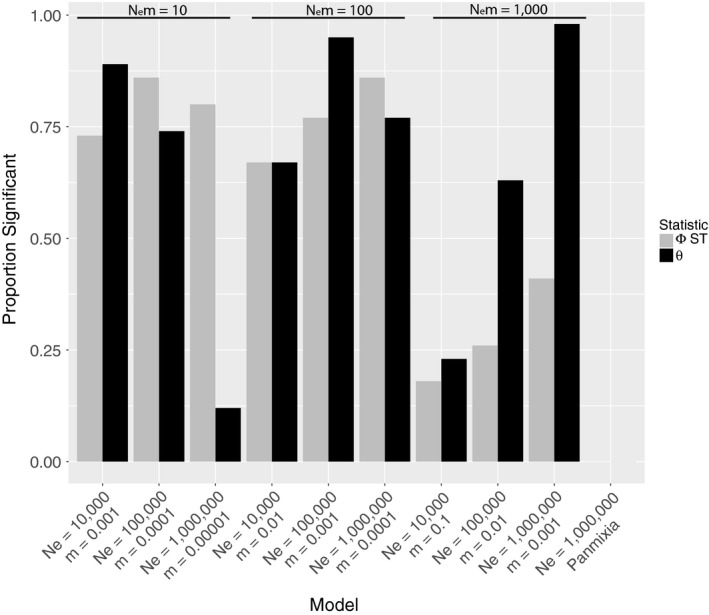
Proportion of simulated datasets showing a significant relationship between lattice distance and Φ_ST_ or Weir and Cockerham's *θ*

The two estimators of *F*_ST_ (Φ_ST_ and θ) had markedly different ability to recover a significant correlation with distance along the lattice in different simulated scenarios (Figure 4). In particular, when *N*_e_ = 10^6^, Φ_ST_ was more likely to be significant with distance at *N*_e_ *m* = 10, while θ was more likely to be significant with distance when *N*_e_ *m* = 1,000. Φ_ST_ ranged from 18% to 86% of datasets with a significant relationship to distance while θ ranged from 12% to 98%. Neither statistic showed any evidence of false‐positives when evaluating the panmictic dataset.

**Figure 5:**
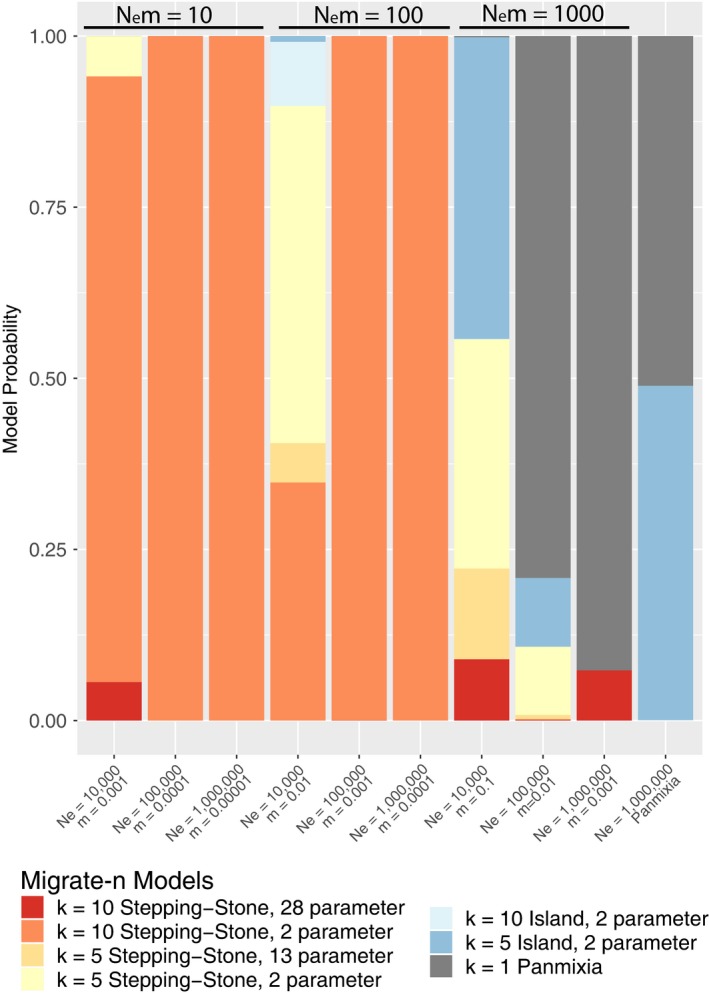
Relative probability for each of seven models evaluated with migrate‐n (depicted in Figure 2) for each simulated dataset in Table 1. Probabilities are averaged across 10 replicate simulated datasets for each combination of effective population size (*N*_e_) and proportion of migrants (*m*)

Migrate‐n chose the true model (Stepping‐stone two parameter) for all 10 datasets for each parameter set where *N*_e_ *m* = 10 with one exception (Figure 5). In one of ten datasets for *N*_e_ = 10^4^, the full 28‐parameter stepping‐stone model was selected. Similarly, for parameter sets where *N*_e_ *m* = 100, migrate‐n selected the true model in every case except when *N*_e_ was 10^4^. For this parameter set, it chose some version of a regional model (five stepping‐stone populations) in six out of 10 datasets. For datasets where *N*_e_ *m* = 1,000, migrate‐n never recovered the true model, inferring mostly panmixia for *N*_e_ of 10^5^ and 10^6^, and a variety of models for *N*_e_ of 10^4^. When the true model was panmixia, migrate‐n selected panmixia for five of the replicates and the *n*‐island model for the other five.

Parameter estimates for *N*_e_ *m* and *N*_e_ were consistently about one twentieth to one half of the true simulated value (Supporting Information Figure S2). We attribute this outcome to the order‐of‐magnitude population growth experienced by each population, as migrate‐n estimates of Θ are expected to be downwardly biased in the case of such population growth (Beerli, 2009). Saturation is also likely to play a role when *N_e_* = 1 million, as these datasets had over 60% variable sites. Migrate's estimates of gene flow are also known to be downwardly biased when true migration is high, due to the need to truncate the number of migration events to avoid memory overflow (Beerli & Felsenstein, 1999).

### 3.2 Empirical data

**Figure 6:**
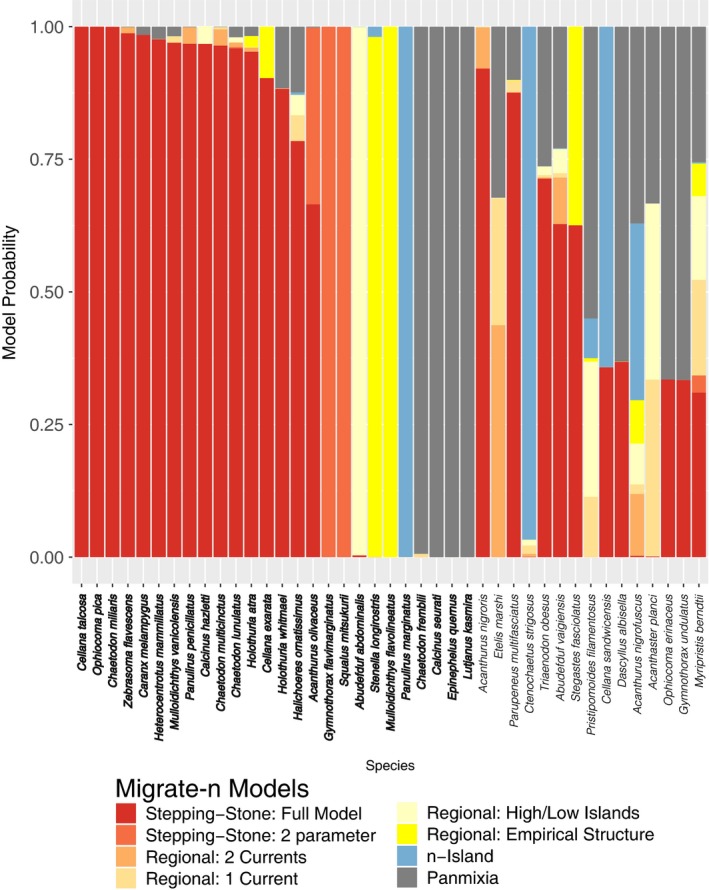
Relative probability for each of eight models evaluated with migrate‐n (depicted in Figure 3) for each empirical dataset. Probabilities are averaged across three replicate migrate‐n runs. Species names for which the best model was unambiguous are printed in boldface

Migrate‐n inferred some form of stepping‐stone model for 18 of 26 species for which the model was unambiguous (69%; Figure 6). Regional structure was inferred for three species, the sergeant major *Abedefduf abdominalis*, spinner dolphin *Stenella longirostris,* and yellowstripe goatfish, *Mulloidichthys flavolineatus*. For all three of these species, migrate‐n analysis confirmed inferences based on *F*_ST_ of regional structure, regional structure, and chaotic structure, respectively (Selkoe et al., 2014). An island model was selected for the spiny lobster, *Panulirus marginatus*, and panmixia was the best model for four species: two with Indo‐Pacific distributions: Bluestripe snapper (*Lutjanus kasmira*) and Zebra hermit crab (*Calcinus seurati*), and two Hawaiian endemics: Bluestripe Butterflyfish (*Chaetodon fremblii*) and Hawaiian grouper (*Epinephelus quernus*).

There was no clear pattern in the 15 species that did not yield a single best model over three replicates. Five species had the full stepping‐stone model at an average posterior probability over 50%, while four species had the same for panmixia and one for the *n*‐island model. The other five had no majority model. A logistic regression found that haplotype diversity and Φ_ST_ were not predictive of whether a dataset gave ambiguous results or not.

For the species that yielded a single unambiguous model, posterior distributions for Θ, *m*/*μ*, and their product *N*_e_ *m* all had effective sample sizes greater than 200 (with the exception of some parameters for *Etelis marshii*). However, while Θ posteriors converged, many *m*/*μ* posteriors did not converge well, as indicated by multimodal distributions (Supporting Information Figure S3) and scale reduction factors >1.2. For this reason, we focus only on the minimum and maximum values (upper and lower bounds of 95% highest posterior density intervals) estimated for these parameters. The lowest *m*/*μ* value that fell within the 95% highest posterior density for any species was 3 × 10^−4^ (*Cellana talcosa*), and the highest was 9.9 × 10^5^ (*Chaetodon lunulatus*). The lowest Θ value for any species was 2.9 × 10^−6^ (*Cellana exarata*), while the highest value was 4 × 10^−1^ (*Gymnothorax flavimarginatus*). For *N*_e_ *m*, the lowest value for any species was 5.2 × 10^−7^ (*Cellana talcosa*) and the highest value was 2.3 × 10^4^ female migrants per generation (*Chaetodon lunulatus*).

The limpet *Cellana exarata* was the only species that showed a significant relationship between habitat size and log Θ (*p* = 0, Supporting Information Figure S4). No species showed a significant relationship between census size and log Θ. The only life history trait that was significantly correlated with our inferred models was herbivory, which was negatively correlated with panmixia (*p* = 0).

## 4 DISCUSSION

Our analysis of 41 marine species sampled along the Hawaiian archipelago with a coalescent genealogy sampler represents the largest and most thorough application of such a model testing framework to date. Out of the species for which we could select a model without significant ambiguity, we found that nearly 70% conformed to a stepping‐stone model of gene flow (Figure 6). This result should not be surprising given what we know about relatively short mean larval dispersal (D'Aloia et al., 2015; Treml et al., 2012) and the seascape of the Hawaiian archipelago (Wren et al., 2016). Yet this finding represents a striking departure from inferences based on *F*_ST_, which have only found evidence for IBD in about 10% of the species (Selkoe et al., 2014).

Although our result agrees with intuition, such a striking reversal requires some skepticism. What if our coalescent approach is somehow biased toward stepping‐stone models, or what if there is simply not enough information in mitochondrial datasets to make a reliable inference? For this reason, we conducted extensive simulations in the large population size and high gene flow region of population genetic parameter space that is occupied by most marine species (Gagnaire et al., 2015; Waples, 1998). We found that migrate‐n is able to return a correct inference of some form of stepping‐stone model (including regional structure) in 100% of cases where gene flow is 100 effective migrants per generation or less (Figure 5). This compares quite favorably to two analogs of *F*_ST_ which had variable success that hovered around 75% (Figure 4) for gene flows of 100 migrants per generation or less. Moreover, migrate‐n and *F*_ST_ methods both have low false‐positive rates, never inferring a stepping‐stone model when the true model was panmixia (although migrate‐n did infer an island model 50% of the time).

When dealing with “real‐world” mitochondrial datasets, the success rate of both coalescent (69%) and *F*_ST_‐based (10%) methods is apparently lower than the simulations would predict (perhaps due in part to natural selection on the mitochondrial genome; Ballard & Whitlock, 2004; Crandall, Sbrocco, et al., 2012, Teske et al., 2018), if we assume that some sort of isolation‐by‐distance model is correct in most marine species with larval dispersal. Indeed, IBD it is only detected in about 33% of studies globally (Selkoe & Toonen, 2011). Mitochondrial DNA data in particular are viewed as being problematic in this application (Teske et al., 2018). However, it may not be the data that are failing so much as the analytical approach: Given that *F*‐statistics remain the primary method by which marine population structure is diagnosed (Selkoe, D'Aloia, et al., 2016), the 10% success rate of *F*_ST_ methods in the Hawaiian archipelago (Selkoe et al., 2014; Toonen et al., 2011) and ~33% success rate globally (Selkoe & Toonen, 2011) are conspicuously low. Our combined simulation and empirical results suggest that coalescent samplers can detect population genetic structure even when *F*_ST_ or Mantel's R are not significantly different from zero, because *F*_ST_ and its analogues are simply not sensitive enough to detect it given realistic limitations to sampling designs for marine populations.

Coalescent methods provide a powerful complement to *F*_ST_ for the analysis of marine population genetic data (Marko & Hart, 2011). Large marine population sizes slow the effects of genetic drift and frequently create very low values of *F*_ST_ (Whitlock & McCauley, 1999). However, the fraction of migrants (*m*) that successfully disperse more than ~100 km (or in our specific case, the fraction that disperse between islands) is probably quite low, but still appreciably higher than the mutation rate. This fraction of migrants also scales with the size of the source population (Treml et al., 2012), meaning that marine populations are an excellent approximation of the structured coalescent model (Wakeley, 2004). In this case where *N*_e_ >> *N*_e_ *m* >> *μ* we have shown that migrate‐n can successfully identify a stepping‐stone structure with *N*_e_ *m* up to 100 migrants per generation (i.e., N_e_ at about 10,000 times larger than *N*_e_ *m*, which is still up to 10,000 times larger than *μ*), even with relatively coarse mitochondrial datasets. This level of sensitivity is analogous to successfully resolving IBD using significant *F*_ST_ values of around 0.002, something that is generally only possible with sample sizes well over 100 (Waples, 1998) and with numerous limiting assumptions (Whitlock & McCauley, 1999). It is worth noting that migrate‐n actually estimated *N*_e_ *m* as greater than 100 migrants per generation in every species in our dataset (Figure S3). We posit that reason for these higher estimated values is twofold: (a) because migrate‐n assumes that shared alleles are due to gene flow rather than recent divergence and (b) because some larvae disperse further than the neighboring island (Wren et al., 2016), meaning that the true model departs from a pure stepping‐stone model. However, with the current single‐locus datasets, migrate‐n was not able to distinguish between models that allowed single versus multi‐island dispersal, so we did not include these here (data not shown).

Waples (1998) astutely pointed out that even if a method is sensitive enough to detect population structure, significant genetic structure may not be biologically meaningful. Waples and Gaggiotti (2006) identified several criteria for biological relevance. First, for populations to be evolutionarily distinct, *N*_e_ *m* must be less than ~1 – 25 migrants per generation. Second, for populations to be ecologically distinct (demographically independent) the fraction of migrants *m* must be less than 10% (Hastings, 1993). In terms of the first criterion, our estimates for *N*_e_ *m* were generally above 100, and no species was geographically reciprocally monophyletic, suggesting that most Hawaiian marine species comprise a single evolutionarily significant unit (ESU). However, given census sizes in the millions per island (McCoy et al., 2017), hundreds or even tens of thousands of effective migrants per generation will not be ecologically relevant for conservation and management. We suggest that species for which we inferred a stepping‐stone model with a prior limit on the fraction of migrants of 10% (assuming a mutation rate of 10%/million years) have island populations that are demographically independent of one another. Confirmation of this suggestion would require a study with more loci, but in general, our results support earlier suggestions that each island should be treated as a distinct management unit (MU; Funk et al., 2012; Moritz, 1994; Palsbøll et al., 2007; Toonen et al., 2011).

Although we did not find much correlation between Θ and habitat size or abundance, it is notable that our approach detected heterogeneous population sizes and migration rates in most species. Of the 18 species for which migrate‐n inferred a stepping‐stone model, a full model where these parameters were free to vary was selected for all but two, for which a simplified two‐parameter stepping‐stone was selected (Figure 6). This is not a case of overfitting: In contrast to the empirical data, the simulations involved homogenous population sizes and migration rates, and migrate‐n almost always selected the corresponding two‐parameter model (Figure 5). Again, the inference of heterogeneous population sizes and migration rates is not surprising from a biological standpoint, but it marks an important improvement on what is detectable with traditional *F*‐statistics. Indeed, it has been shown that this parameter heterogeneity is likely masking the expected correlation between pelagic larval duration and genetic structure (Faurby & Barber, 2012). We expect that parameter estimates will improve with the addition of more loci (Felsenstein, 2006).

While migrate‐n did much better than *F*_ST_ with simulated data, and inferred structure more readily with the empirical data, it is also instructive to look at seven cases where migrate‐n did not detect IBD. Of the four species for which migrate‐n inferred panmixia, we know that one of them, *Lutjanus kasmira,* is an alien invasive species recently introduced to the archipelago that has undergone rapid population growth indicative of the source population rather than geographic structure (Gaither, Toonen, & Bowen, 2012). As is often inferred in the literature for results where *F*_ST_ is not significantly different from zero, we do not believe that the other three species are truly panmictic, but that they simply have recent, non‐equilibrium gene flow throughout the archipelago that is substantially greater than 100 migrants per generation (resulting from, e.g., range expansions; Dawson, Grosberg, Stuart, & Sanford, 2010). In three cases when migrate‐n inferred regional structure, the inference was in complete agreement with that achieved by *F*‐statistics (Supporting Information Table S1). Again, we doubt that each region is fully panmictic, but rather that we are likely detecting hierarchical structure on top of weaker isolation‐by‐distance processes.

Twenty years ago, when Waples (1998) first described the challenges inherent to describing population structure in marine species with genetic data, he highlighted a low signal‐to‐noise ratio in genetic data that has persisted through to today's research (Selkoe, D'Aloia, et al., 2016). Here, we have shown that, using a model‐selection framework, coalescent genealogy samplers are able to distinguish demographically independent stocks, or management units (signal), in marine species with evolutionarily high levels of gene flow (noise) that overwhelm traditional *F*‐statistics. With the recent availability of data from thousands of loci, such as microhaplotypes (Baetscher, Clemento, Ng, Anderson, & Garza, 2018) or whole genome sequencing, we expect that our approach will be of great use when applied by marine ecologists and managers looking for more sensitive tools for stock delineation, and that this approach will help to define the appropriate geographic scale for management.

## 5 DATA ARCHIVING STATEMENT

All empirical genetic data are available in the Genomics Observatories Metadatabase (GeOMe; Deck et al., 2017; https://www.geome-db.org/query). Code, simulated datasets, and supplemental figures are available at https://github.com/ericcrandall/hawaii_migrate.
