# Supplementary material for: A coalescent sampler successfully detects biologically meaningful population structure overlooked by F‐statistics
Source: Evol Appl. 2018 Oct 15;12(2):255–65. doi: 10.1111/eva.12712 (PMC6346657; doi:10.1111/eva.12712)
Supplement: Supplementary file 1 [file EVA-12-255-s001.pdf]

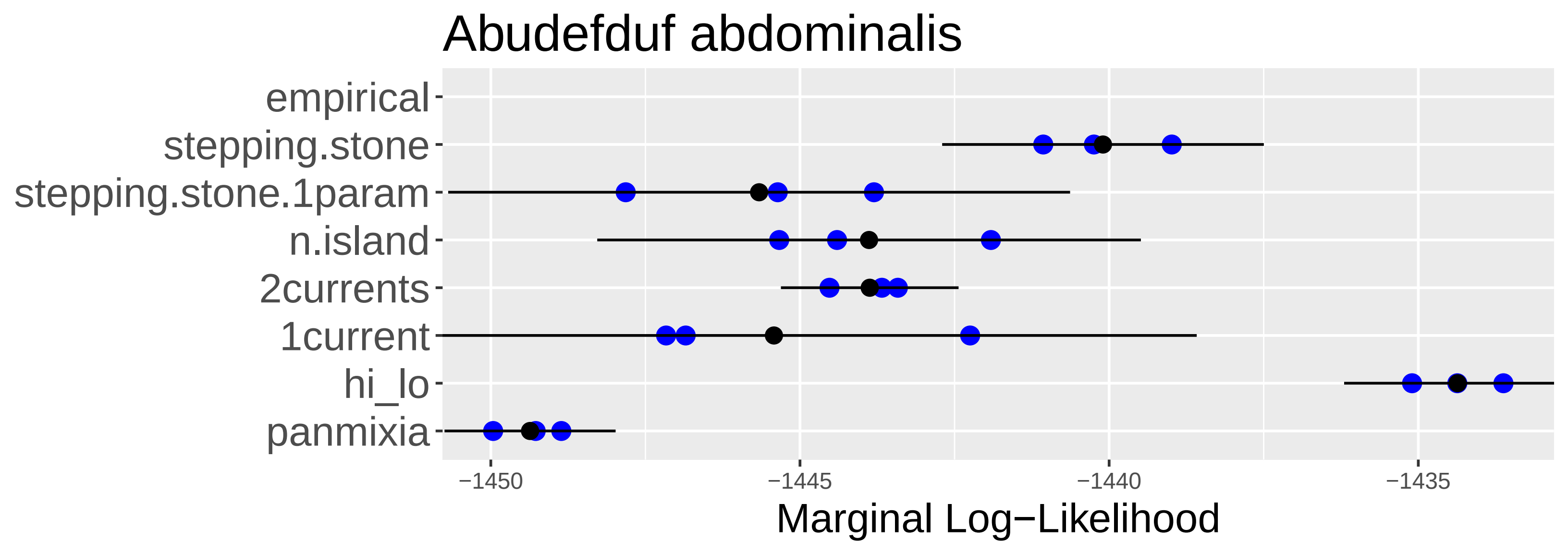

Abudefduf abdominalis
empirical
stepping.stone
stepping.stone.1param
n.island
2currents
1current
hi_lo
panmixia
−1450
−1445
−1440
−1435
Marginal Log−Likelihood

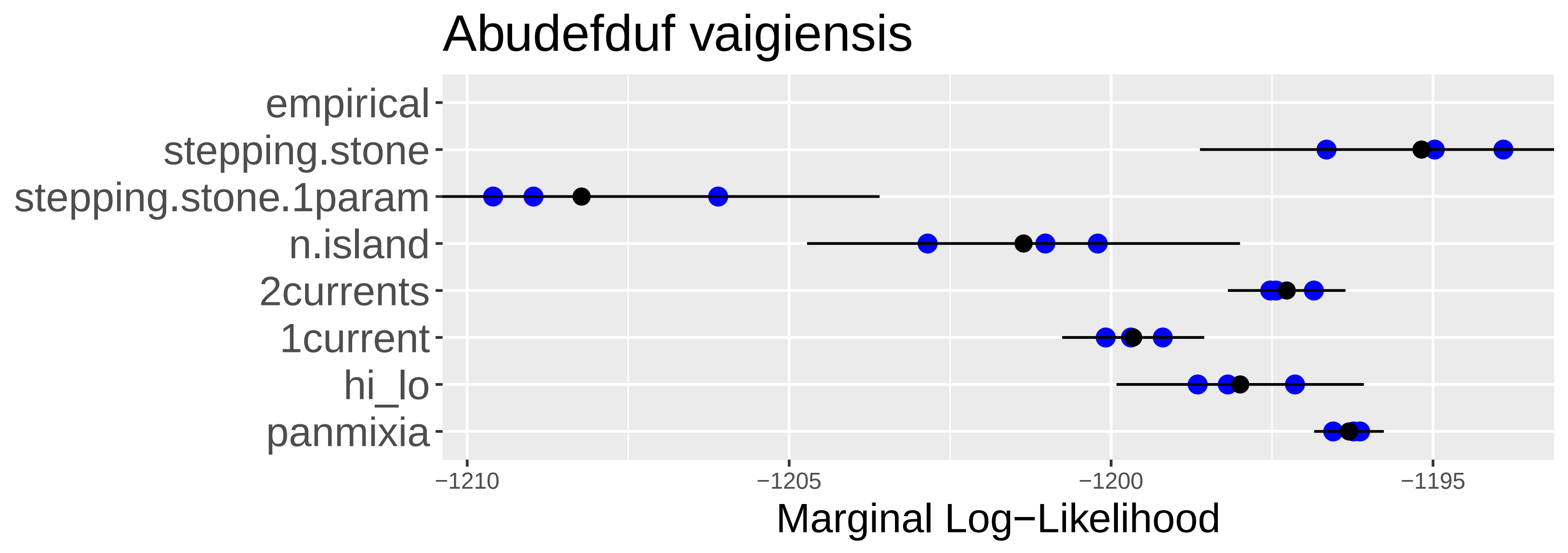

Abudefduf vaigiensis
empirical
stepping.stone
stepping.stone.1param
n.island
2currents
1current
hi_lo
panmixia
−1210
−1205
−1200
−1195
Marginal Log−Likelihood

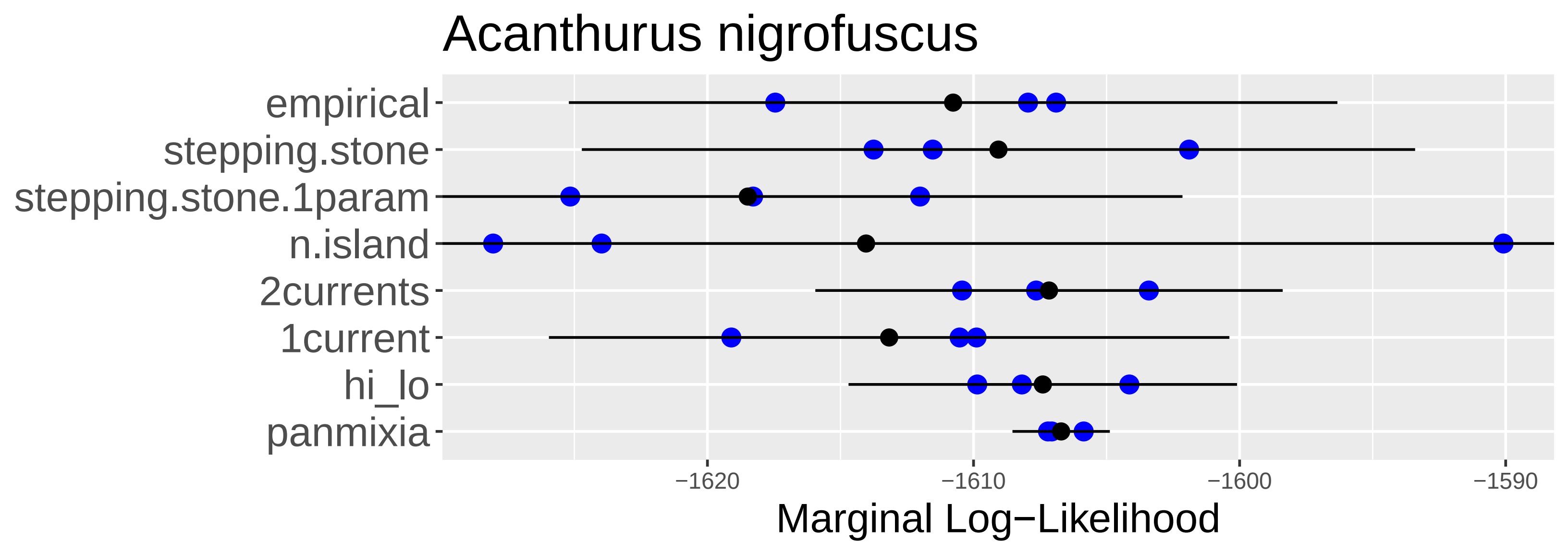

Acanthurus nigrofuscus
empirical
stepping.stone
stepping.stone.1param
n.island
2currents
1current
hi_lo
panmixia
−1620
−1610
−1600
−1590
Marginal Log−Likelihood

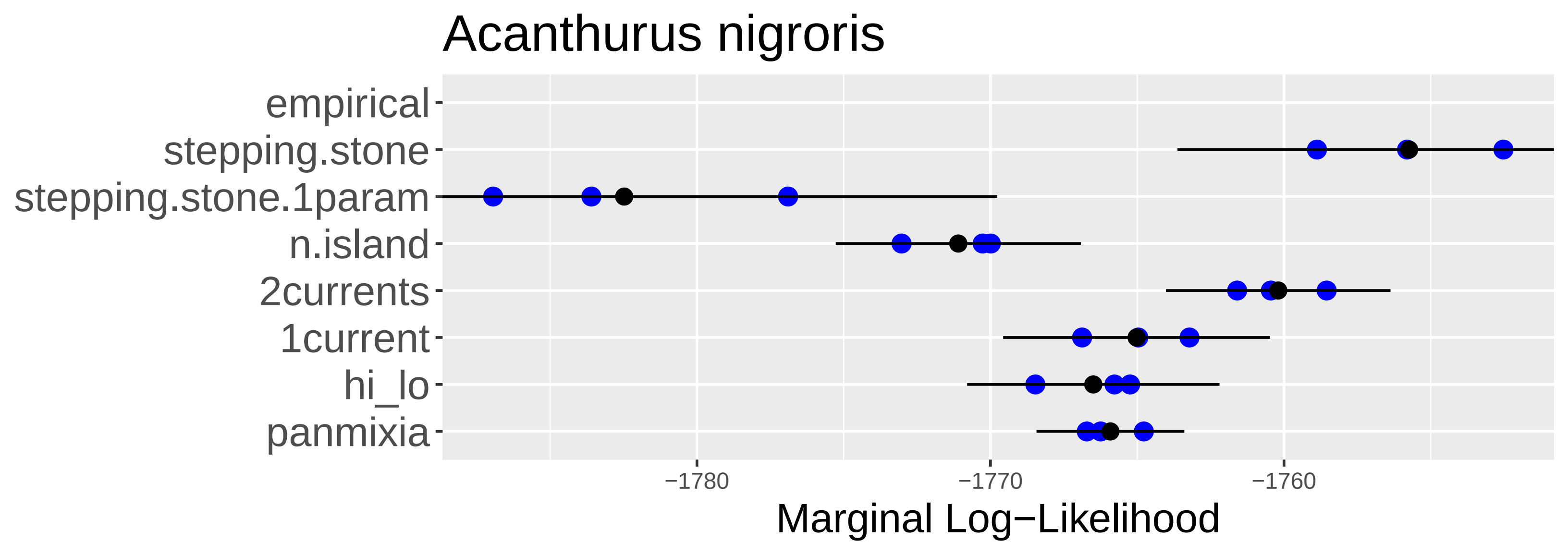
Acanthurus nigroris
empirical
stepping.stone
stepping.stone.1param
n.island
2currents
1current
hi_lo
panmixia
−1780
−1770
−1760
Marginal Log−Likelihood

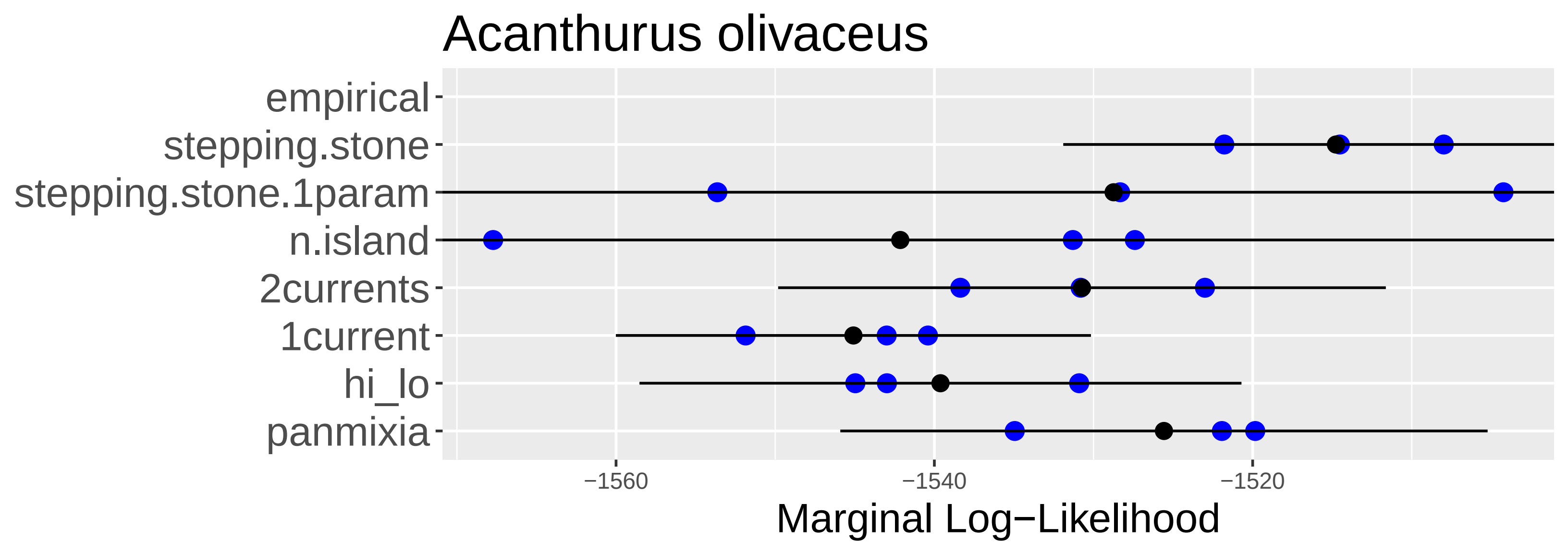

Acanthurus olivaceus
empirical
stepping.stone
stepping.stone.1param
n.island
2currents
1current
hi_lo
panmixia
−1560
−1540
−1520
Marginal Log−Likelihood

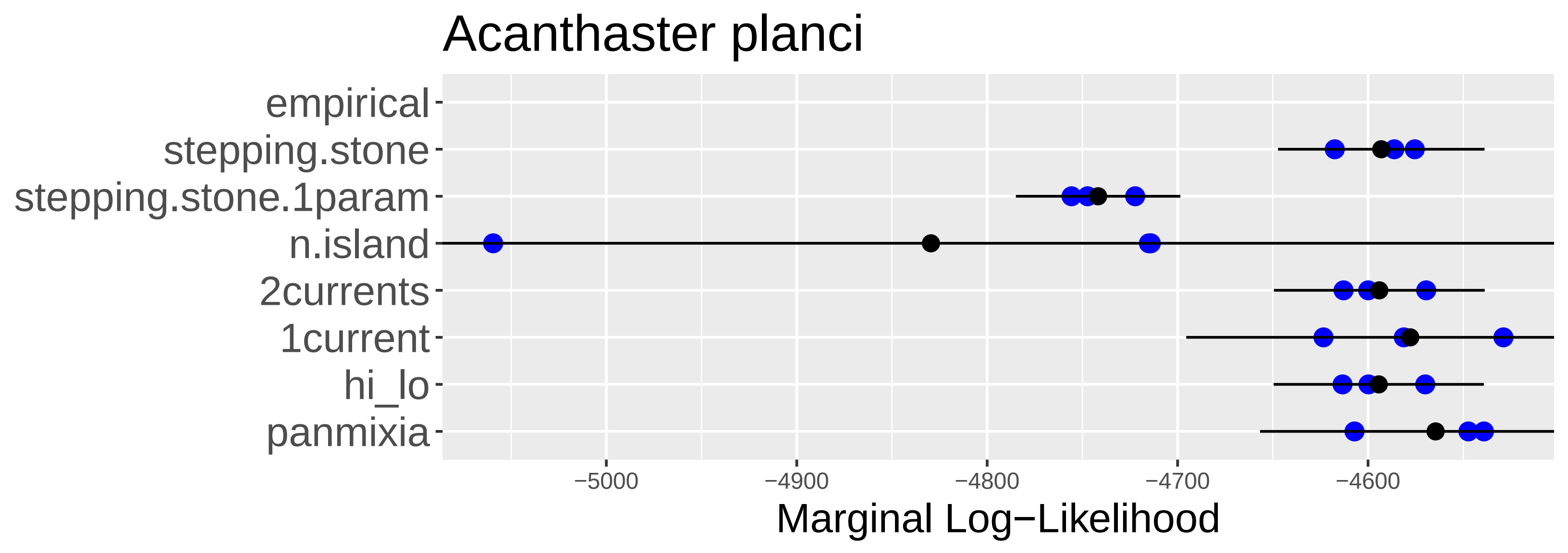

Acanthaster planci
empirical
stepping.stone
stepping.stone.1param
n.island
2currents
1current
hi_lo
panmixia
−5000
−4900
−4800
−4700
−4600
Marginal Log−Likelihood

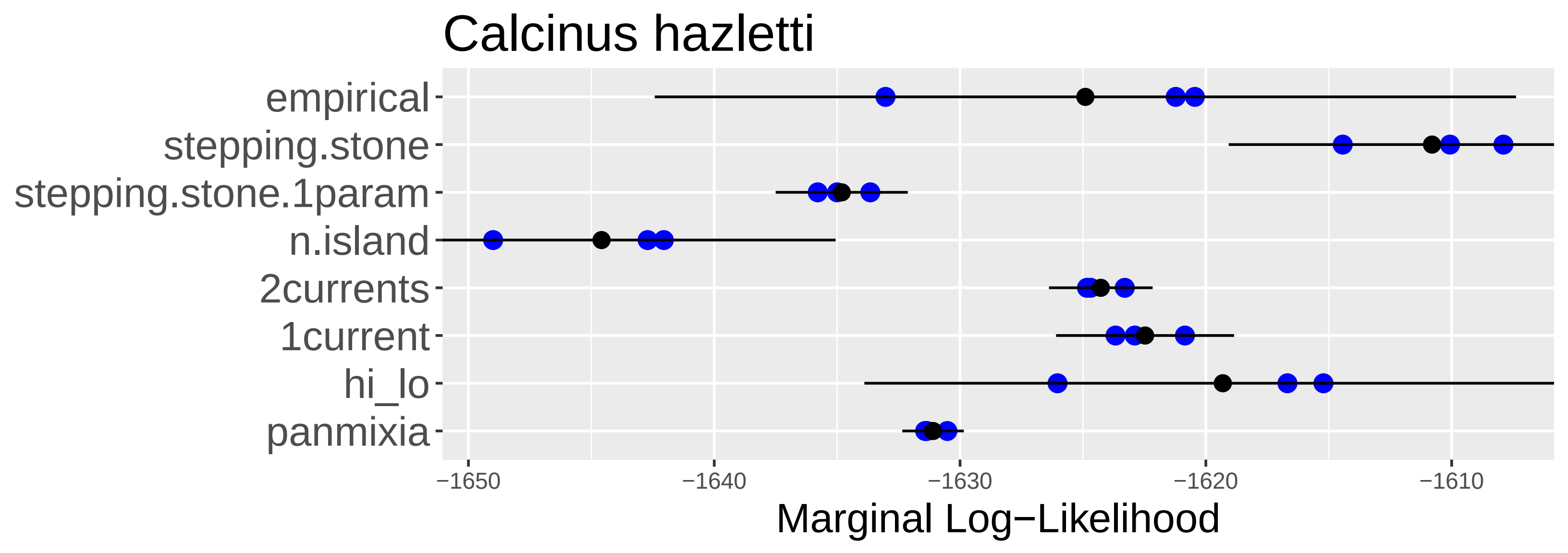

Calcinus hazletti
empirical
stepping.stone
stepping.stone.1param
n.island
2currents
1current
hi_lo
panmixia
−1650
−1640
−1630
−1620
−1610
Marginal Log−Likelihood

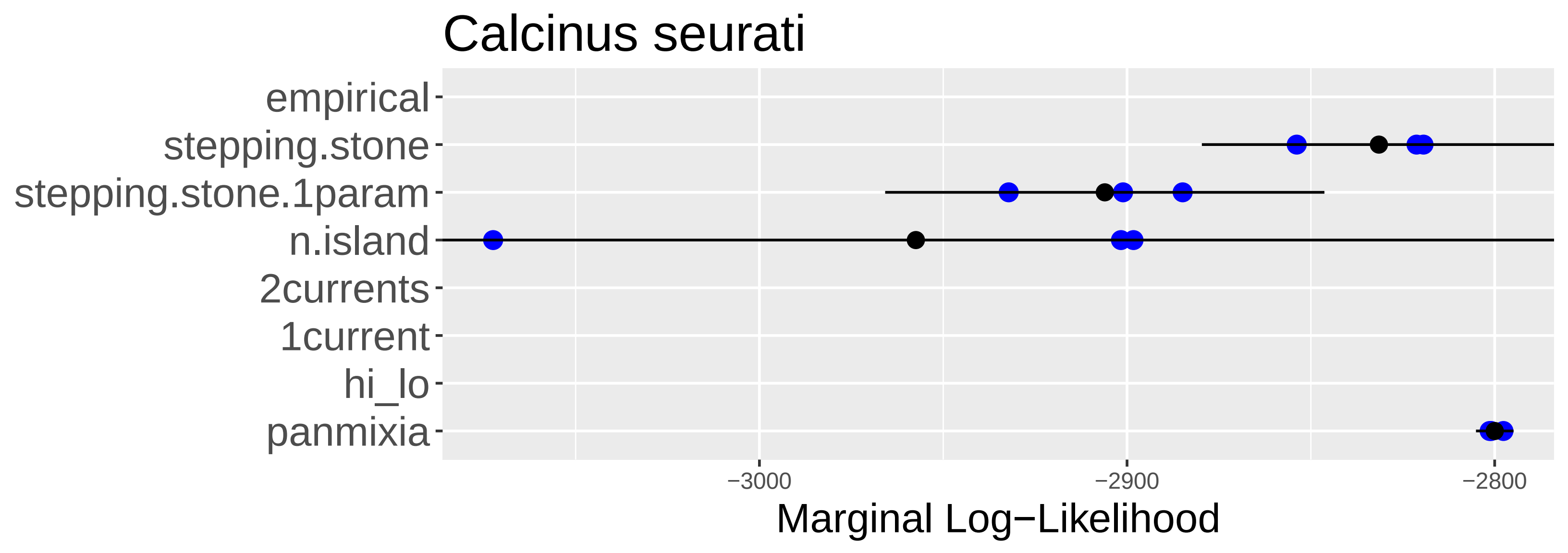

Calcinus seurati
empirical
stepping.stone
stepping.stone.1param
n.island
2currents
1current
hi_lo
panmixia
−3000
−2900
−2800
Marginal Log−Likelihood

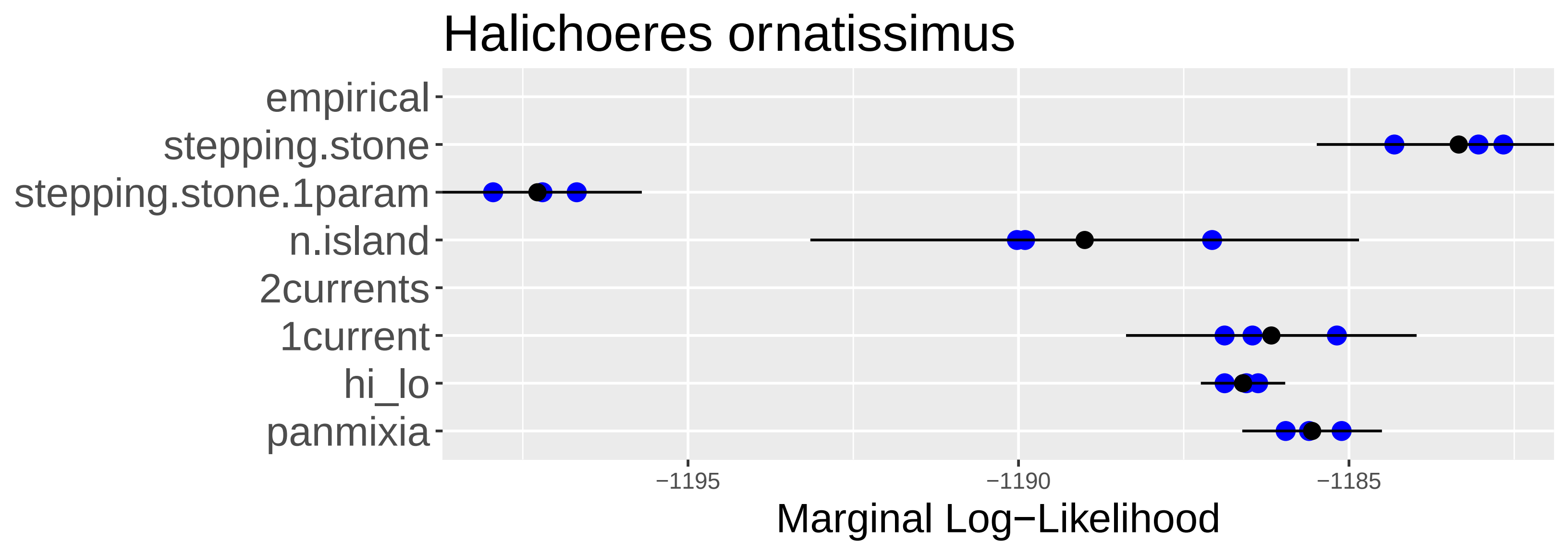
Halichoeres ornatissimus
empirical
stepping.stone
stepping.stone.1param
n.island
2currents
1current
hi_lo
panmixia
−1195
−1190
−1185
Marginal Log−Likelihood

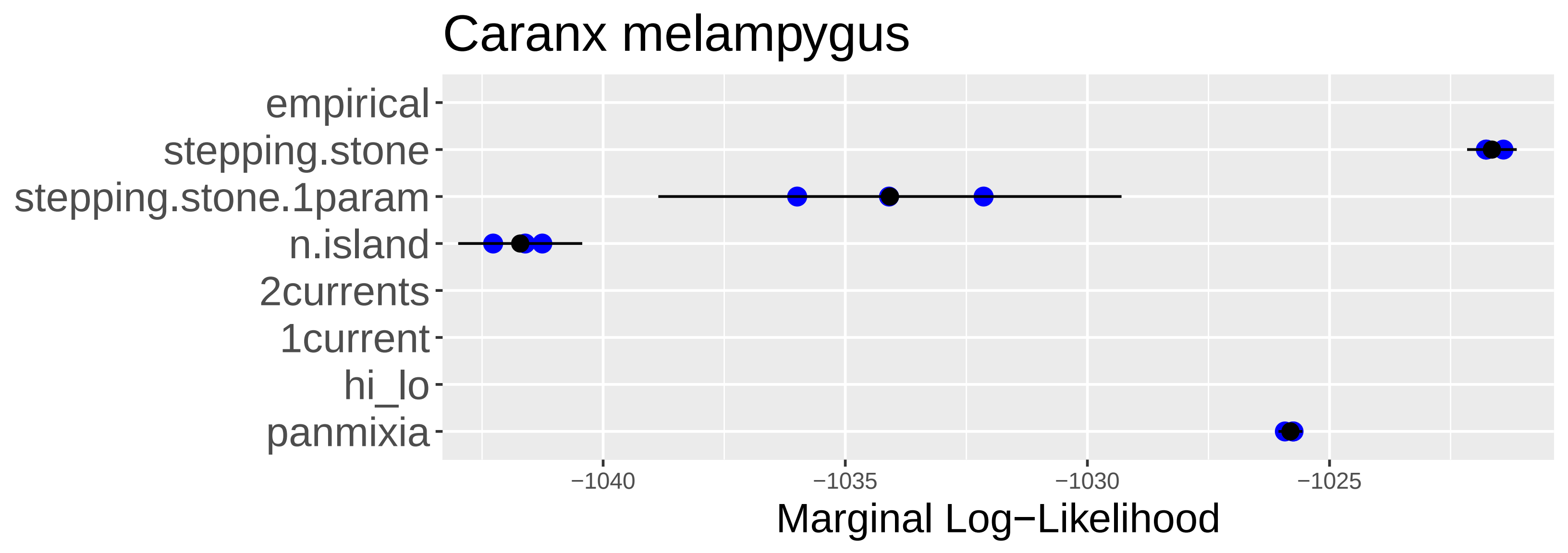
Caranx melampygus
empirical
stepping.stone
stepping.stone.1param
n.island
2currents
1current
hi_lo
panmixia
−1040
−1035
−1030
−1025
Marginal Log−Likelihood

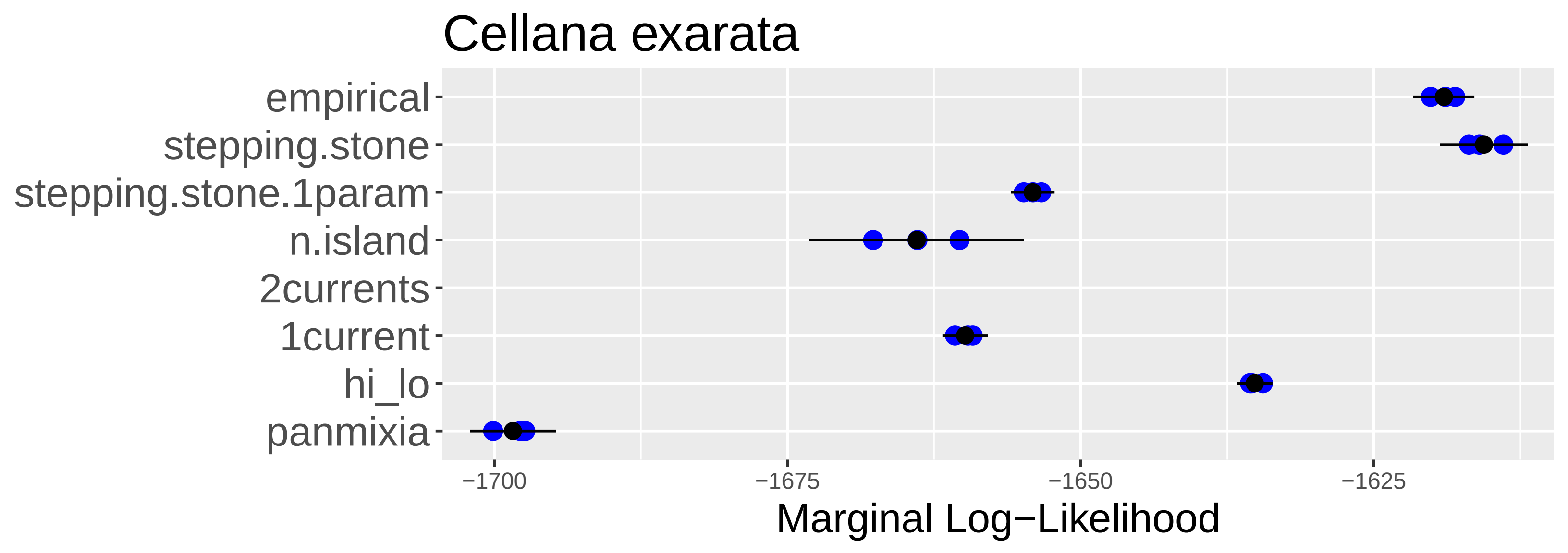
Cellana exarata
empirical
stepping.stone
stepping.stone.1param
n.island
2currents
1current
hi_lo
panmixia
−1700
−1675
−1650
−1625
Marginal Log−Likelihood

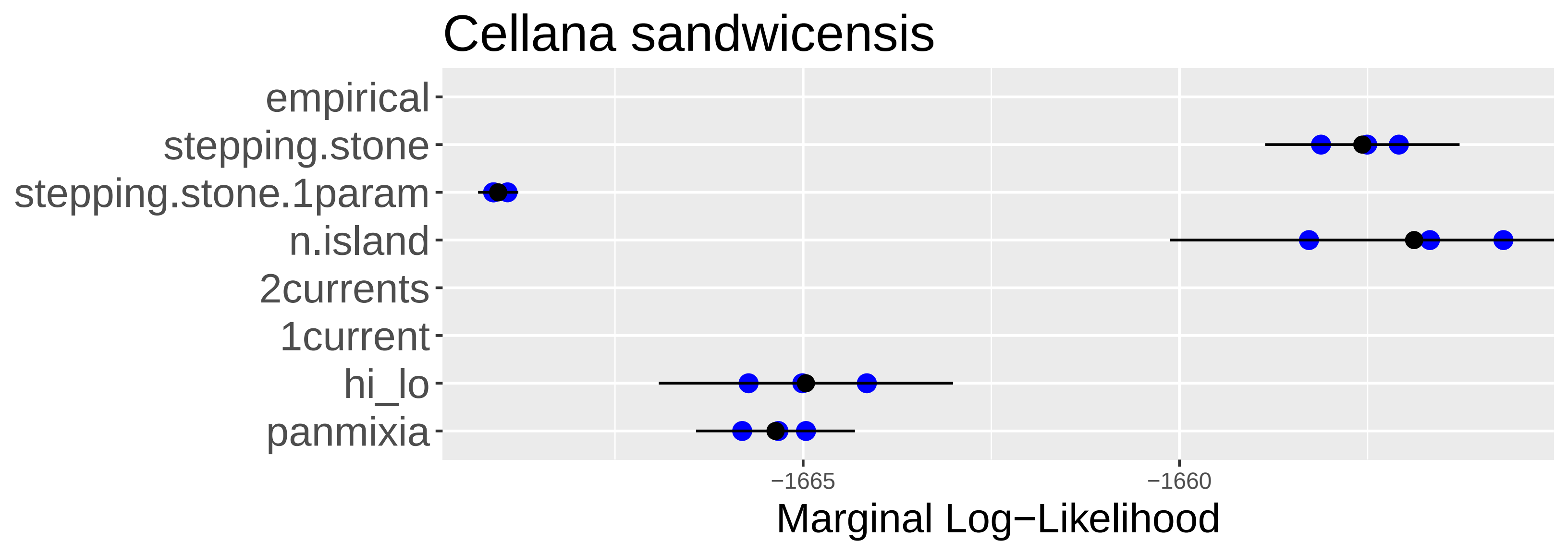
Cellana sandwicensis
empirical
stepping.stone
stepping.stone.1param
n.island
2currents
1current
hi_lo
panmixia
−1665
−1660
Marginal Log−Likelihood

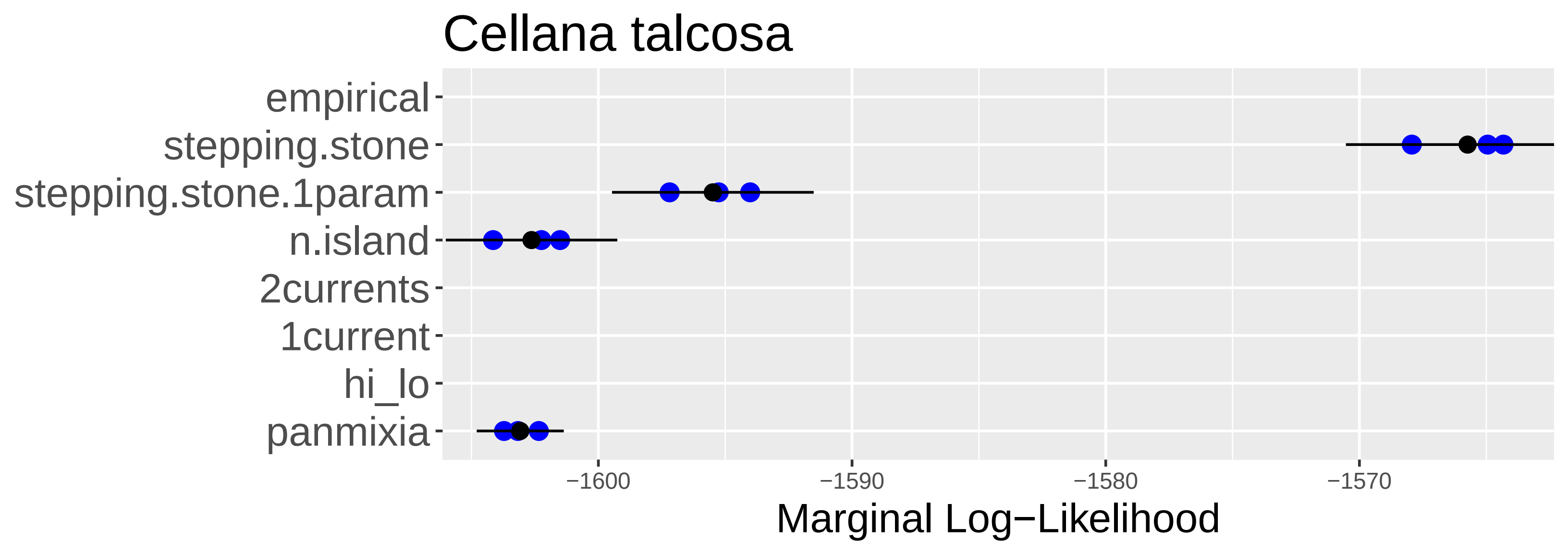
Cellana talcosa
empirical
stepping.stone
stepping.stone.1param
n.island
2currents
1current
hi_lo
panmixia
−1600
−1590
−1580
−1570
Marginal Log−Likelihood

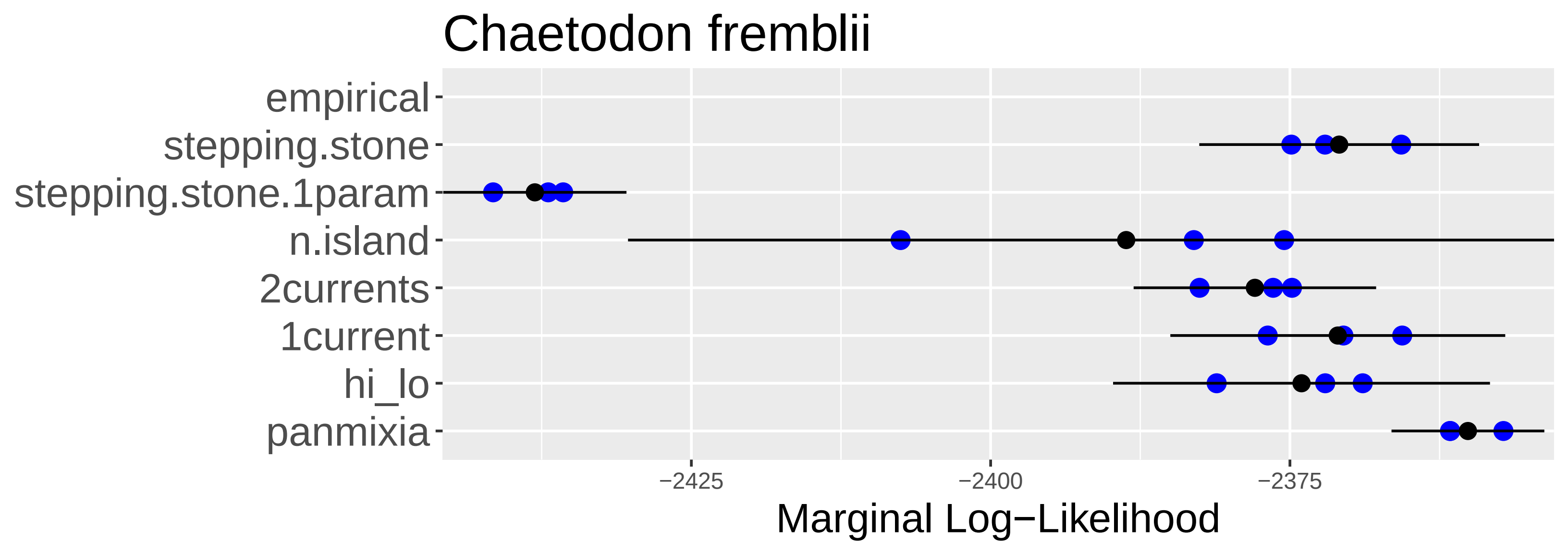

Chaetodon fremblii
empirical
stepping.stone
stepping.stone.1param
n.island
2currents
1current
hi_lo
panmixia
−2425
−2400
−2375
Marginal Log−Likelihood

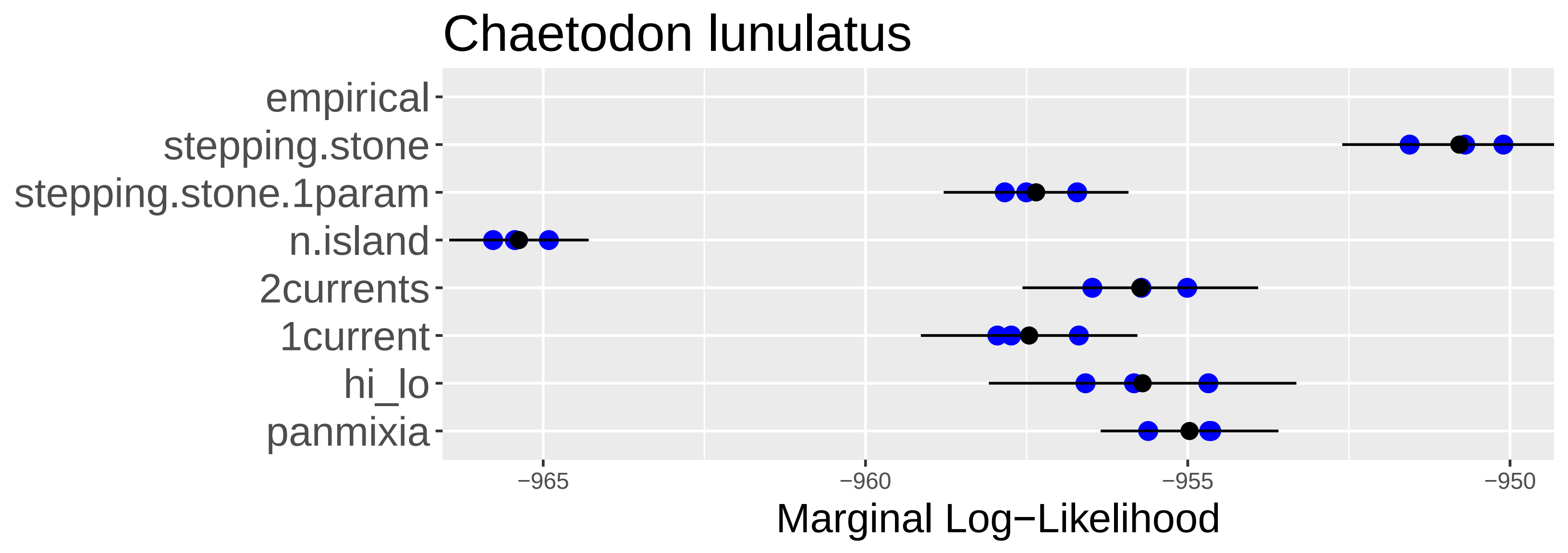

Chaetodon lunulatus
empirical
stepping.stone
stepping.stone.1param
n.island
2currents
1current
hi_lo
panmixia
−965
−960
−955
−950
Marginal Log−Likelihood

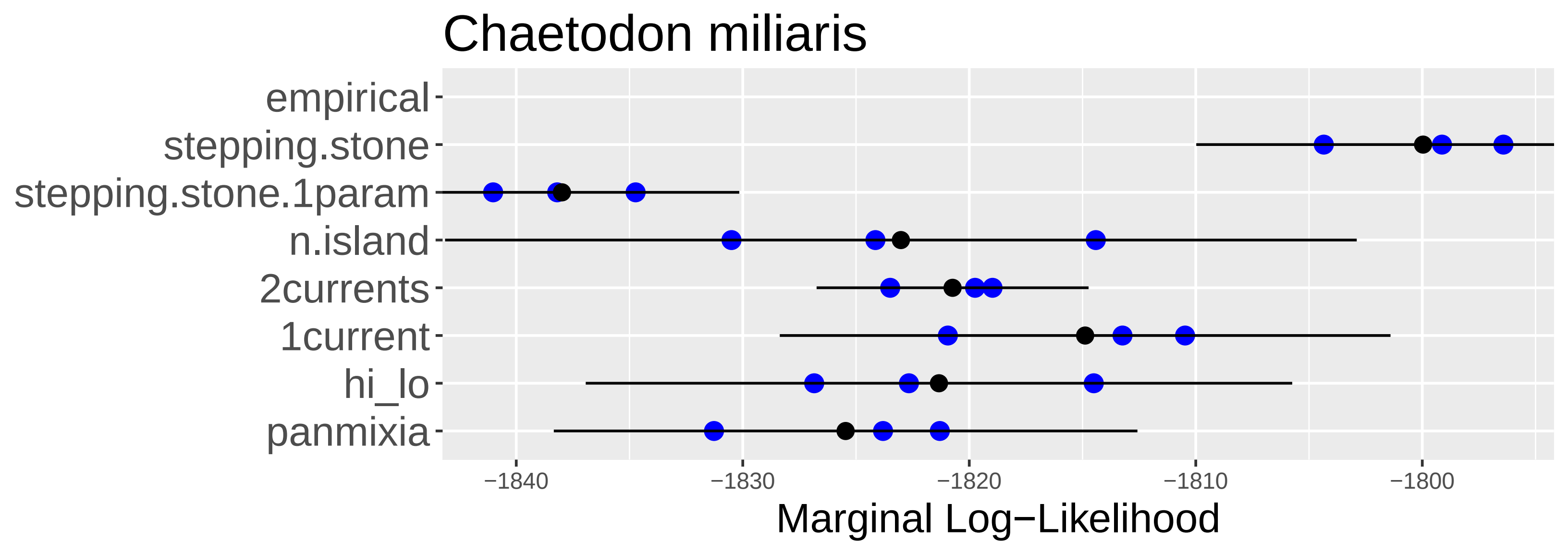

Chaetodon miliaris
empirical
stepping.stone
stepping.stone.1param
n.island
2currents
1current
hi_lo
panmixia
−1840
−1830
−1820
−1810
−1800
Marginal Log−Likelihood

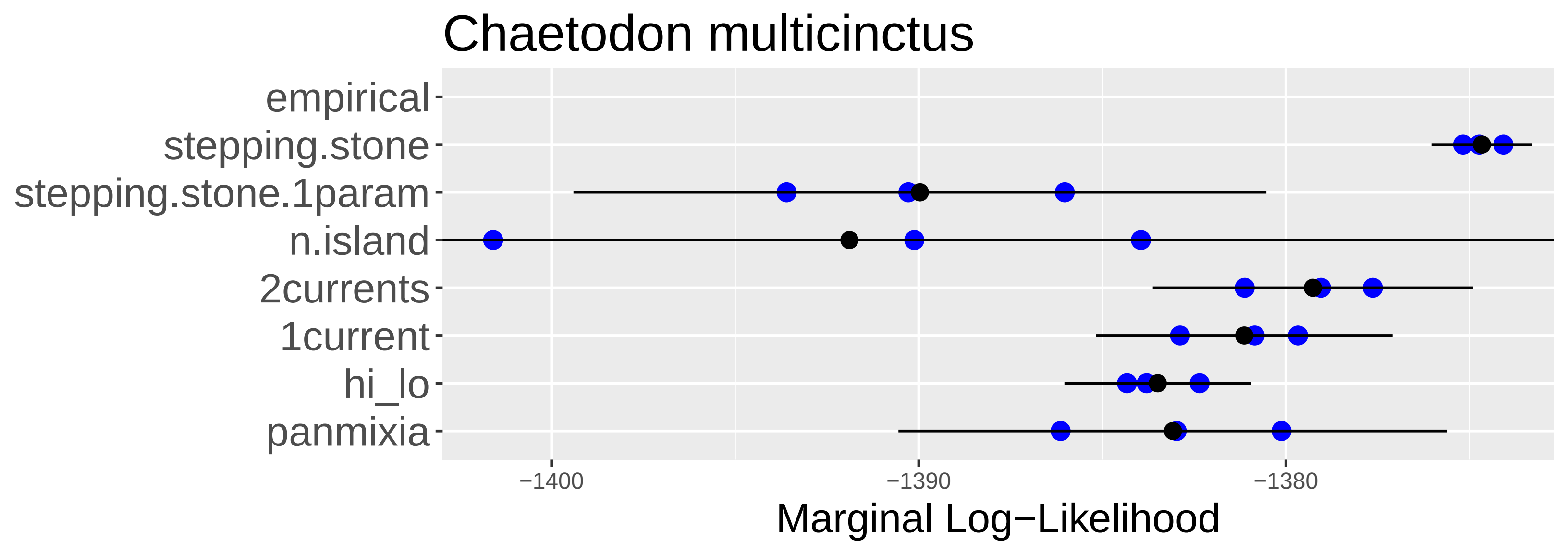

Chaetodon multicinctus
empirical
stepping.stone
stepping.stone.1param
n.island
2currents
1current
hi_lo
panmixia
−1400
−1390
−1380
Marginal Log−Likelihood

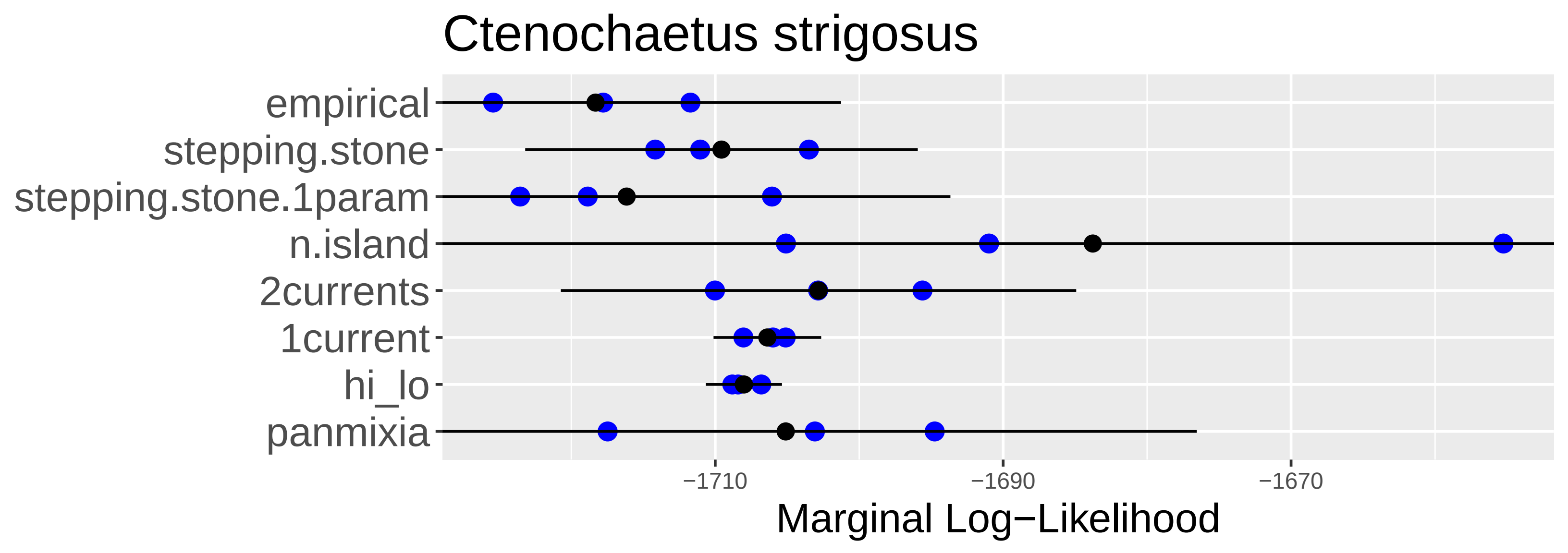
Ctenochaetus strigosus
empirical
stepping.stone
stepping.stone.1param
n.island
2currents
1current
hi_lo
panmixia
−1710
−1690
−1670
Marginal Log−Likelihood

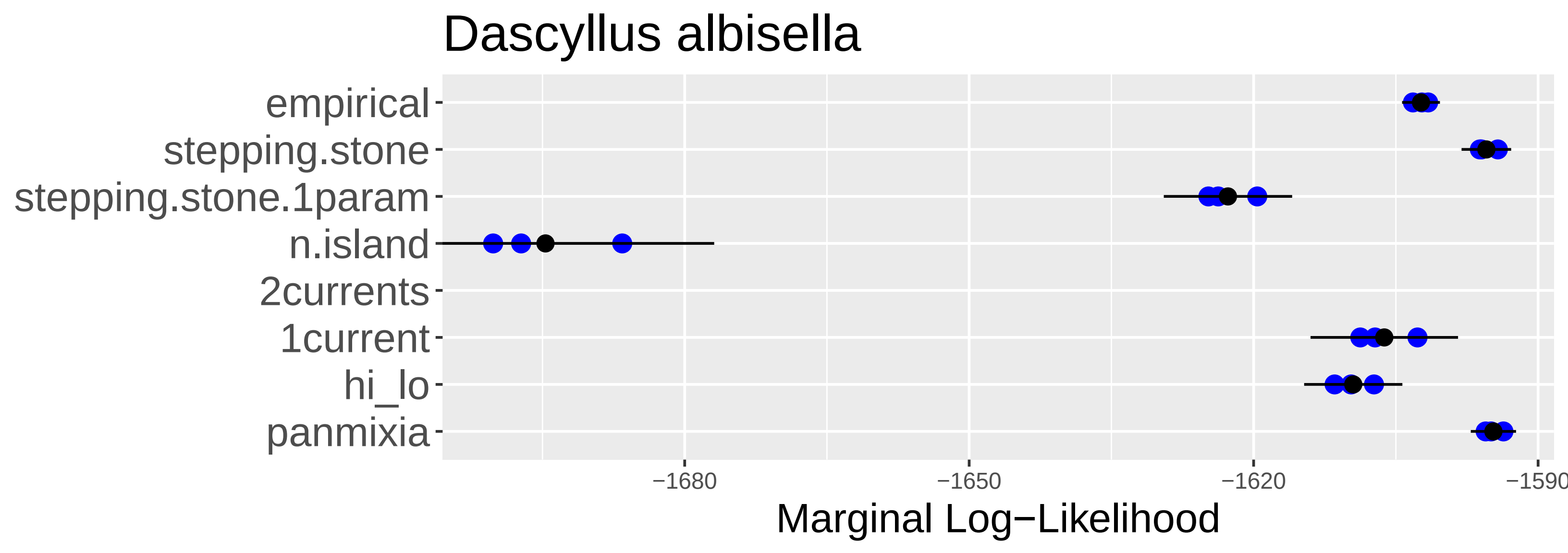
Dascyllus albisella
empirical
stepping.stone
stepping.stone.1param
n.island
2currents
1current
hi_lo
panmixia
−1680
−1650
−1620
−1590
Marginal Log−Likelihood

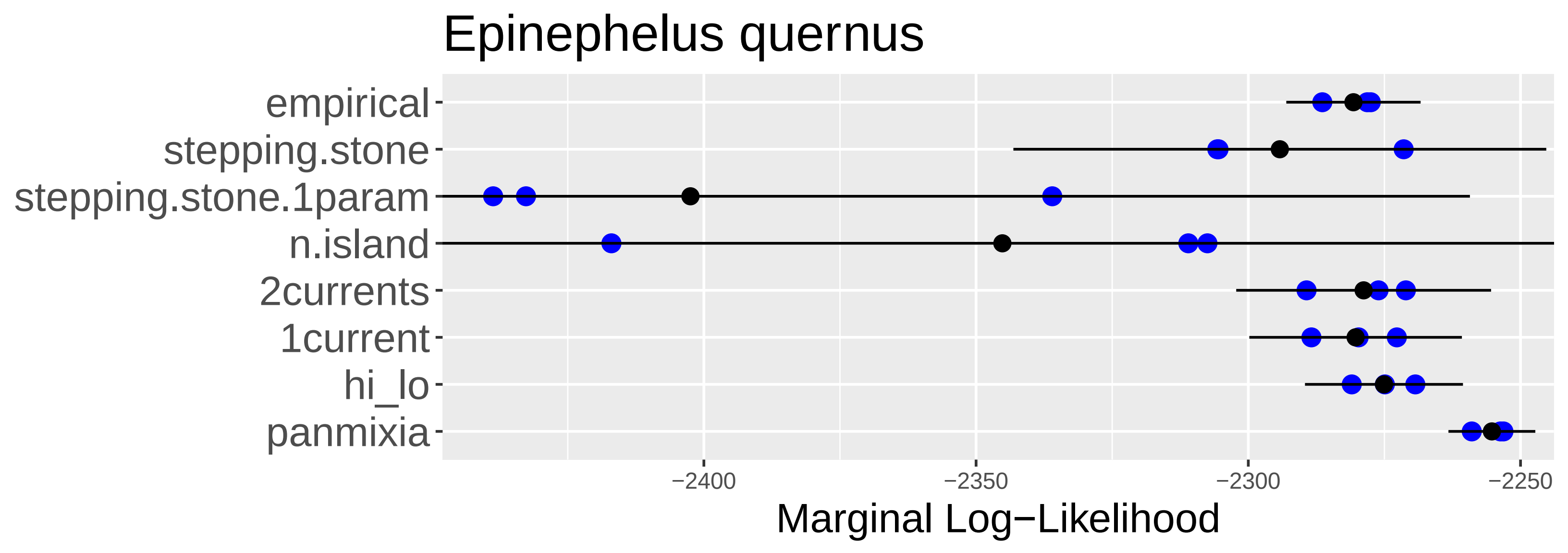

Epinephelus quernus
empirical
stepping.stone
stepping.stone.1param
n.island
2currents
1current
hi_lo
panmixia
−2400
−2350
−2300
−2250
Marginal Log−Likelihood

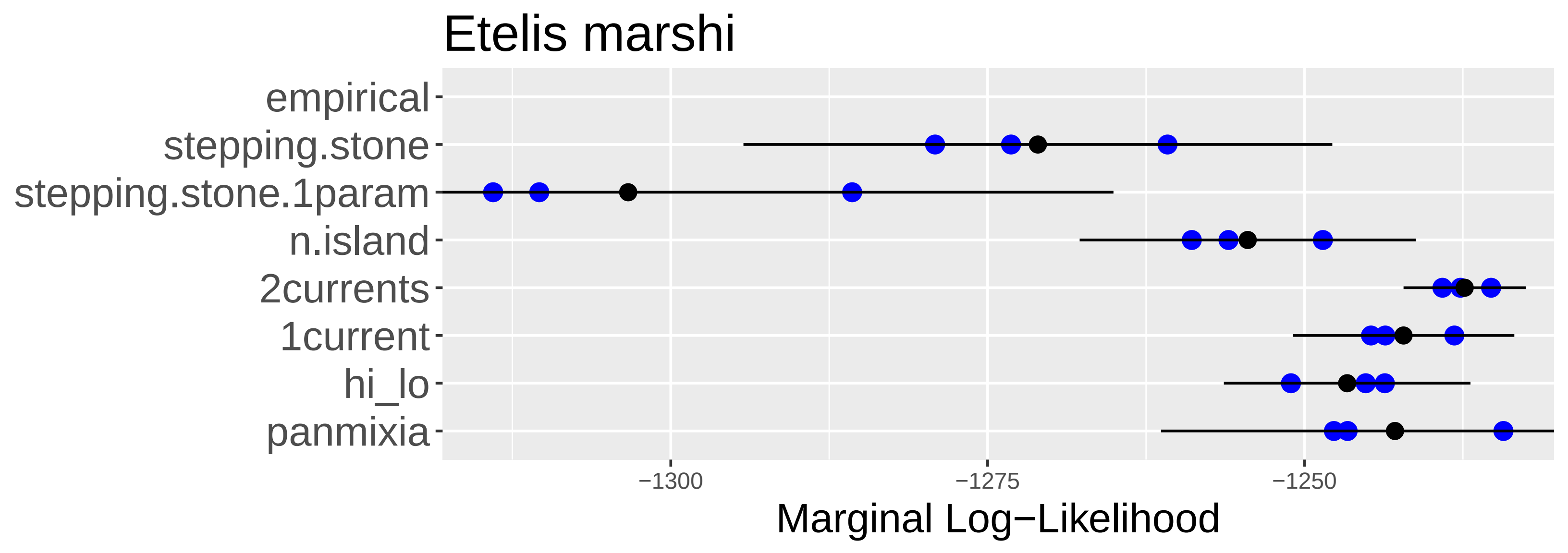
Etelis marshi
empirical
stepping.stone
stepping.stone.1param
n.island
2currents
1current
hi_lo
panmixia
−1300
−1275
−1250
Marginal Log−Likelihood

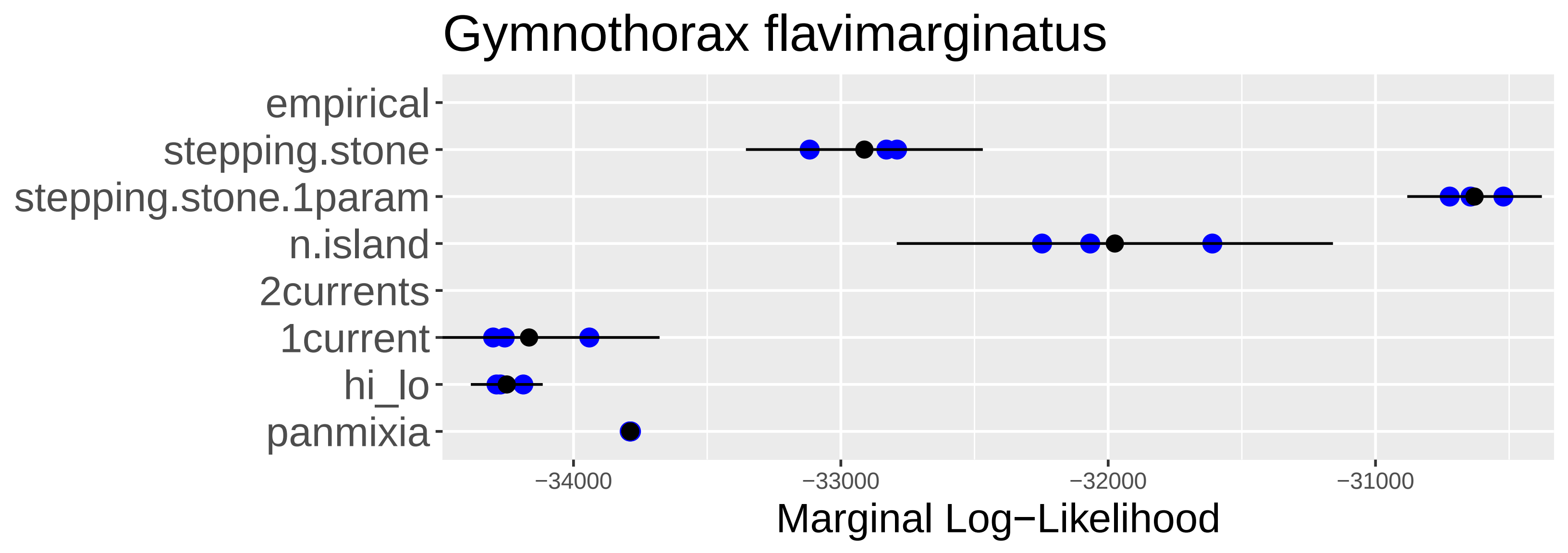
Gymnothorax flavimarginatus
empirical
stepping.stone
stepping.stone.1param
n.island
2currents
1current
hi_lo
panmixia
−34000
−33000
−32000
−31000
Marginal Log−Likelihood

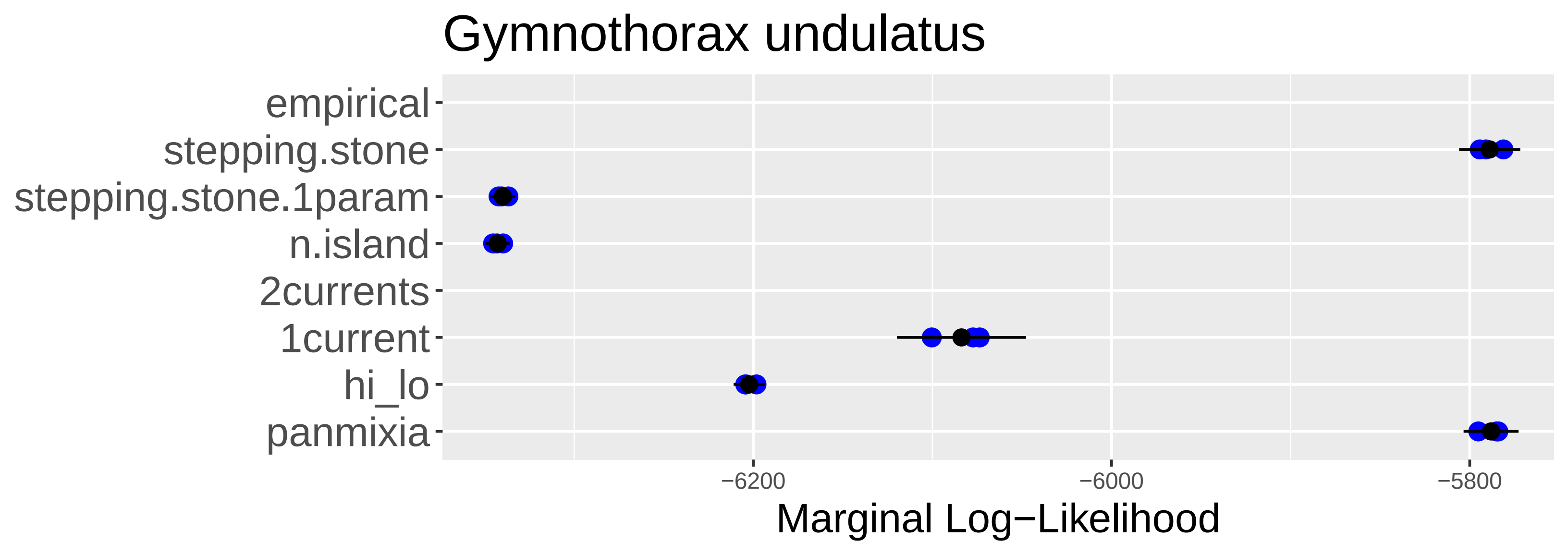

Gymnothorax undulatus
empirical
stepping.stone
stepping.stone.1param
n.island
2currents
1current
hi_lo
panmixia
−6200
−6000
−5800
Marginal Log−Likelihood

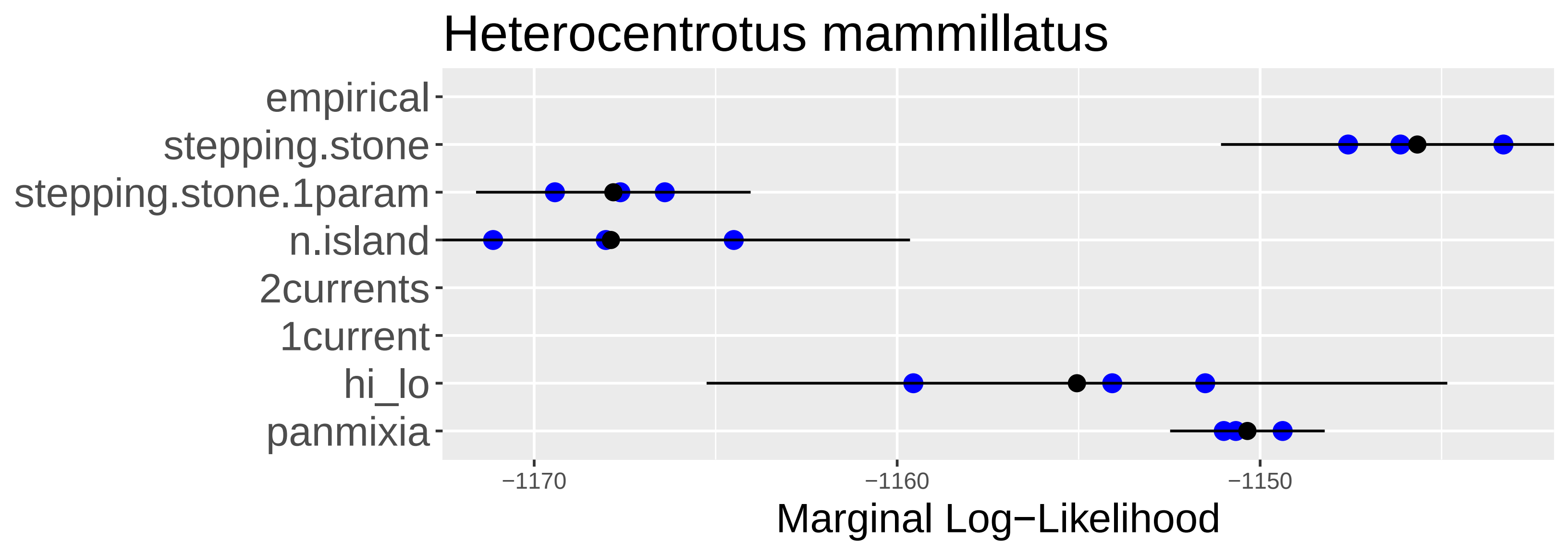

Heterocentrotus mammillatus
empirical
stepping.stone
stepping.stone.1param
n.island
2currents
1current
hi_lo
panmixia
−1170
−1160
−1150
Marginal Log−Likelihood

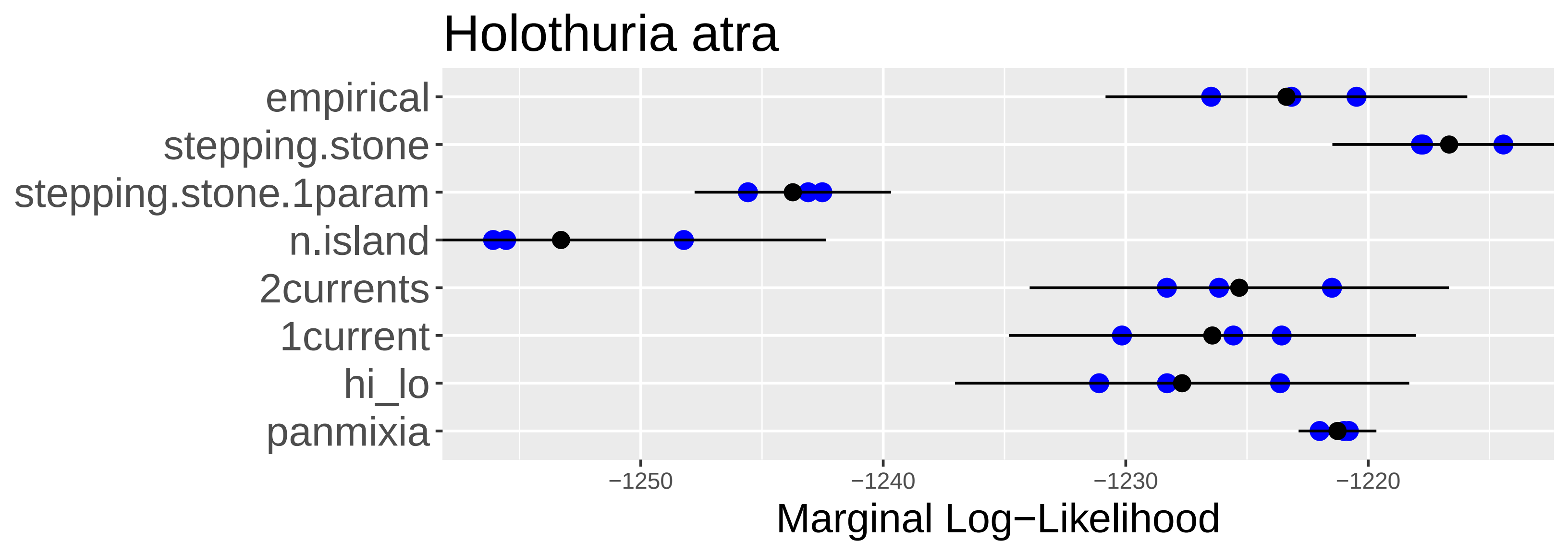

Holothuria atra
empirical
stepping.stone
stepping.stone.1param
n.island
2currents
1current
hi_lo
panmixia
−1250
−1240
−1230
−1220
Marginal Log−Likelihood

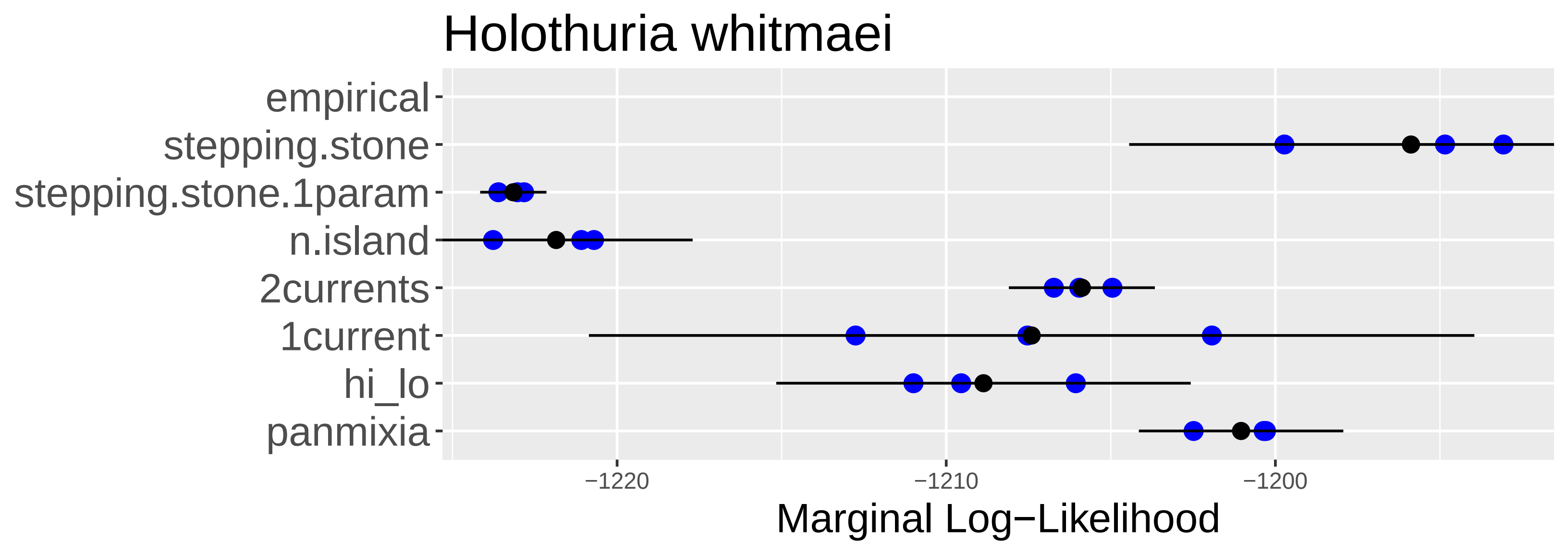
Holothuria whitmaei
empirical
stepping.stone
stepping.stone.1param
n.island
2currents
1current
hi_lo
panmixia
−1220
−1210
−1200
Marginal Log−Likelihood

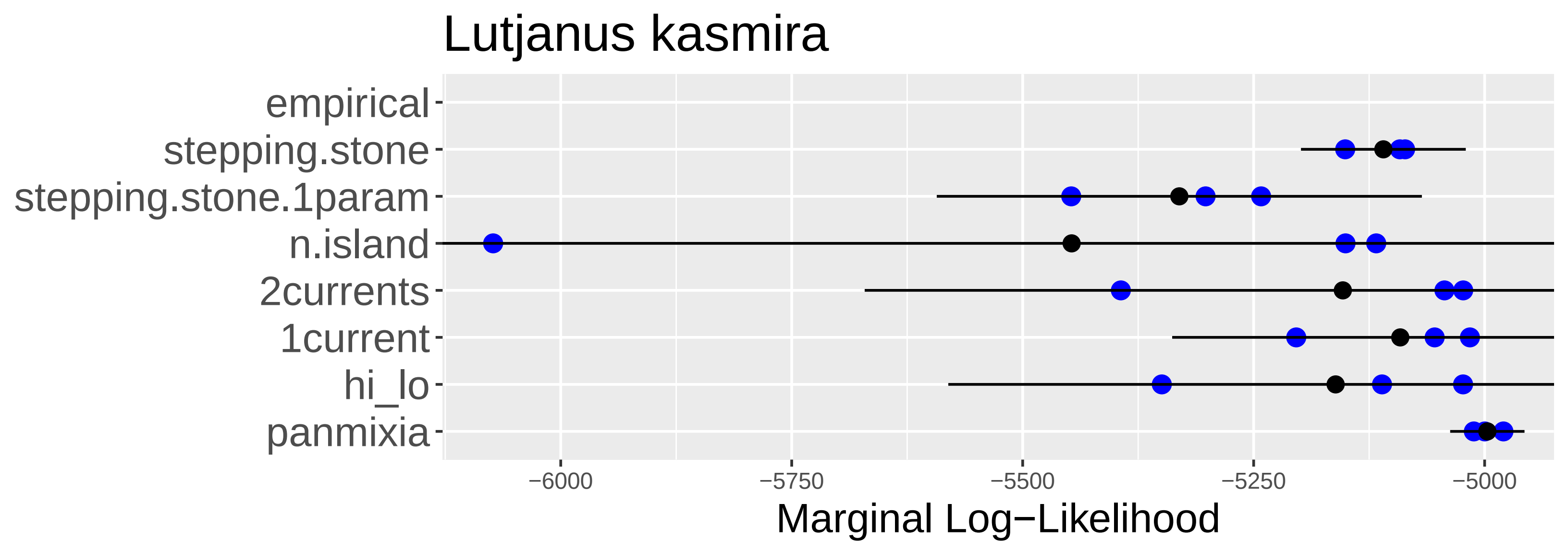

Lutjanus kasmira
empirical
stepping.stone
stepping.stone.1param
n.island
2currents
1current
hi_lo
panmixia
−6000
−5750
−5500
−5250
−5000
Marginal Log−Likelihood

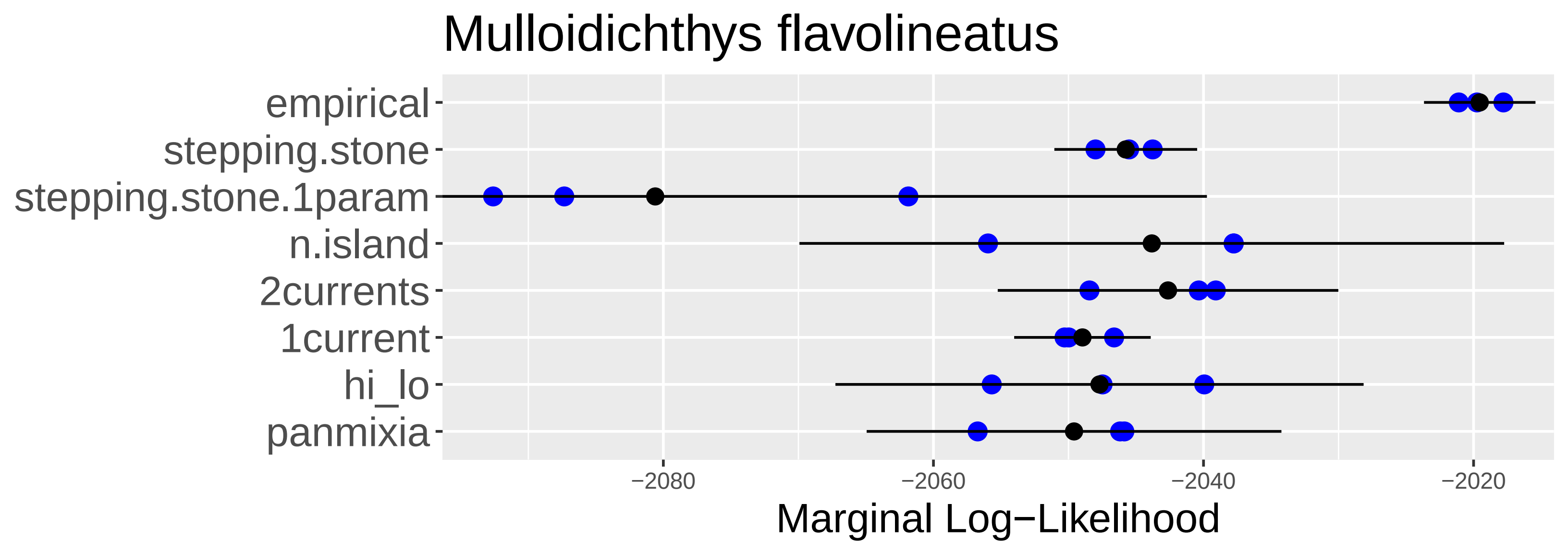

Mulloidichthys flavolineatus
empirical
stepping.stone
stepping.stone.1param
n.island
2currents
1current
hi_lo
panmixia
−2080
−2060
−2040
−2020
Marginal Log−Likelihood

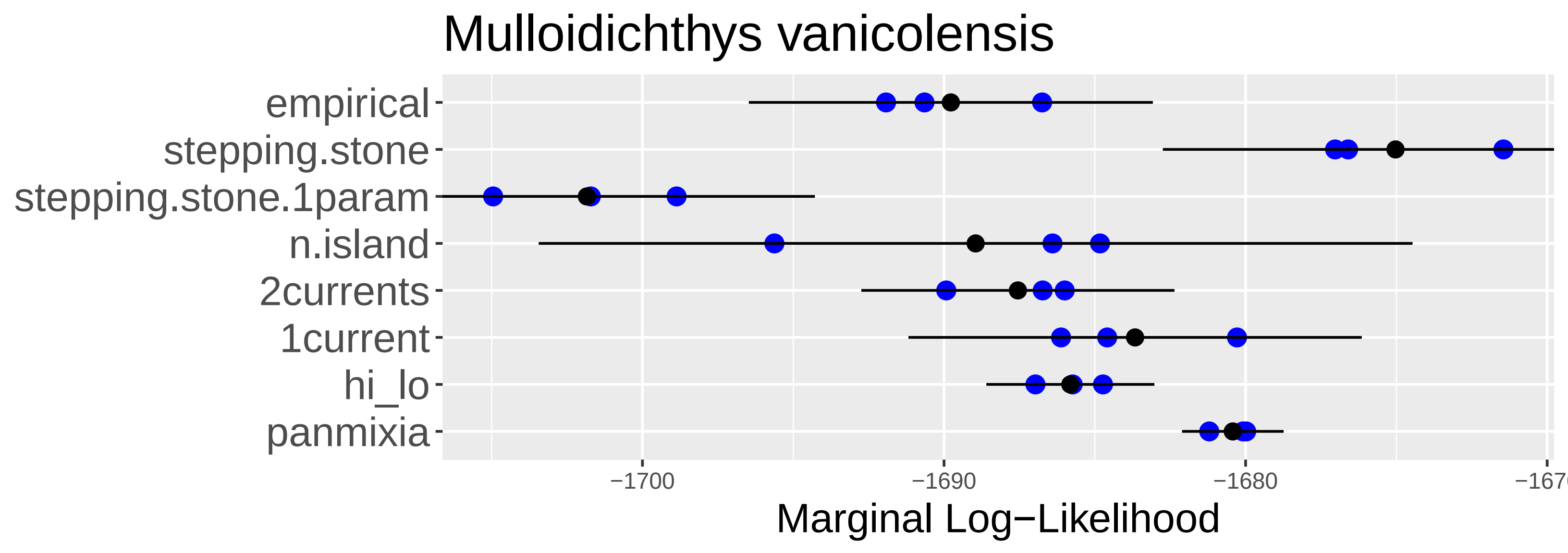

Mulloidichthys vanicolensis
empirical
stepping.stone
stepping.stone.1param
n.island
2currents
1current
hi_lo
panmixia
−1700
−1690
−1680
Marginal Log−Likelihood

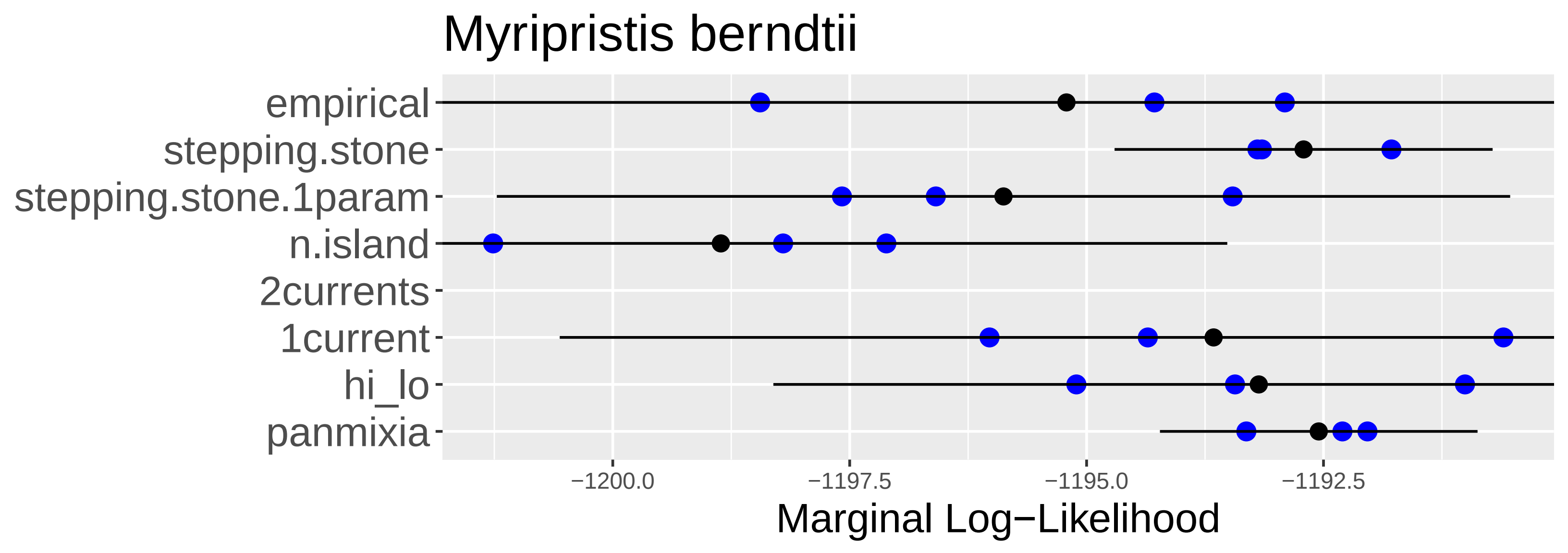

Myripristis berndtii
empirical
stepping.stone
stepping.stone.1param
n.island
2currents
1current
hi_lo
panmixia
−1200.0
−1197.5
−1195.0
−1192.5
Marginal Log−Likelihood

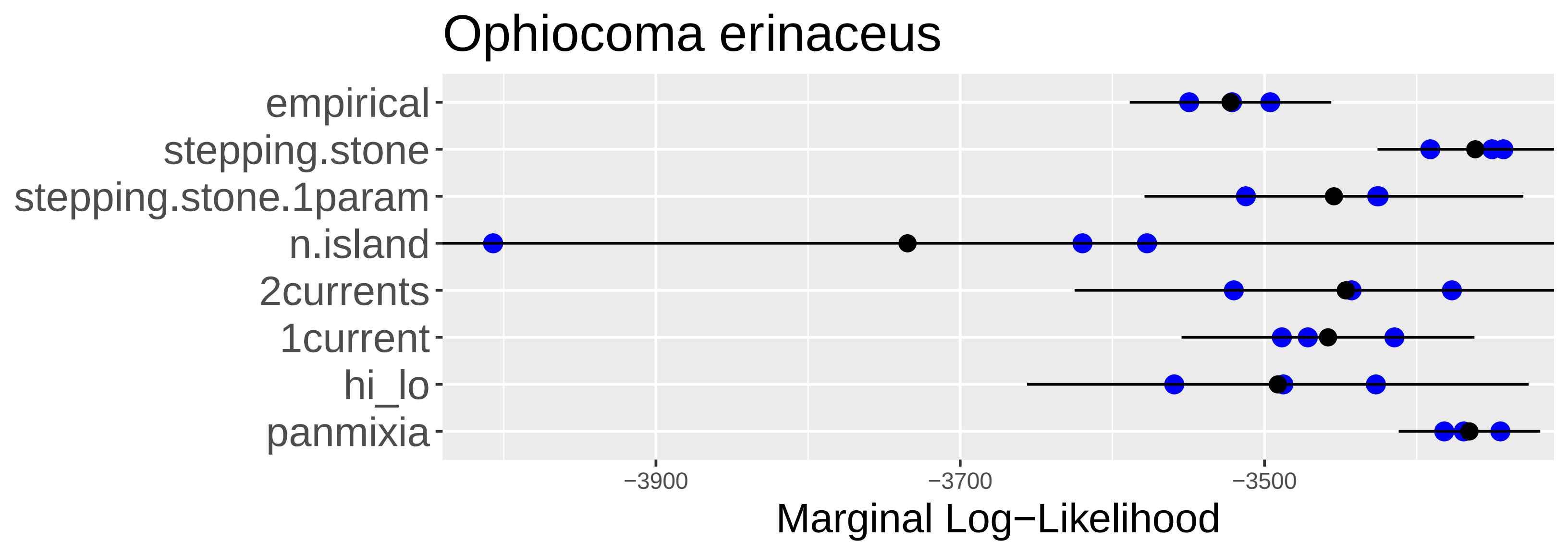

Ophiocoma erinaceus
empirical
stepping.stone
stepping.stone.1param
n.island
2currents
1current
hi_lo
panmixia
−3900
−3700
−3500
Marginal Log−Likelihood

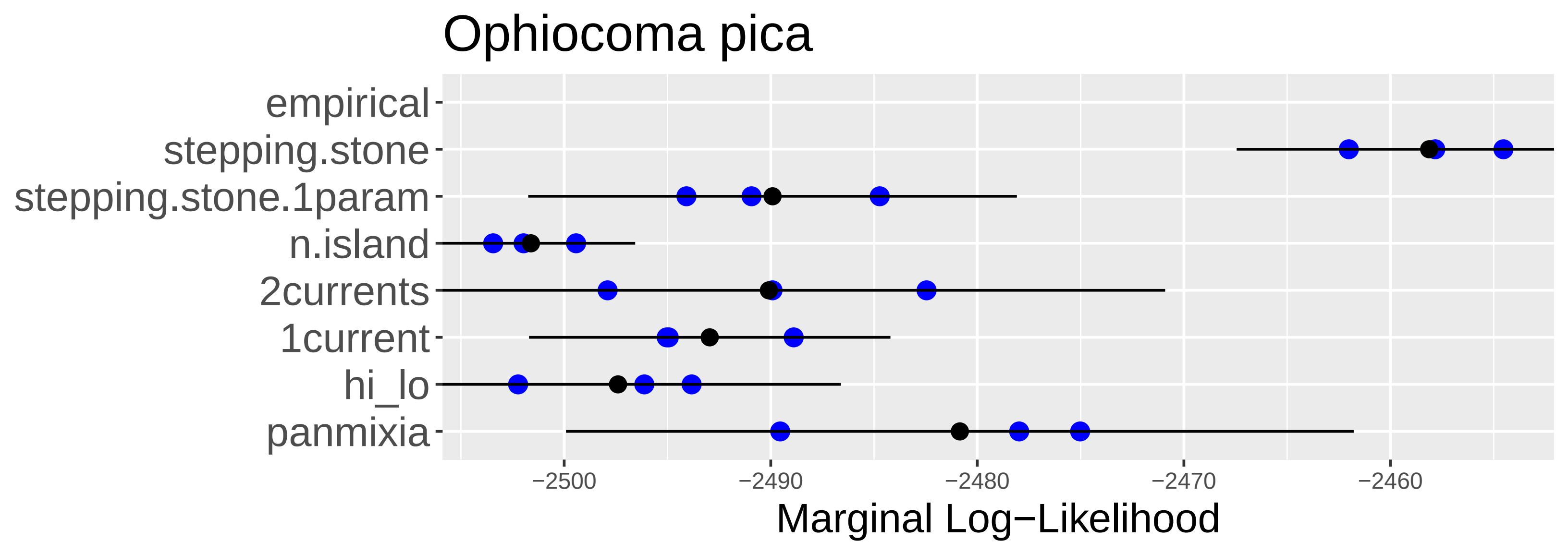

Ophiocoma pica
empirical
stepping.stone
stepping.stone.1param
n.island
2currents
1current
hi_lo
panmixia
−2500
−2490
−2480
−2470
−2460
Marginal Log−Likelihood

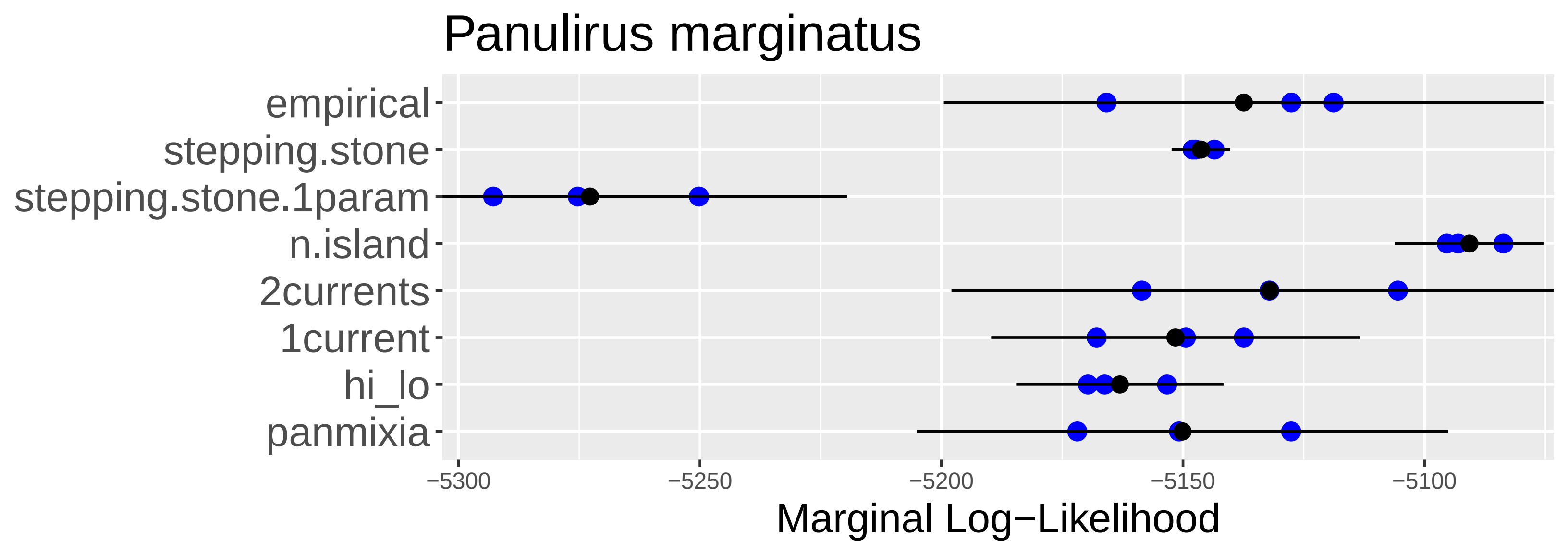

Panulirus marginatus
empirical
stepping.stone
stepping.stone.1param
n.island
2currents
1current
hi_lo
panmixia
−5300
−5250
−5200
−5150
−5100
Marginal Log−Likelihood

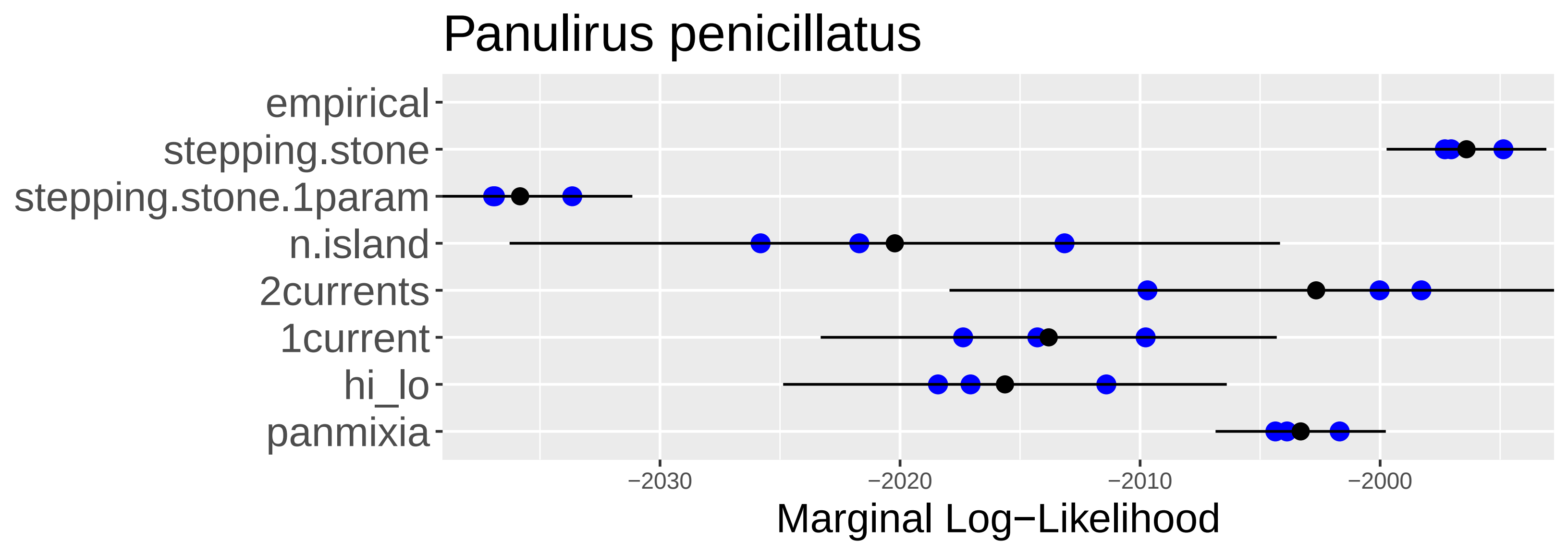
Panulirus penicillatus
empirical
stepping.stone
stepping.stone.1param
n.island
2currents
1current
hi_lo
panmixia
−2030
−2020
−2010
−2000
Marginal Log−Likelihood

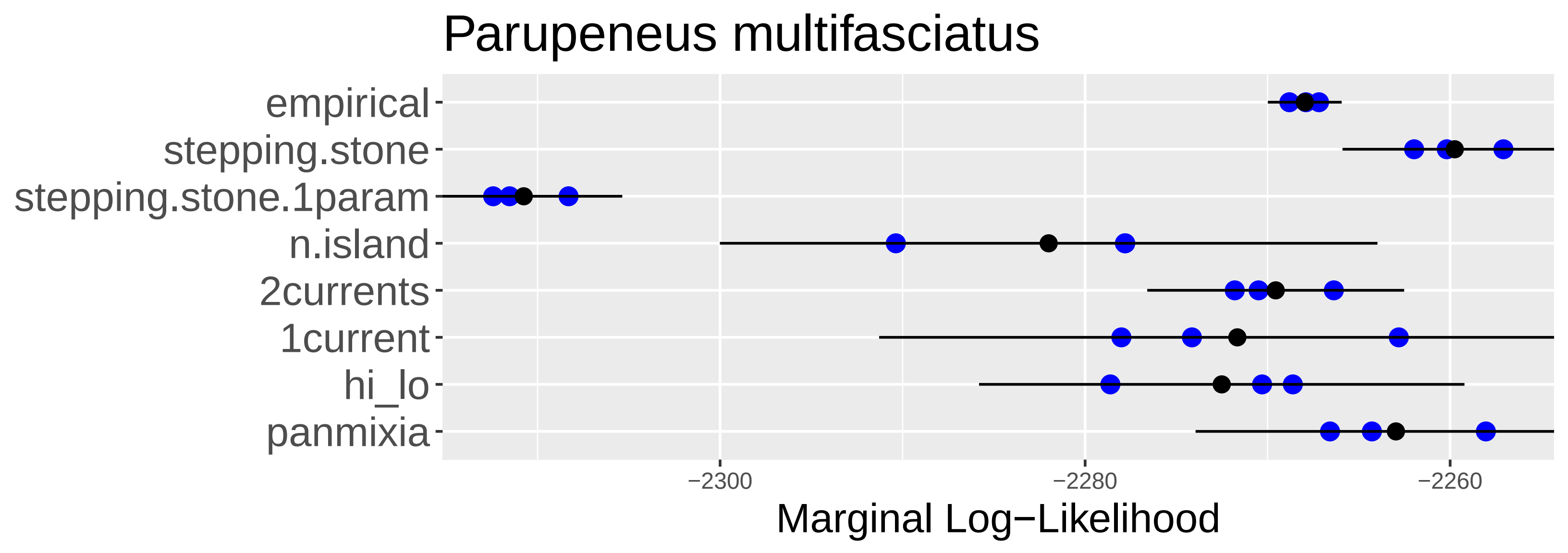
Parupeneus multifasciatus
empirical
stepping.stone
stepping.stone.1param
n.island
2currents
1current
hi_lo
panmixia
−2300
−2280
−2260
Marginal Log−Likelihood

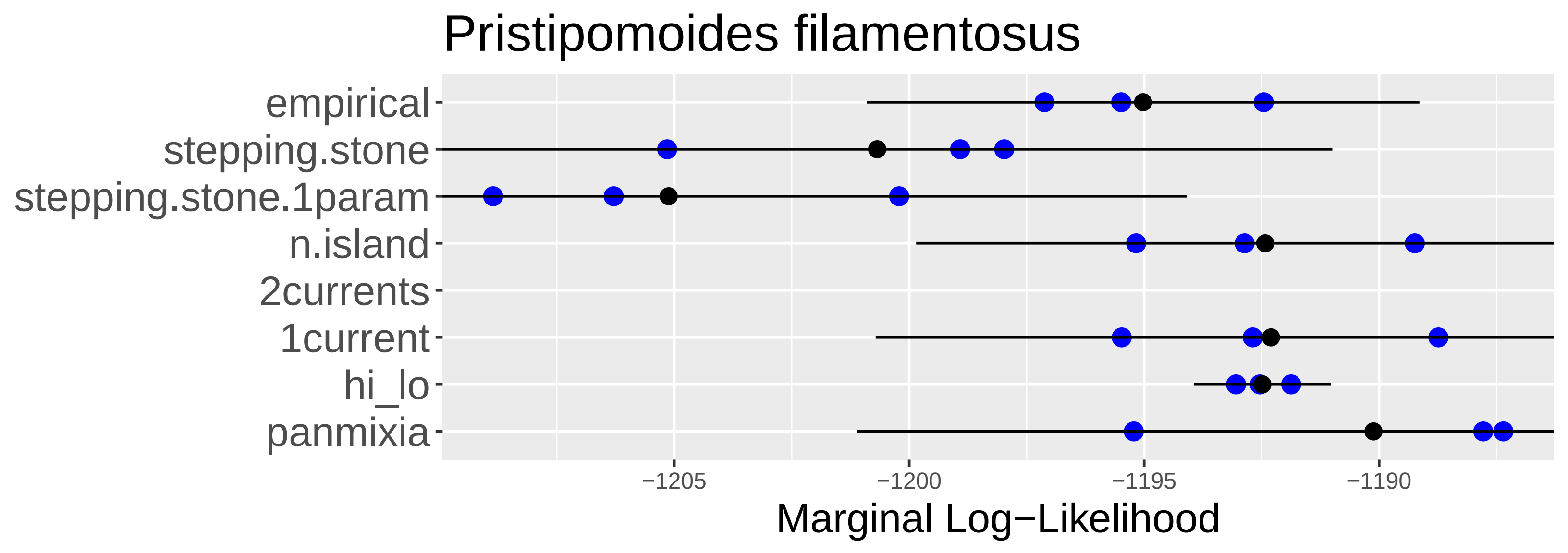

Pristipomoides filamentosus
empirical
stepping.stone
stepping.stone.1param
n.island
2currents
1current
hi_lo
panmixia
−1205
−1200
−1195
−1190
Marginal Log−Likelihood

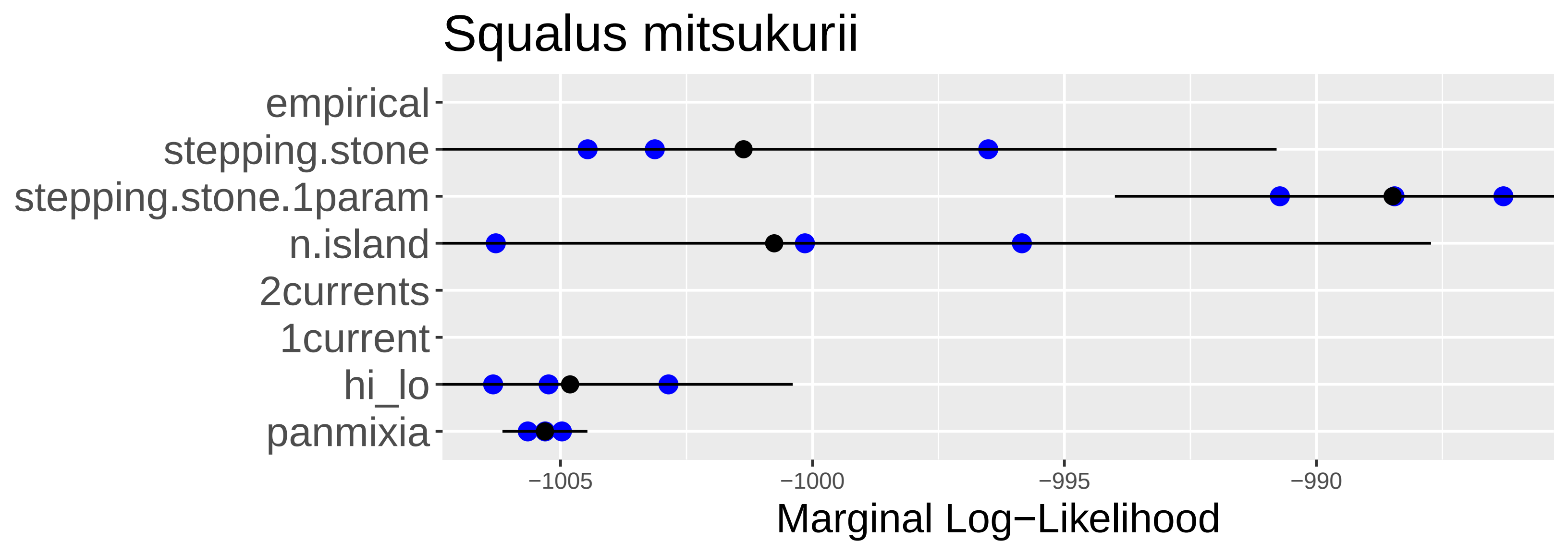

Squalus mitsukurii
empirical
stepping.stone
stepping.stone.1param
n.island
2currents
1current
hi_lo
panmixia
−1005
−1000
−995
−990
Marginal Log−Likelihood

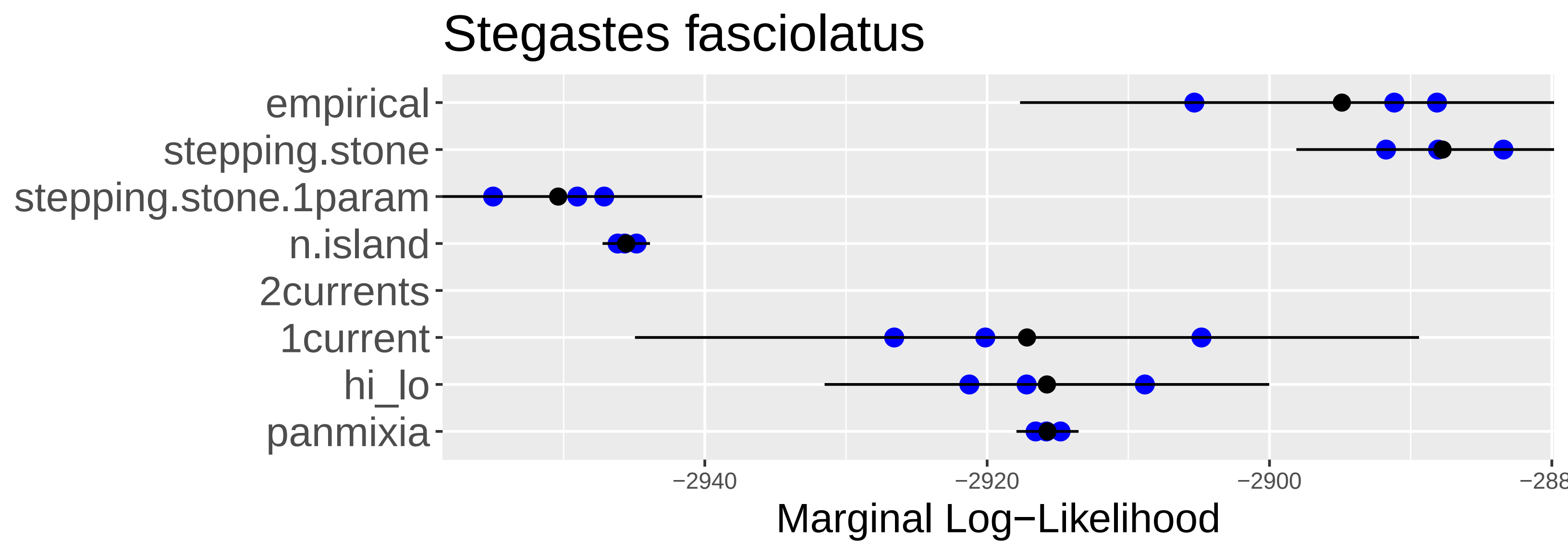
Stegastes fasciolatus
empirical
stepping.stone
stepping.stone.1param
n.island
2currents
1current
hi_lo
panmixia
−2940
−2920
−2900
Marginal Log−Likelihood

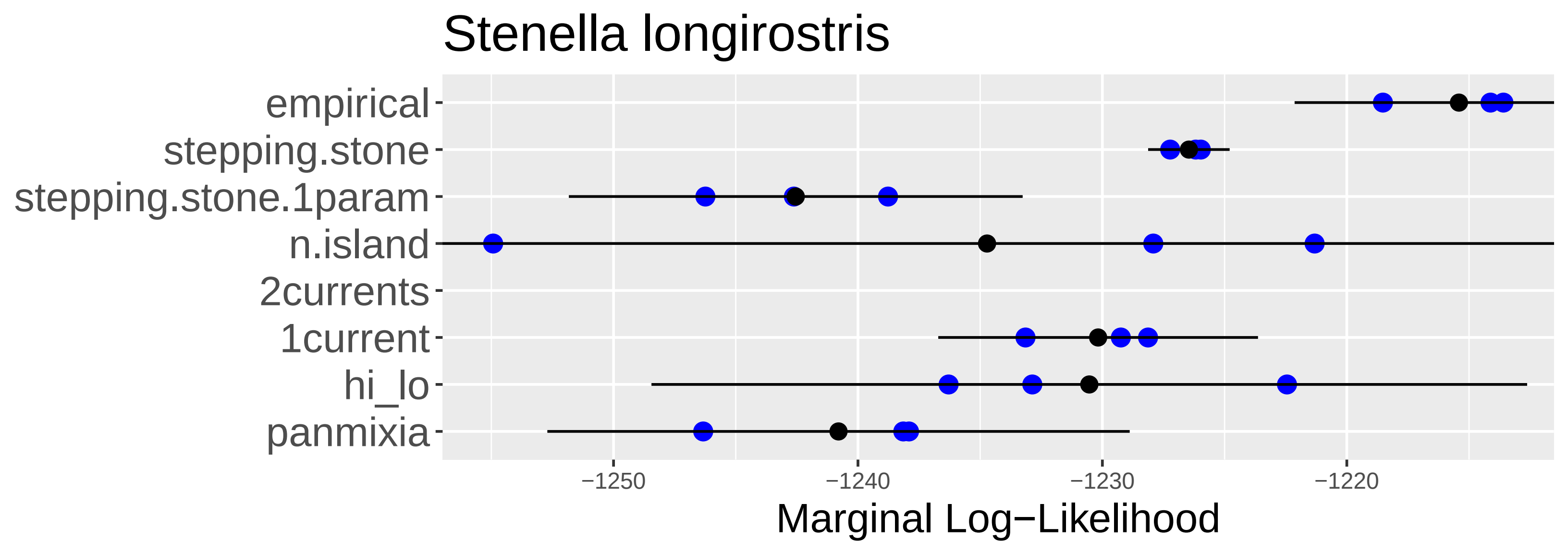

Stenella longirostris
empirical
stepping.stone
stepping.stone.1param
n.island
2currents
1current
hi_lo
panmixia
−1250
−1240
−1230
−1220
Marginal Log−Likelihood

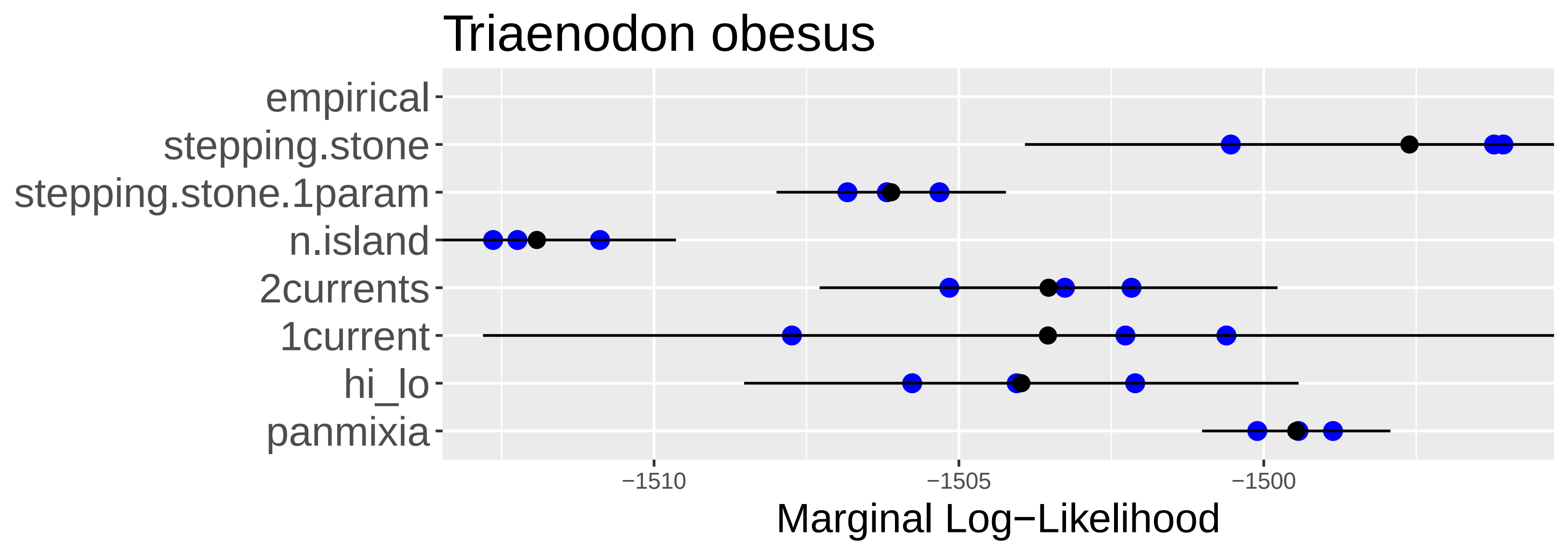

Triaenodon obesus
empirical
stepping.stone
stepping.stone.1param
n.island
2currents
1current
hi_lo
panmixia
−1510
−1505
−1500
Marginal Log−Likelihood

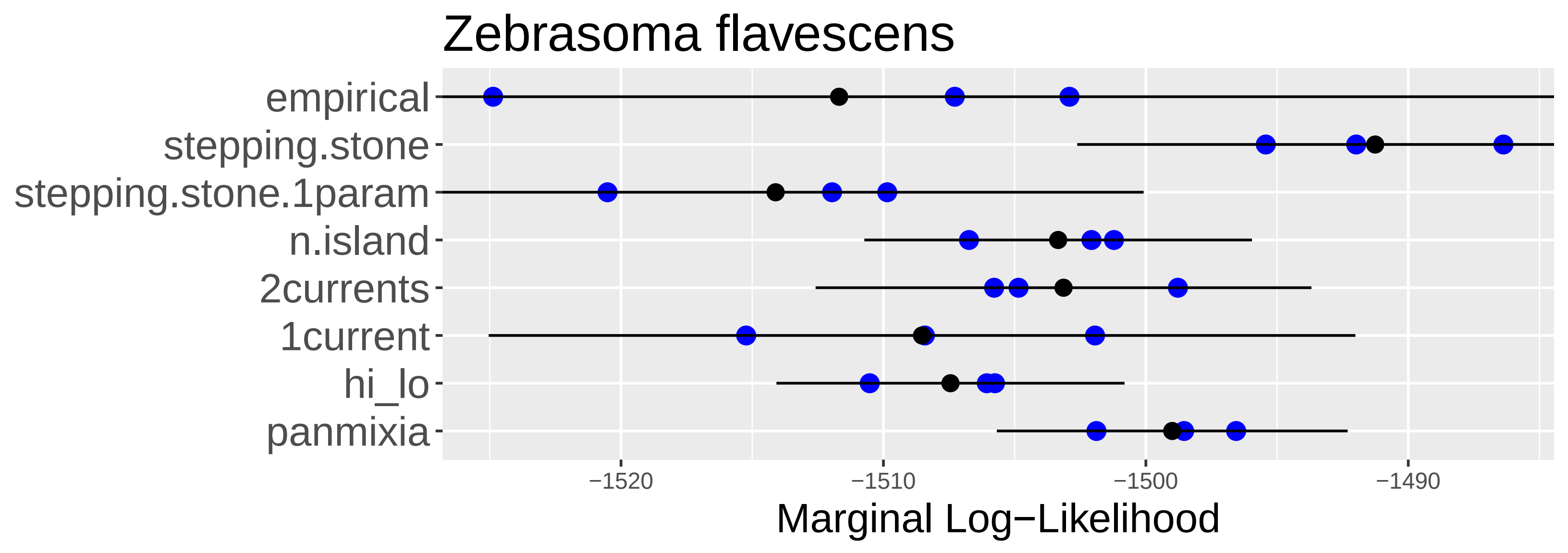
Zebrasoma flavescens
empirical
stepping.stone
stepping.stone.1param
n.island
2currents
1current
hi_lo
panmixia
−1520
−1510
−1500
−1490
Marginal Log−Likelihood
