# Supplementary figures and images for: A coalescent sampler successfully detects biologically meaningful population structure overlooked by F‐statistics
Source: Evol Appl. 2018 Oct 15;12(2):255–65. doi: 10.1111/eva.12712 (PMC6346657; doi:10.1111/eva.12712)

Effective Female Migrants per Generation

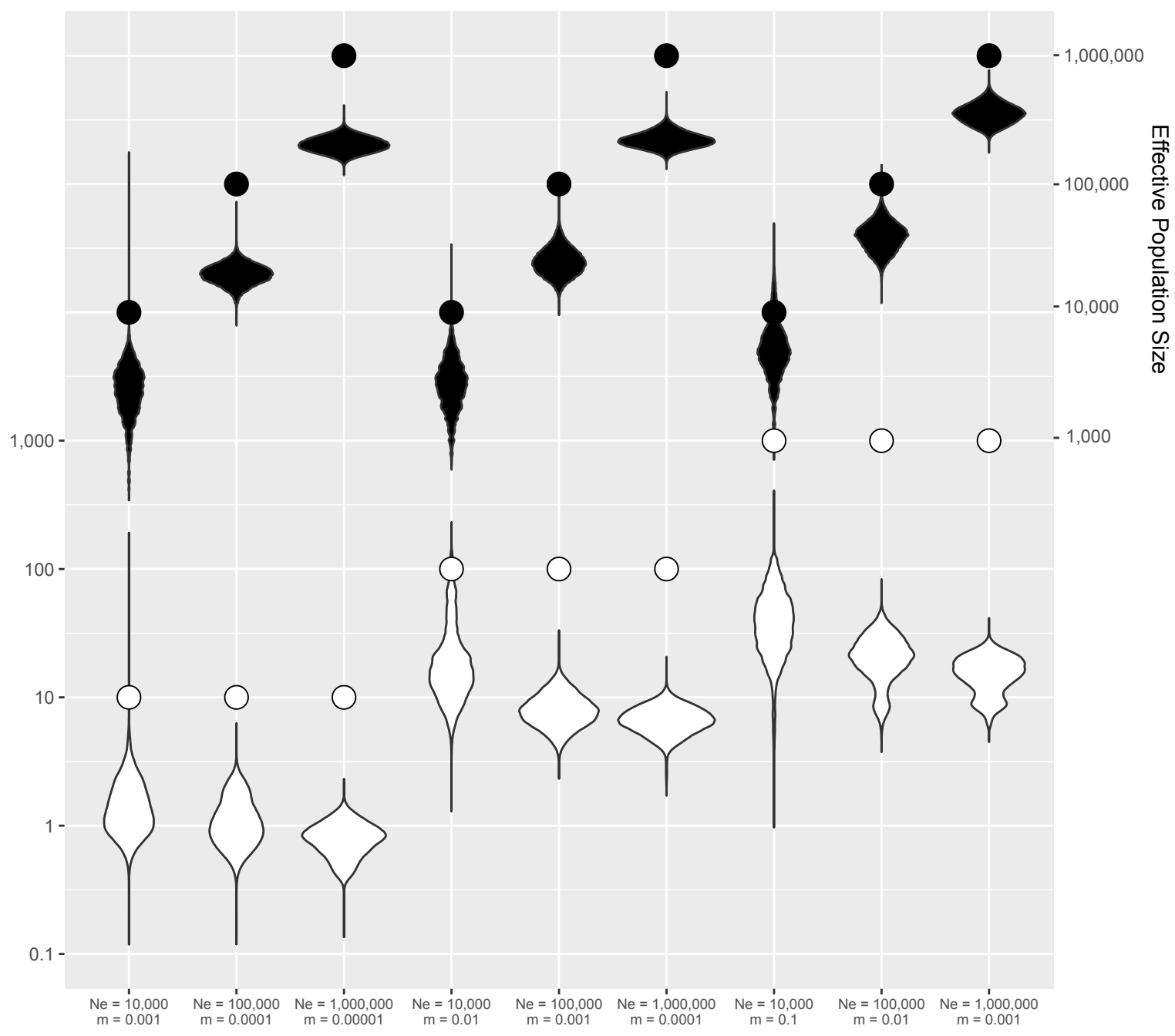

Supplement: Supplementary file 2 [file EVA-12-255-s002.pdf]
