# Supplementary material for: A coalescent sampler successfully detects biologically meaningful population structure overlooked by F‐statistics
Source: Evol Appl. 2018 Oct 15;12(2):255–65. doi: 10.1111/eva.12712 (PMC6346657; doi:10.1111/eva.12712)
Supplement: Supplementary file 3 [file EVA-12-255-s003.pdf]

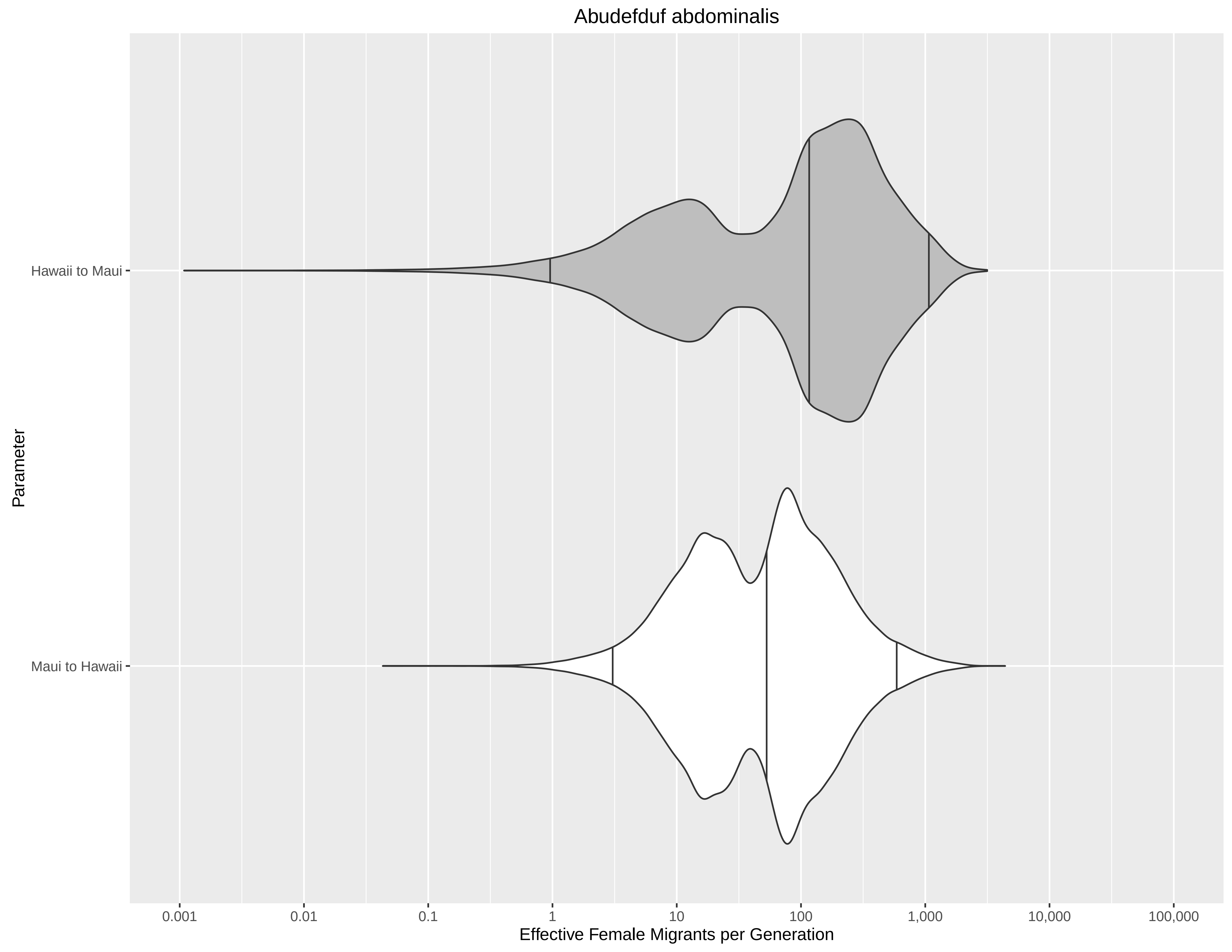

Abudefduf abdominalis
Parameter
Hawaii to Maui
Maui to Hawaii
0.001
0.01
0.1
1
10
100
1,000
10,000
100,000
Effective Female Migrants per Generation

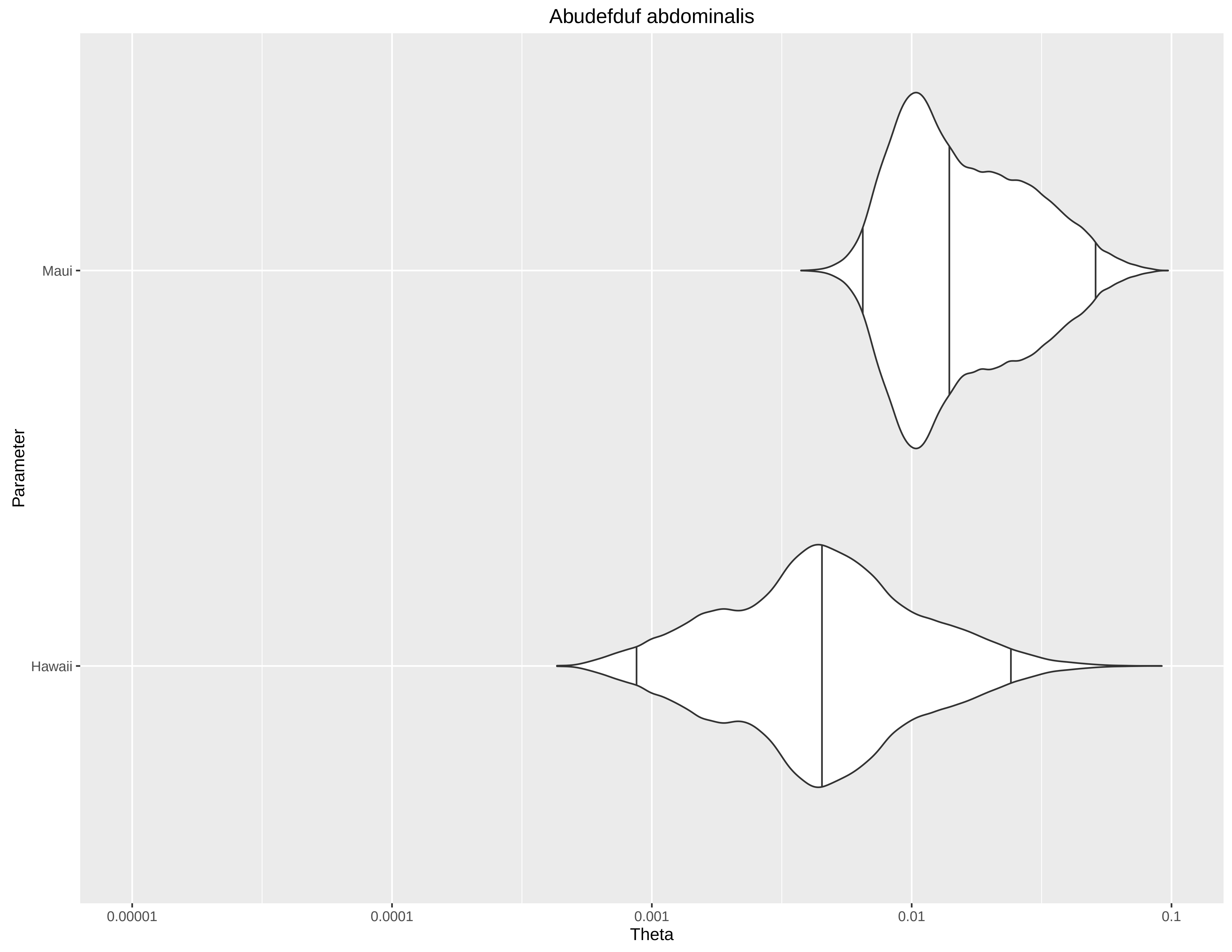
Abudefduf abdominalis
Maui
Hawaii
Parameter
0.00001
0.0001
0.001
0.01
0.1
Theta

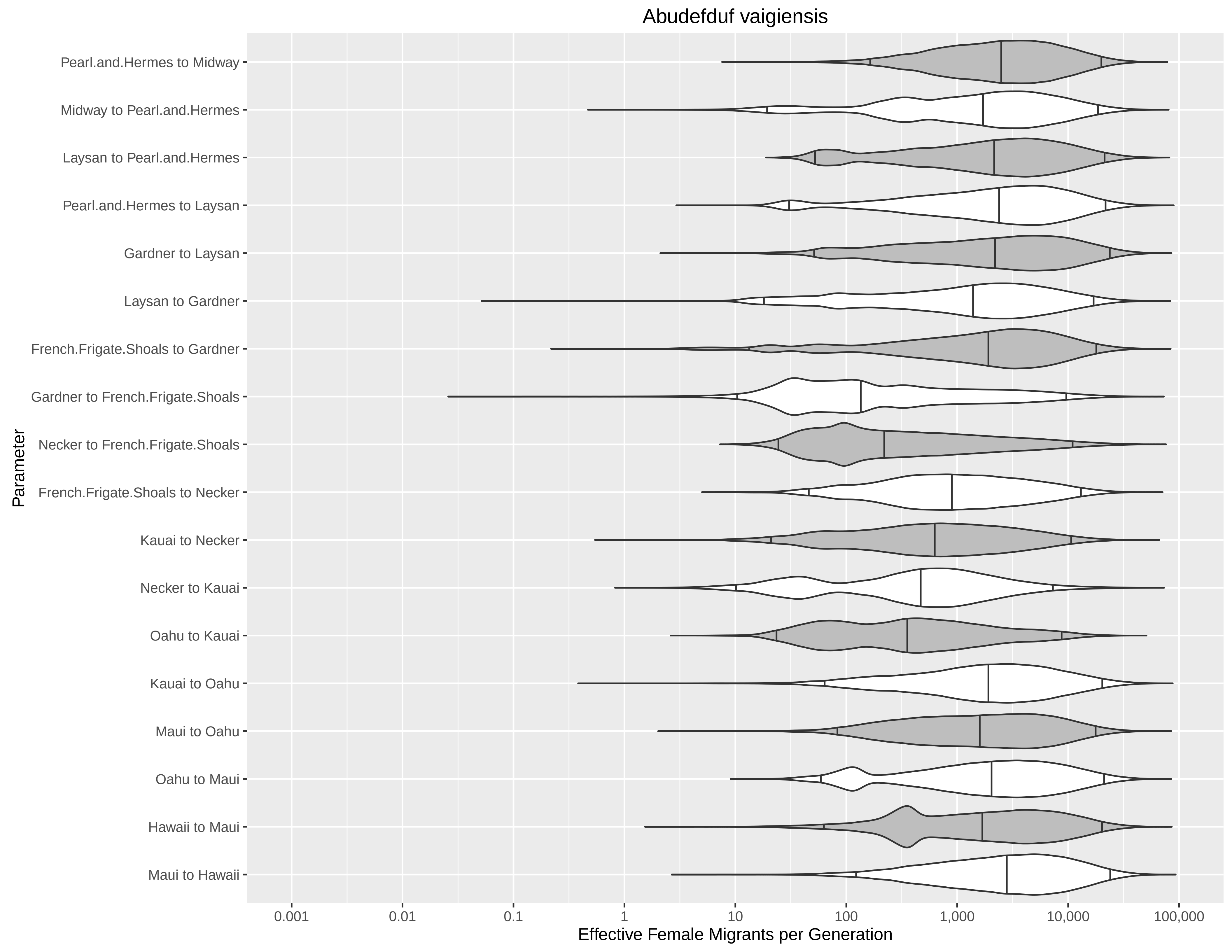

Abudefduf vaigiensis
Pearl.and.Hermes to Midway
Midway to Pearl.and.Hermes
Laysan to Pearl.and.Hermes
Pearl.and.Hermes to Laysan
Gardner to Laysan
Laysan to Gardner
French.Frigate.Shoals to Gardner
Gardner to French.Frigate.Shoals
Necker to French.Frigate.Shoals
French.Frigate.Shoals to Necker
Kauai to Necker
Necker to Kauai
Oahu to Kauai
Kauai to Oahu
Maui to Oahu
Oahu to Maui
Hawaii to Maui
Maui to Hawaii
Parameter
0.001
0.01
0.1
1
10
100
1,000
10,000
100,000
Effective Female Migrants per Generation

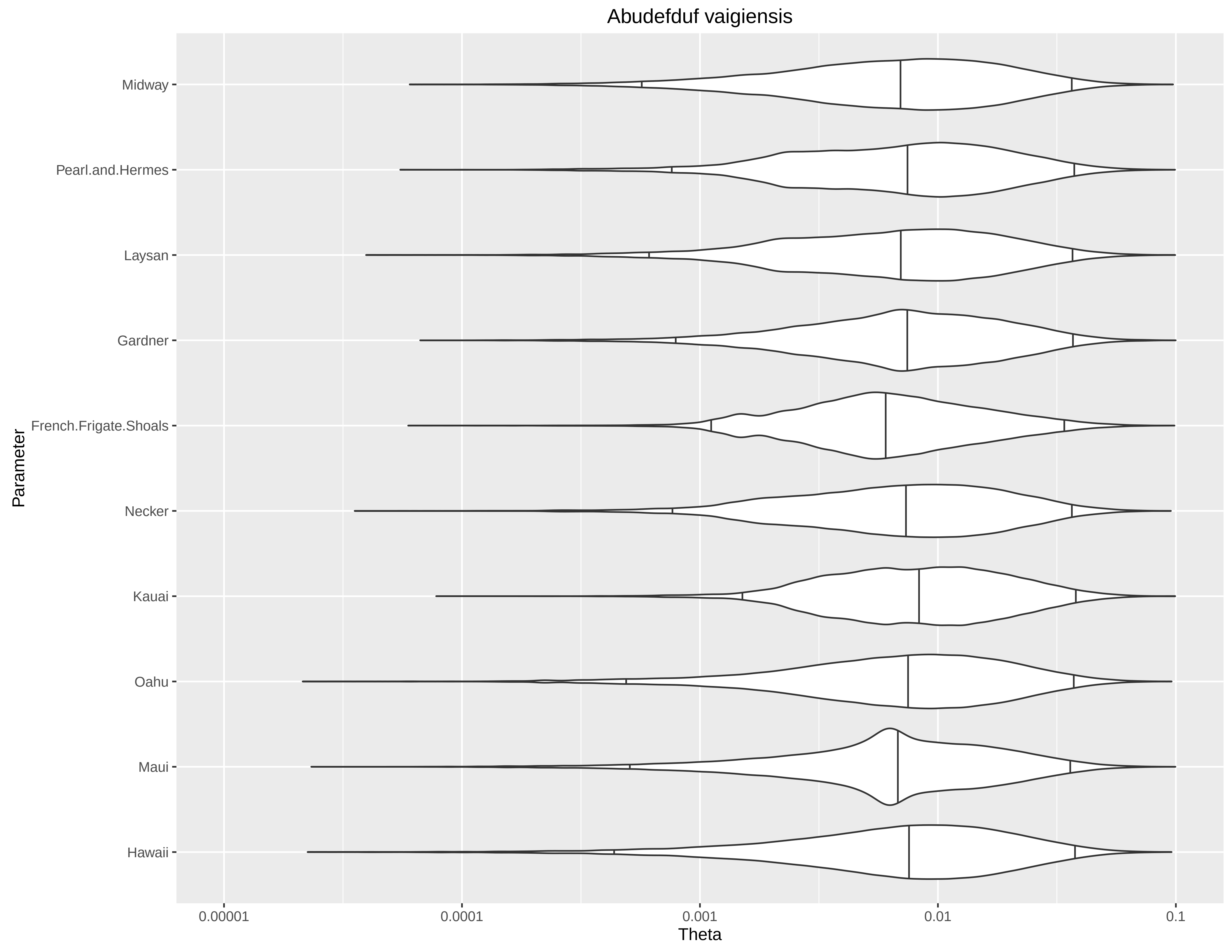

Abudefduf vaigiensis
Parameter
Midway
Pearl.and.Hermes
Laysan
Gardner
French.Frigate.Shoals
Necker
Kauai
Oahu
Maui
Hawaii
0.00001
0.0001
0.001
0.01
0.1
Theta

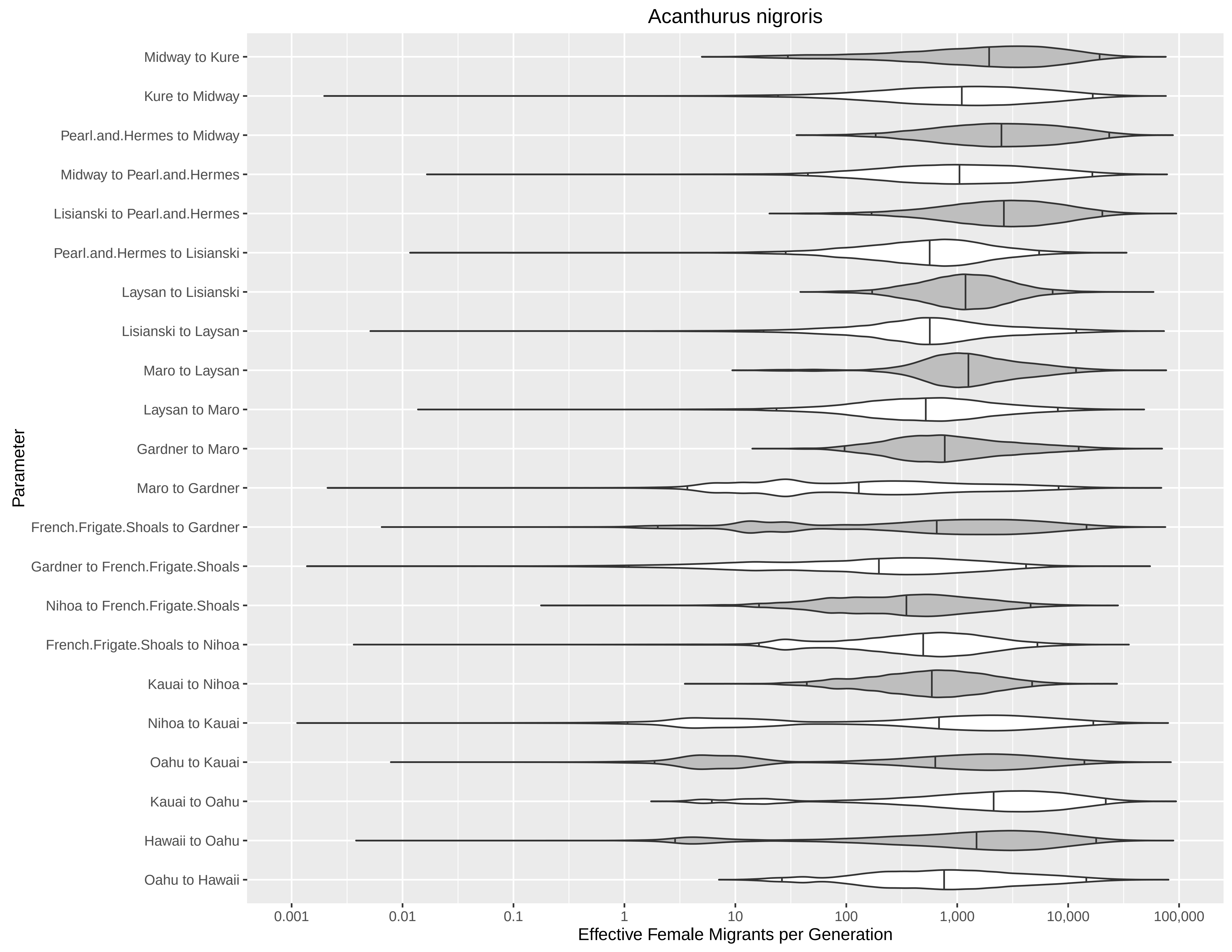

Acanthurus nigroris
Parameter
Midway to Kure
Kure to Midway
Pearl.and.Hermes to Midway
Midway to Pearl.and.Hermes
Lisianski to Pearl.and.Hermes
Pearl.and.Hermes to Lisianski
Laysan to Lisianski
Lisianski to Laysan
Maro to Laysan
Laysan to Maro
Gardner to Maro
Maro to Gardner
French.Frigate.Shoals to Gardner
Gardner to French.Frigate.Shoals
Nihoa to French.Frigate.Shoals
French.Frigate.Shoals to Nihoa
Kauai to Nihoa
Nihoa to Kauai
Oahu to Kauai
Kauai to Oahu
Hawaii to Oahu
Oahu to Hawaii
0.001
0.01
0.1
1
10
100
1,000
10,000
100,000
Effective Female Migrants per Generation

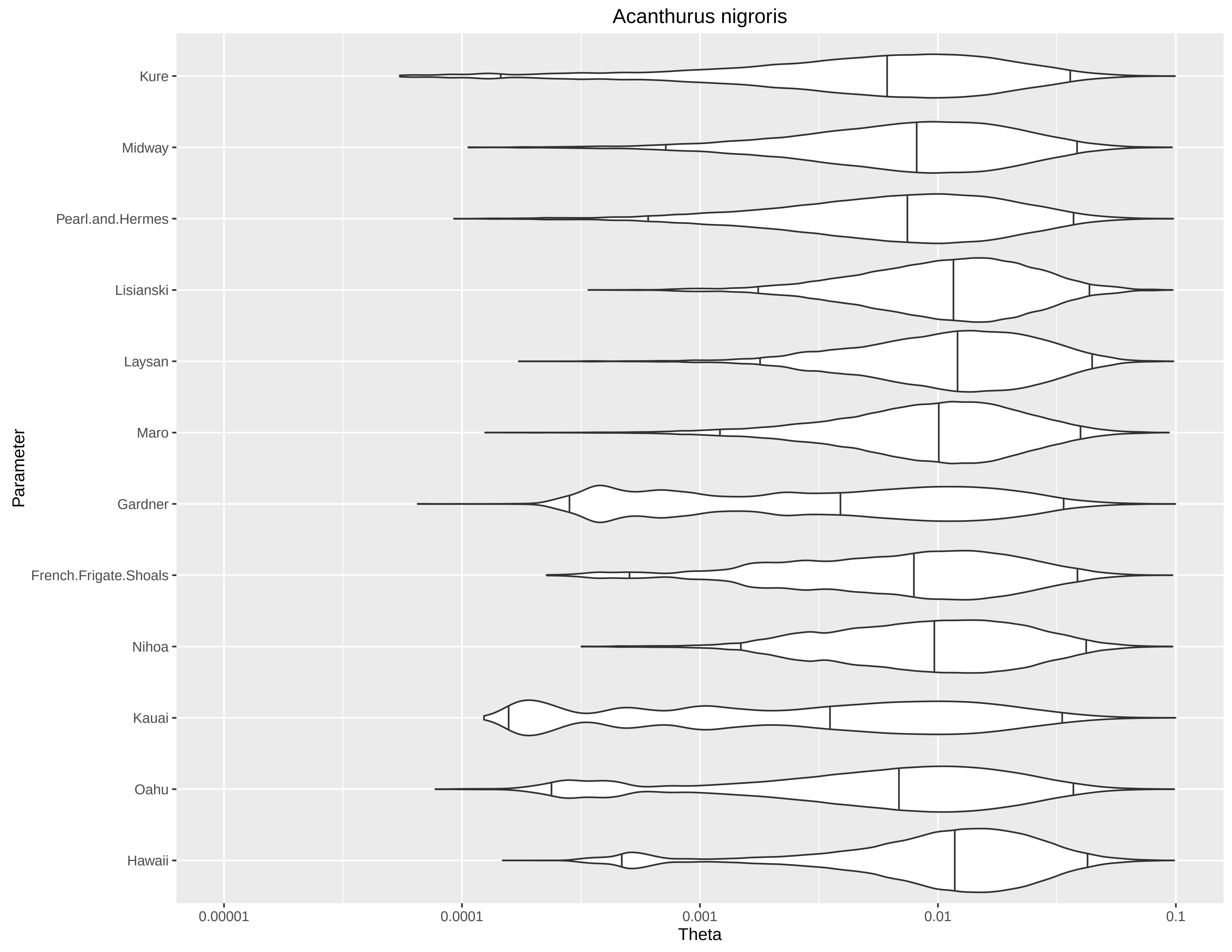

Acanthurus nigroris
Kure
Midway
Pearl.and.Hermes
Lisianski
Laysan
Maro
Gardner
French.Frigate.Shoals
Nihoa
Kauai
Oahu
Hawaii
Parameter
0.00001
0.0001
0.001
0.01
0.1
Theta

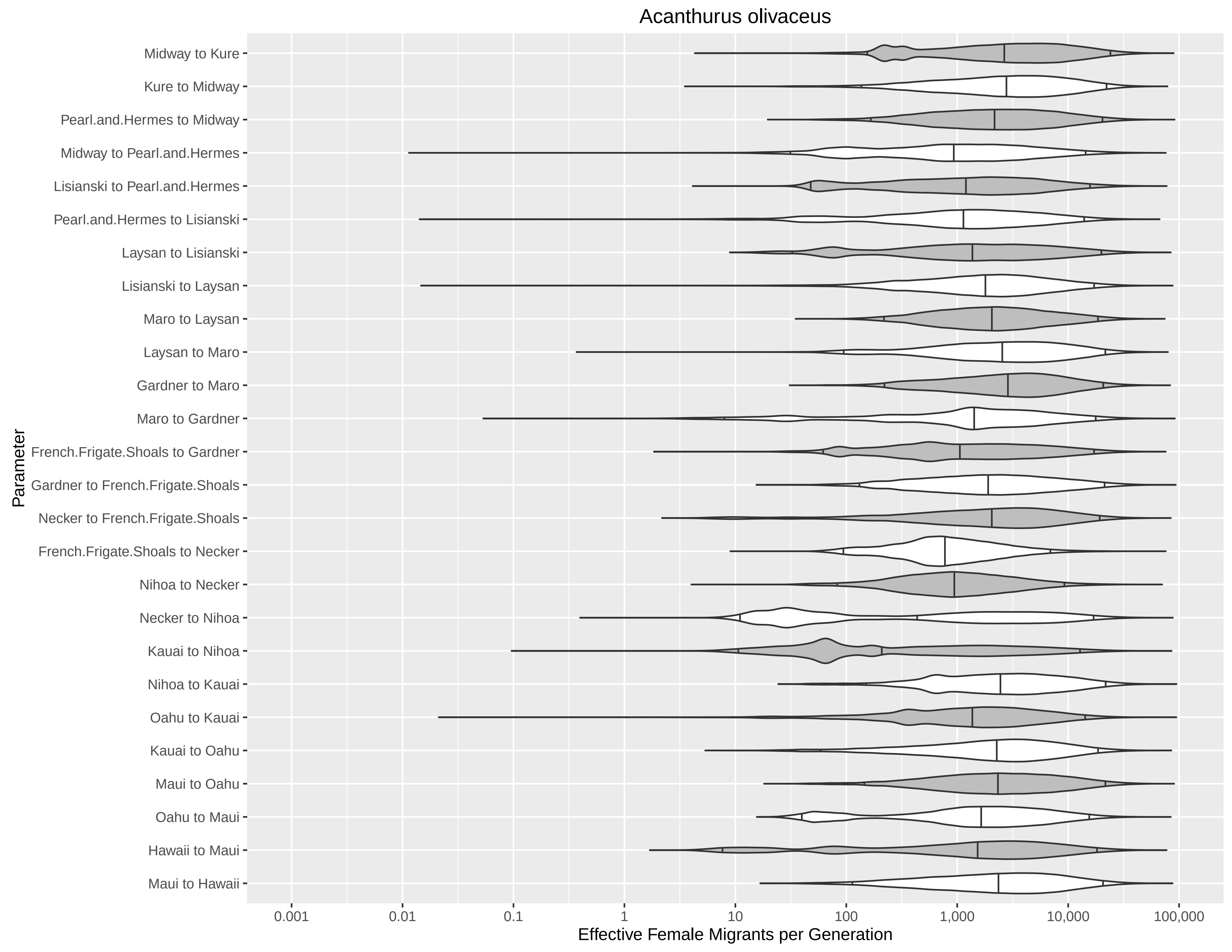

Acanthurus olivaceus
Midway to Kure
Kure to Midway
Pearl.and.Hermes to Midway
Midway to Pearl.and.Hermes
Lisianski to Pearl.and.Hermes
Pearl.and.Hermes to Lisianski
Laysan to Lisianski
Lisianski to Laysan
Maro to Laysan
Laysan to Maro
Gardner to Maro
Maro to Gardner
French.Frigate.Shoals to Gardner
Gardner to French.Frigate.Shoals
Necker to French.Frigate.Shoals
French.Frigate.Shoals to Necker
Nihoa to Necker
Necker to Nihoa
Kauai to Nihoa
Nihoa to Kauai
Oahu to Kauai
Kauai to Oahu
Maui to Oahu
Oahu to Maui
Hawaii to Maui
Maui to Hawaii
Parameter
0.001
0.01
0.1
1
10
100
1,000
10,000
100,000
Effective Female Migrants per Generation

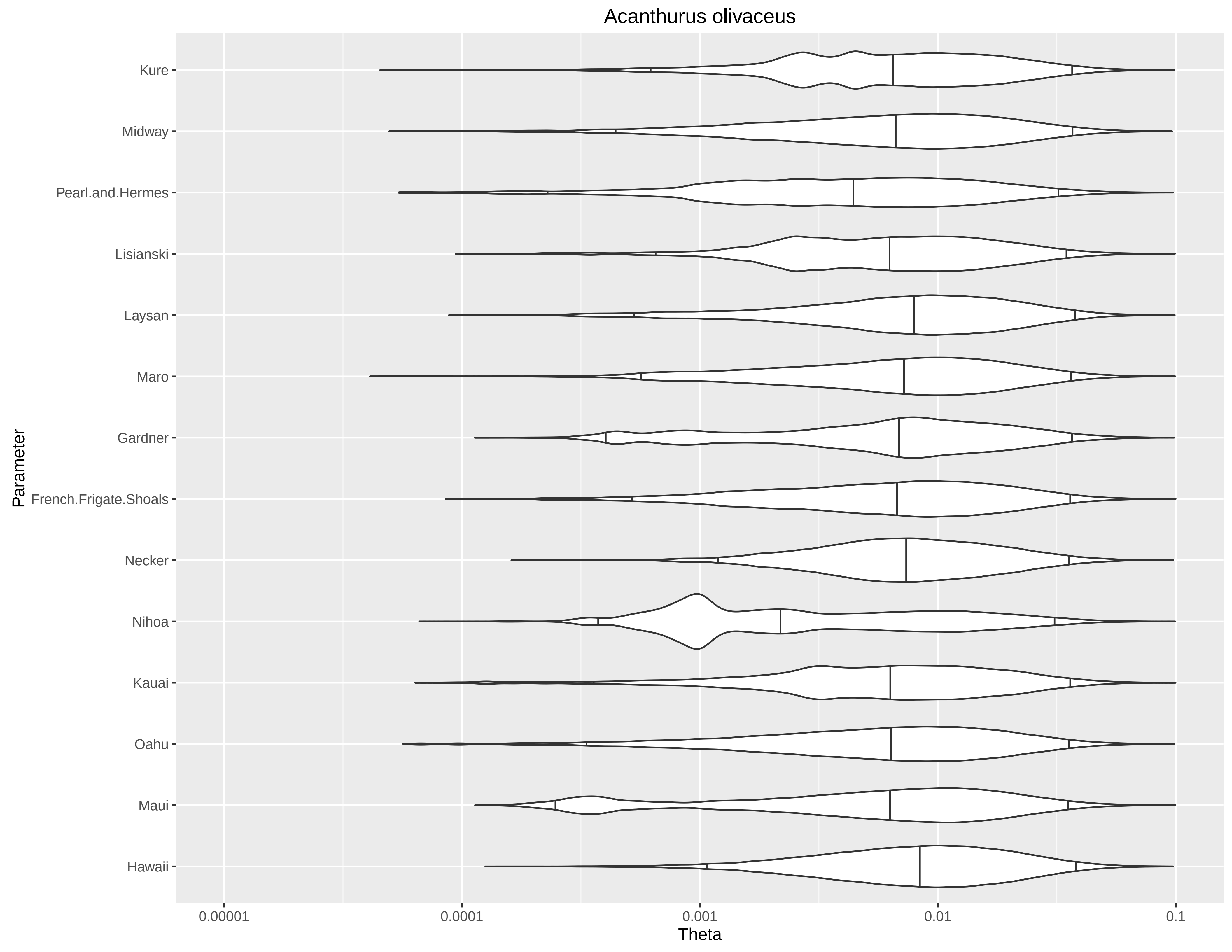

Acanthurus olivaceus
Parameter
Kure
Midway
Pearl.and.Hermes
Lisianski
Laysan
Maro
Gardner
French.Frigate.Shoals
Necker
Nihoa
Kauai
Oahu
Maui
Hawaii
0.00001
0.0001
0.001
0.01
0.1
Theta

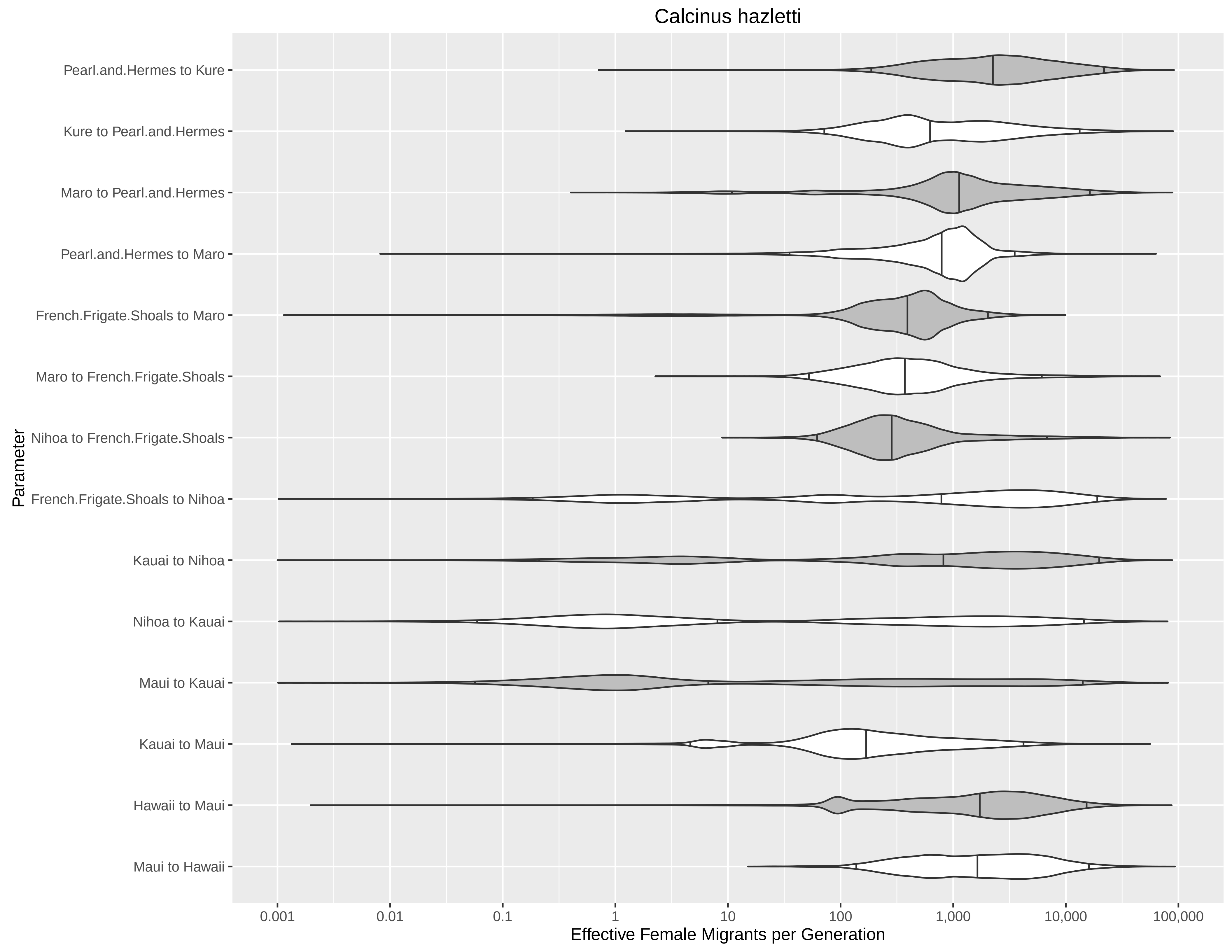
Calcinus hazletti
Parameter
Pearl.and.Hermes to Kure
Kure to Pearl.and.Hermes
Maro to Pearl.and.Hermes
Pearl.and.Hermes to Maro
French.Frigate.Shoals to Maro
Maro to French.Frigate.Shoals
Nihoa to French.Frigate.Shoals
French.Frigate.Shoals to Nihoa
Kauai to Nihoa
Nihoa to Kauai
Maui to Kauai
Kauai to Maui
Hawaii to Maui
Maui to Hawaii
0.001
0.01
0.1
1
10
100
1,000
10,000
100,000
Effective Female Migrants per Generation

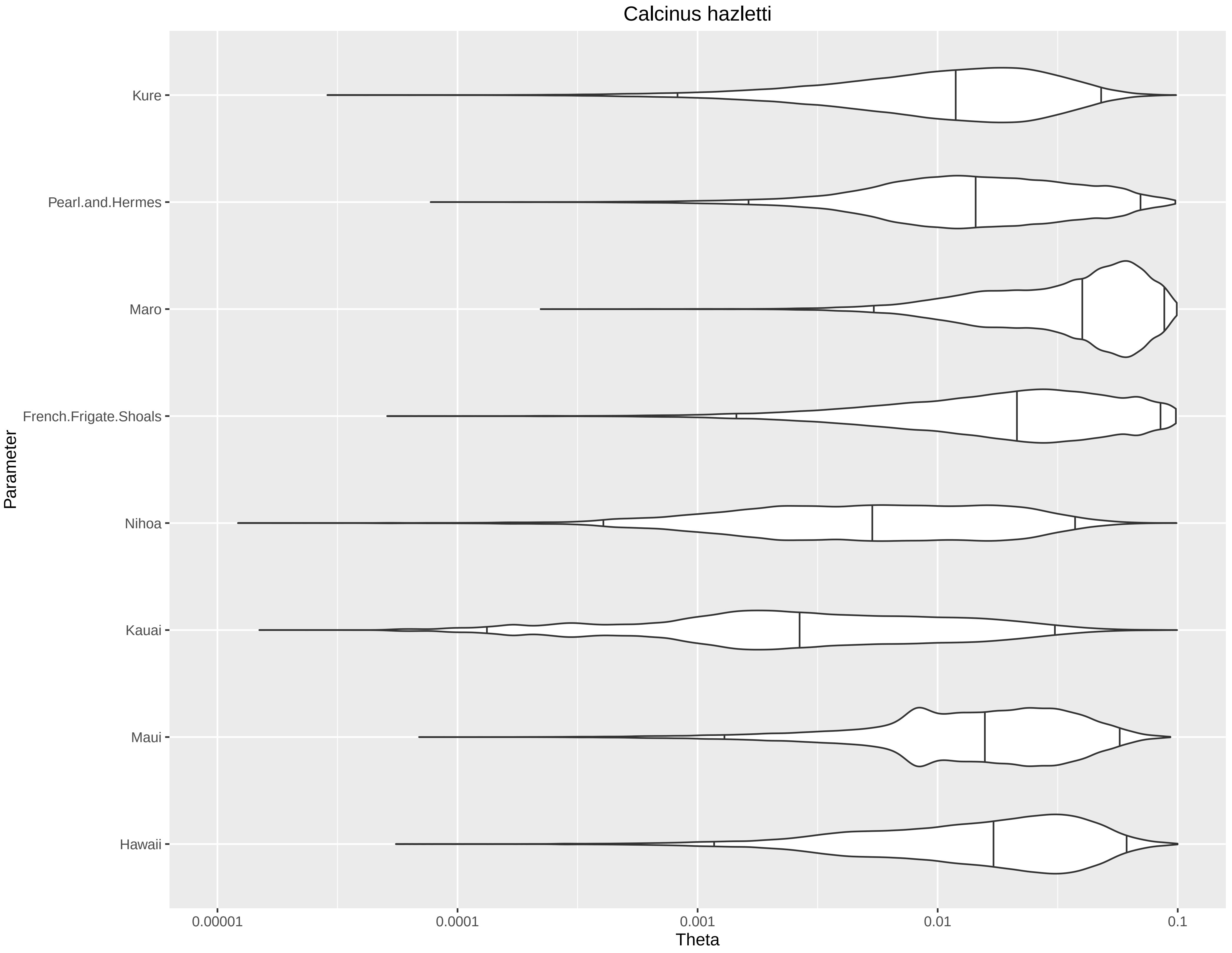
Calcinus hazletti
Kure
Pearl.and.Hermes
Maro
French.Frigate.Shoals
Nihoa
Kauai
Maui
Hawaii
Parameter
0.00001
0.0001
0.001
0.01
0.1
Theta

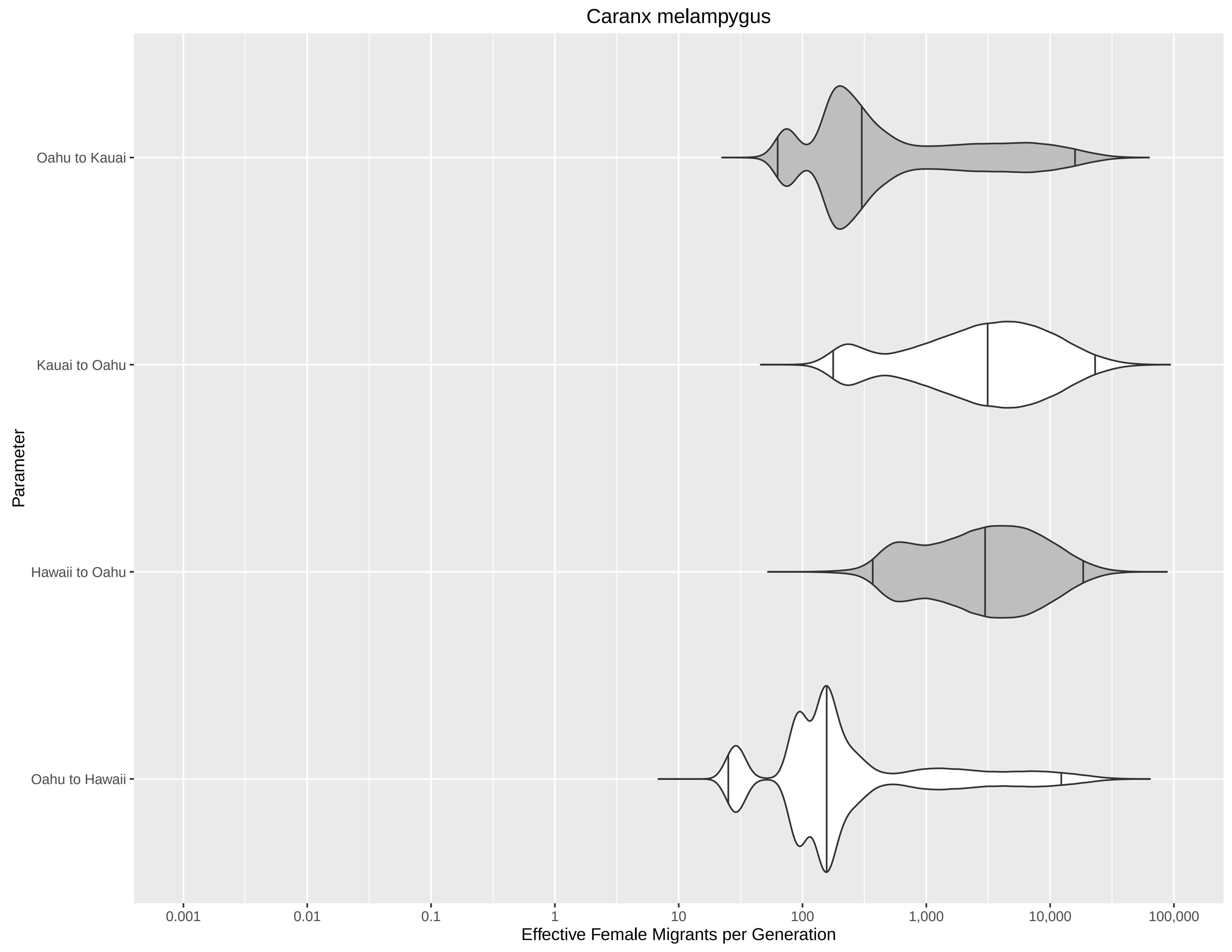

Caranx melampygus
Oahu to Kauai
Kauai to Oahu
Hawaii to Oahu
Oahu to Hawaii
Parameter
0.001
0.01
0.1
1
10
100
1,000
10,000
100,000
Effective Female Migrants per Generation

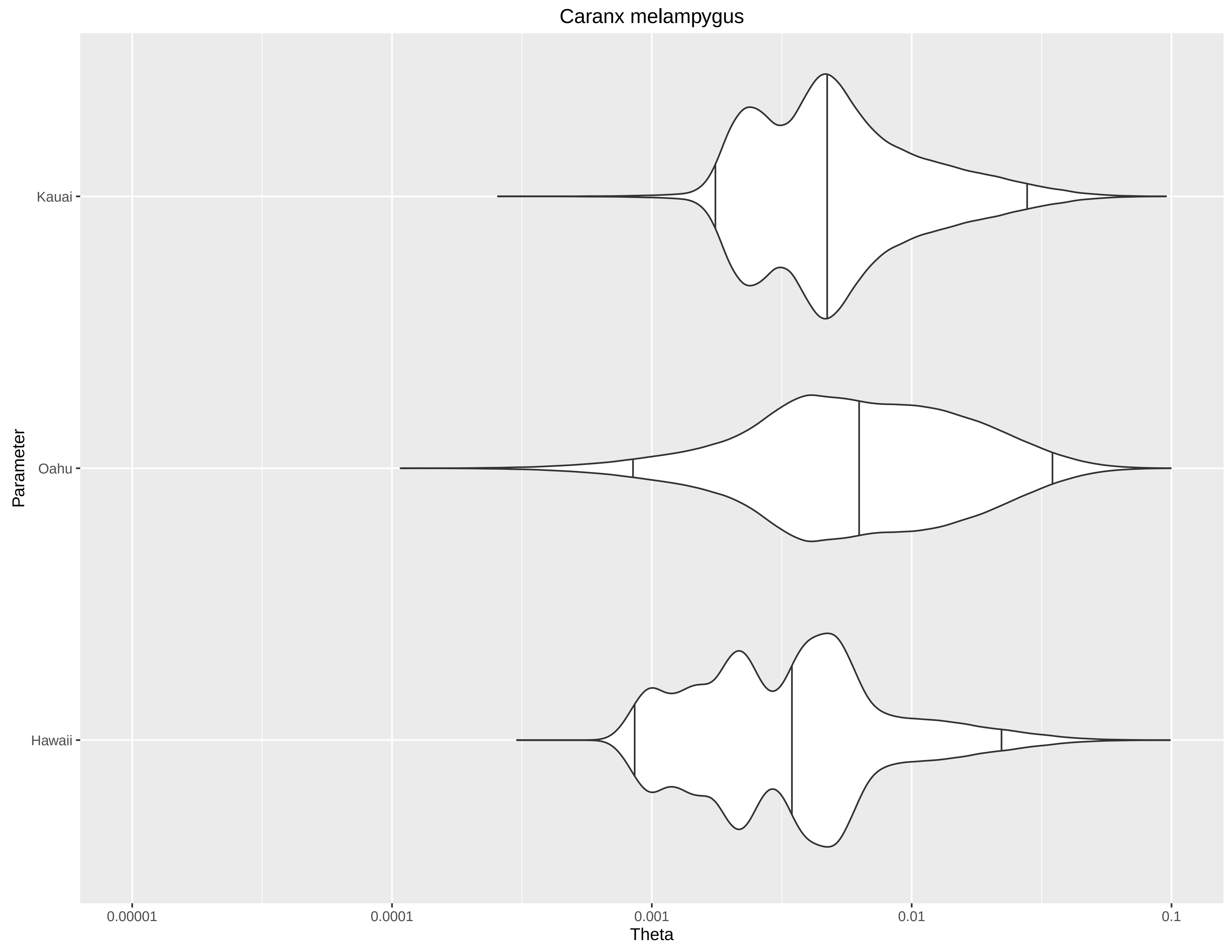

Caranx melampygus
Kauai
Oahu
Hawaii
Parameter
0.00001
0.0001
0.001
0.01
0.1
Theta

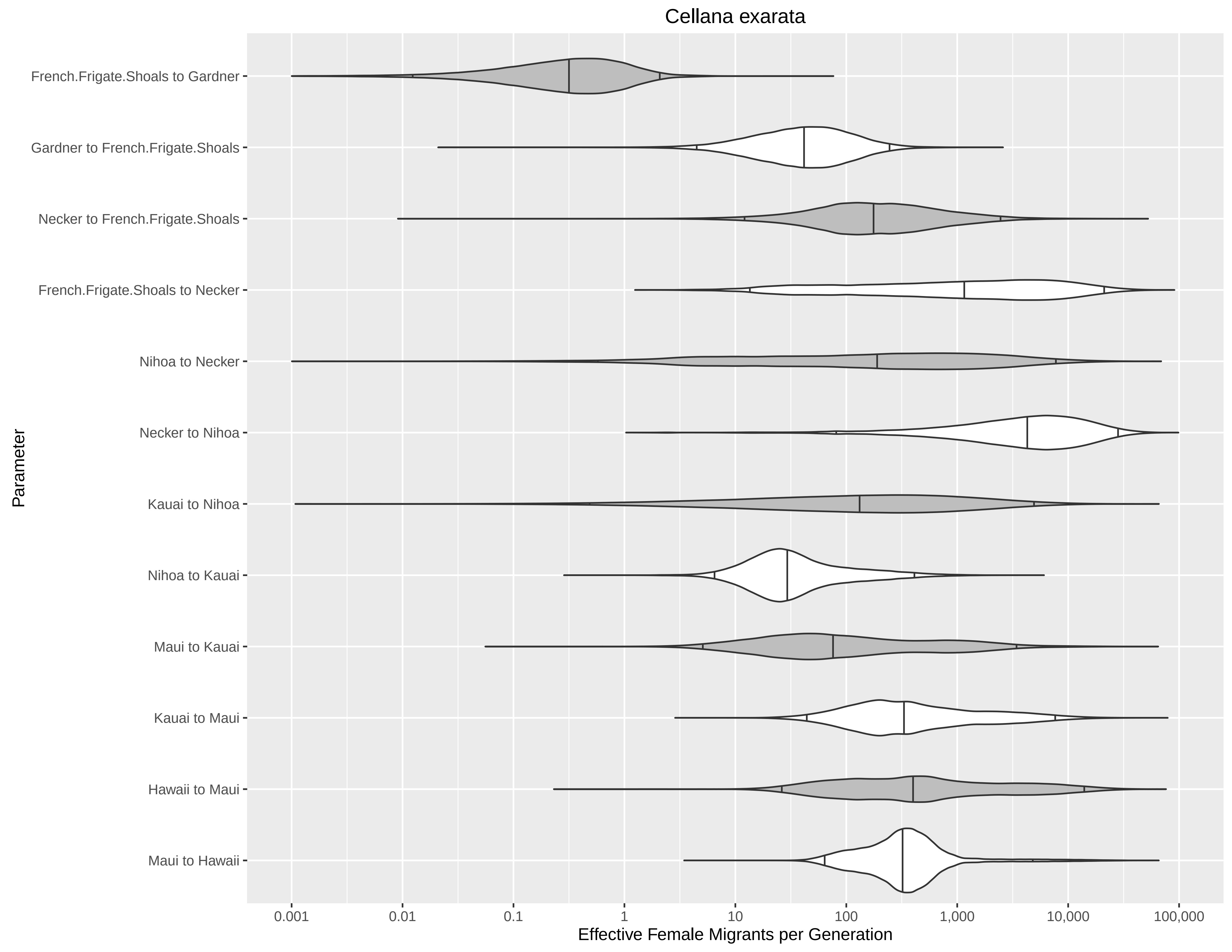
Cellana exarata
French.Frigate.Shoals to Gardner
Gardner to French.Frigate.Shoals
Necker to French.Frigate.Shoals
French.Frigate.Shoals to Necker
Nihoa to Necker
Necker to Nihoa
Kauai to Nihoa
Nihoa to Kauai
Maui to Kauai
Kauai to Maui
Hawaii to Maui
Maui to Hawaii
Parameter
0.001
0.01
0.1
1
10
100
1,000
10,000
100,000
Effective Female Migrants per Generation

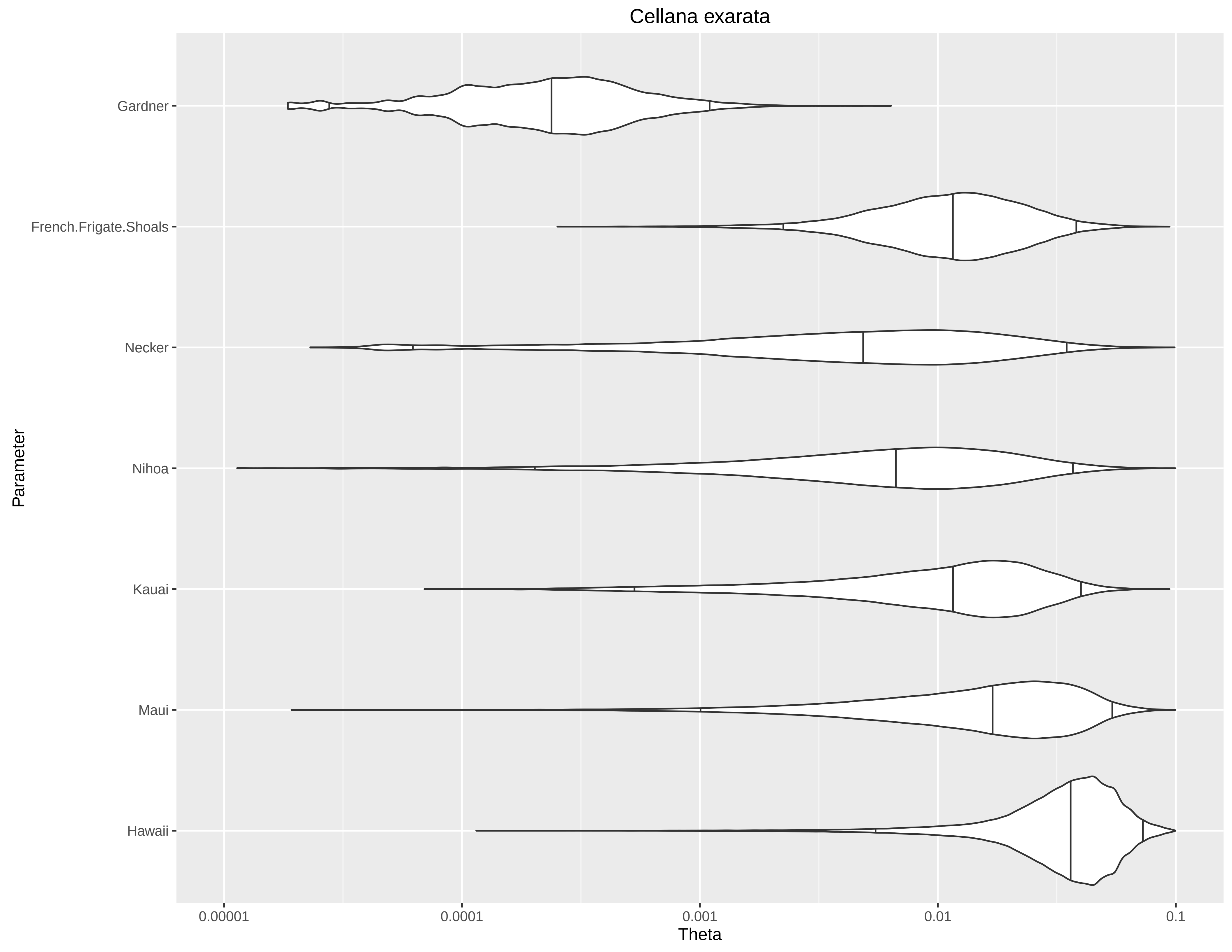

Cellana exarata
Parameter
Gardner
French.Frigate.Shoals
Necker
Nihoa
Kauai
Maui
Hawaii
0.00001
0.0001
0.001
0.01
0.1
Theta

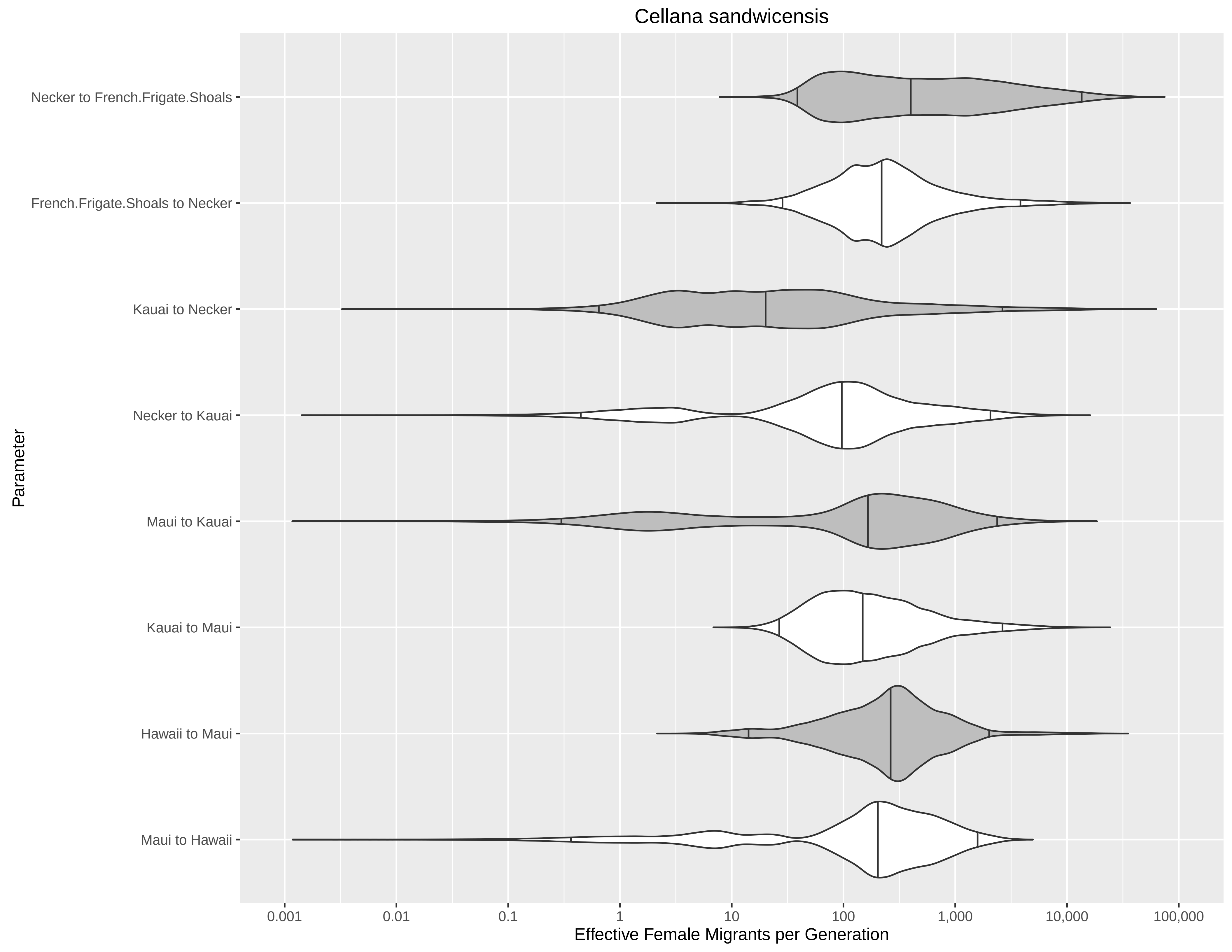

Cellana sandwicensis
Necker to French.Frigate.Shoals
French.Frigate.Shoals to Necker
Kauai to Necker
Necker to Kauai
Maui to Kauai
Kauai to Maui
Hawaii to Maui
Maui to Hawaii
Parameter
0.001
0.01
0.1
1
10
100
1,000
10,000
100,000
Effective Female Migrants per Generation

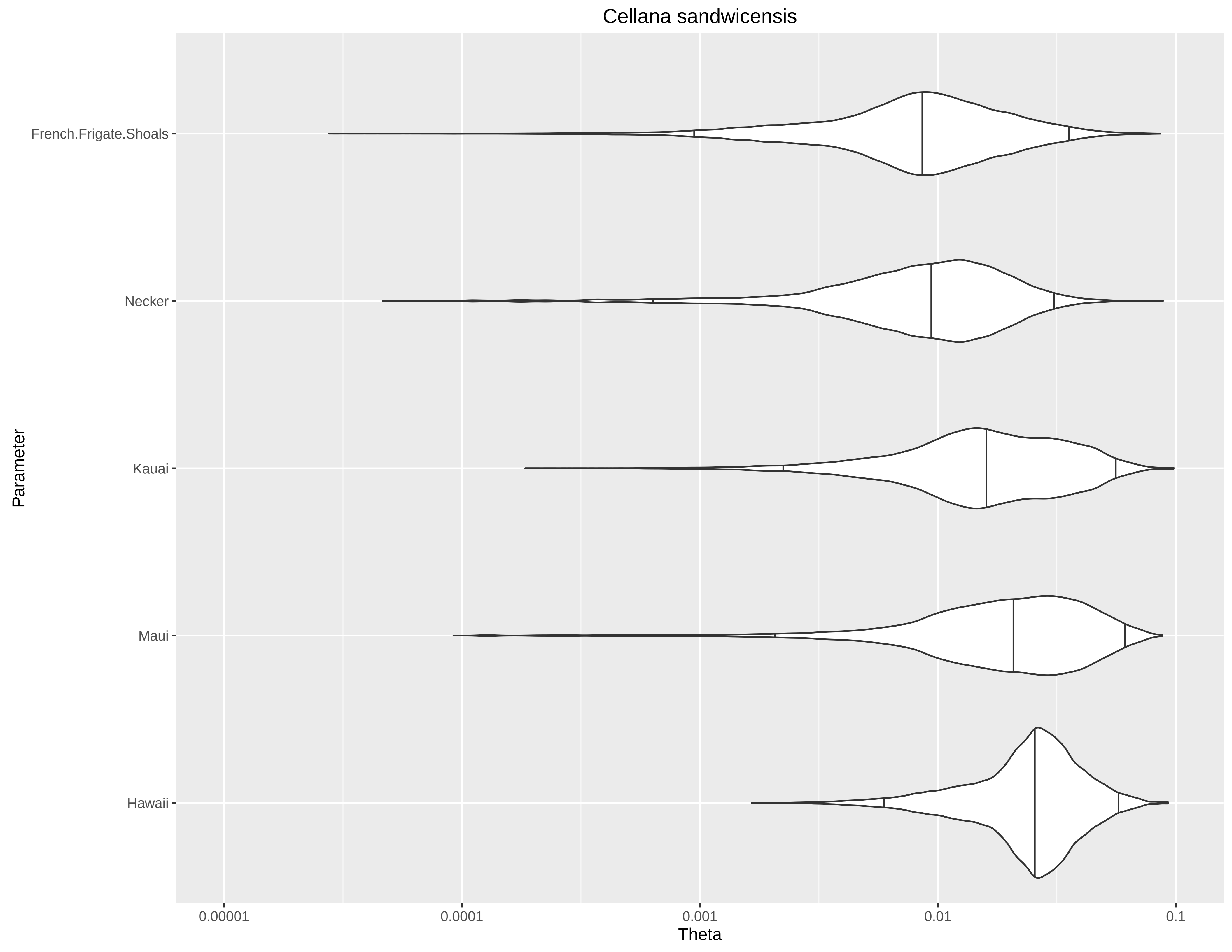
Cellana sandwicensis
French.Frigate.Shoals
Necker
Kauai
Maui
Hawaii
Parameter
0.00001
0.0001
0.001
0.01
0.1
Theta

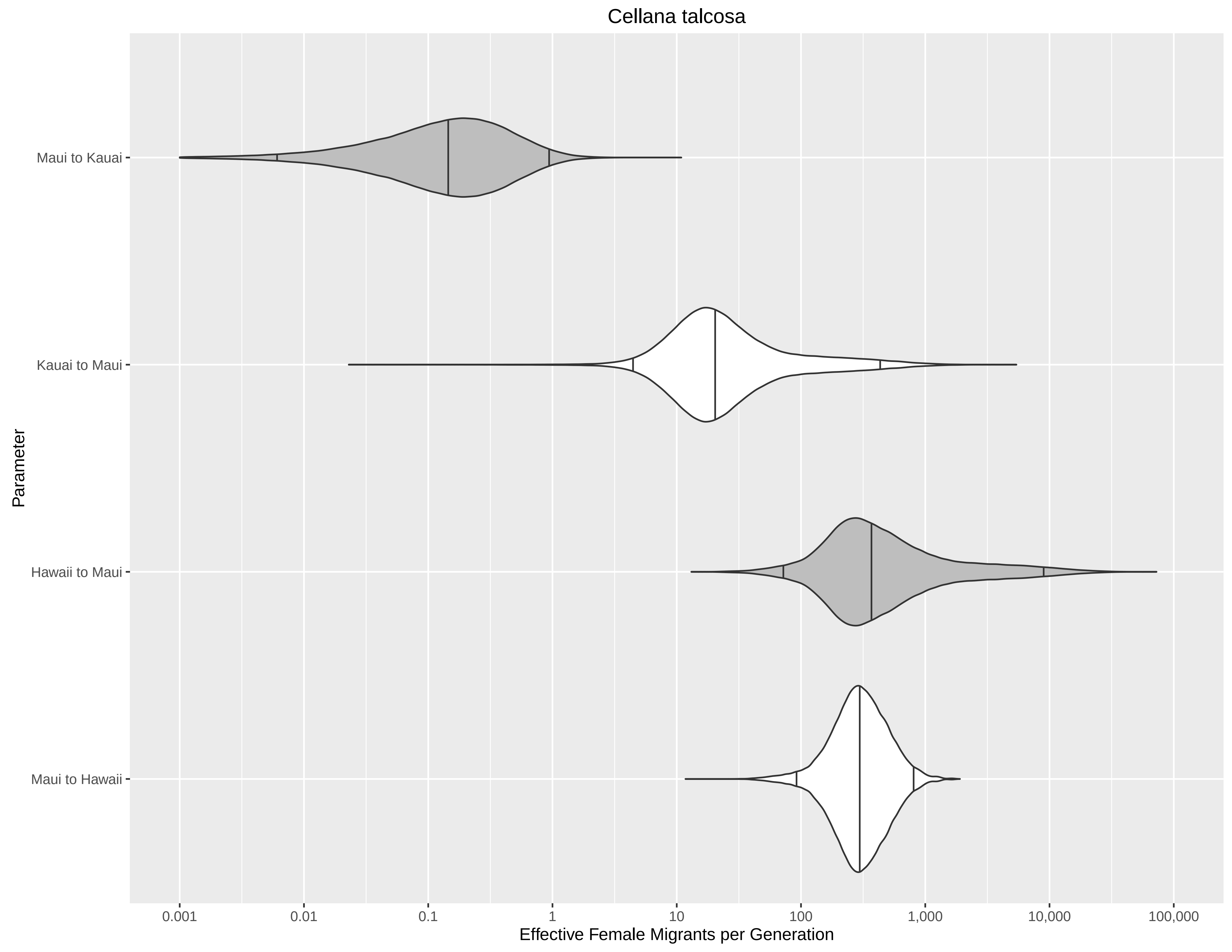
Cellana talcosa
Parameter
Maui to Kauai
Kauai to Maui
Hawaii to Maui
Maui to Hawaii
0.001
0.01
0.1
1
10
100
1,000
10,000
100,000
Effective Female Migrants per Generation

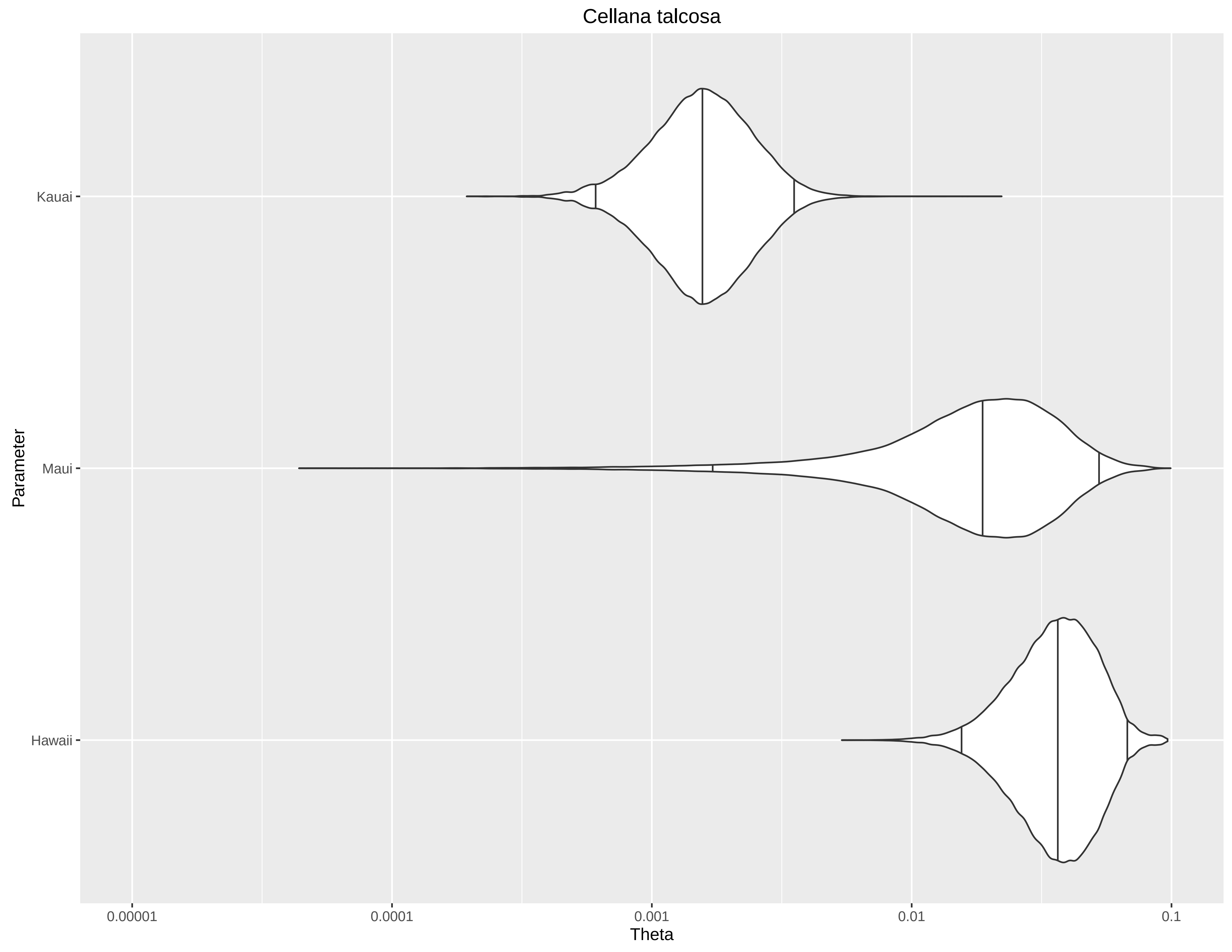

Cellana talcosa
Kauai
Maui
Hawaii
Parameter
0.00001
0.0001
0.001
0.01
0.1
Theta

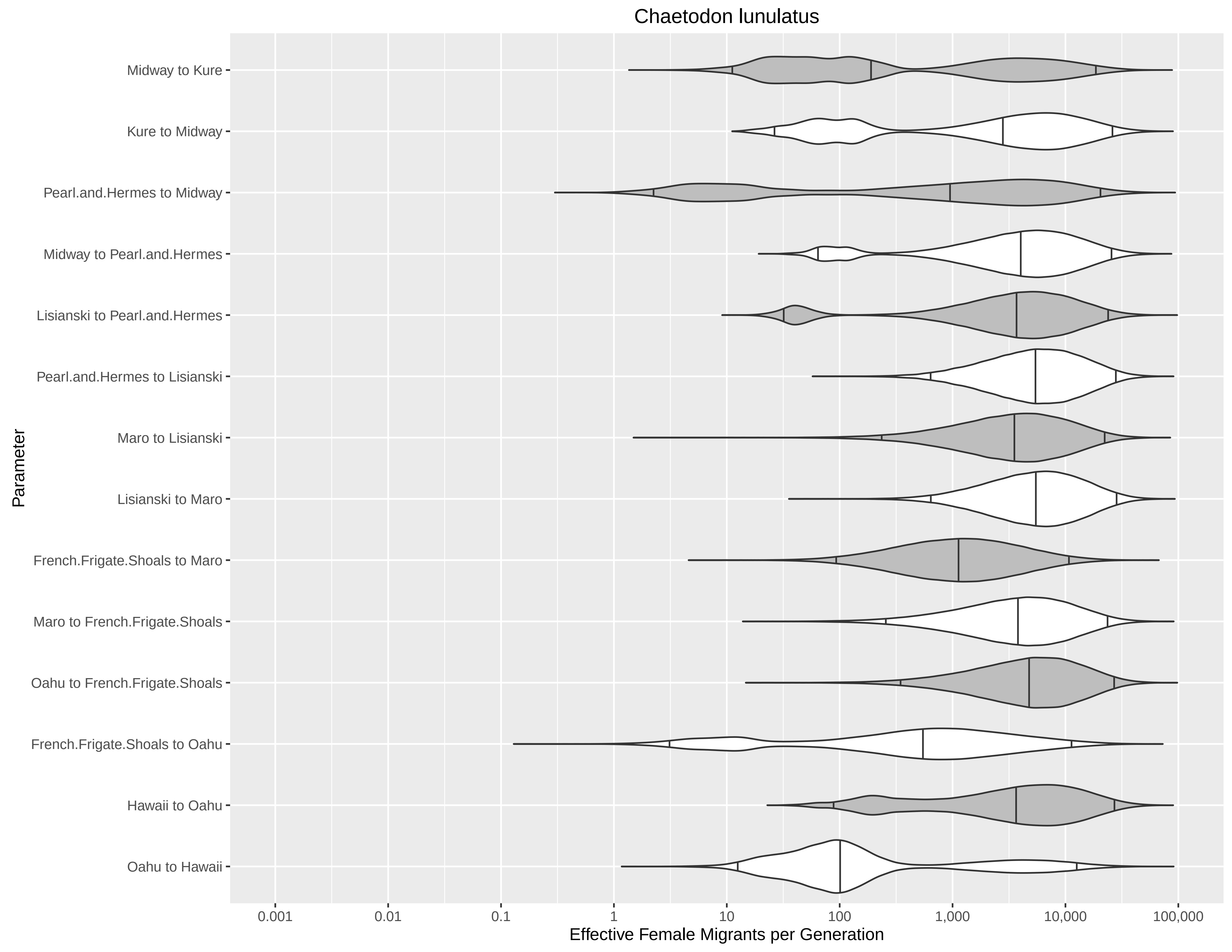
Chaetodon lunulatus
Parameter
Midway to Kure
Kure to Midway
Pearl.and.Hermes to Midway
Midway to Pearl.and.Hermes
Lisianski to Pearl.and.Hermes
Pearl.and.Hermes to Lisianski
Maro to Lisianski
Lisianski to Maro
French.Frigate.Shoals to Maro
Maro to French.Frigate.Shoals
Oahu to French.Frigate.Shoals
French.Frigate.Shoals to Oahu
Hawaii to Oahu
Oahu to Hawaii
0.001
0.01
0.1
1
10
100
1,000
10,000
100,000
Effective Female Migrants per Generation

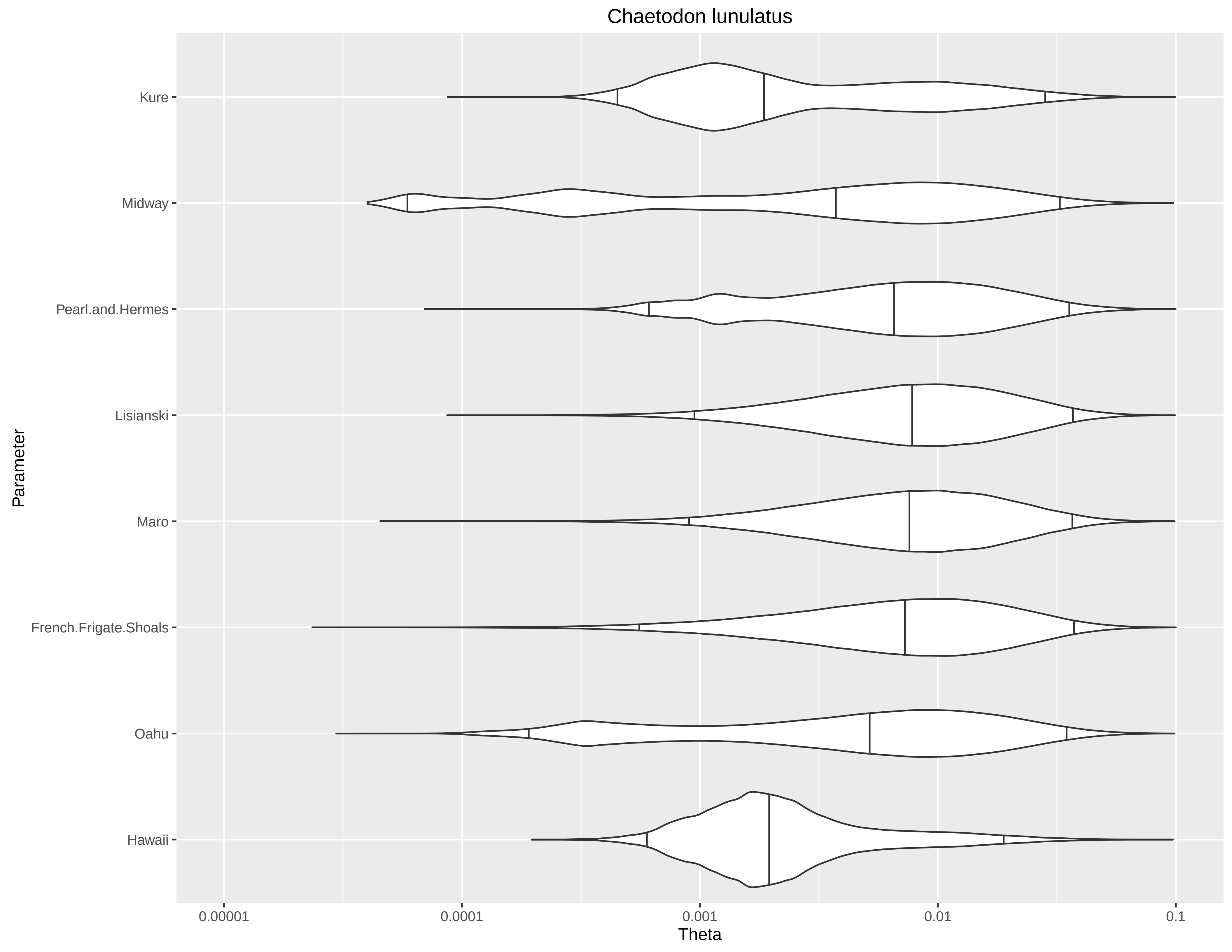
Chaetodon lunulatus
Kure
Midway
Pearl.and.Hermes
Lisianski
Maro
French.Frigate.Shoals
Oahu
Hawaii
Parameter
0.00001
0.0001
0.001
0.01
0.1
Theta

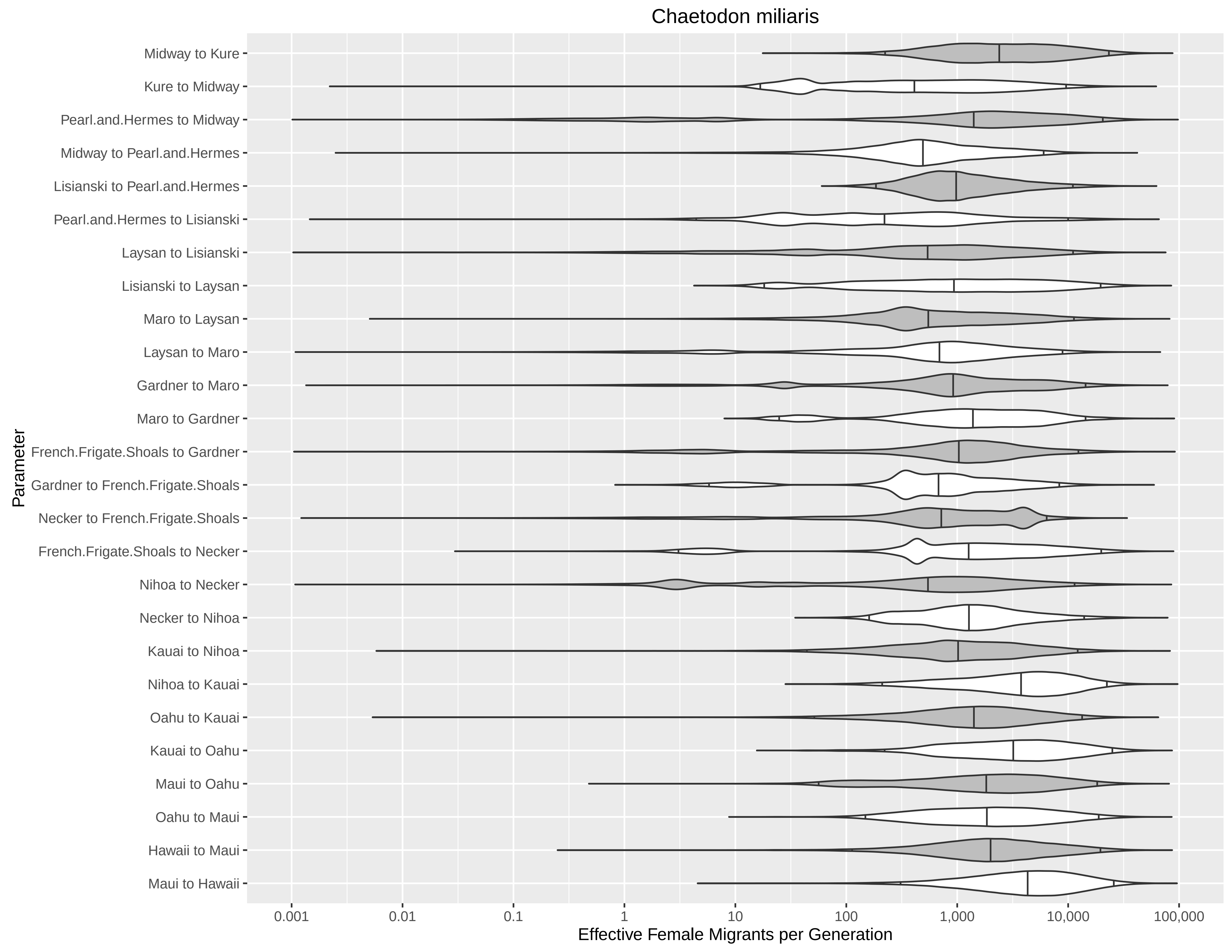

Chaetodon miliaris
Parameter
Midway to Kure
Kure to Midway
Pearl.and.Hermes to Midway
Midway to Pearl.and.Hermes
Lisianski to Pearl.and.Hermes
Pearl.and.Hermes to Lisianski
Laysan to Lisianski
Lisianski to Laysan
Maro to Laysan
Laysan to Maro
Gardner to Maro
Maro to Gardner
French.Frigate.Shoals to Gardner
Gardner to French.Frigate.Shoals
Necker to French.Frigate.Shoals
French.Frigate.Shoals to Necker
Nihoa to Necker
Necker to Nihoa
Kauai to Nihoa
Nihoa to Kauai
Oahu to Kauai
Kauai to Oahu
Maui to Oahu
Oahu to Maui
Hawaii to Maui
Maui to Hawaii
0.001
0.01
0.1
1
10
100
1,000
10,000
100,000
Effective Female Migrants per Generation

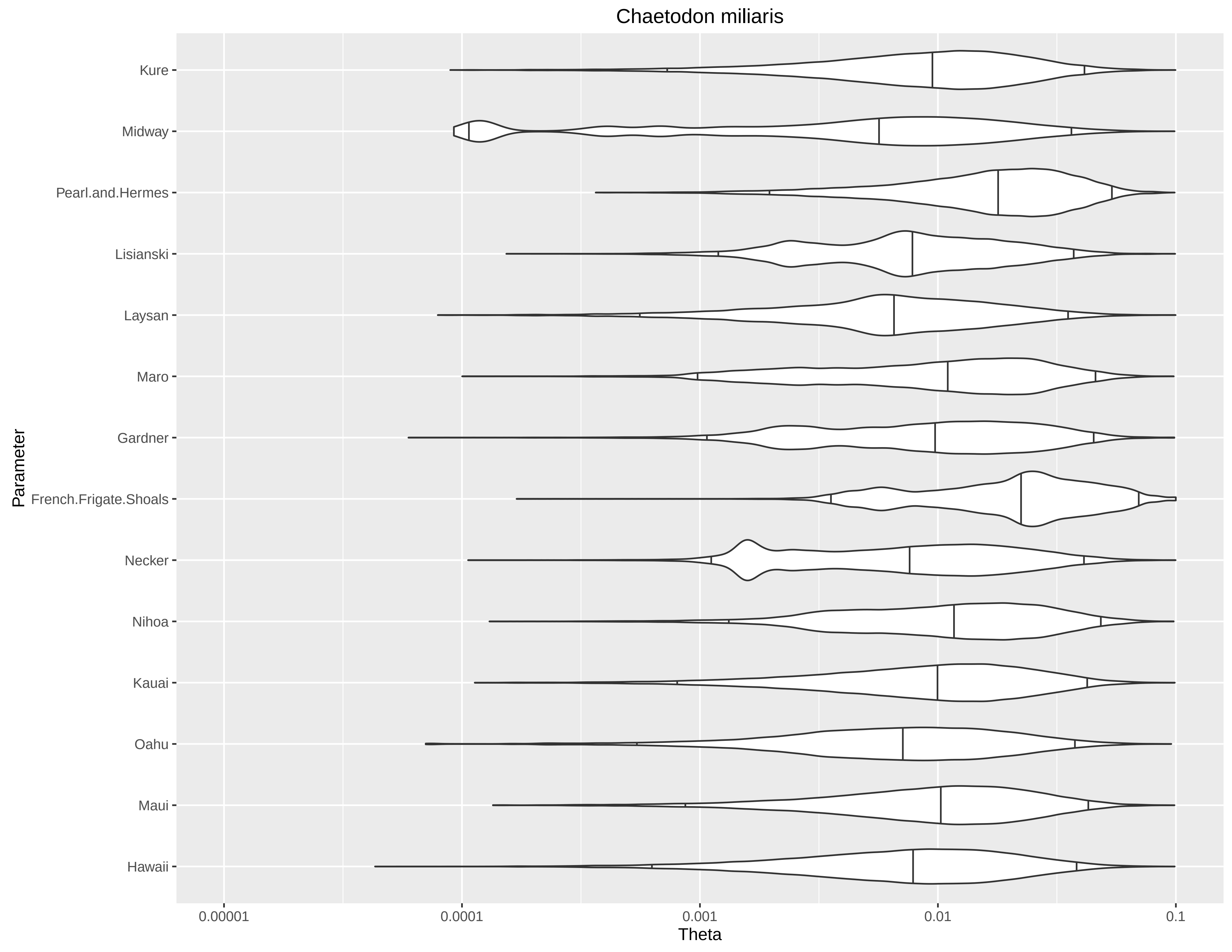

Chaetodon miliaris
Kure
Midway
Pearl.and.Hermes
Lisianski
Laysan
Maro
Gardner
French.Frigate.Shoals
Necker
Nihoa
Kauai
Oahu
Maui
Hawaii
Parameter
0.00001
0.0001
0.001
0.01
0.1
Theta

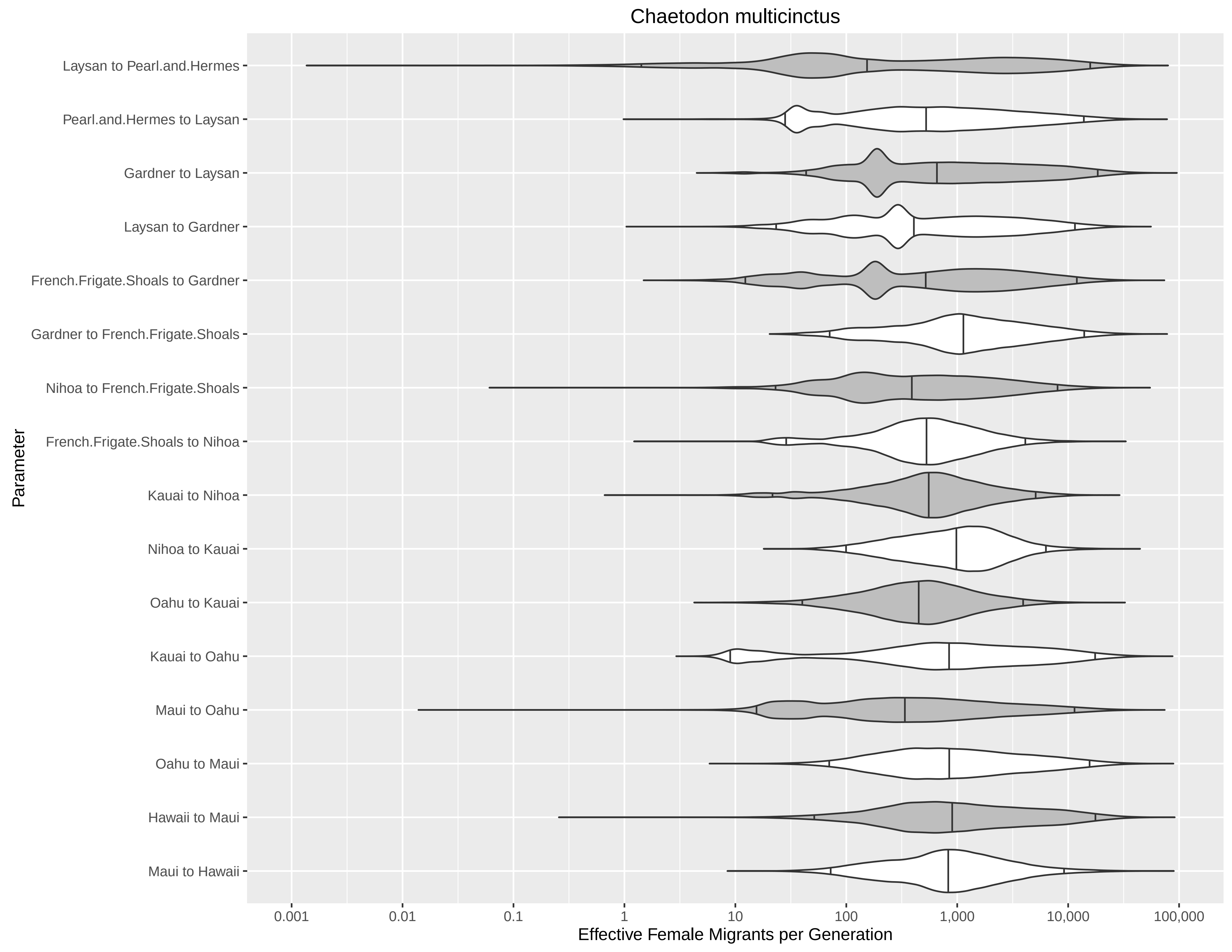
Chaetodon multicinctus
Laysan to Pearl.and.Hermes
Pearl.and.Hermes to Laysan
Gardner to Laysan
Laysan to Gardner
French.Frigate.Shoals to Gardner
Gardner to French.Frigate.Shoals
Nihoa to French.Frigate.Shoals
French.Frigate.Shoals to Nihoa
Kauai to Nihoa
Nihoa to Kauai
Oahu to Kauai
Kauai to Oahu
Maui to Oahu
Oahu to Maui
Hawaii to Maui
Maui to Hawaii
Parameter
0.001
0.01
0.1
1
10
100
1,000
10,000
100,000
Effective Female Migrants per Generation

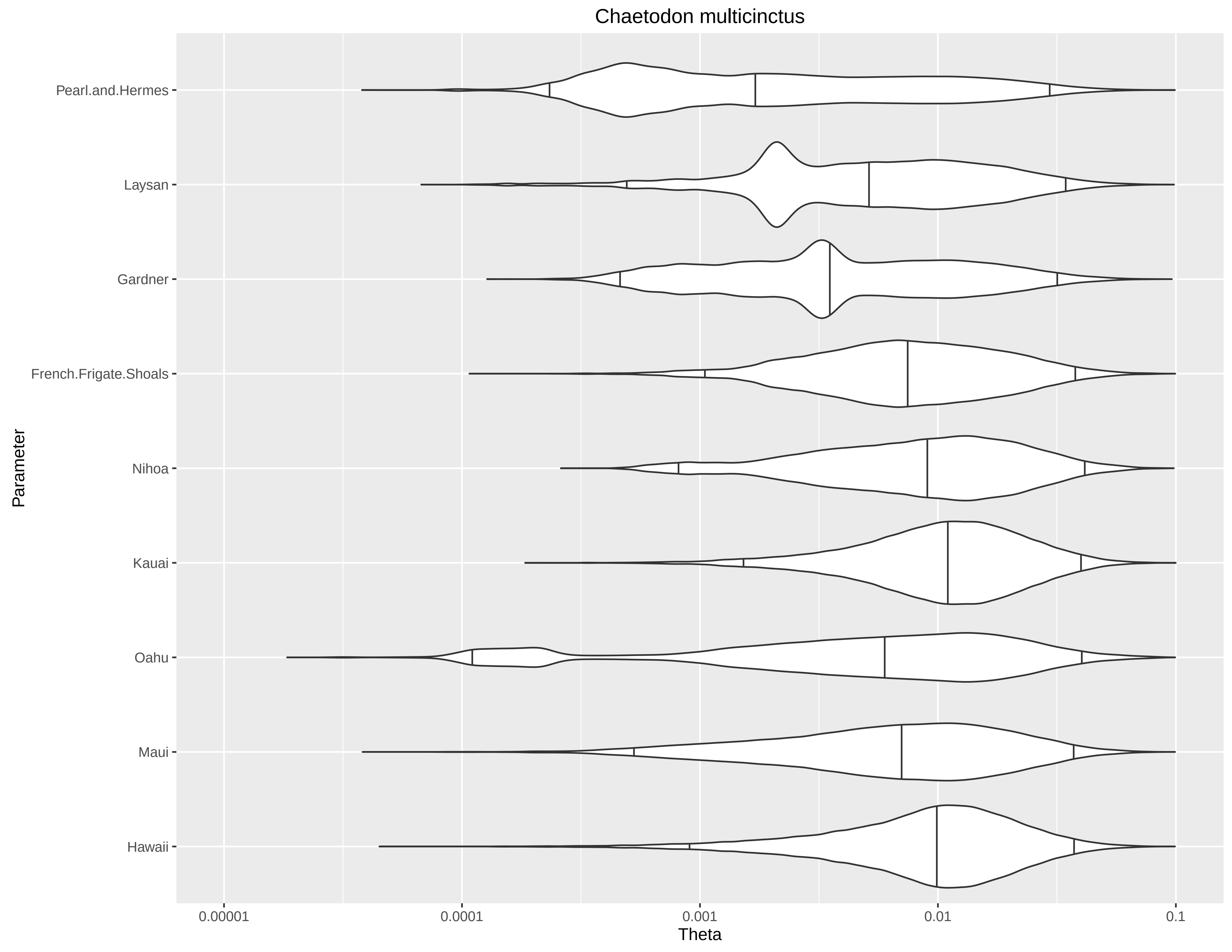

Chaetodon multicinctus
Pearl.and.Hermes
Laysan
Gardner
French.Frigate.Shoals
Nihoa
Kauai
Oahu
Maui
Hawaii
Parameter
0.00001
0.0001
0.001
0.01
0.1
Theta

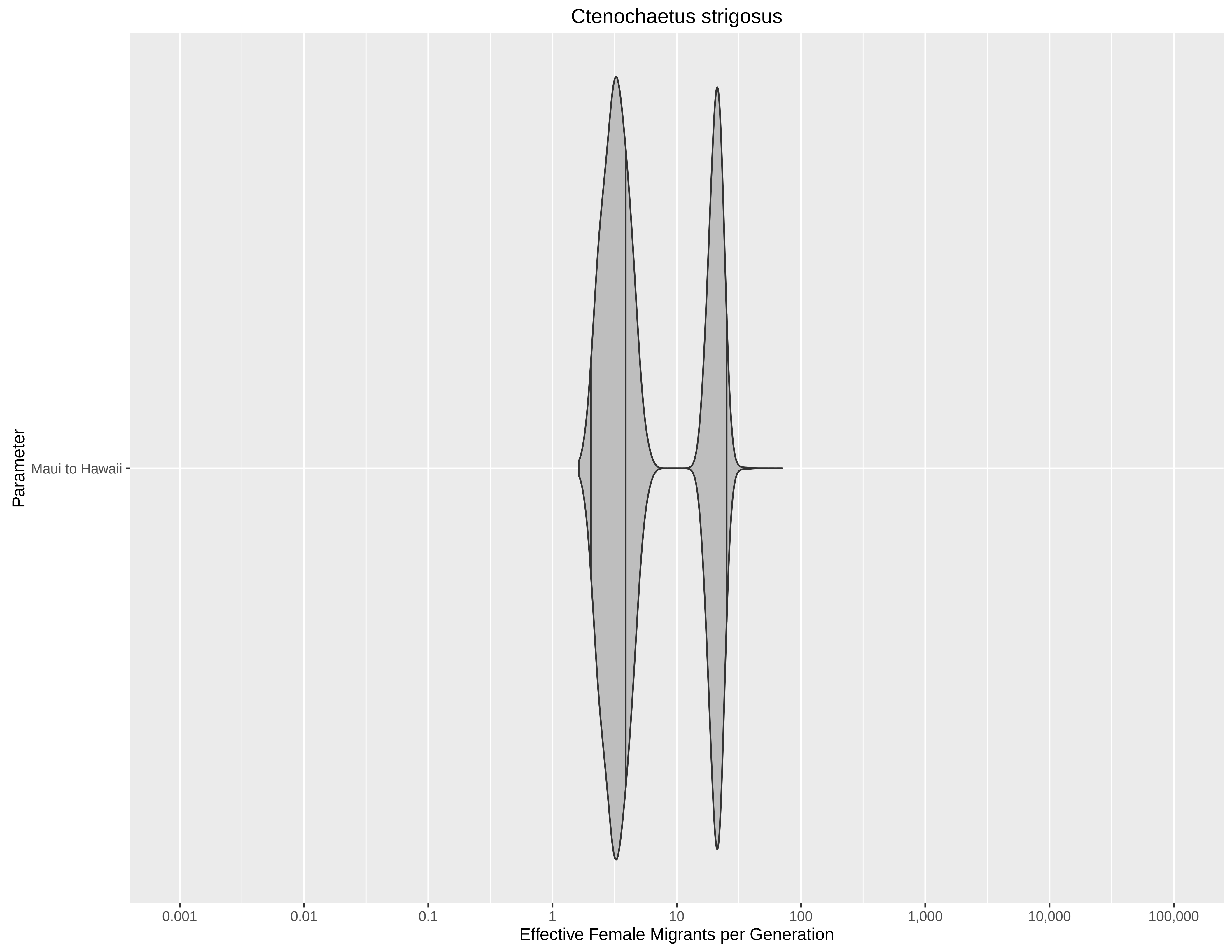

Ctenochaetus strigosus
Parameter
Maui to Hawaii
0.001
0.01
0.1
1
10
100
1,000
10,000
100,000
Effective Female Migrants per Generation

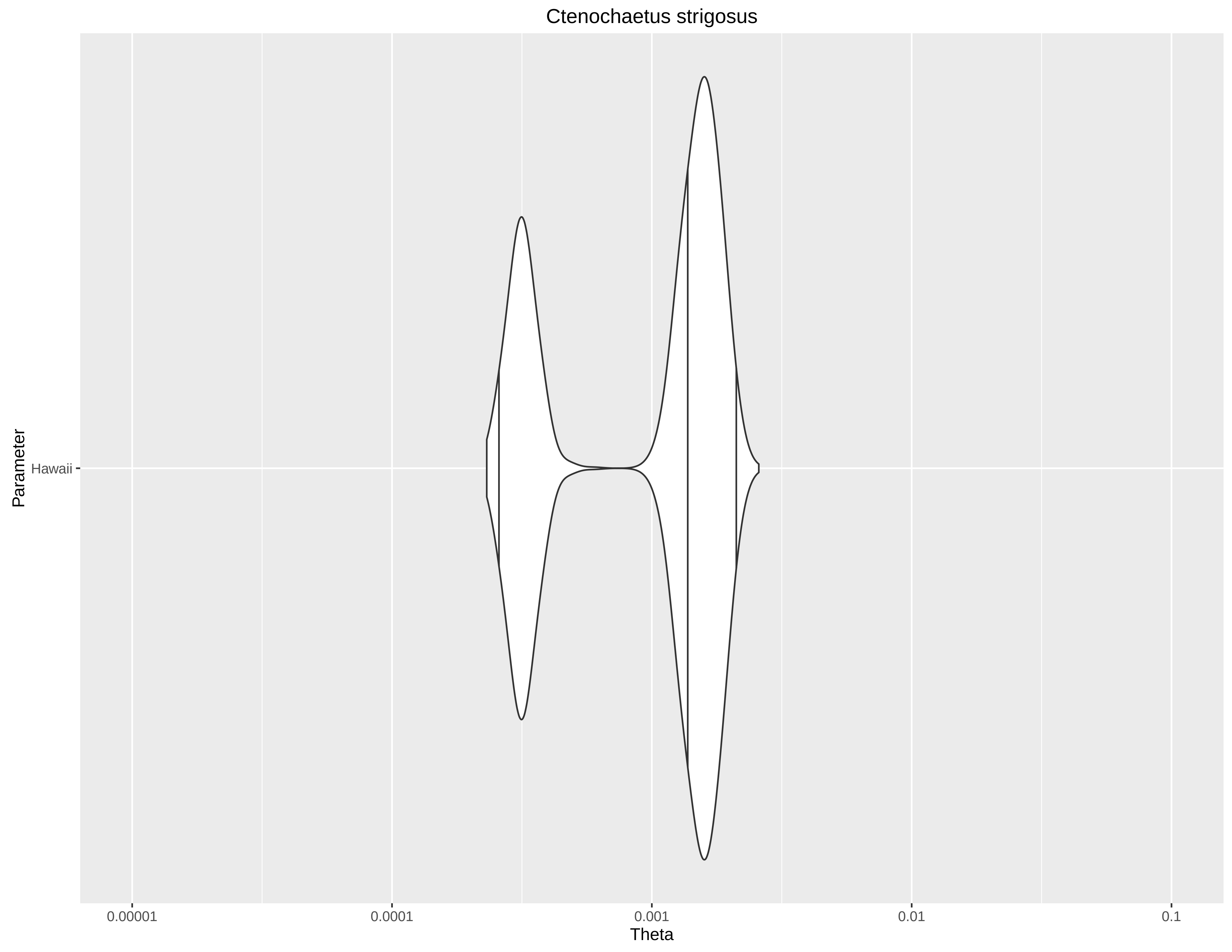
Ctenochaetus strigosus
Parameter
Hawaii
0.00001
0.0001
0.001
0.01
0.1
Theta

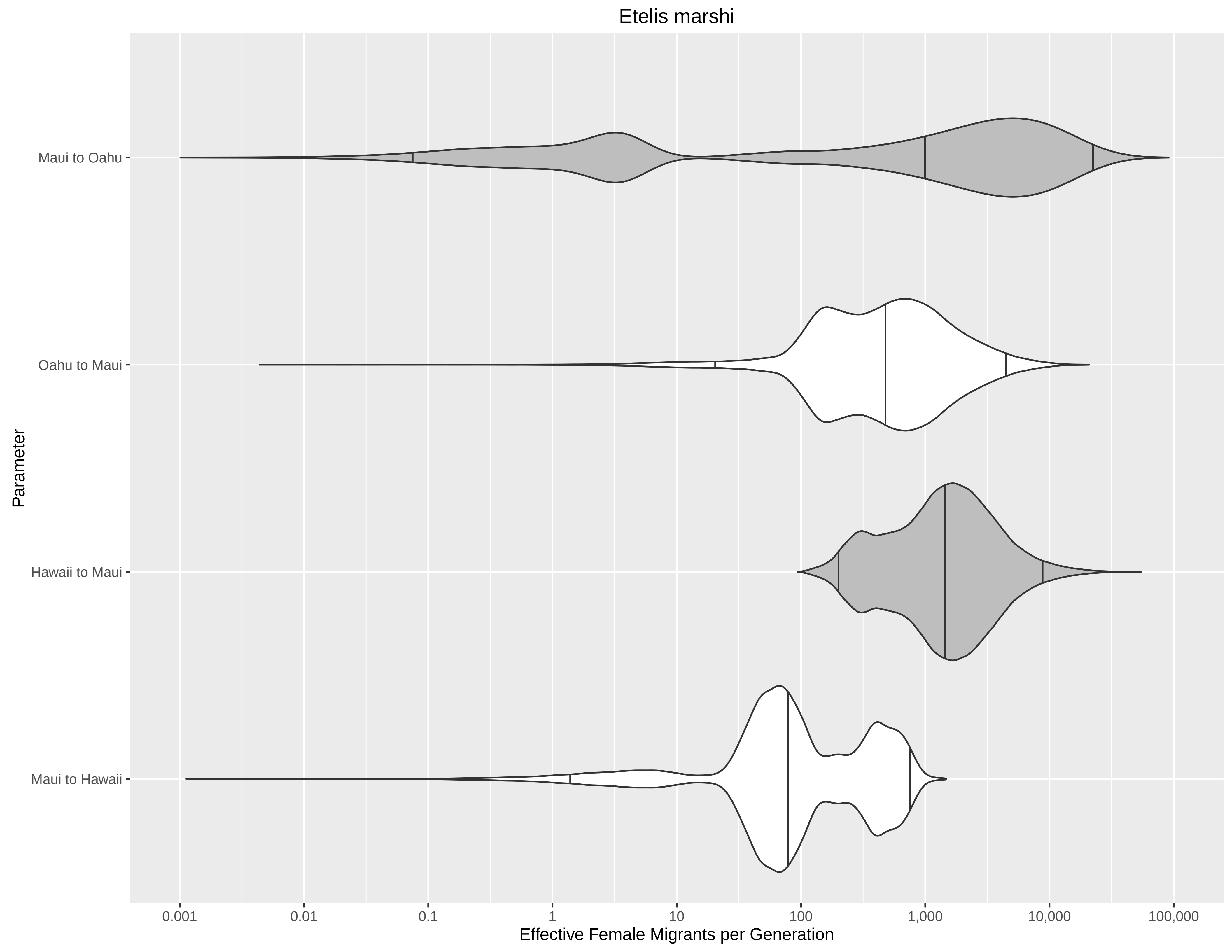

Etelis marshi
Maui to Oahu
Oahu to Maui
Hawaii to Maui
Maui to Hawaii
Parameter
0.001
0.01
0.1
1
10
100
1,000
10,000
100,000
Effective Female Migrants per Generation

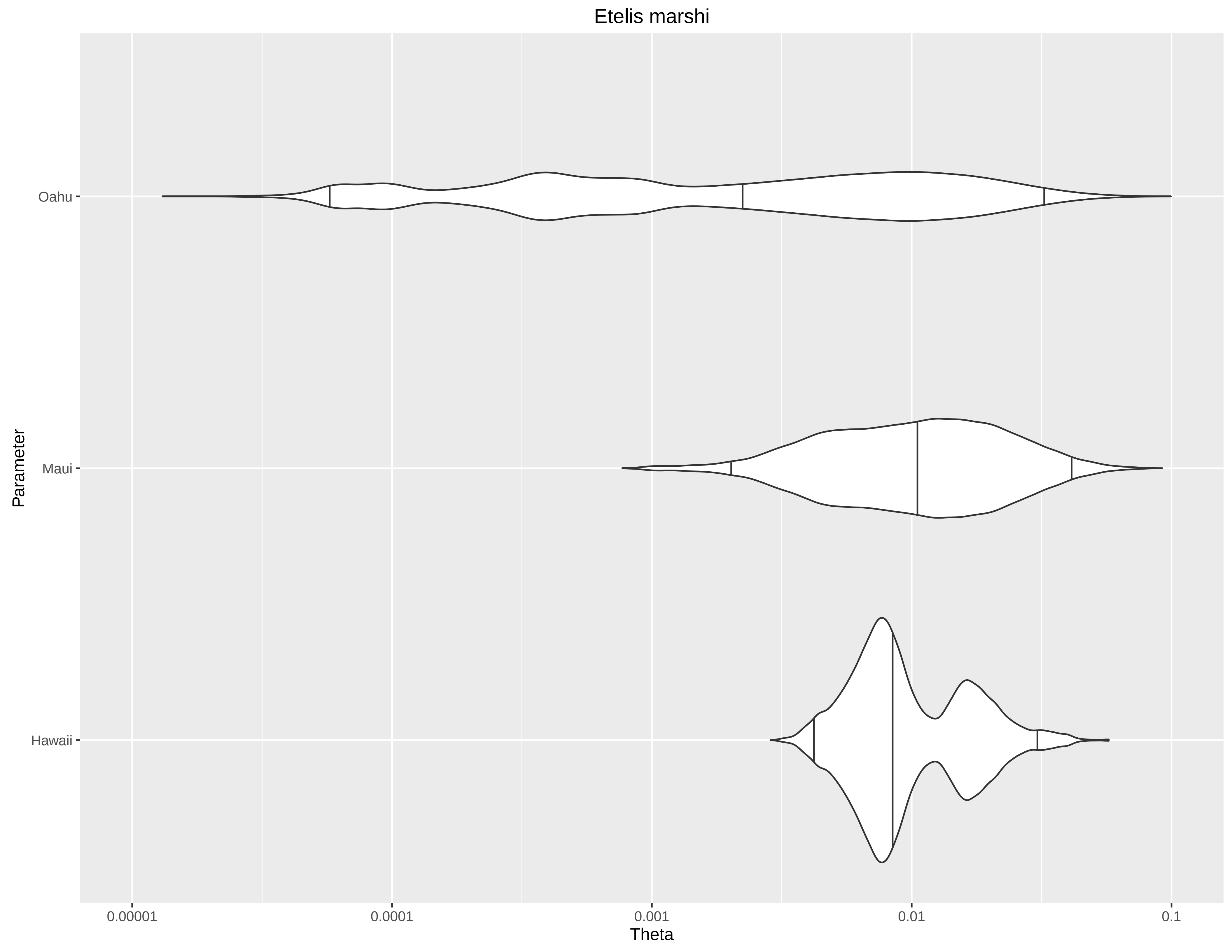

Etelis marshi
Parameter
Oahu
Maui
Hawaii
0.00001
0.0001
0.001
0.01
0.1
Theta

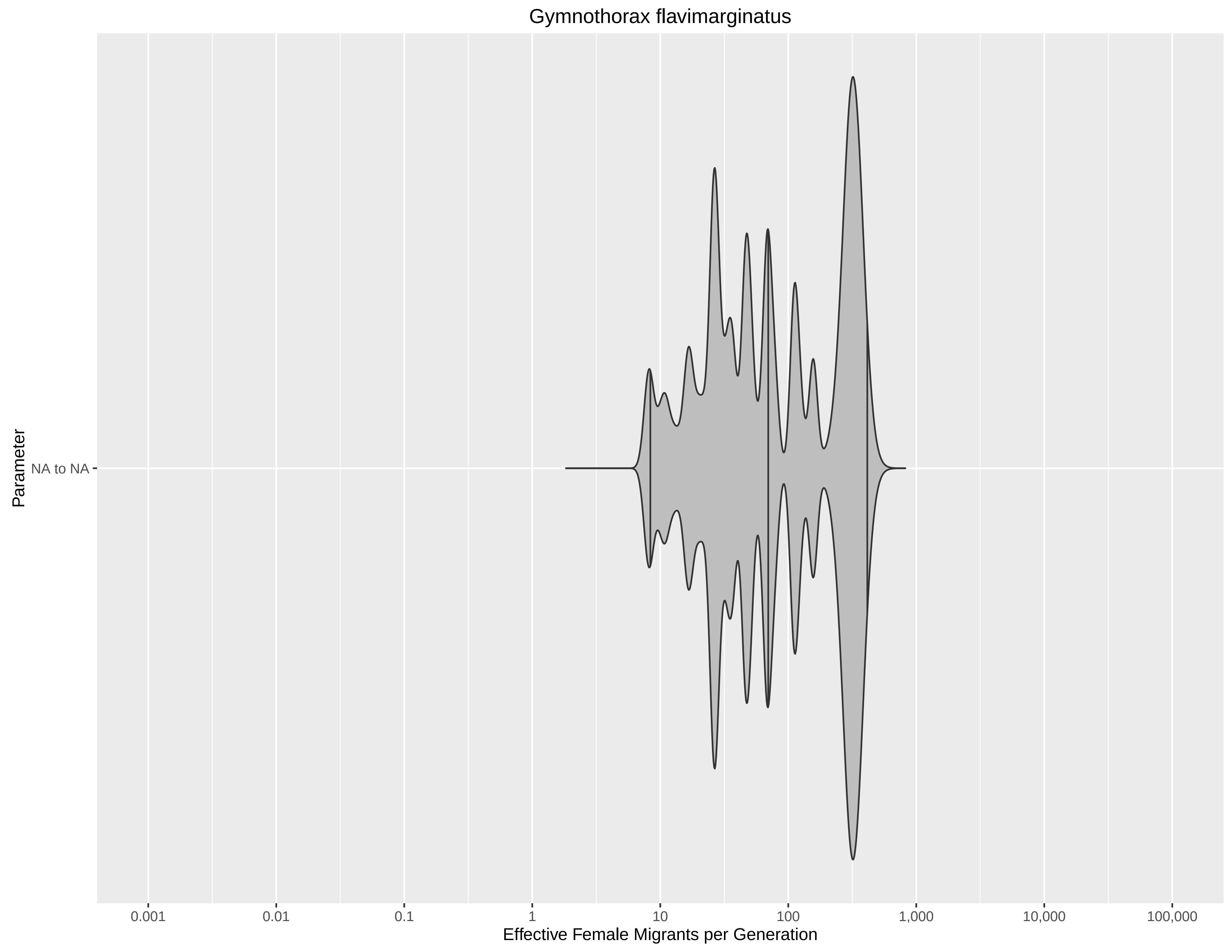
Gymnothorax flavimarginatus
Parameter
NA to NA
0.001
0.01
0.1
1
10
100
1,000
10,000
100,000
Effective Female Migrants per Generation

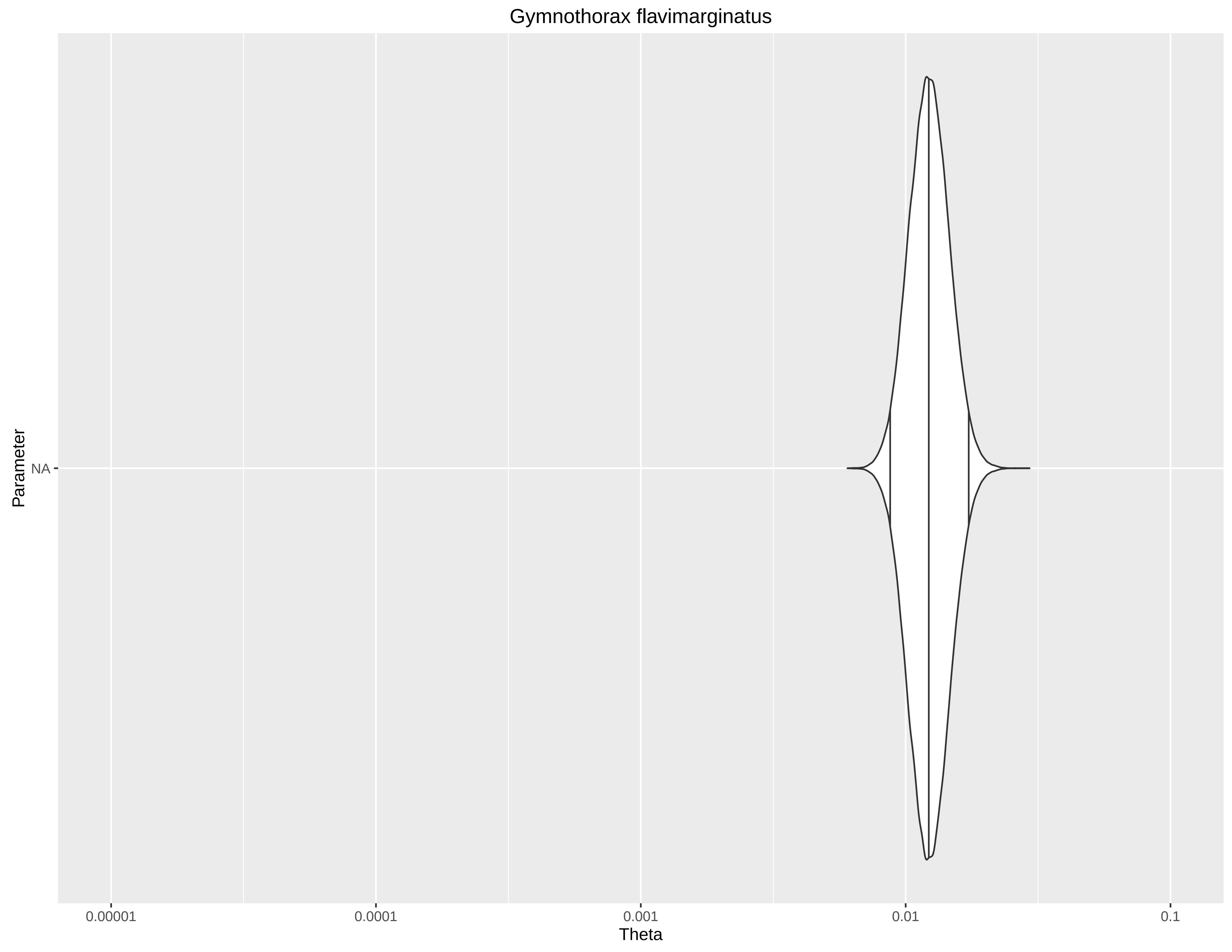

Gymnothorax flavimarginatus
Parameter
NA
0.00001
0.0001
0.001
0.01
0.1
Theta

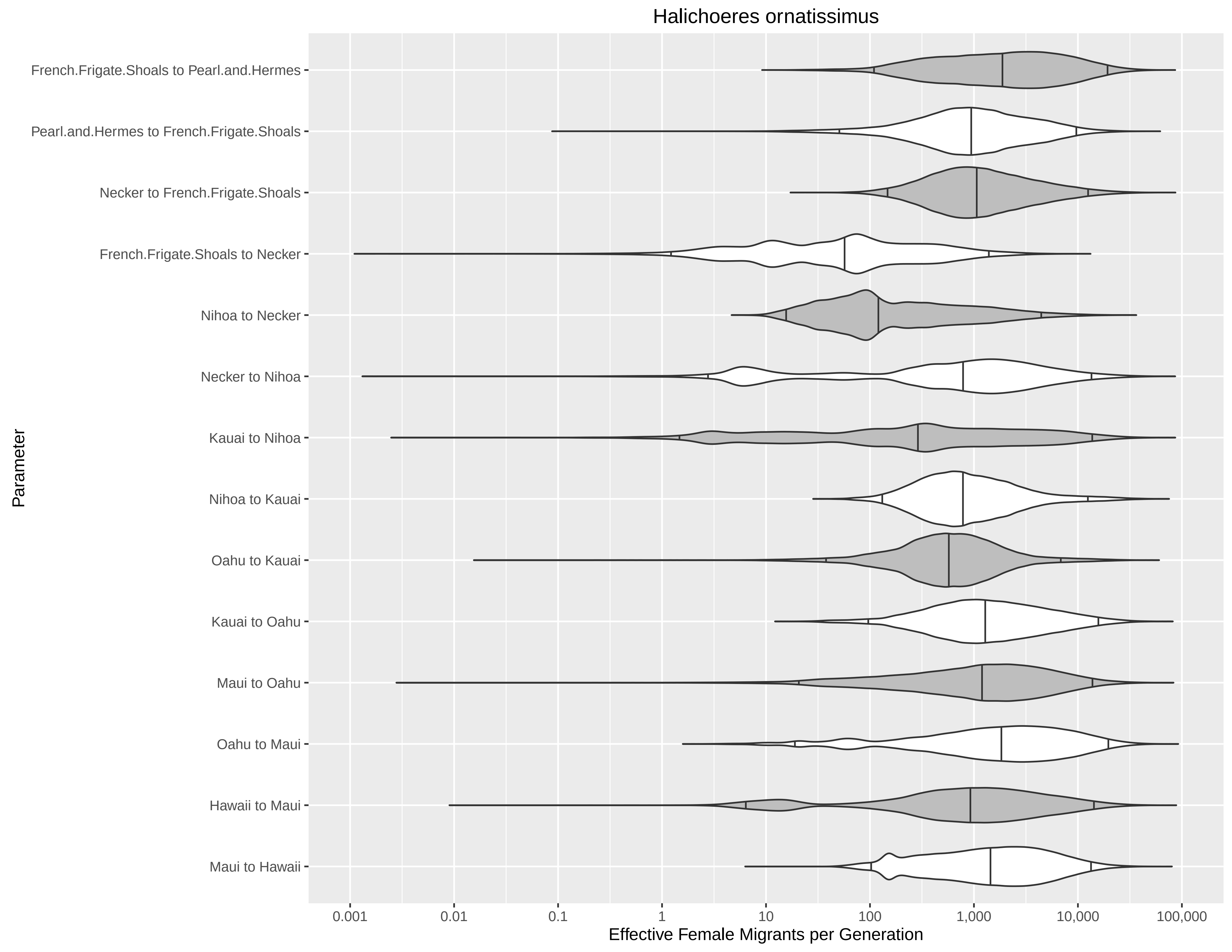

Halichoeres ornatissimus
French.Frigate.Shoals to Pearl.and.Hermes
Pearl.and.Hermes to French.Frigate.Shoals
Necker to French.Frigate.Shoals
French.Frigate.Shoals to Necker
Nihoa to Necker
Necker to Nihoa
Kauai to Nihoa
Nihoa to Kauai
Oahu to Kauai
Kauai to Oahu
Maui to Oahu
Oahu to Maui
Hawaii to Maui
Maui to Hawaii
Parameter
0.001
0.01
0.1
1
10
100
1,000
10,000
100,000
Effective Female Migrants per Generation

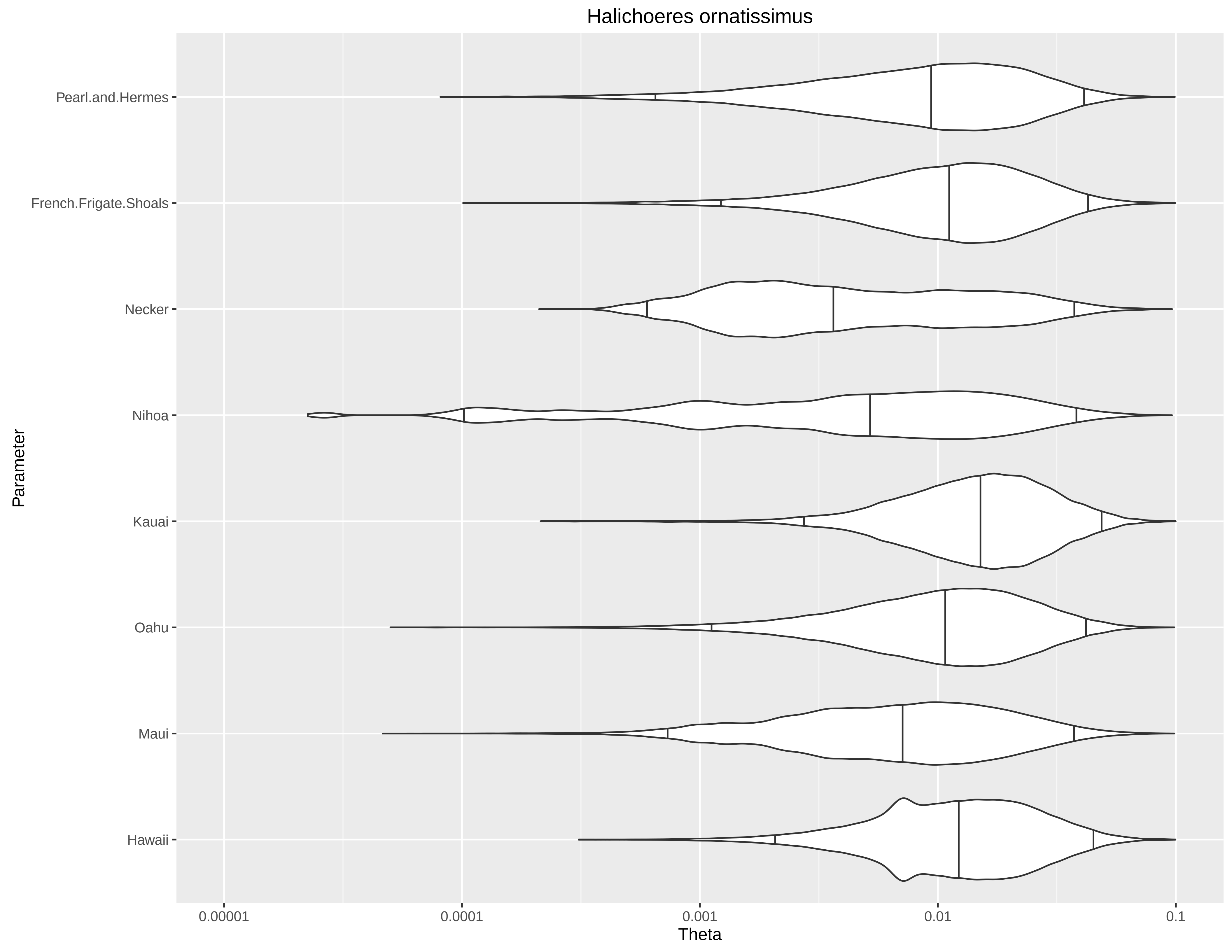
Halichoeres ornatissimus
Parameter
Pearl.and.Hermes
French.Frigate.Shoals
Necker
Nihoa
Kauai
Oahu
Maui
Hawaii
0.00001
0.0001
0.001
0.01
0.1
Theta

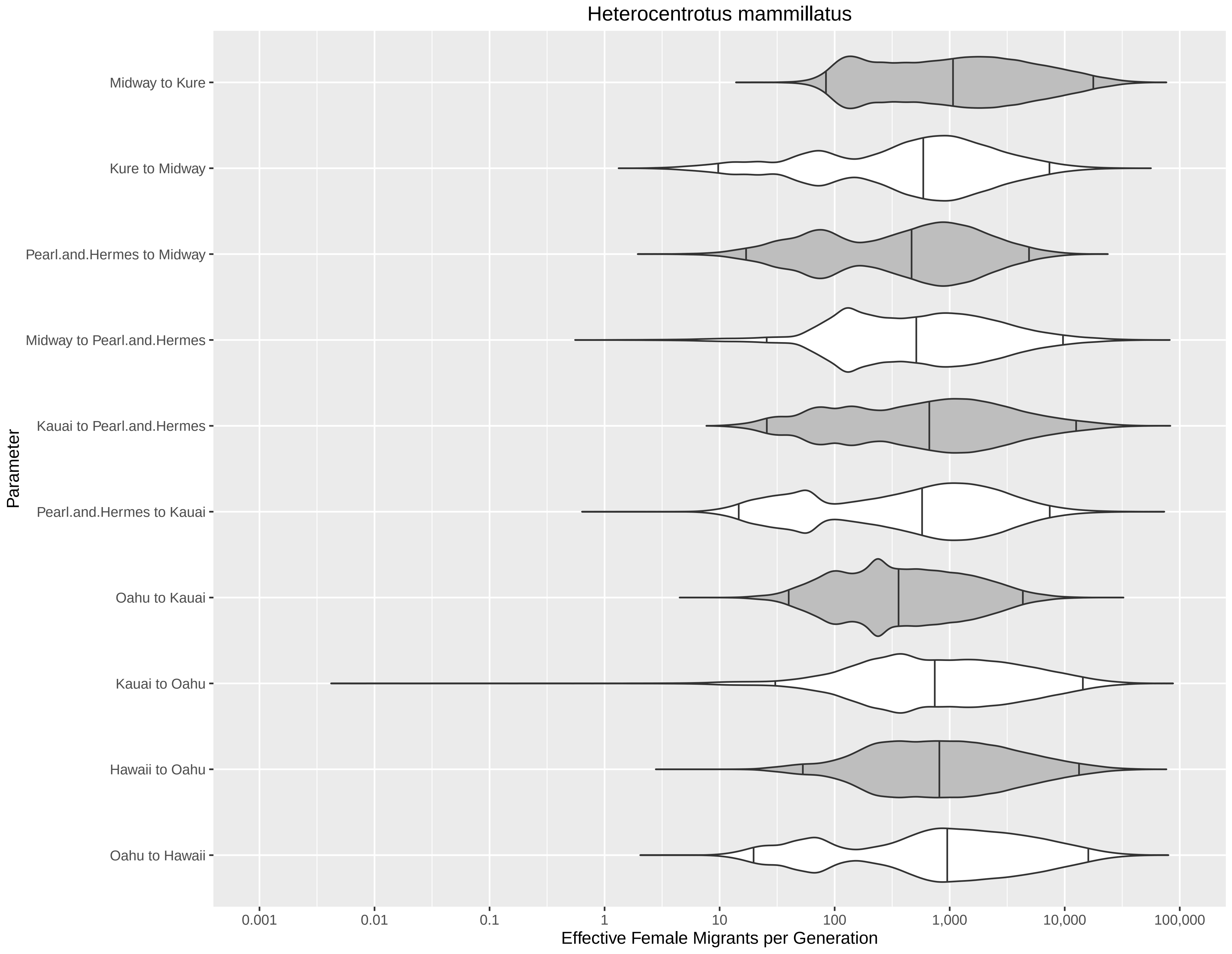
Heterocentrotus mammillatus
Midway to Kure
Kure to Midway
Pearl.and.Hermes to Midway
Midway to Pearl.and.Hermes
Kauai to Pearl.and.Hermes
Pearl.and.Hermes to Kauai
Oahu to Kauai
Kauai to Oahu
Hawaii to Oahu
Oahu to Hawaii
Parameter
0.001
0.01
0.1
1
10
100
1,000
10,000
100,000
Effective Female Migrants per Generation

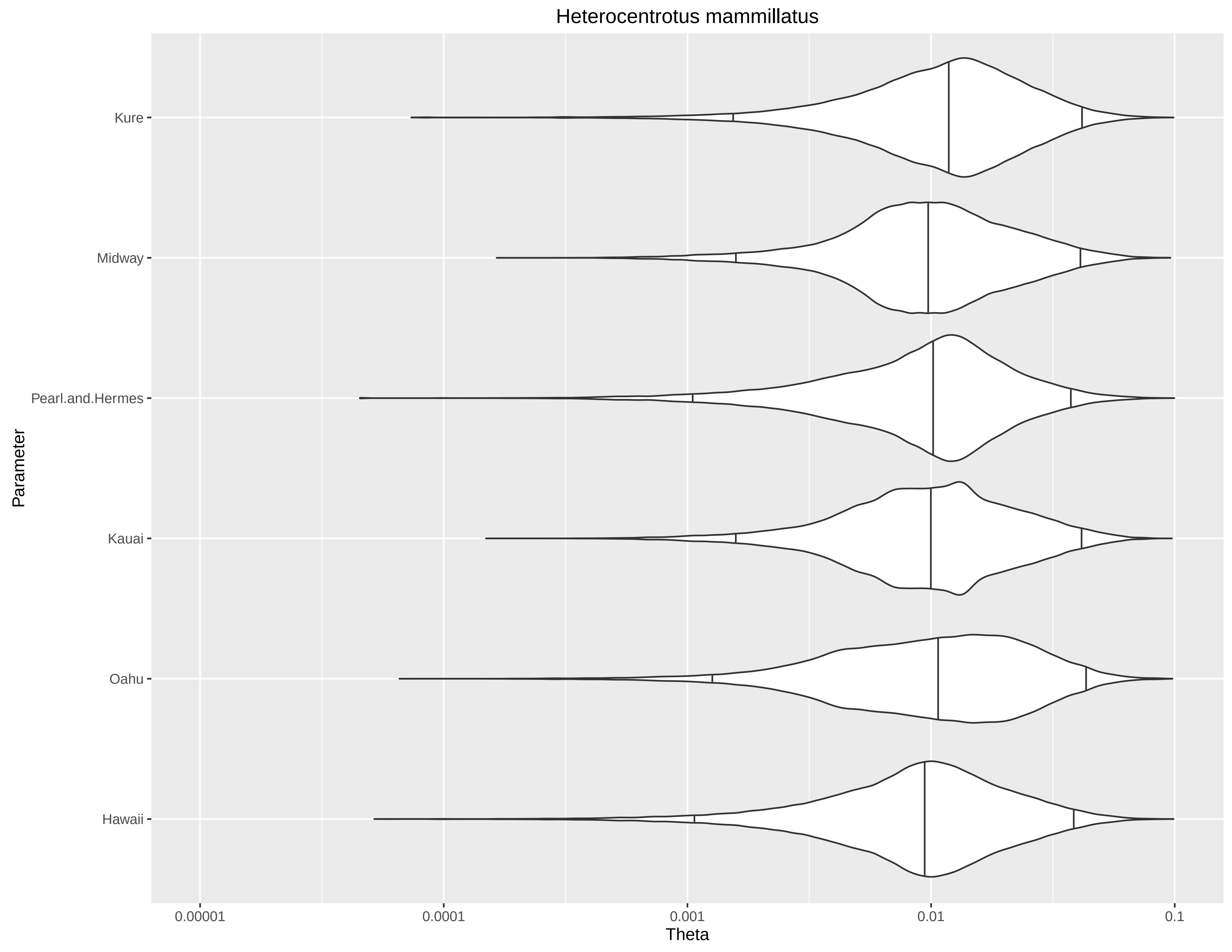

Heterocentrotus mammillatus
Kure
Midway
Pearl.and.Hermes
Kauai
Oahu
Hawaii
Parameter
0.00001
0.0001
0.001
0.01
0.1
Theta

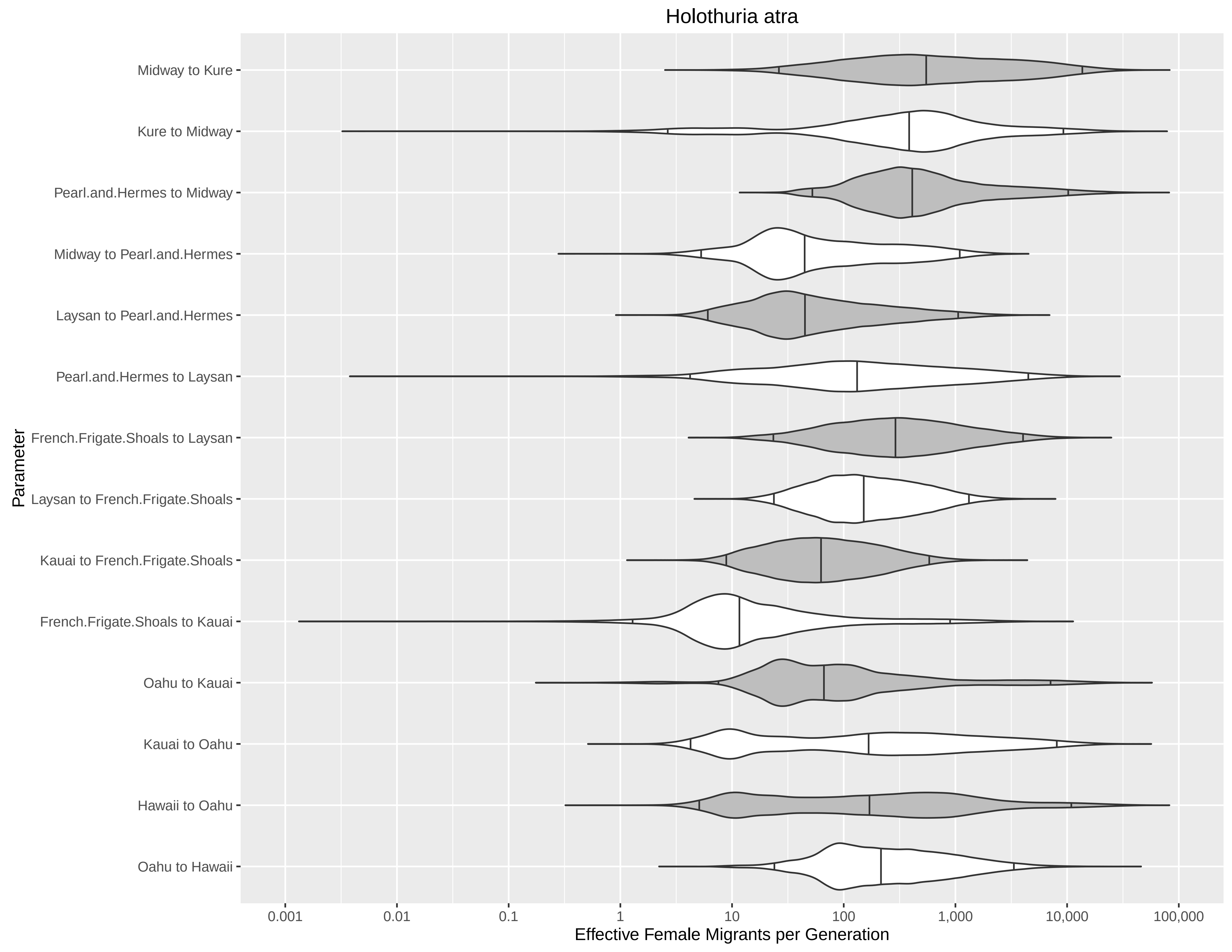

Holothuria atra
Midway to Kure
Kure to Midway
Pearl.and.Hermes to Midway
Midway to Pearl.and.Hermes
Laysan to Pearl.and.Hermes
Pearl.and.Hermes to Laysan
French.Frigate.Shoals to Laysan
Laysan to French.Frigate.Shoals
Kauai to French.Frigate.Shoals
French.Frigate.Shoals to Kauai
Oahu to Kauai
Kauai to Oahu
Hawaii to Oahu
Oahu to Hawaii
Parameter
0.001
0.01
0.1
1
10
100
1,000
10,000
100,000
Effective Female Migrants per Generation

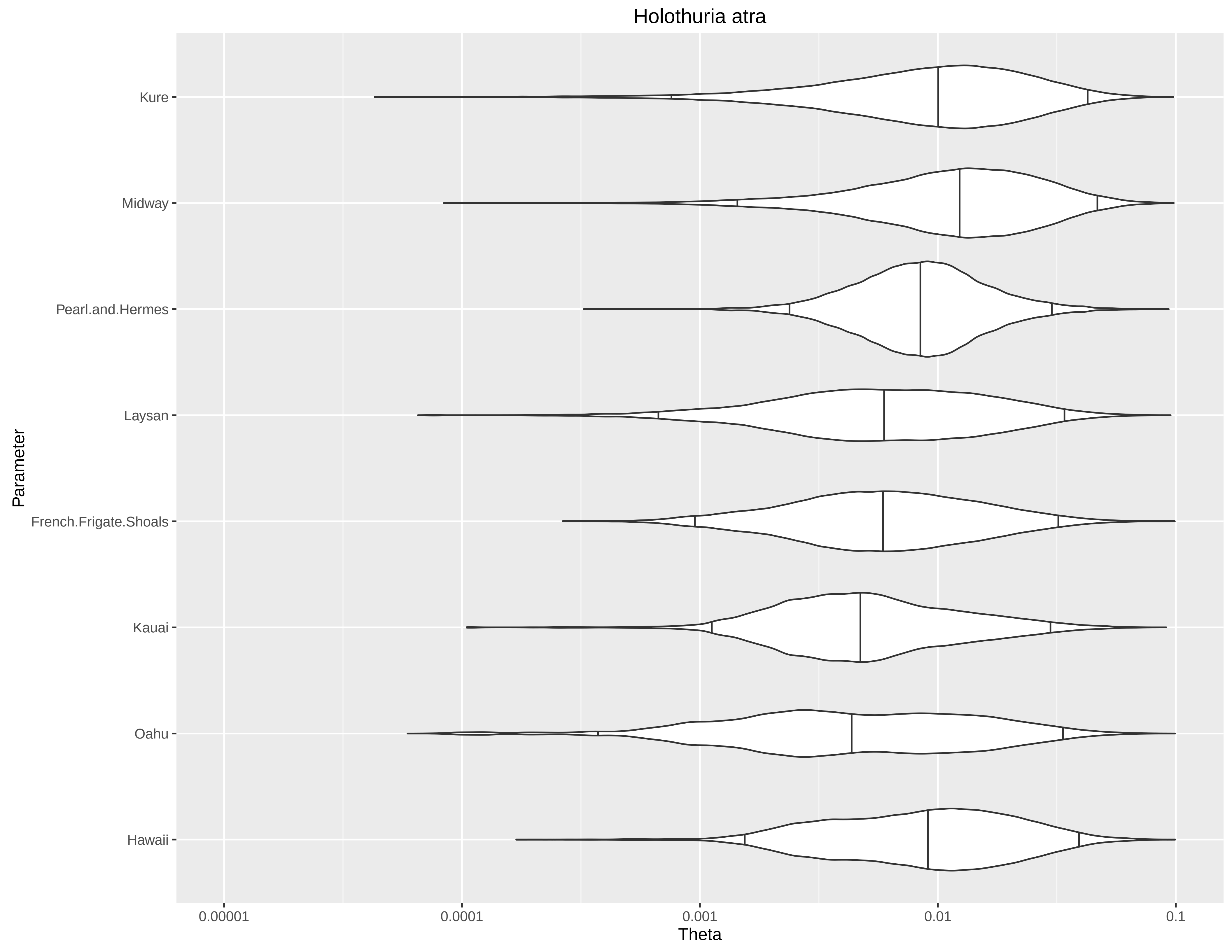

Holothuria atra
Kure
Midway
Pearl.and.Hermes
Laysan
French.Frigate.Shoals
Kauai
Oahu
Hawaii
Parameter
0.00001
0.0001
0.001
0.01
0.1
Theta

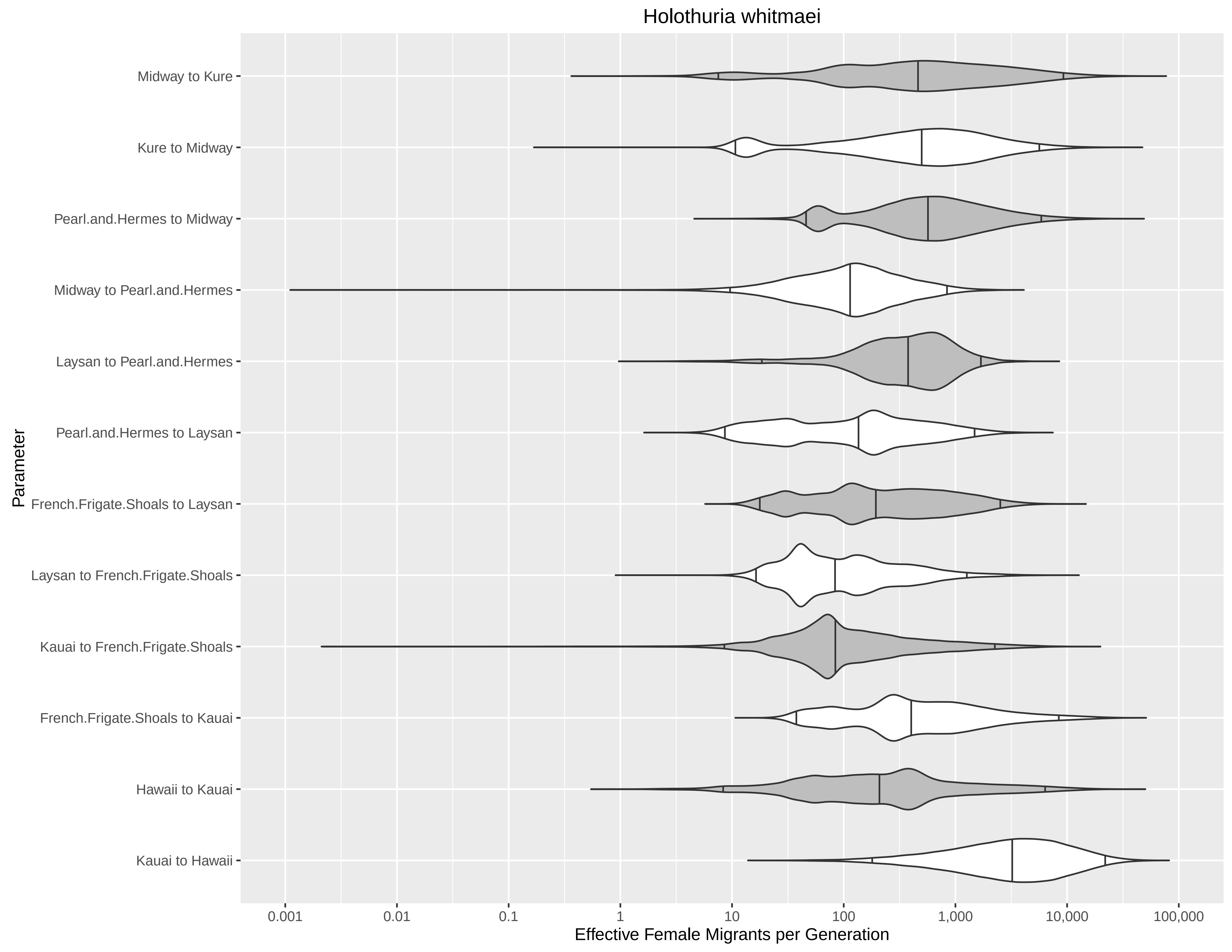

Holothuria whitmaei
Midway to Kure
Kure to Midway
Pearl.and.Hermes to Midway
Midway to Pearl.and.Hermes
Laysan to Pearl.and.Hermes
Pearl.and.Hermes to Laysan
French.Frigate.Shoals to Laysan
Laysan to French.Frigate.Shoals
Kauai to French.Frigate.Shoals
French.Frigate.Shoals to Kauai
Hawaii to Kauai
Kauai to Hawaii
Parameter
0.001
0.01
0.1
1
10
100
1,000
10,000
100,000
Effective Female Migrants per Generation

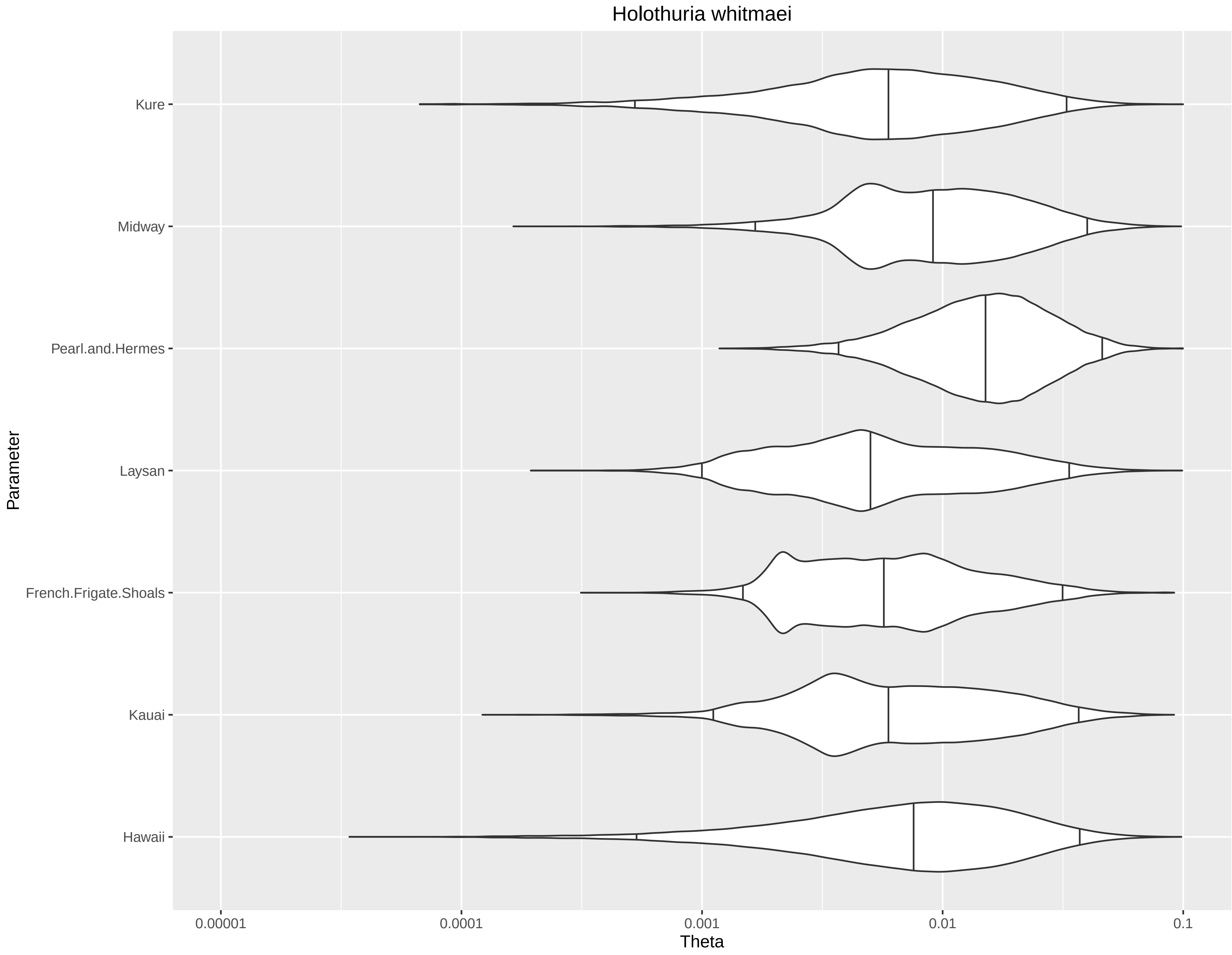

Holothuria whitmaei
Kure
Midway
Pearl.and.Hermes
Laysan
French.Frigate.Shoals
Kauai
Hawaii
Parameter
0.00001
0.0001
0.001
0.01
0.1
Theta

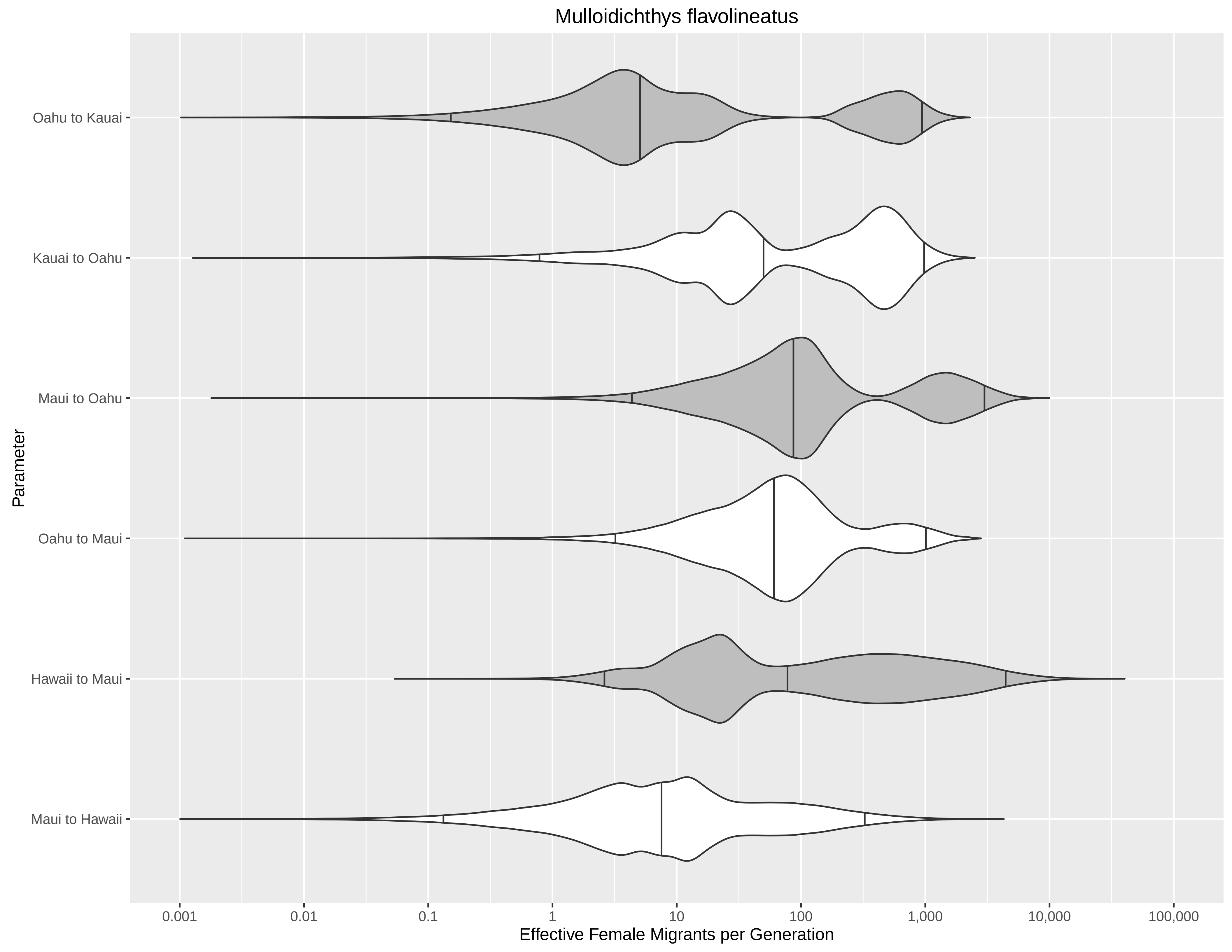
Mulloidichthys flavolineatus
Oahu to Kauai
Kauai to Oahu
Maui to Oahu
Oahu to Maui
Hawaii to Maui
Maui to Hawaii
Parameter
0.001
0.01
0.1
1
10
100
1,000
10,000
100,000
Effective Female Migrants per Generation

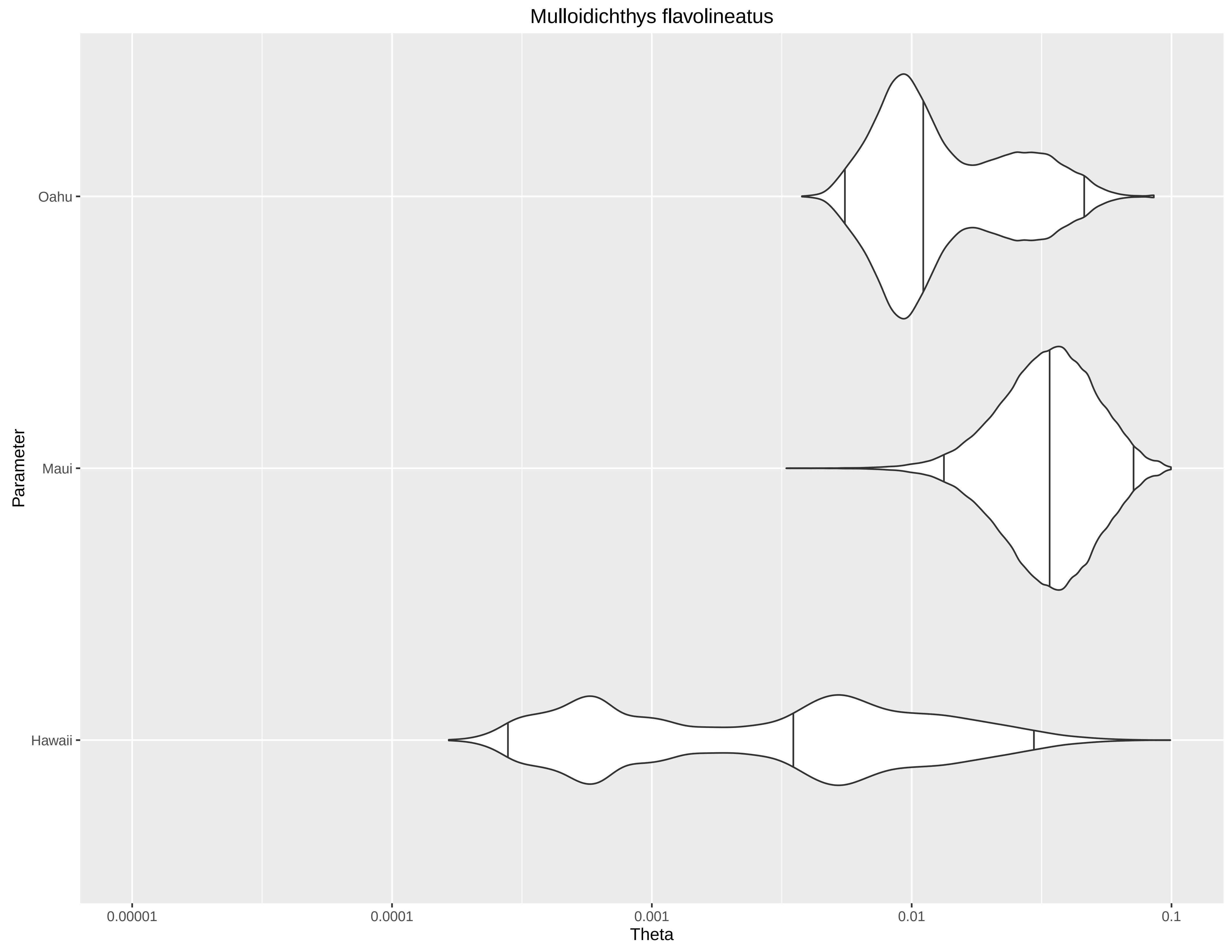

Mulloidichthys flavolineatus
Oahu
Maui
Hawaii
Parameter
0.00001
0.0001
0.001
0.01
0.1
Theta

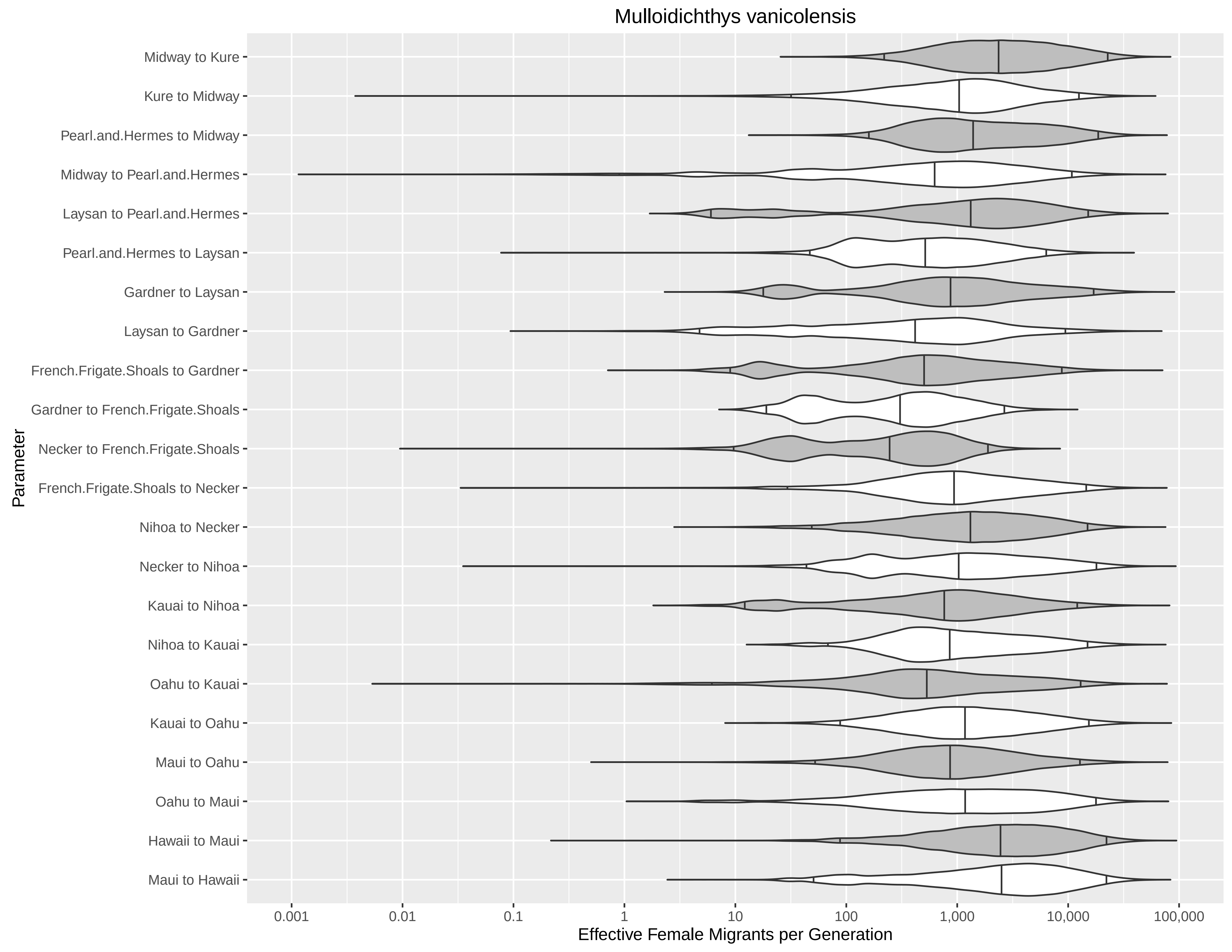

Mulloidichthys vanicolensis
Parameter
Midway to Kure
Kure to Midway
Pearl.and.Hermes to Midway
Midway to Pearl.and.Hermes
Laysan to Pearl.and.Hermes
Pearl.and.Hermes to Laysan
Gardner to Laysan
Laysan to Gardner
French.Frigate.Shoals to Gardner
Gardner to French.Frigate.Shoals
Necker to French.Frigate.Shoals
French.Frigate.Shoals to Necker
Nihoa to Necker
Necker to Nihoa
Kauai to Nihoa
Nihoa to Kauai
Oahu to Kauai
Kauai to Oahu
Maui to Oahu
Oahu to Maui
Hawaii to Maui
Maui to Hawaii
0.001
0.01
0.1
1
10
100
1,000
10,000
100,000
Effective Female Migrants per Generation

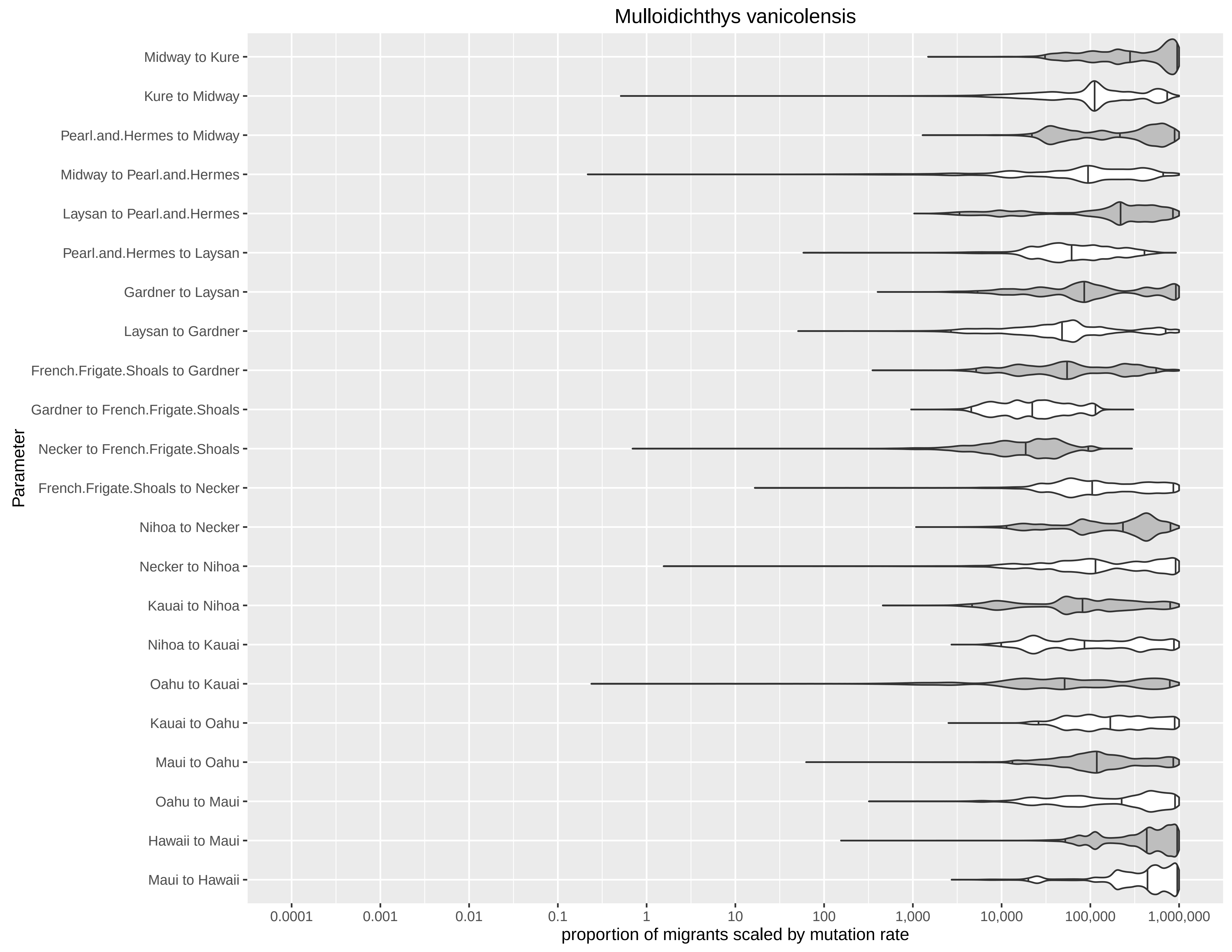

Mulloidichthys vanicolensis
Midway to Kure
Kure to Midway
Pearl.and.Hermes to Midway
Midway to Pearl.and.Hermes
Laysan to Pearl.and.Hermes
Pearl.and.Hermes to Laysan
Gardner to Laysan
Laysan to Gardner
French.Frigate.Shoals to Gardner
Gardner to French.Frigate.Shoals
Necker to French.Frigate.Shoals
French.Frigate.Shoals to Necker
Nihoa to Necker
Necker to Nihoa
Kauai to Nihoa
Nihoa to Kauai
Oahu to Kauai
Kauai to Oahu
Maui to Oahu
Oahu to Maui
Hawaii to Maui
Maui to Hawaii
Parameter
0.0001
0.001
0.01
0.1
1
10
100
1,000
10,000
100,000
1,000,000
proportion of migrants scaled by mutation rate

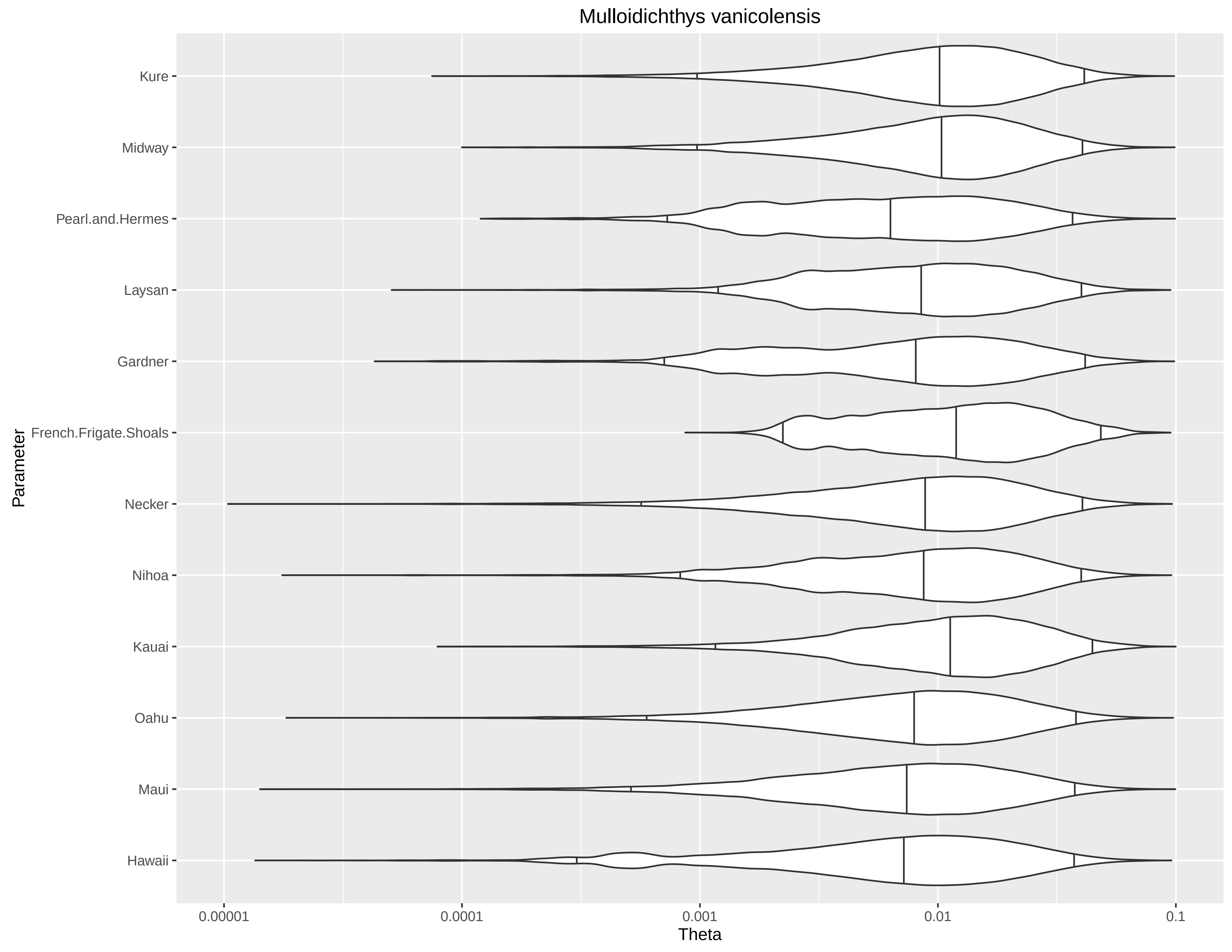

Mulloidichthys vanicolensis
Kure
Midway
Pearl.and.Hermes
Laysan
Gardner
French.Frigate.Shoals
Necker
Nihoa
Kauai
Oahu
Maui
Hawaii
Parameter
0.00001
0.0001
0.001
0.01
0.1
Theta

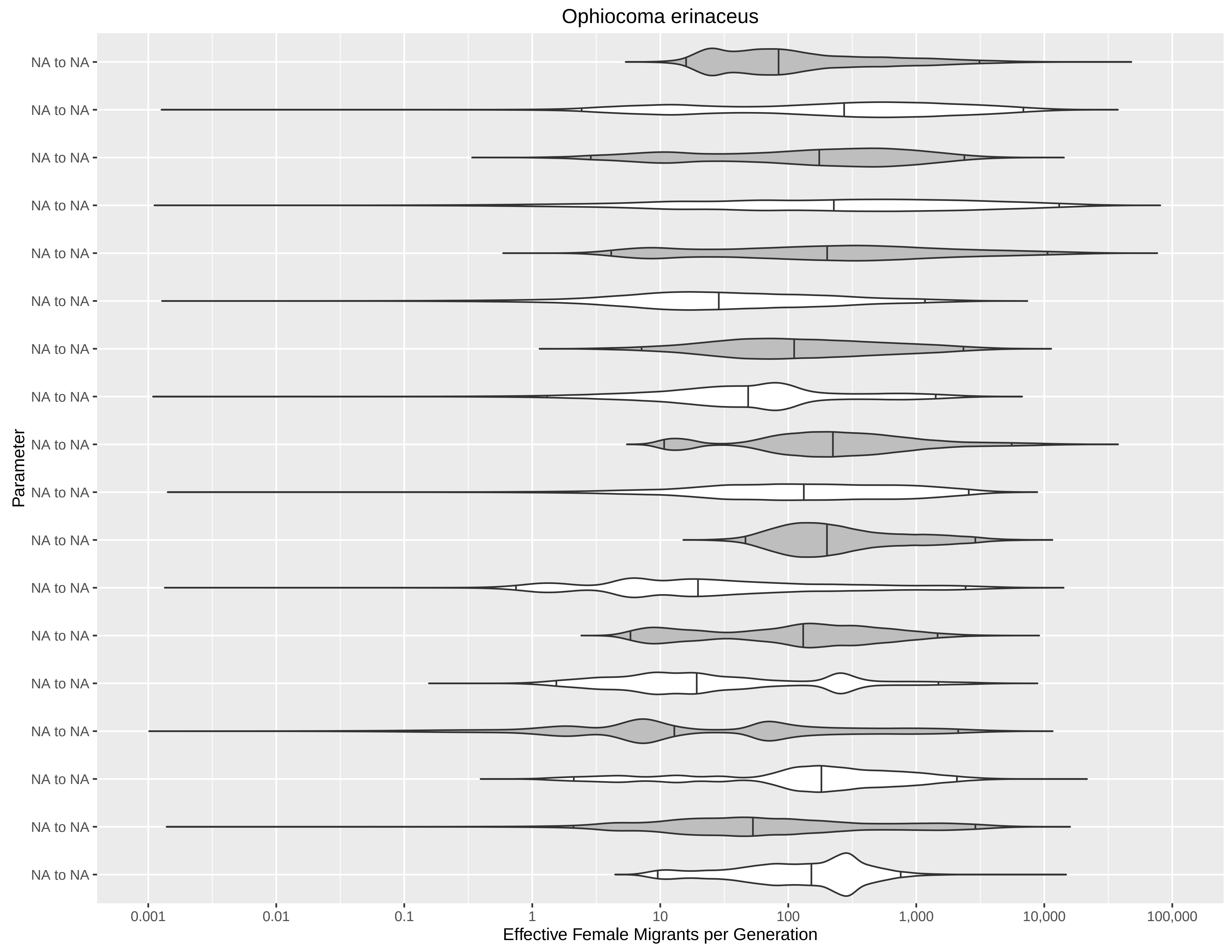
Ophiocoma erinaceus
NA to NA
NA to NA
NA to NA
NA to NA
NA to NA
NA to NA
NA to NA
NA to NA
NA to NA
NA to NA
NA to NA
NA to NA
NA to NA
NA to NA
NA to NA
NA to NA
NA to NA
NA to NA
Parameter
0.001
0.01
0.1
1
10
100
1,000
10,000
100,000
Effective Female Migrants per Generation

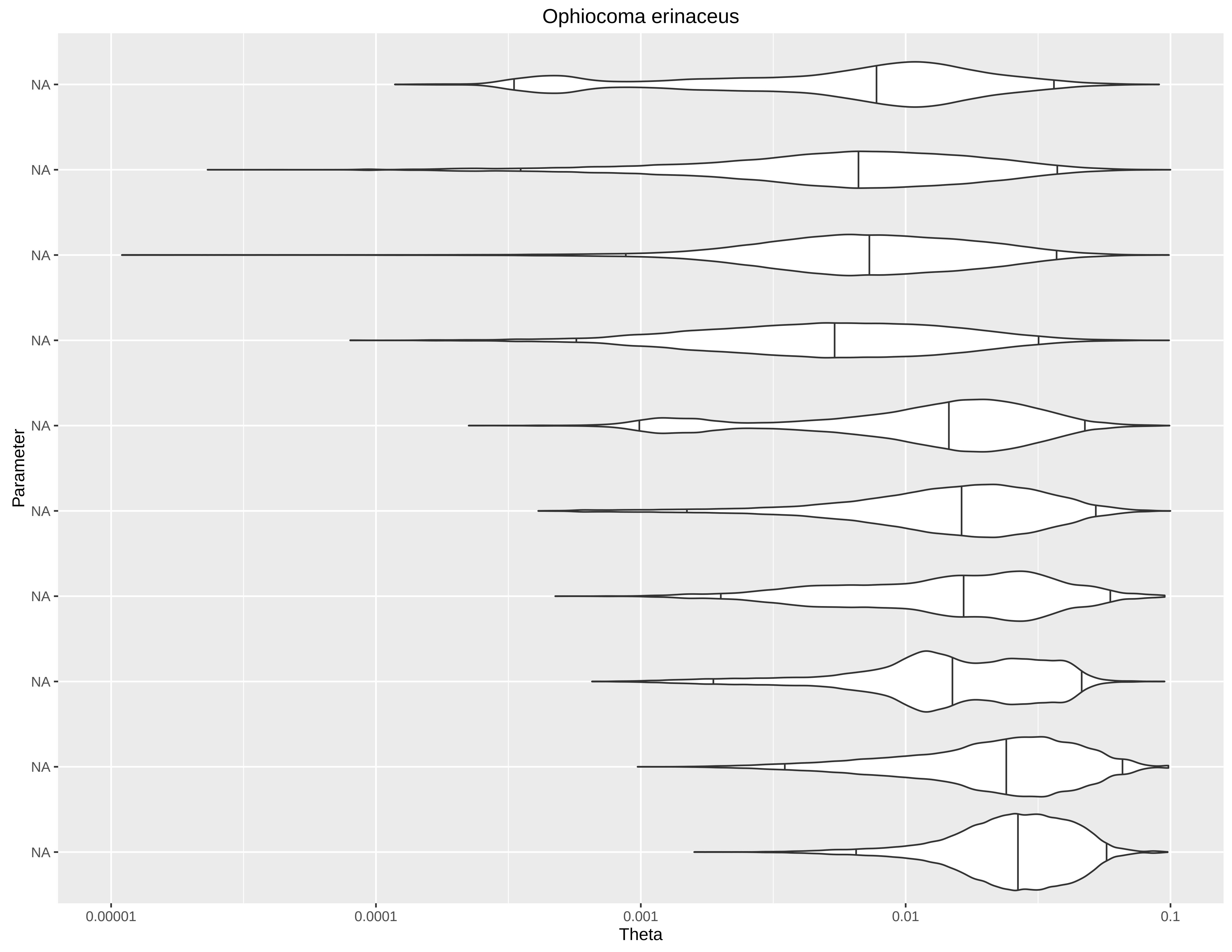
Ophiocoma erinaceus
Parameter
NA
NA
NA
NA
NA
NA
NA
NA
NA
NA
0.00001
0.0001
0.001
0.01
0.1
Theta

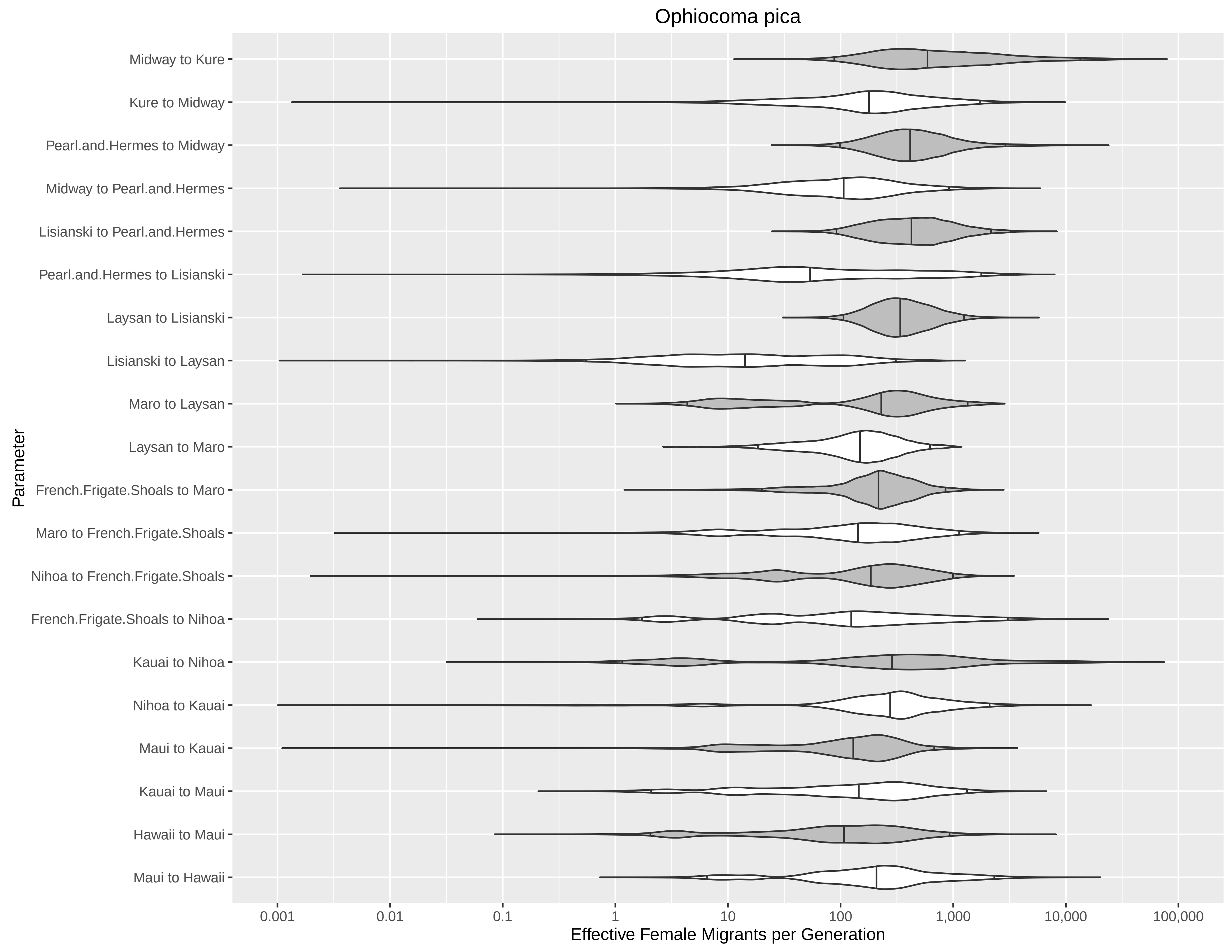

Ophiocoma pica
Parameter
Midway to Kure
Kure to Midway
Pearl.and.Hermes to Midway
Midway to Pearl.and.Hermes
Lisianski to Pearl.and.Hermes
Pearl.and.Hermes to Lisianski
Laysan to Lisianski
Lisianski to Laysan
Maro to Laysan
Laysan to Maro
French.Frigate.Shoals to Maro
Maro to French.Frigate.Shoals
Nihoa to French.Frigate.Shoals
French.Frigate.Shoals to Nihoa
Kauai to Nihoa
Nihoa to Kauai
Maui to Kauai
Kauai to Maui
Hawaii to Maui
Maui to Hawaii
0.001
0.01
0.1
1
10
100
1,000
10,000
100,000
Effective Female Migrants per Generation

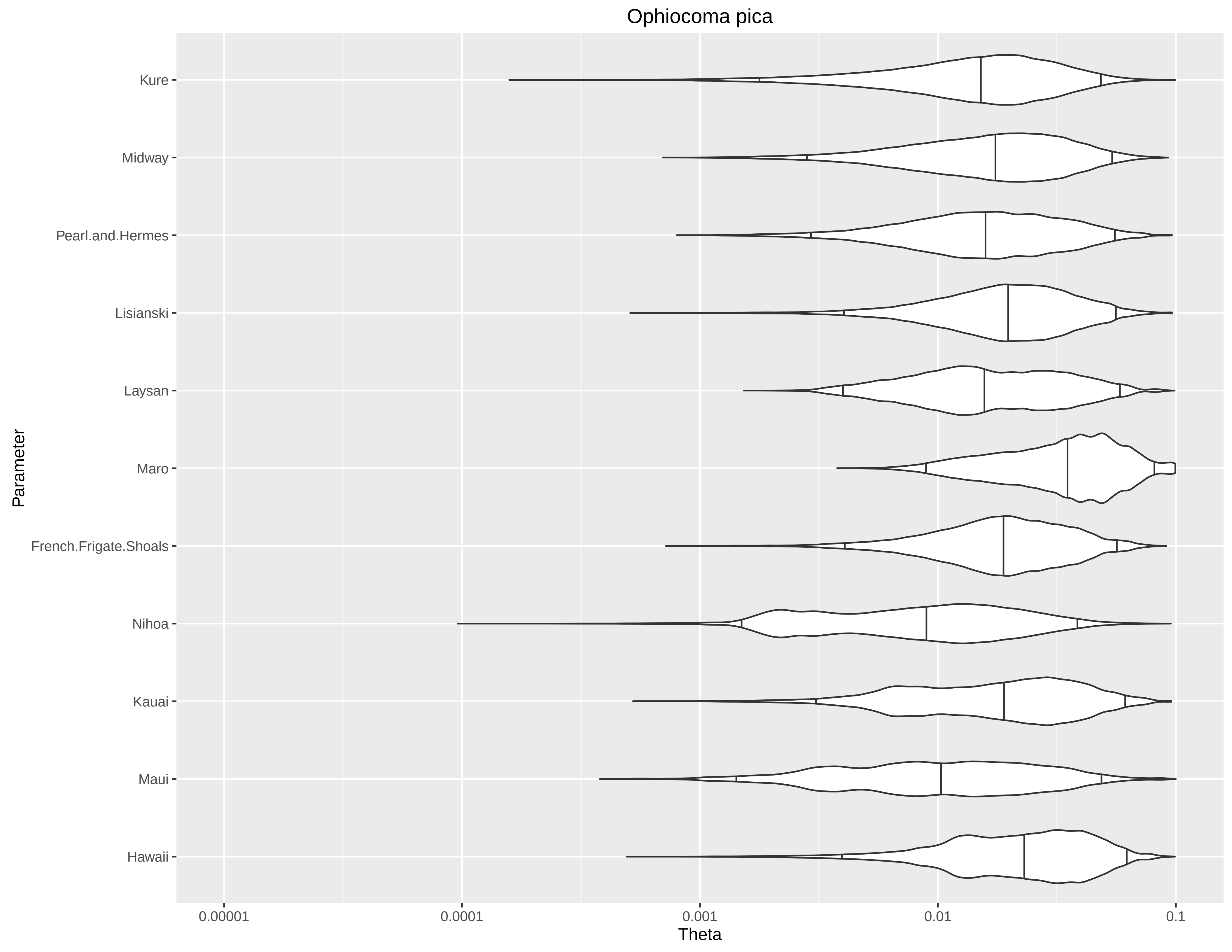

Ophiocoma pica
Kure
Midway
Pearl.and.Hermes
Lisianski
Laysan
Maro
French.Frigate.Shoals
Nihoa
Kauai
Maui
Hawaii
Parameter
0.00001
0.0001
0.001
0.01
0.1
Theta

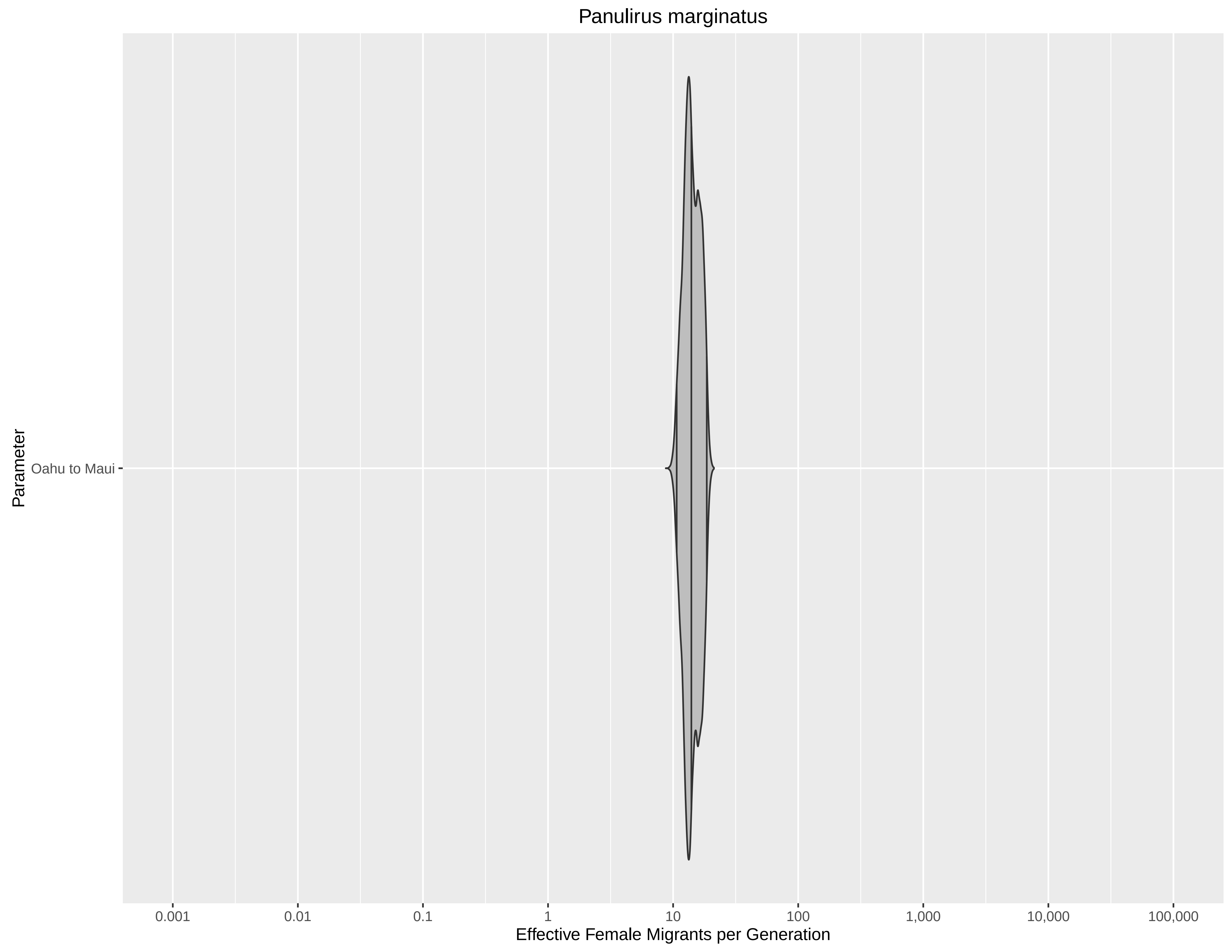

Panulirus marginatus
Parameter
Oahu to Maui
0.001
0.01
0.1
1
10
100
1,000
10,000
100,000
Effective Female Migrants per Generation

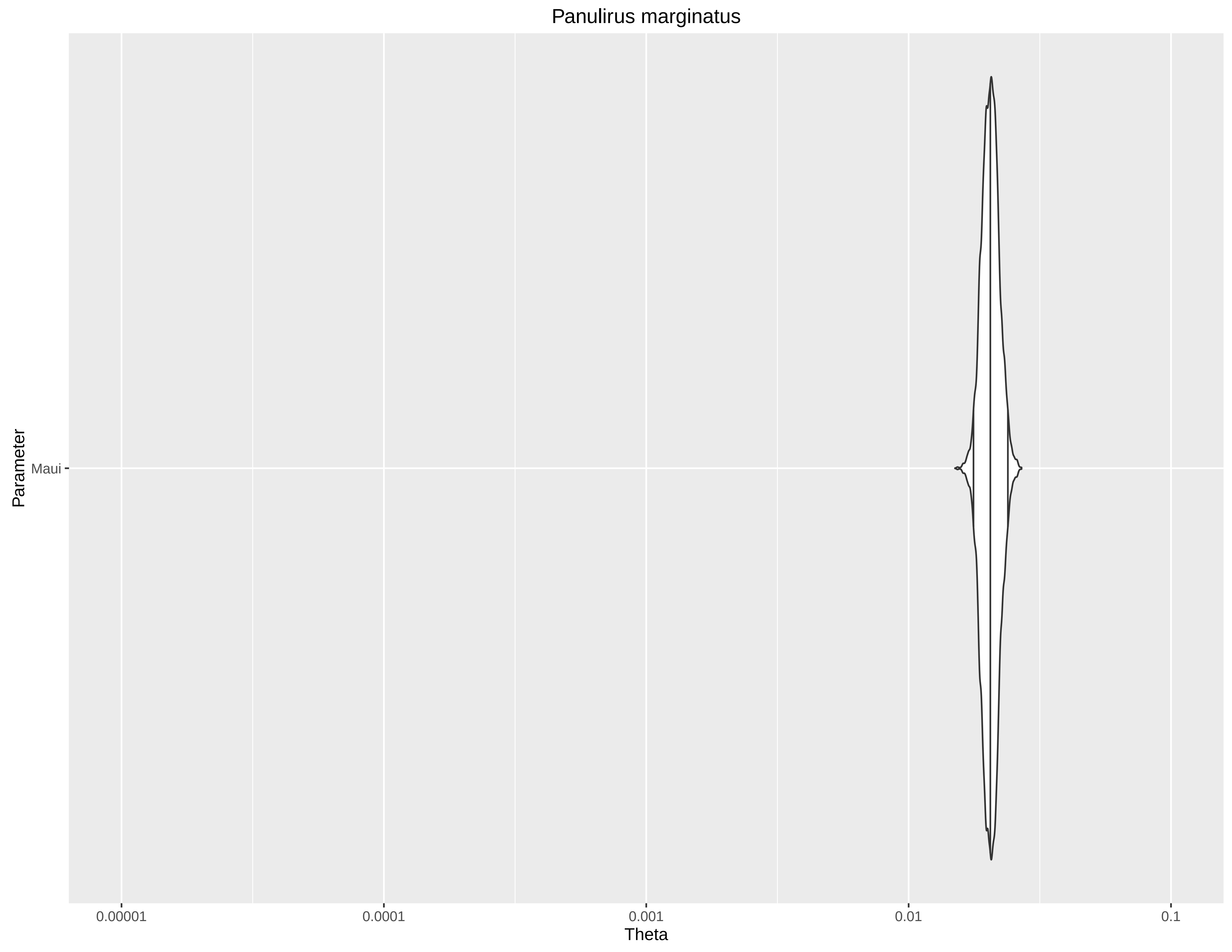
Panulirus marginatus
Parameter
Maui
0.00001
0.0001
0.001
0.01
0.1
Theta

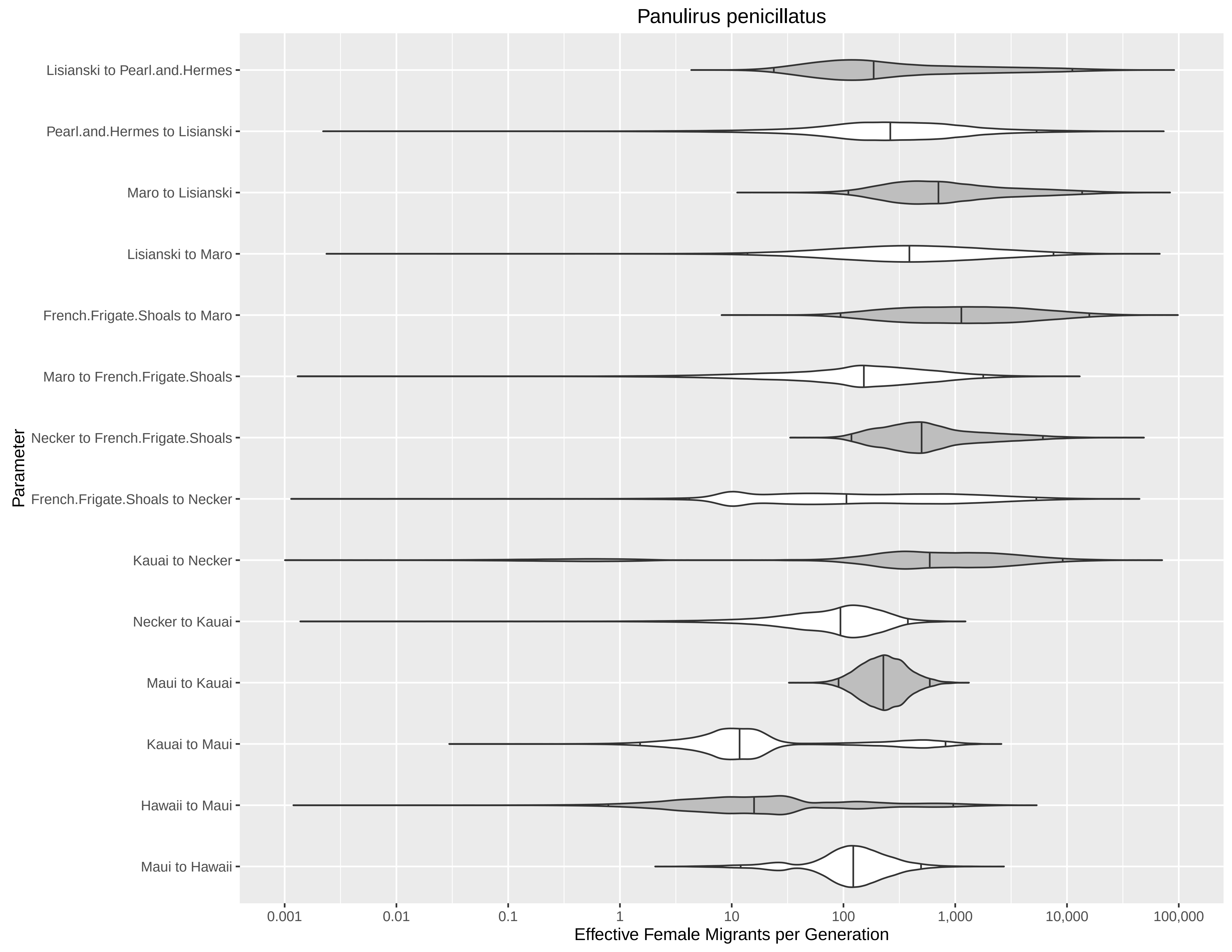
Panulirus penicillatus
Parameter
Lisianski to Pearl.and.Hermes
Pearl.and.Hermes to Lisianski
Maro to Lisianski
Lisianski to Maro
French.Frigate.Shoals to Maro
Maro to French.Frigate.Shoals
Necker to French.Frigate.Shoals
French.Frigate.Shoals to Necker
Kauai to Necker
Necker to Kauai
Maui to Kauai
Kauai to Maui
Hawaii to Maui
Maui to Hawaii
0.001
0.01
0.1
1
10
100
1,000
10,000
100,000
Effective Female Migrants per Generation

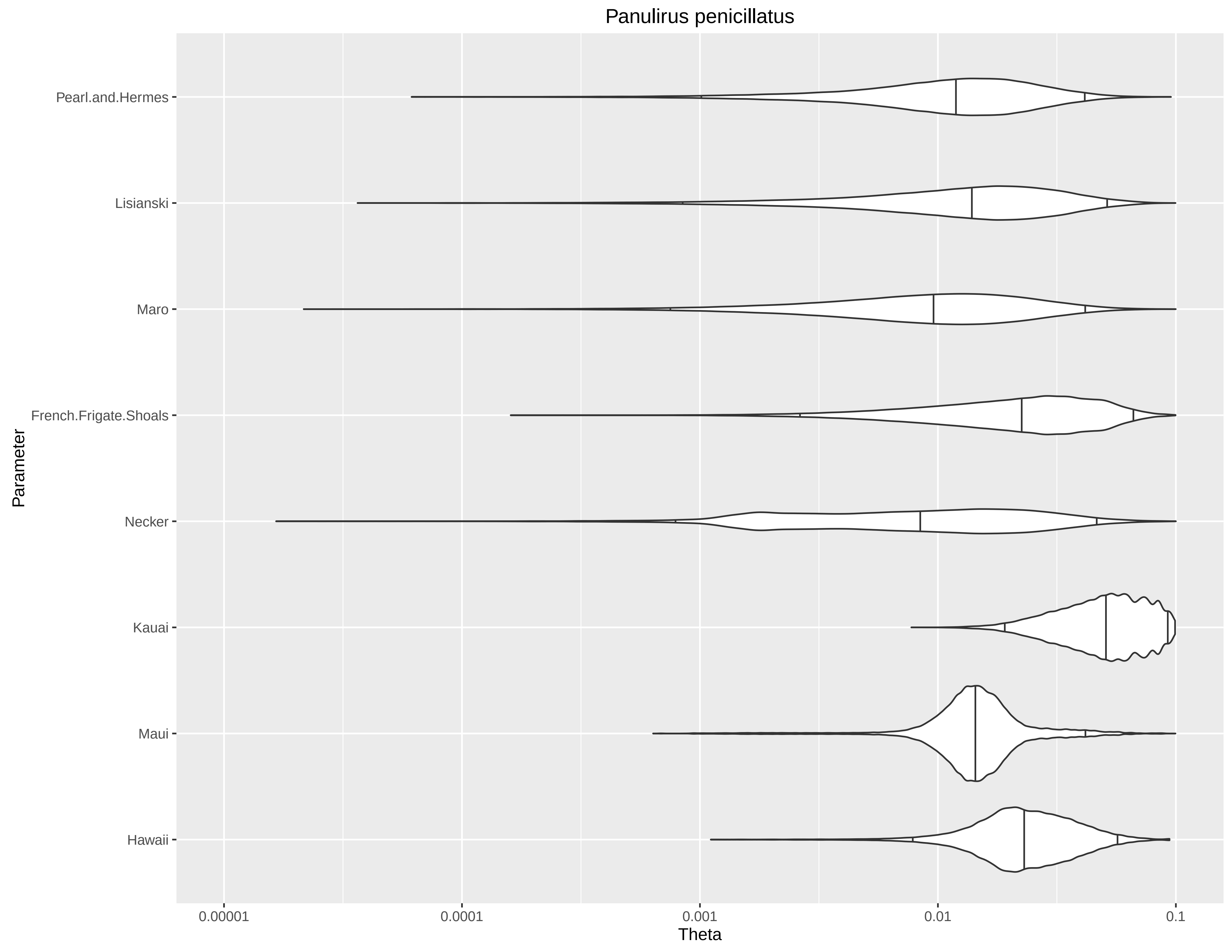
Panulirus penicillatus
Pearl.and.Hermes
Lisianski
Maro
French.Frigate.Shoals
Necker
Kauai
Maui
Hawaii
Parameter
0.00001
0.0001
0.001
0.01
0.1
Theta

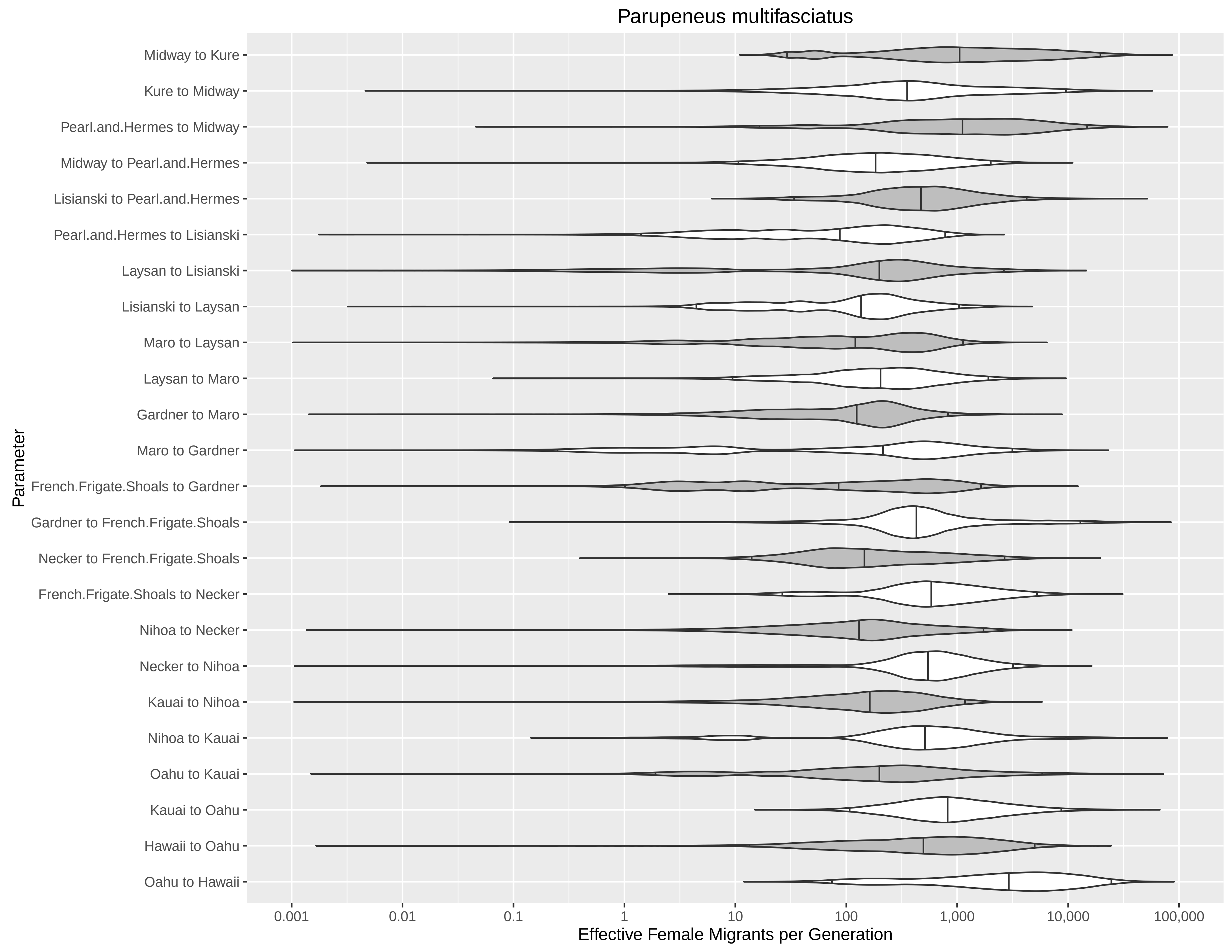

Parupeneus multifasciatus
Midway to Kure
Kure to Midway
Pearl.and.Hermes to Midway
Midway to Pearl.and.Hermes
Lisianski to Pearl.and.Hermes
Pearl.and.Hermes to Lisianski
Laysan to Lisianski
Lisianski to Laysan
Maro to Laysan
Laysan to Maro
Gardner to Maro
Maro to Gardner
French.Frigate.Shoals to Gardner
Gardner to French.Frigate.Shoals
Necker to French.Frigate.Shoals
French.Frigate.Shoals to Necker
Nihoa to Necker
Necker to Nihoa
Kauai to Nihoa
Nihoa to Kauai
Oahu to Kauai
Kauai to Oahu
Hawaii to Oahu
Oahu to Hawaii
Parameter
0.001
0.01
0.1
1
10
100
1,000
10,000
100,000
Effective Female Migrants per Generation

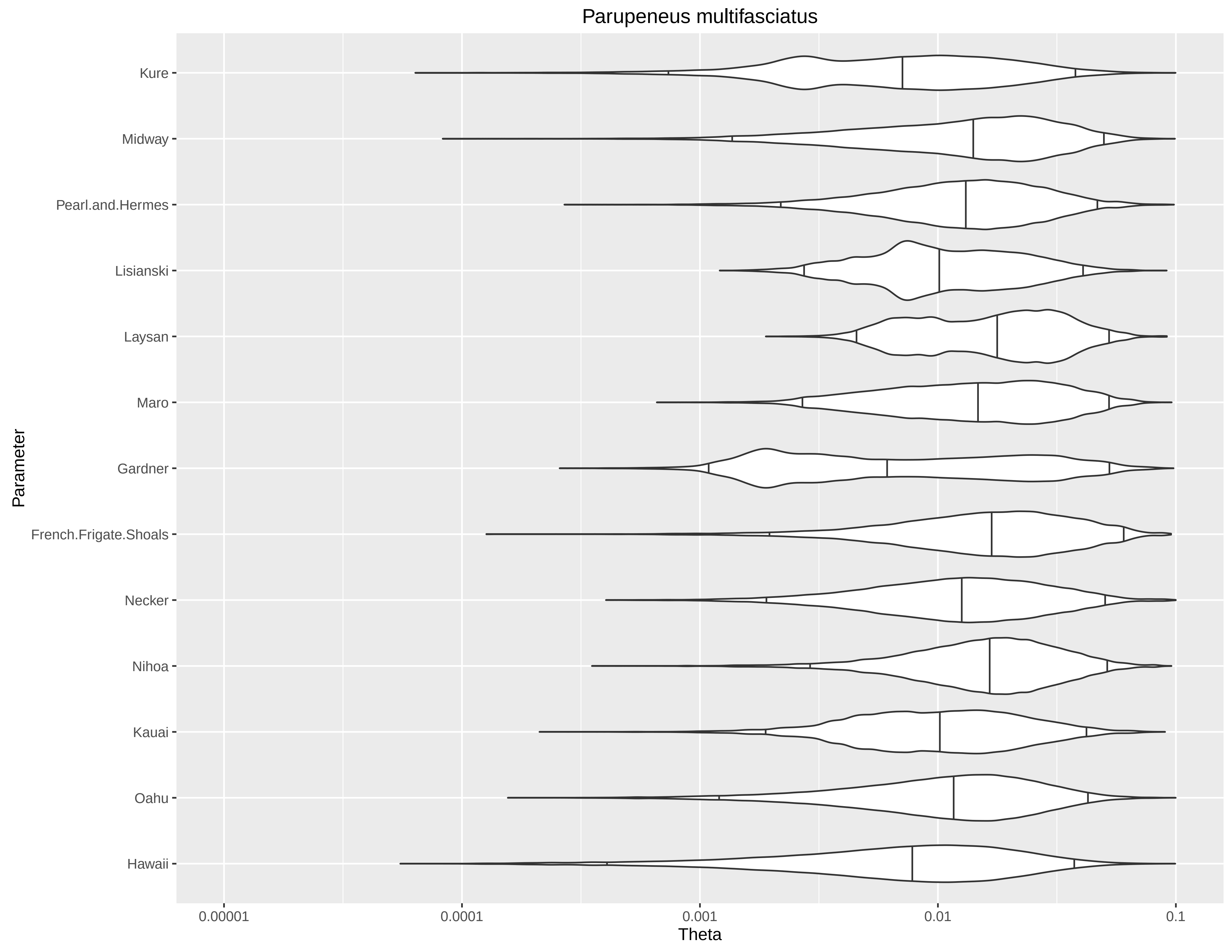

Parupeneus multifasciatus
Kure
Midway
Pearl.and.Hermes
Lisianski
Laysan
Maro
Gardner
French.Frigate.Shoals
Necker
Nihoa
Kauai
Oahu
Hawaii
Parameter
0.00001
0.0001
0.001
0.01
0.1
Theta

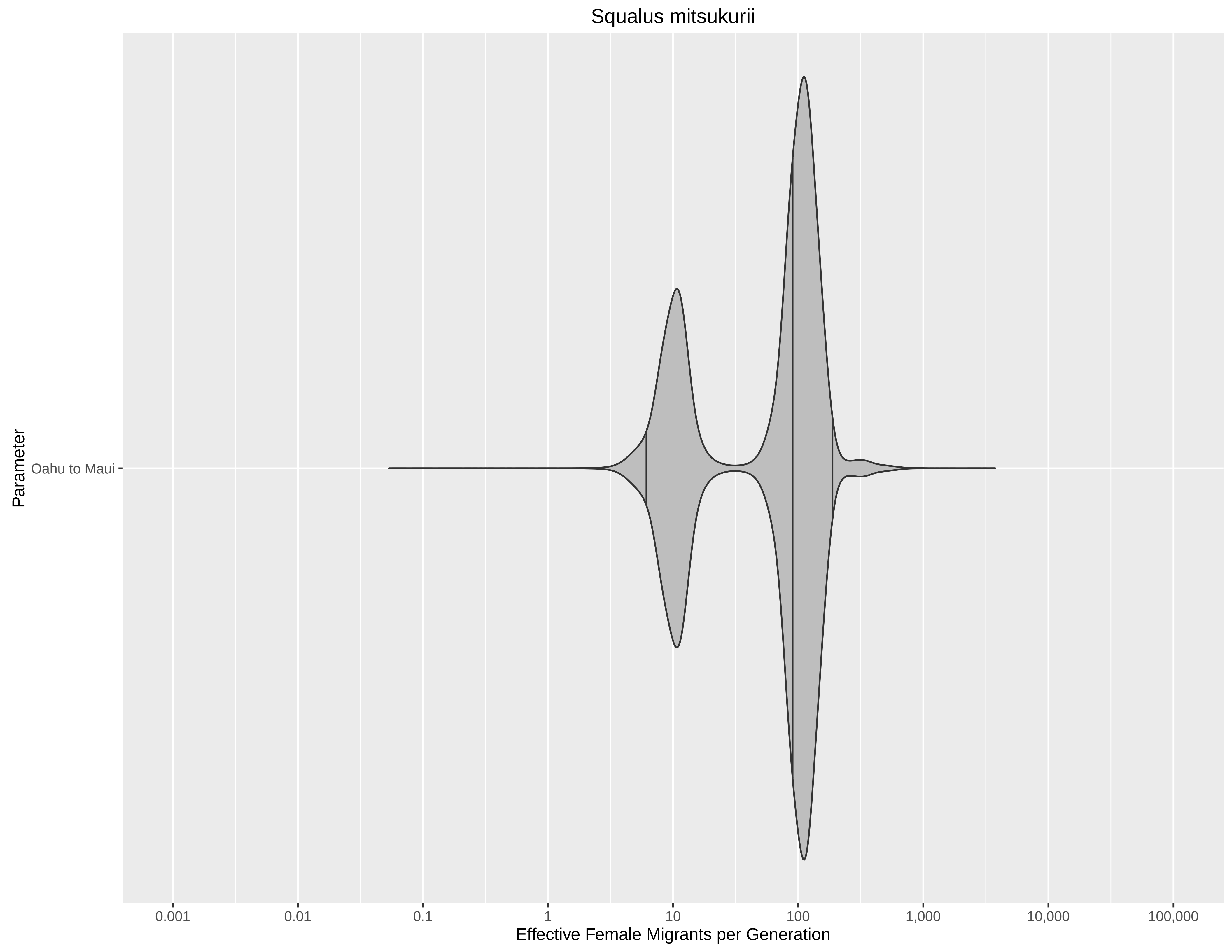

Squalus mitsukurii
Parameter
Oahu to Maui
0.001
0.01
0.1
1
10
100
1,000
10,000
100,000
Effective Female Migrants per Generation

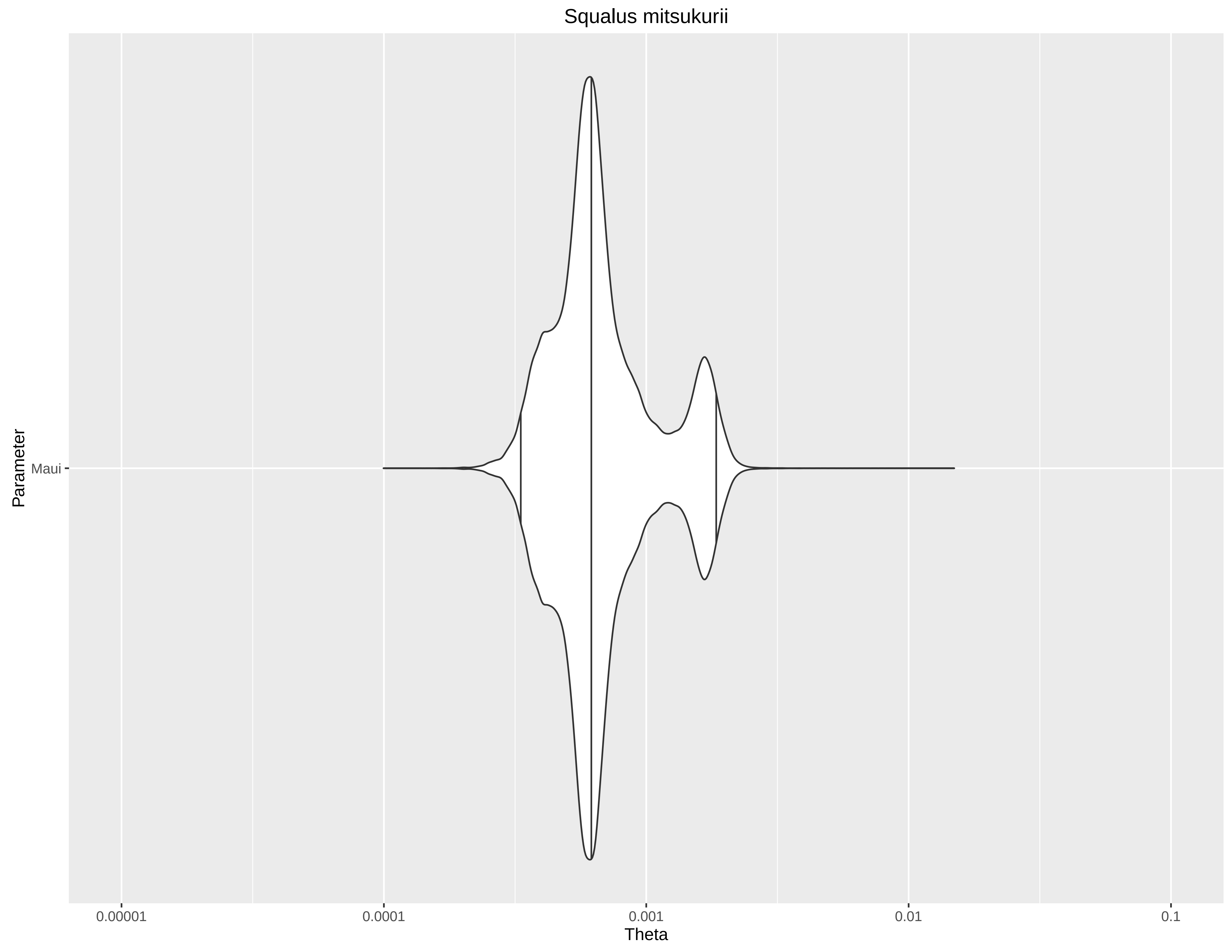
Squalus mitsukurii
Parameter
Maui
0.00001
0.0001
0.001
0.01
0.1
Theta

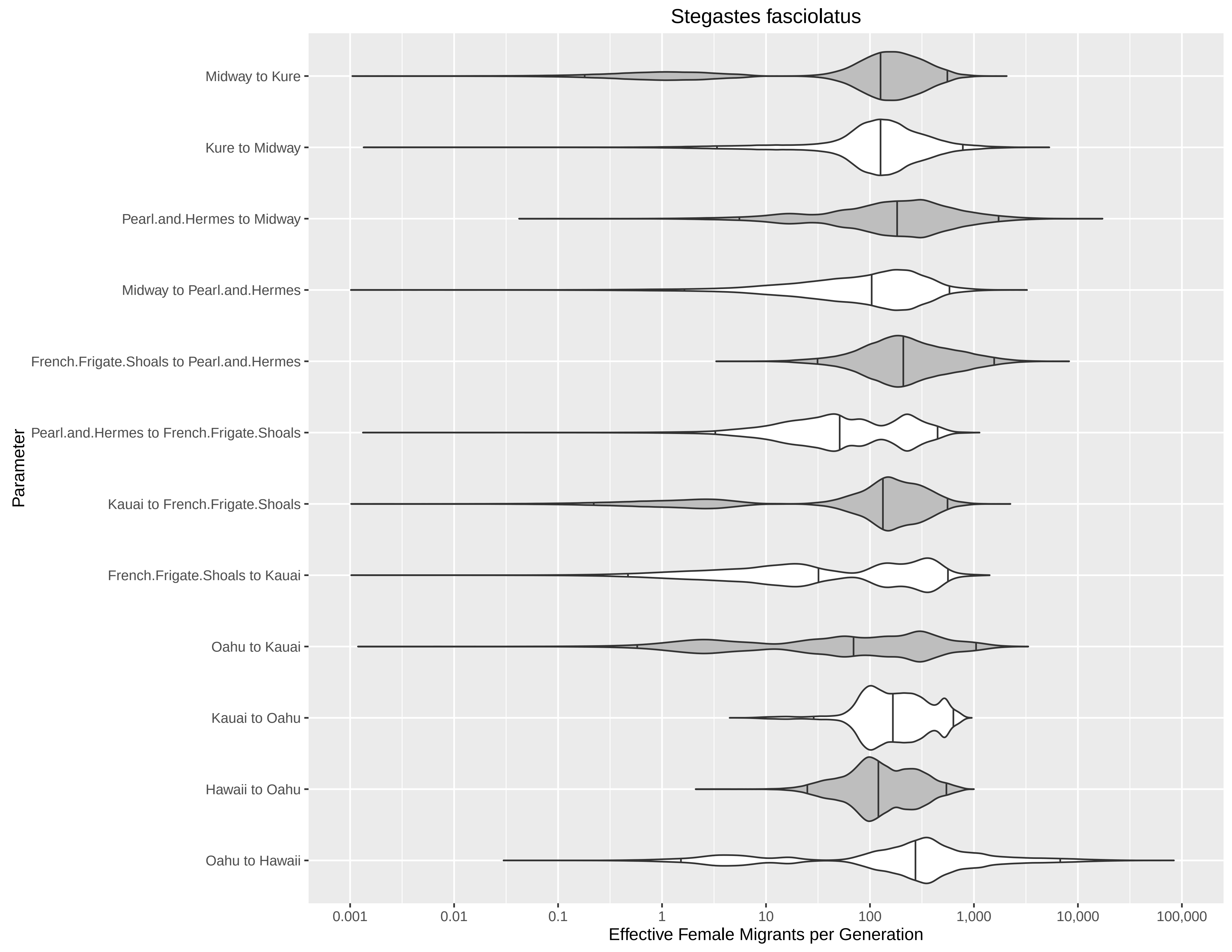
Stegastes fasciolatus
Parameter
Midway to Kure
Kure to Midway
Pearl.and.Hermes to Midway
Midway to Pearl.and.Hermes
French.Frigate.Shoals to Pearl.and.Hermes
Pearl.and.Hermes to French.Frigate.Shoals
Kauai to French.Frigate.Shoals
French.Frigate.Shoals to Kauai
Oahu to Kauai
Kauai to Oahu
Hawaii to Oahu
Oahu to Hawaii
0.001
0.01
0.1
1
10
100
1,000
10,000
100,000
Effective Female Migrants per Generation

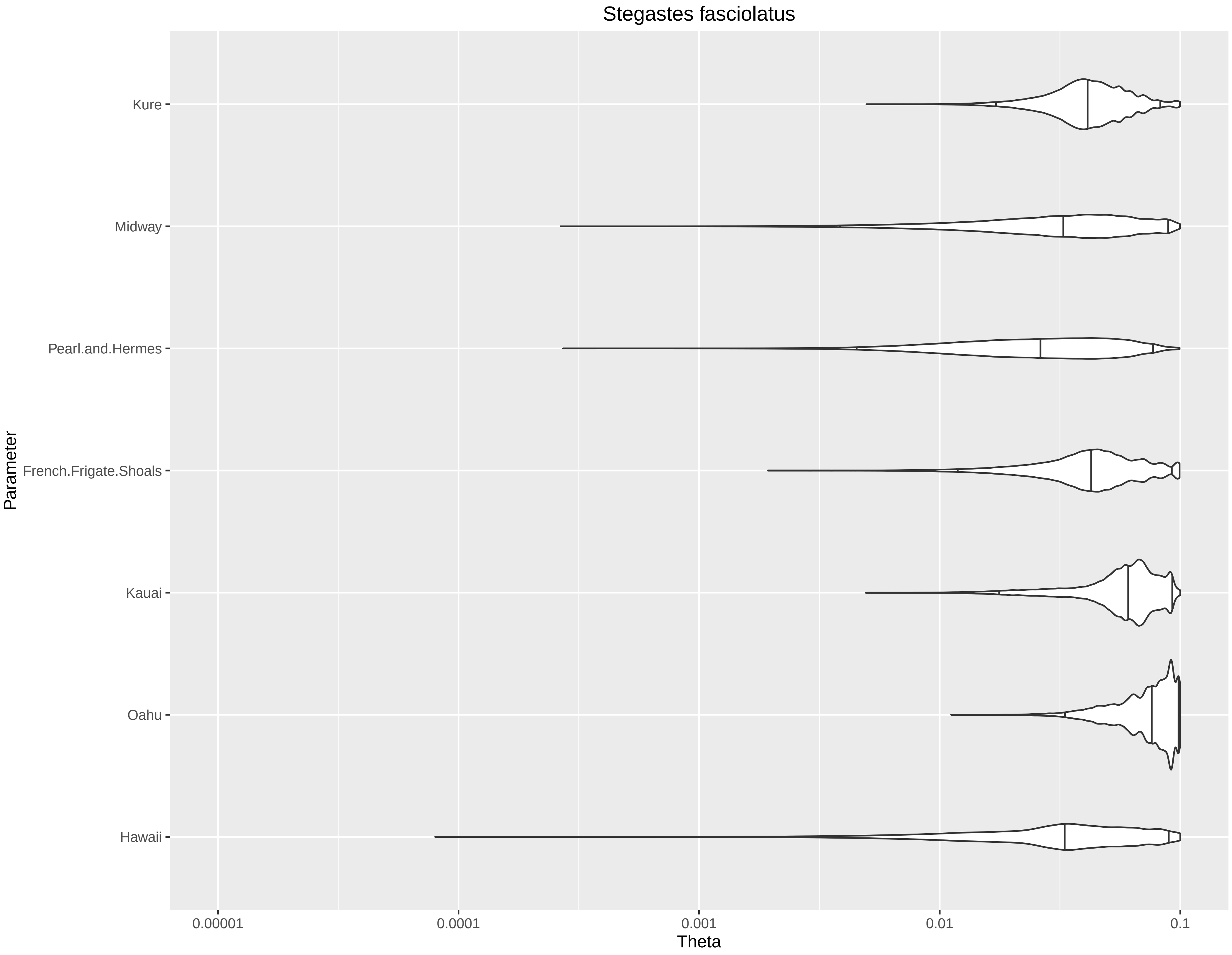

Stegastes fasciolatus
Kure
Midway
Pearl.and.Hermes
French.Frigate.Shoals
Kauai
Oahu
Hawaii
Parameter
0.00001
0.0001
0.001
0.01
0.1
Theta

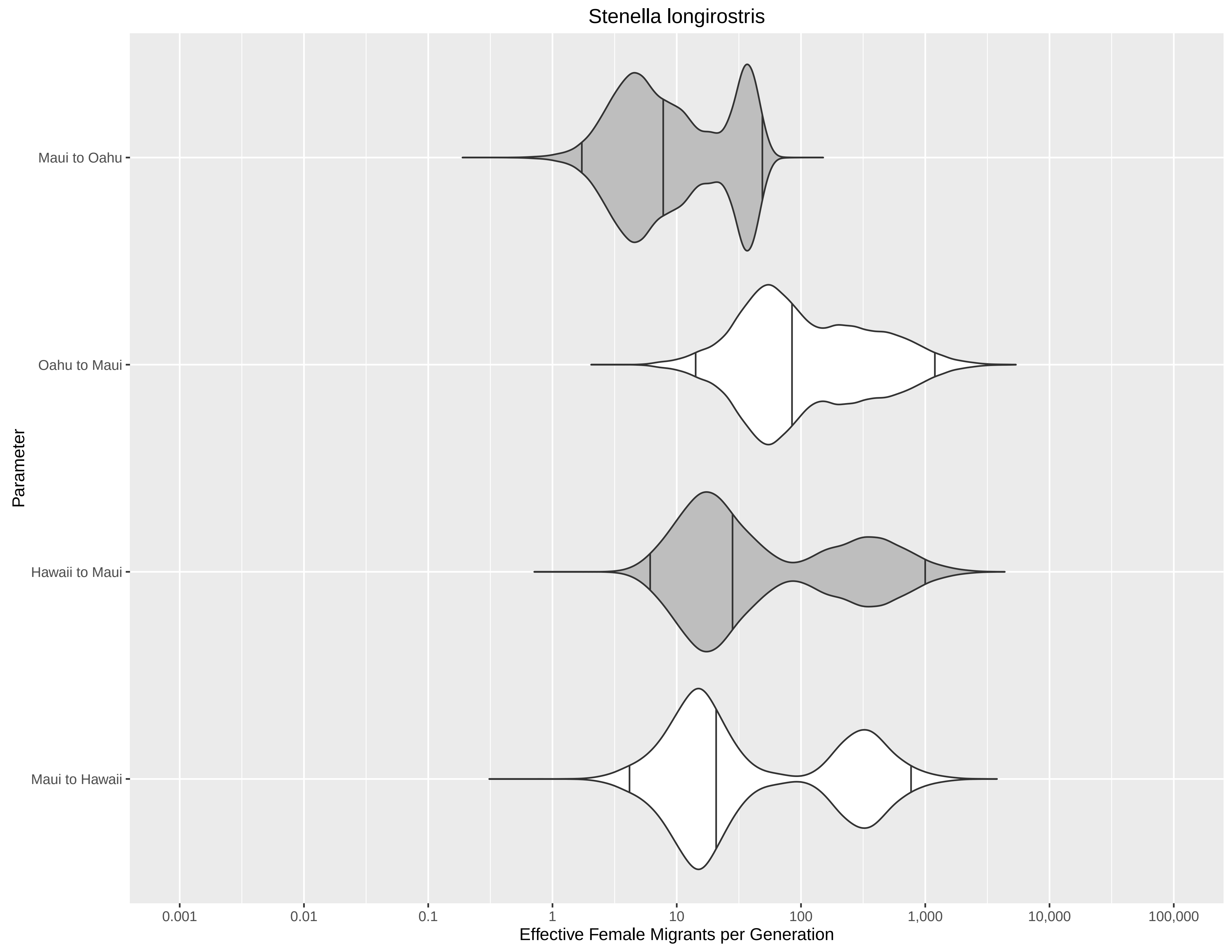

Stenella longirostris
Parameter
Maui to Oahu
Oahu to Maui
Hawaii to Maui
Maui to Hawaii
0.001
0.01
0.1
1
10
100
1,000
10,000
100,000
Effective Female Migrants per Generation

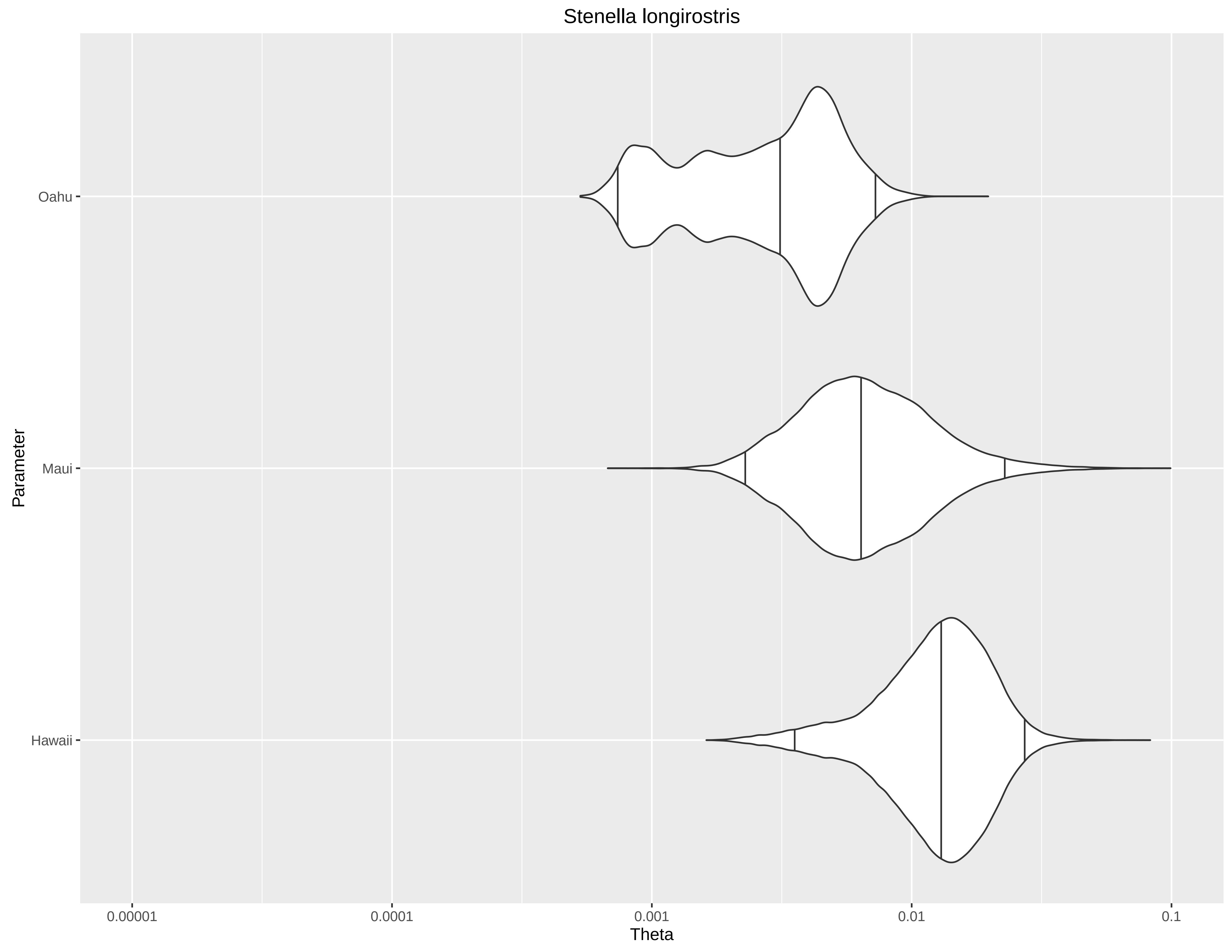

Stenella longirostris
Oahu
Maui
Hawaii
Parameter
0.00001
0.0001
0.001
0.01
0.1
Theta

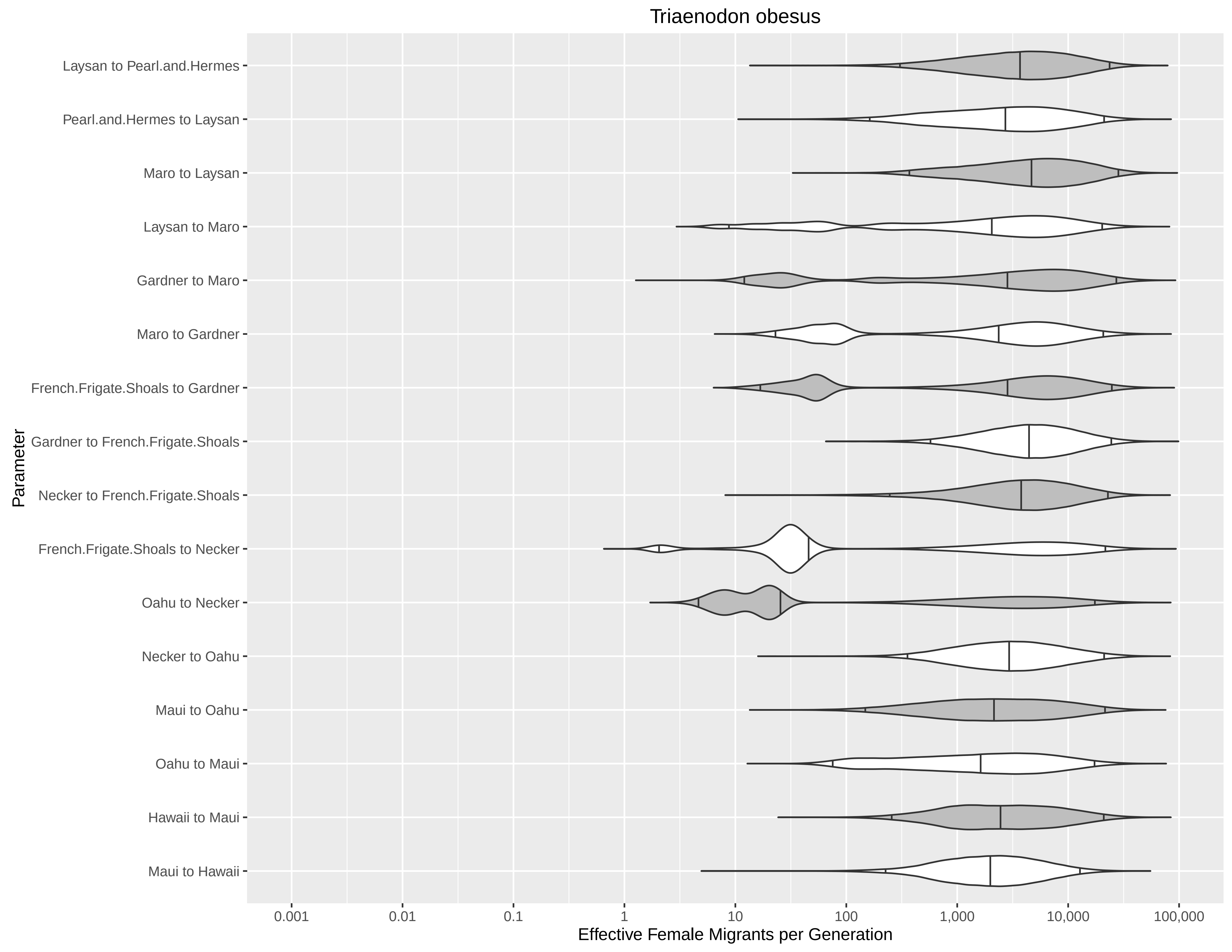

Triaenodon obesus
Parameter
Laysan to Pearl.and.Hermes
Pearl.and.Hermes to Laysan
Maro to Laysan
Laysan to Maro
Gardner to Maro
Maro to Gardner
French.Frigate.Shoals to Gardner
Gardner to French.Frigate.Shoals
Necker to French.Frigate.Shoals
French.Frigate.Shoals to Necker
Oahu to Necker
Necker to Oahu
Maui to Oahu
Oahu to Maui
Hawaii to Maui
Maui to Hawaii
0.001
0.01
0.1
1
10
100
1,000
10,000
100,000
Effective Female Migrants per Generation

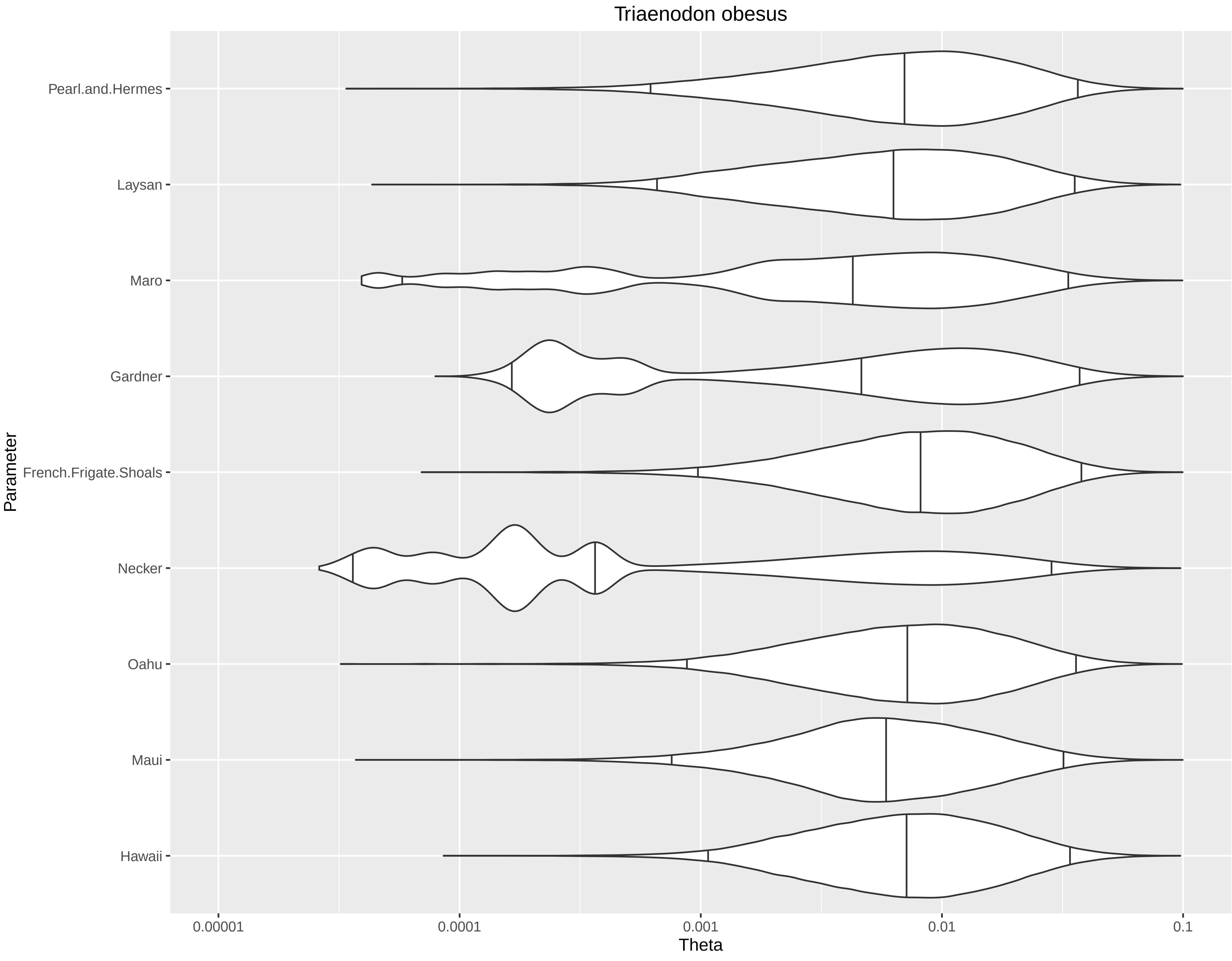

Triaenodon obesus
Pearl.and.Hermes
Laysan
Maro
Gardner
French.Frigate.Shoals
Necker
Oahu
Maui
Hawaii
Parameter
0.00001
0.0001
0.001
0.01
0.1
Theta

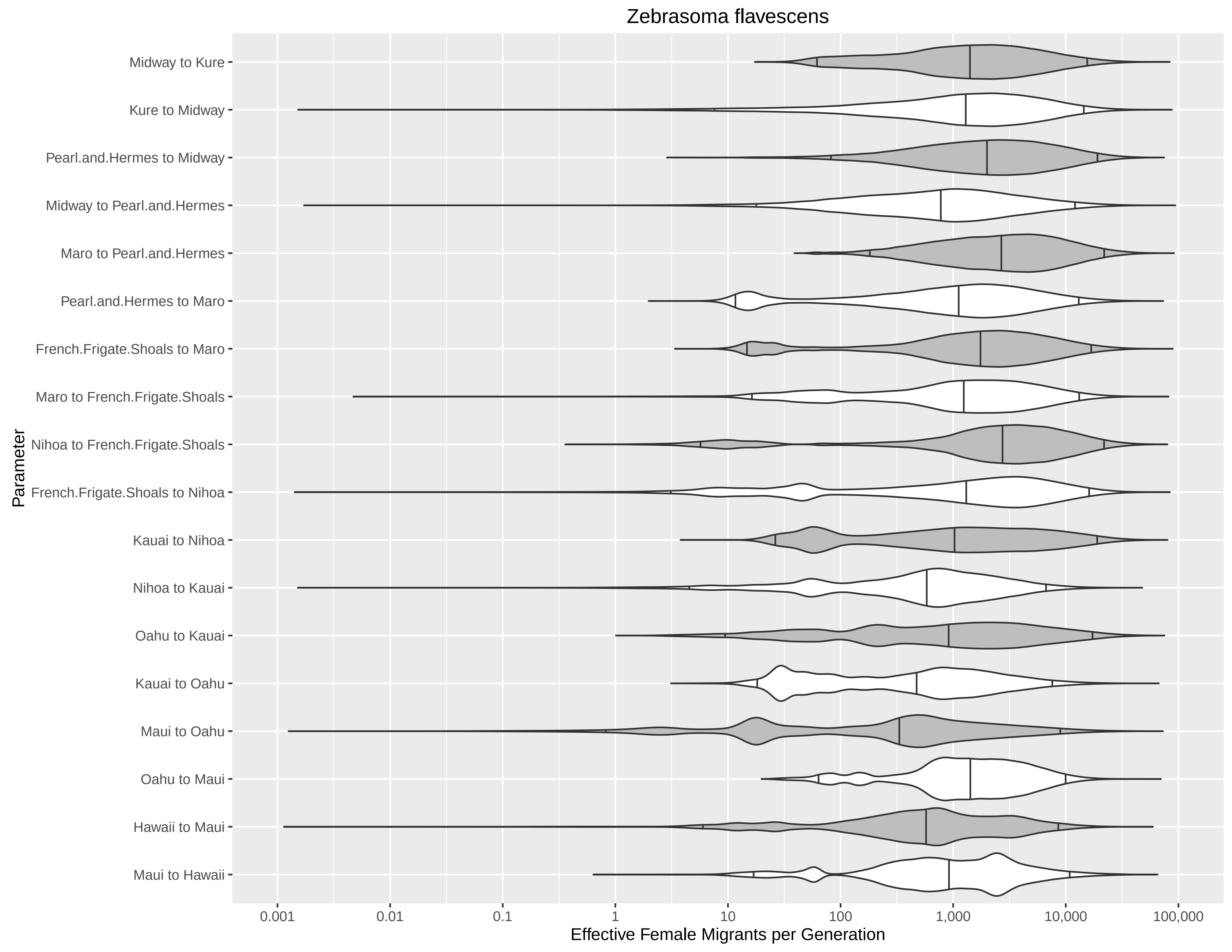
Zebrasoma flavescens
Parameter
Midway to Kure
Kure to Midway
Pearl.and.Hermes to Midway
Midway to Pearl.and.Hermes
Maro to Pearl.and.Hermes
Pearl.and.Hermes to Maro
French.Frigate.Shoals to Maro
Maro to French.Frigate.Shoals
Nihoa to French.Frigate.Shoals
French.Frigate.Shoals to Nihoa
Kauai to Nihoa
Nihoa to Kauai
Oahu to Kauai
Kauai to Oahu
Maui to Oahu
Oahu to Maui
Hawaii to Maui
Maui to Hawaii
0.001
0.01
0.1
1
10
100
1,000
10,000
100,000
Effective Female Migrants per Generation

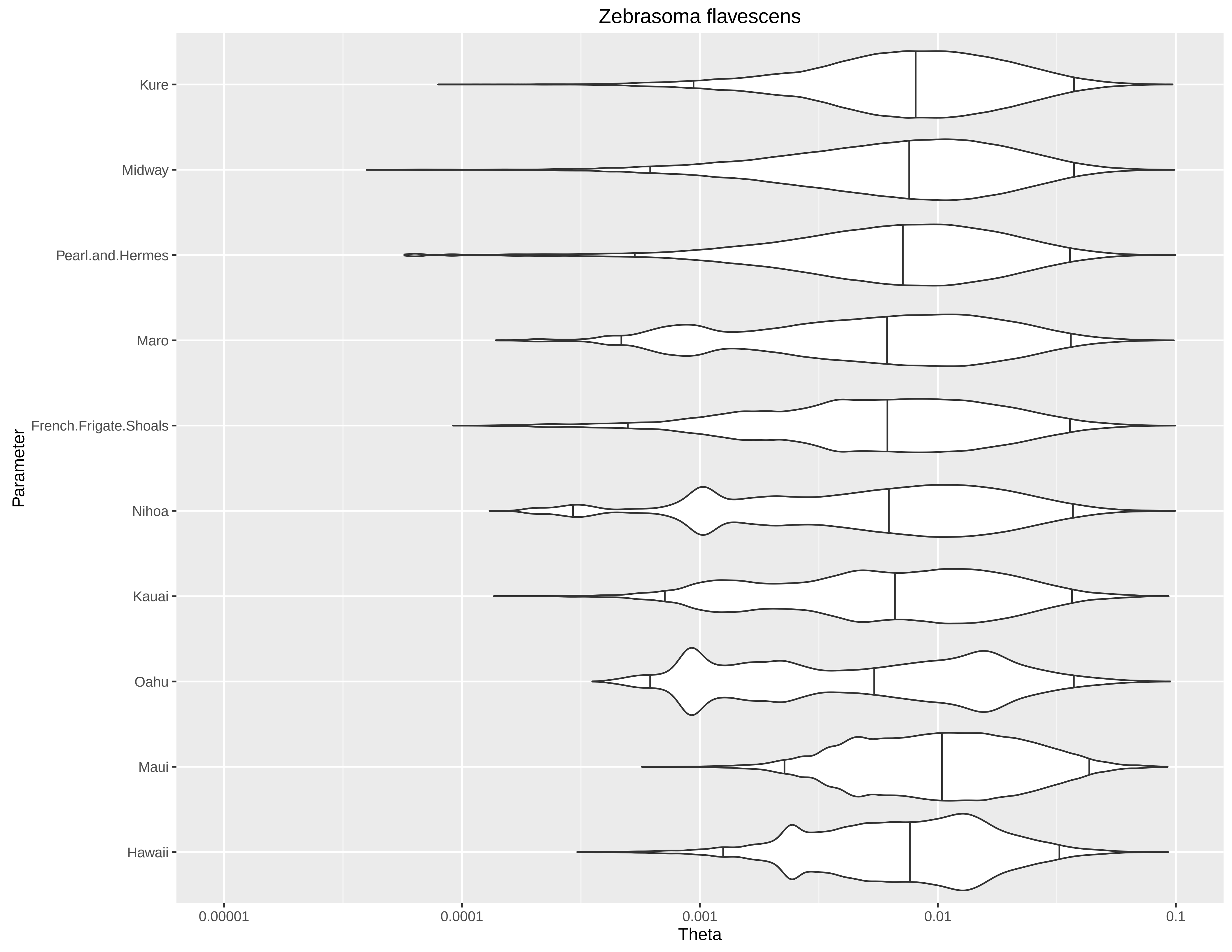

Zebrasoma flavescens
Kure
Midway
Pearl.and.Hermes
Maro
French.Frigate.Shoals
Nihoa
Kauai
Oahu
Maui
Hawaii
Parameter
0.00001
0.0001
0.001
0.01
0.1
Theta
