# Supplementary material for: A coalescent sampler successfully detects biologically meaningful population structure overlooked by F‐statistics
Source: Evol Appl. 2018 Oct 15;12(2):255–65. doi: 10.1111/eva.12712 (PMC6346657; doi:10.1111/eva.12712)

Slopes for Theta ~ Area for: Abudefduf vaigiensis p = 0.52

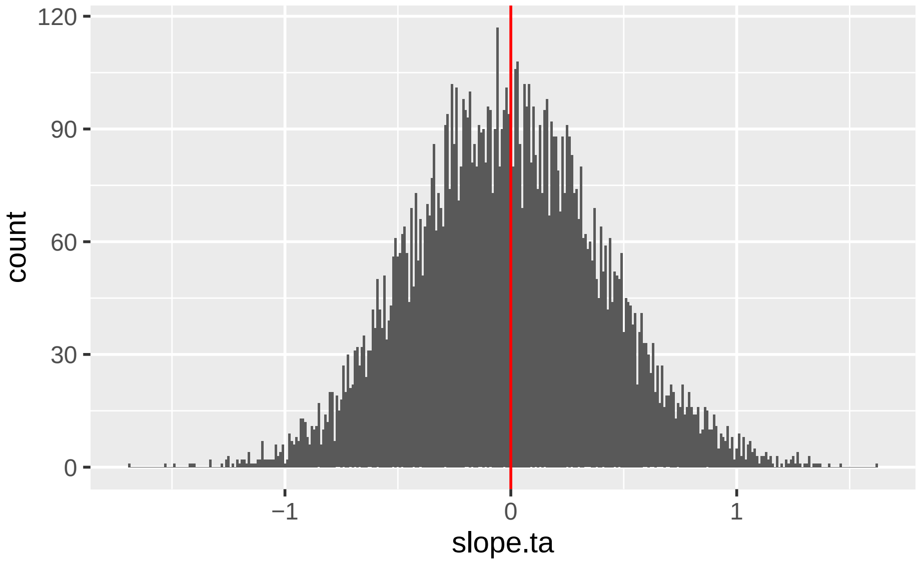

Slopes for Theta ~ Area for: Acanthurus nigroris p = 0.465

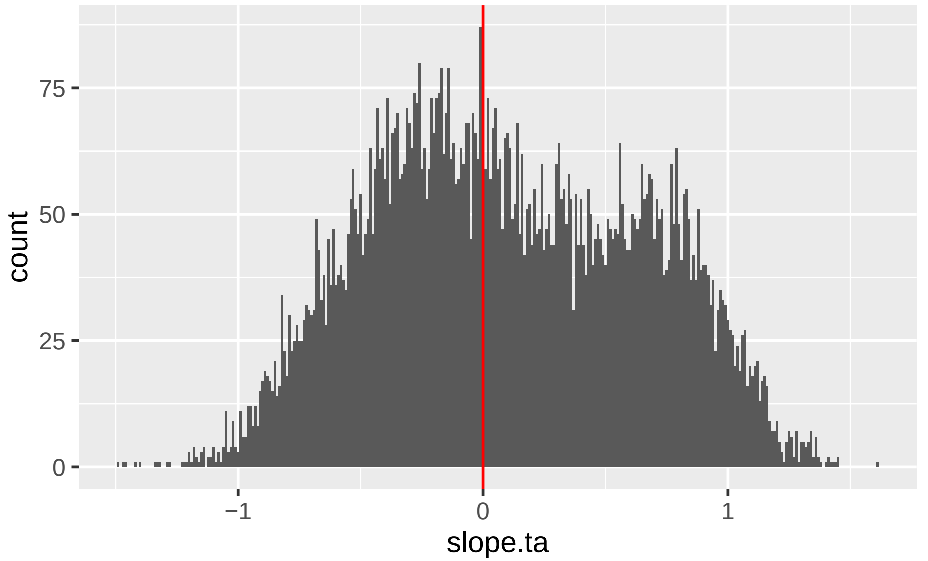

Slopes for Theta ~ Area for: Acanthurus olivaceus p = 0.45

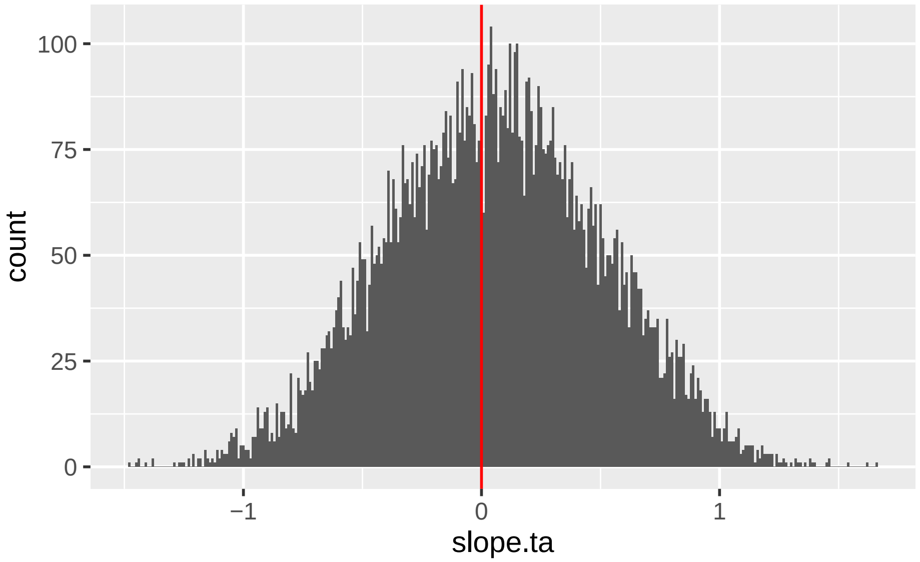

Slopes for Theta ~ Area for: Calcinus hazletti p = 0.2005

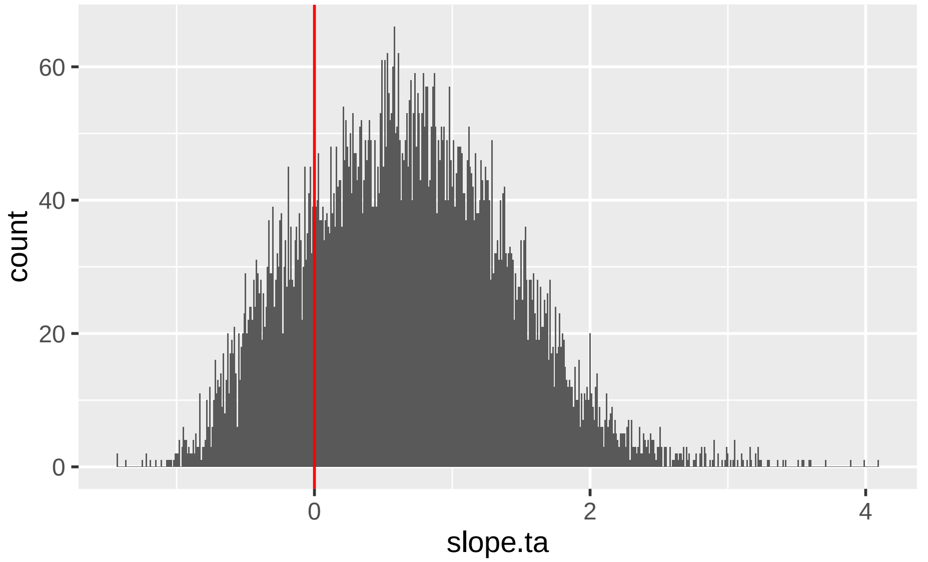

Slopes for Theta ~ Area for: Halichoeres ornatissimus p = 0.1

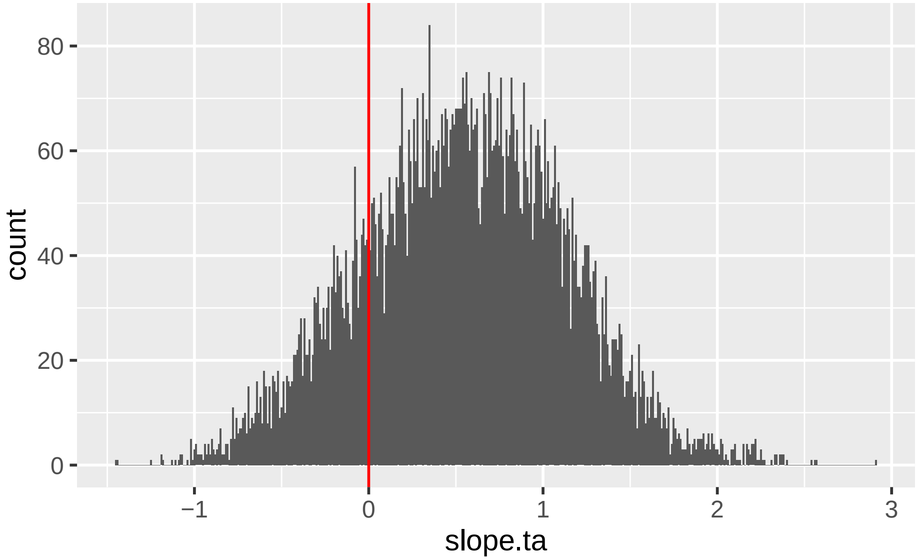

Slopes for Theta ~ Area for: Caranx melampygus p = 0.359

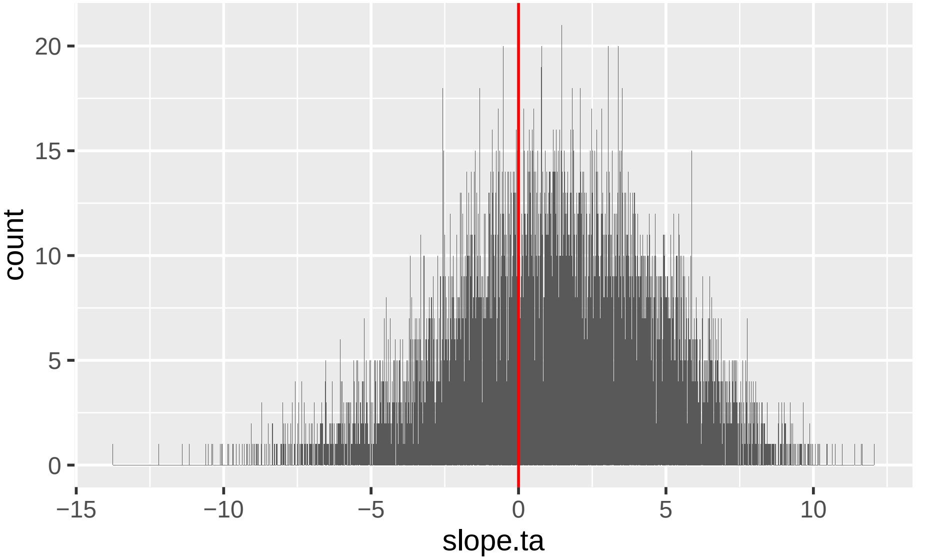

Slopes for Theta ~ Area for: Cellana exarata p = 0

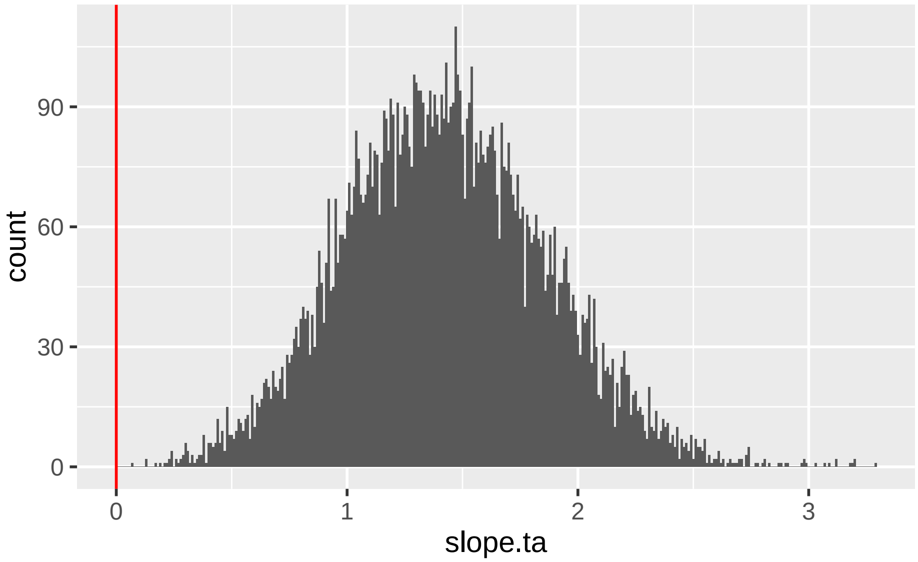

Slopes for Theta ~ Area for: Cellana talcosa p = 0.2465

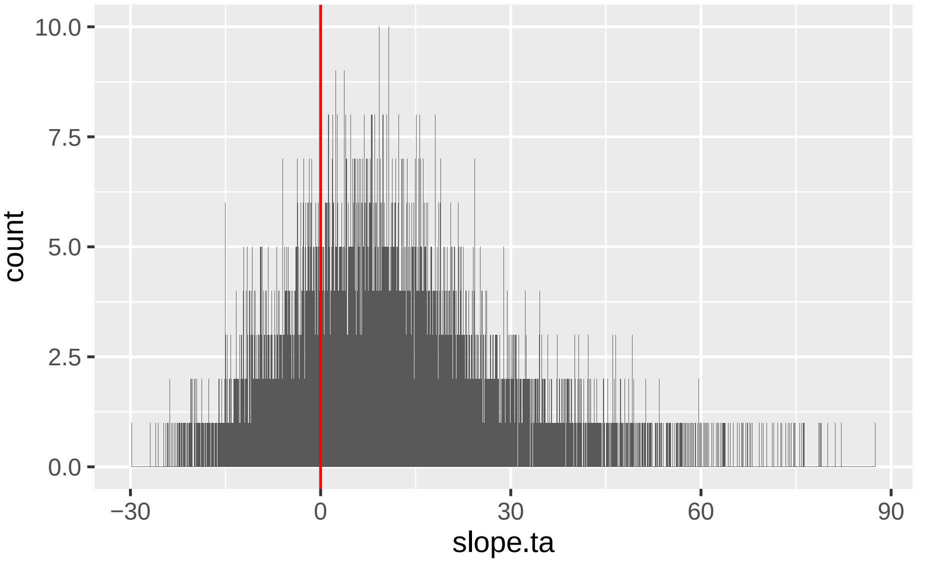

Slopes for Theta ~ Area for: Chaetodon lunulatus p = 0.22[illegible]

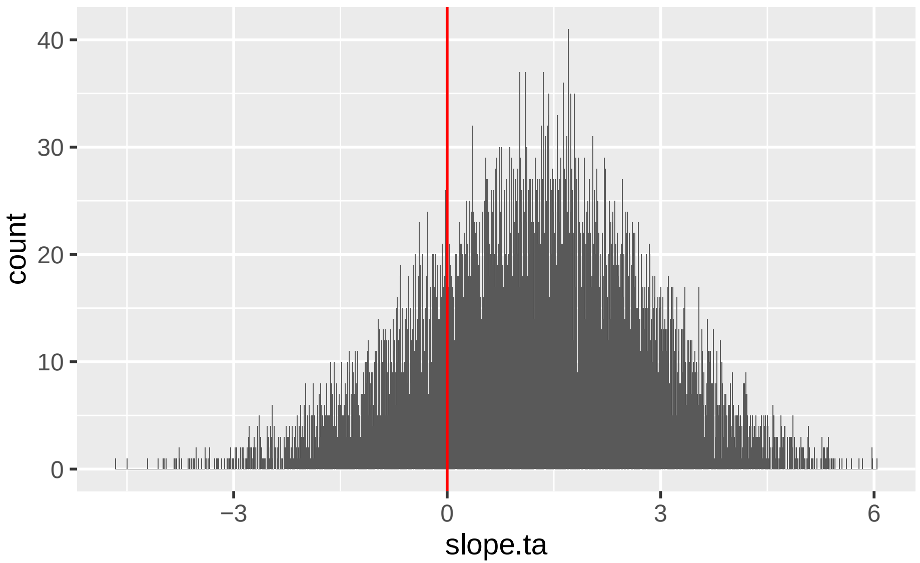

Slopes for Theta ~ Area for: Chaetodon miliaris p = 0.371

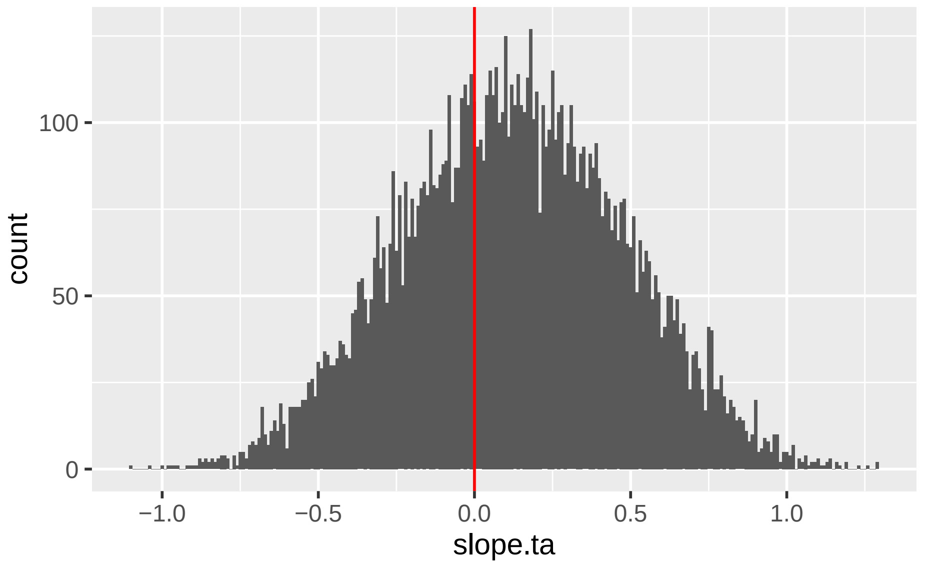

Slopes for Theta ~ Area for: Chaetodon multicinctus p = 0.4[illegible]

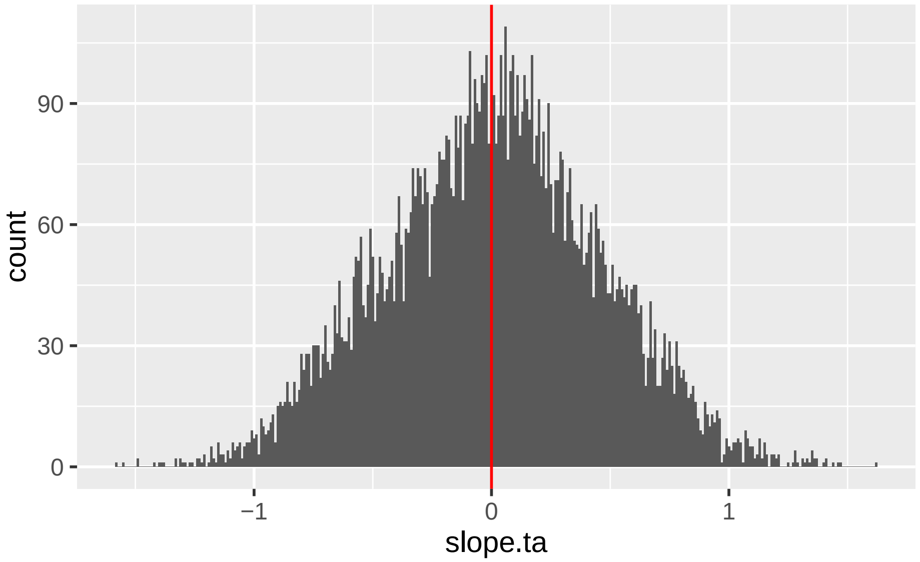

Slopes for Theta ~ Area for: Heterocentrotus mammillatus p = [illegible]

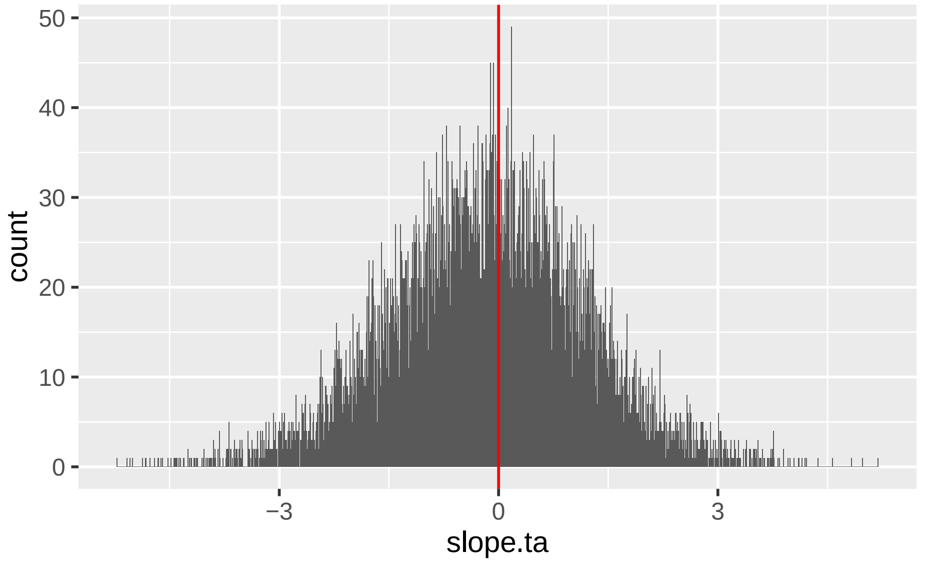

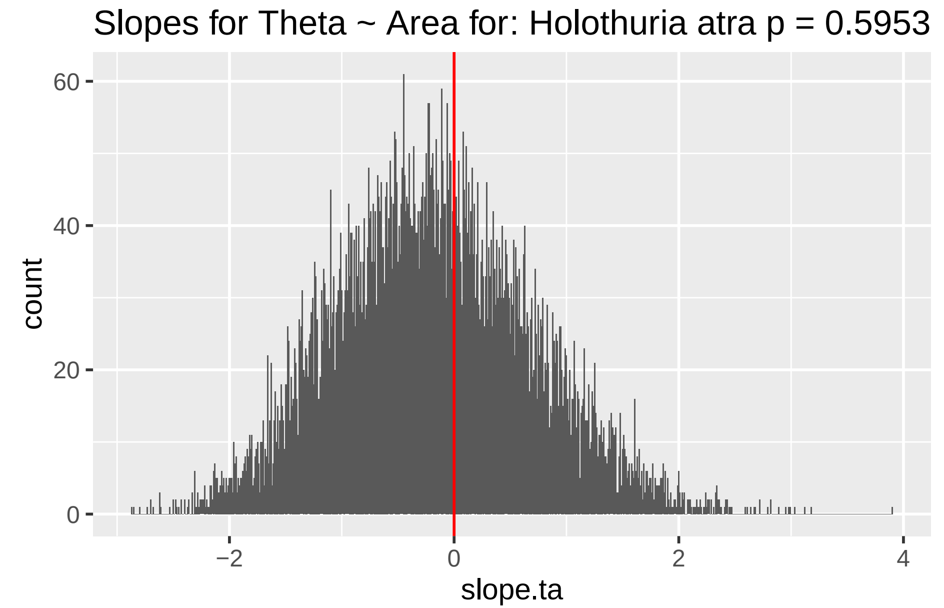
Slopes for Theta ~ Area for: Holothuria atra p = 0.5953
count
0
20
40
60
−2
0
2
4
slope.ta

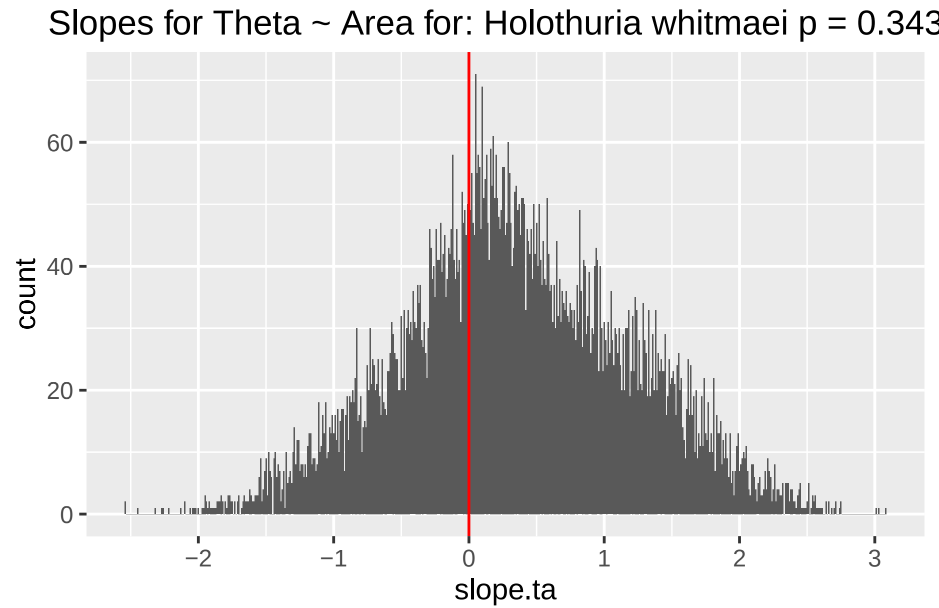
Slopes for Theta ~ Area for: Holothuria whitmaei p = 0.343
count
0
20
40
60
−2
−1
0
1
2
3
slope.ta

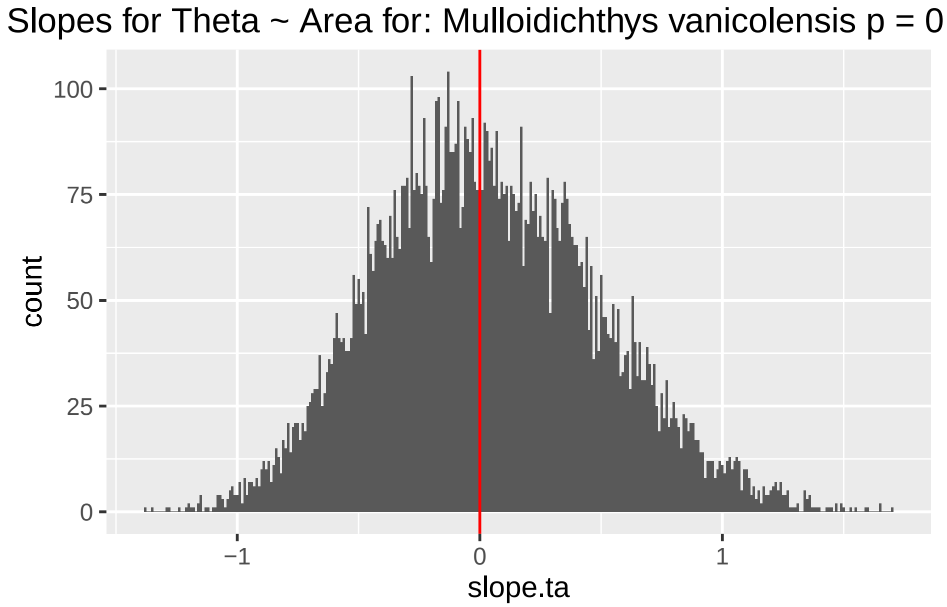
Slopes for Theta ~ Area for: Mulloidichthys vanicolensis p = 0
count
0
25
50
75
100
−1
0
1
slope.ta

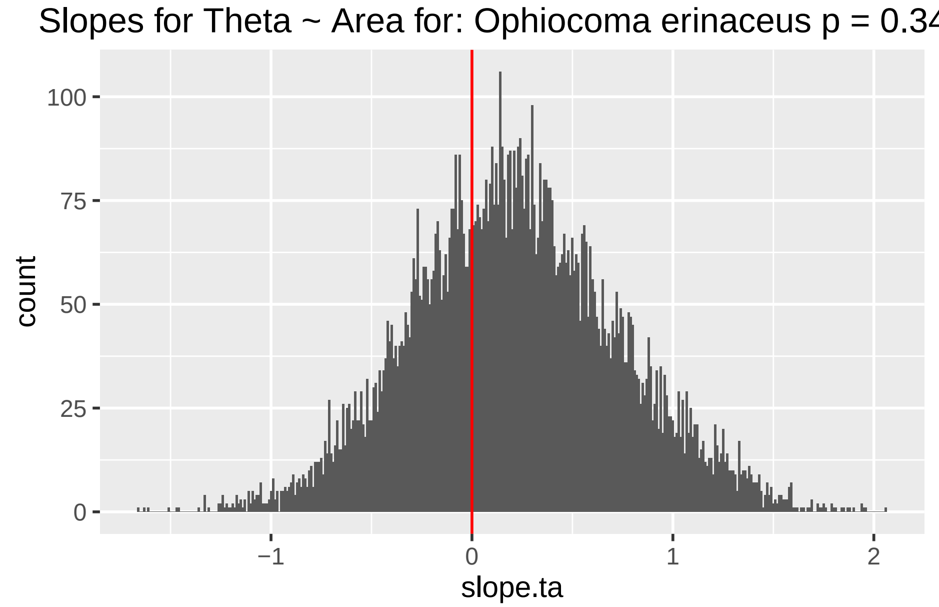
Slopes for Theta ~ Area for: Ophiocoma erinaceus p = 0.34
count
0
25
50
75
100
−1
0
1
2
slope.ta

Slopes for Theta ~ Area for: Ophiocoma pica p = 0.2737

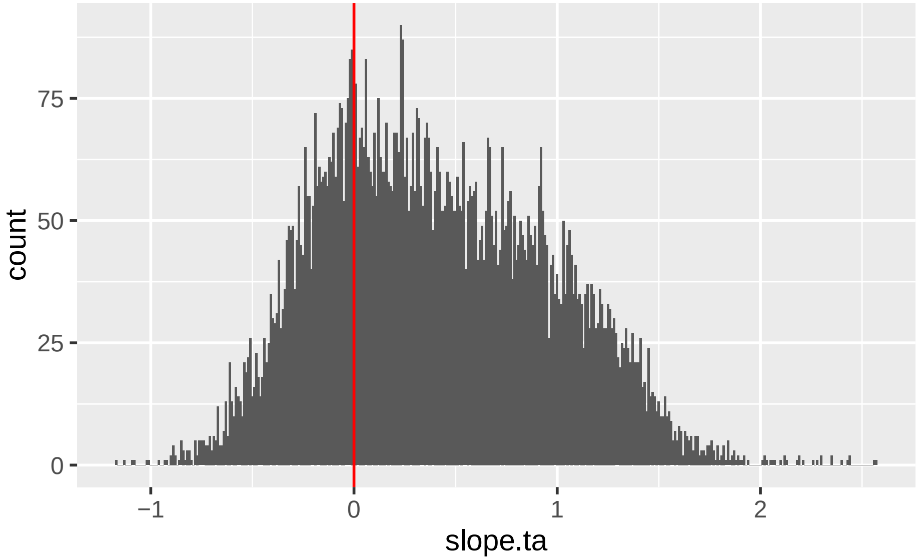

Slopes for Theta ~ Area for: Panulirus penicillatus p = 0.34

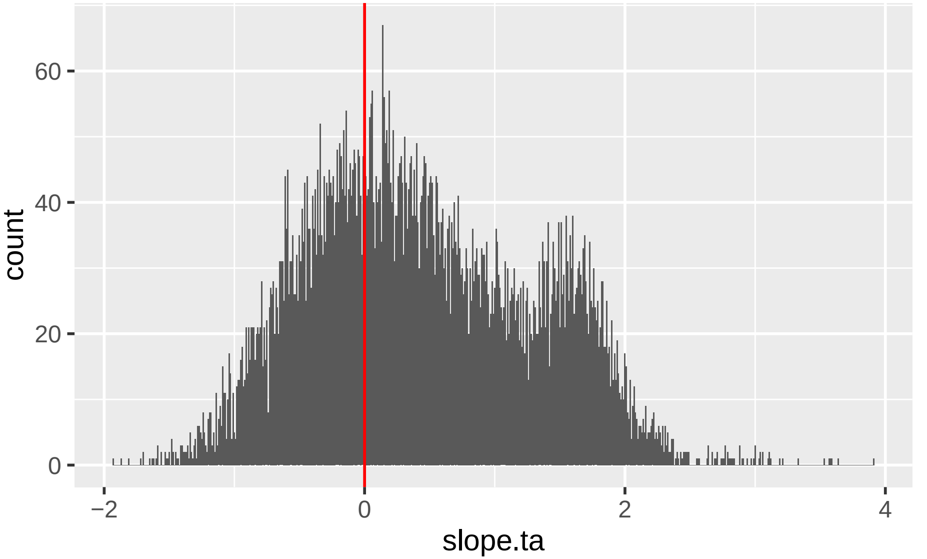

Slopes for Theta ~ Area for: Parupeneus multifasciatus p = 0.

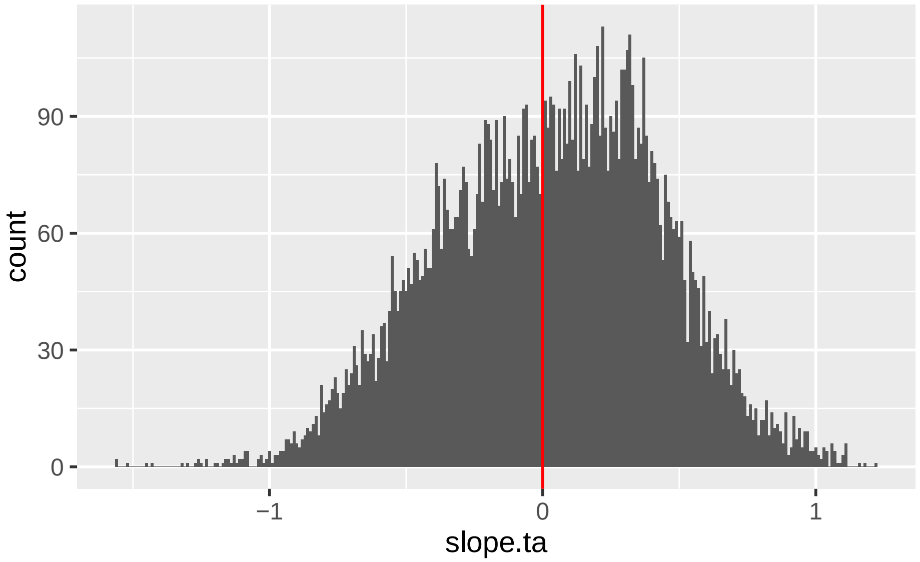

Slopes for Theta ~ Area for: Stegastes fasciolatus p = 0.38

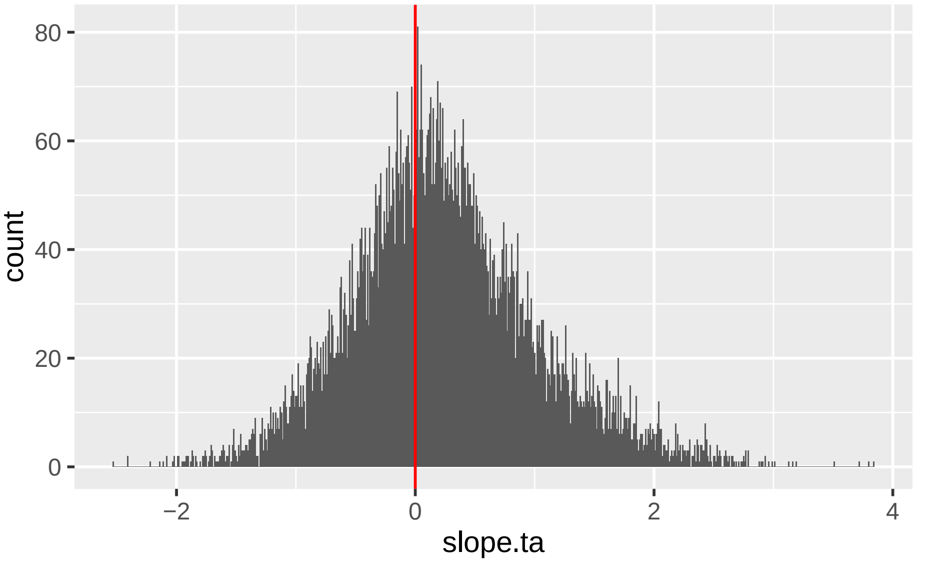

Slopes for Theta ~ Area for: Triaenodon obesus p = 0.196

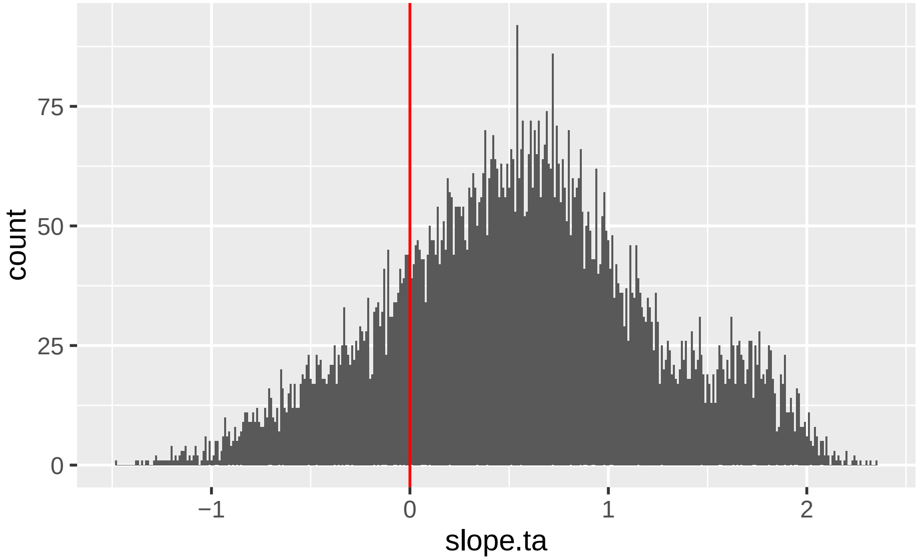

Slopes for Theta ~ Area for: Zebrasoma flavescens p = 0.53

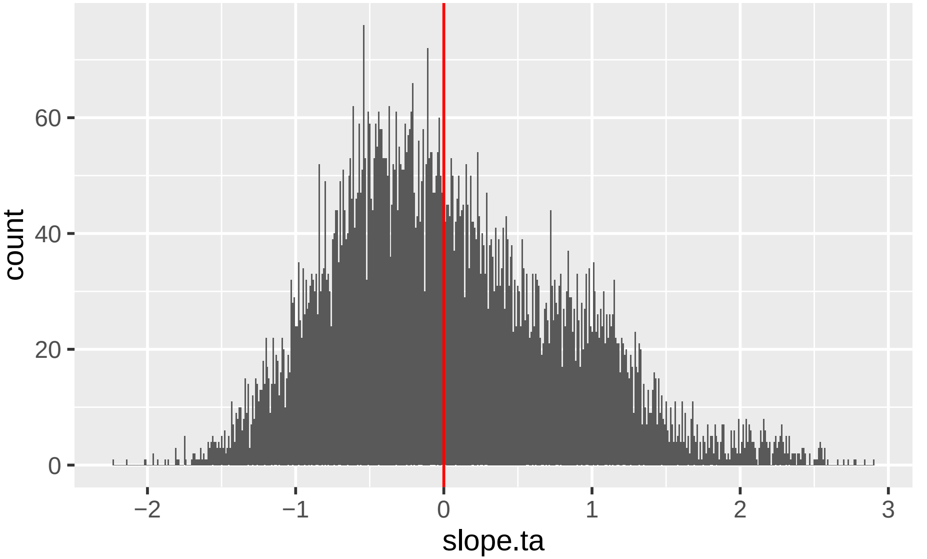

Supplement: Supplementary file 4 [file EVA-12-255-s004.pdf]
