# Supplementary material for: A coalescent sampler successfully detects biologically meaningful population structure overlooked by F‐statistics
Source: Evol Appl. 2018 Oct 15;12(2):255–65. doi: 10.1111/eva.12712 (PMC6346657; doi:10.1111/eva.12712)
Supplement: Supplementary file 5 [file EVA-12-255-s005.docx]

Supplemental Table S1: Summary Statistics for mitochondrial datasets from 41 marine species sampled along the Hawaiian archipelago. n = total number of sequenced individuals; BP = base pairs of sequence; PLD = pelagic larval duration; t-test p-value = -p-value for permutation t-test between replicated marginal likelihood values of the first and second best models for each species.

| Species | Common Name | Locus | n | BP | PLD | h | Φ_ST_ | Φ_ST_  p-value | Selkoe 2014 Inference | Migrate  Inference | t-test  P-value | # Islands Sampled | Selkoe # of Regions | Migrate # of Regions |
| --- | --- | --- | --- | --- | --- | --- | --- | --- | --- | --- | --- | --- | --- | --- |
| *Abudefduf abdominalis* | Sergeant Major | CytB | 315 | 574 | 24 | 0.68 | 0.003 | 0.026 | Regional: High-Low | Regional: High-Low | 0.047 | 12 | 2 | 2 |
| *Abudefduf vaigiensis* | Sergeant Major | CytB | 209 | 574 | 20 | 0.68 | 0.001 | 0.125 | Panmixia | Ambiguous | 0.197 | 12 | 1 | NA |
| *Acanthurus nigrofuscus* | Brown Tang | CytB | 280 | 692 | 31 | 0.71 | 0.000 | 0.353 | Stepping-Stone | Ambiguous | 0.386 | 8 | 8 | NA |
| *Acanthurus nigroris* | Blue-lined Surgeonfish | CytB | 445 | 797 | 58 | 0.65 | 0.000 | 0.288 | Regional: 1 Current | Ambiguous | 0.091 | 12 | 2 | NA |
| *Acanthurus olivaceus* | Orange Band Surgeonfish | CytB | 345 | 700 | 60 | 0.66 | -0.005 | 0.737 | Panmixia | Ambiguous | 0.095 | 14 | 1 | NA |
| *Acanthaster planci* | Crown-of-Thorns Seastar | CR | 476 | 548 | 14 | 0.81 | 0.013 | 0.001 | Stepping-Stone | Ambiguous | 0.387 | 6 | 6 | NA |
| *Calcinus hazletti* | Whitefoot Hermit Crab | CO1 | 172 | 582 | 50 | 0.66 | 0.000 | 0.847 | Regional: Other | Stepping-Stone | 0.047 | 10 | 2 | 10 |
| *Calcinus seurati* | Zebra Hermit Crab | CO1 | 158 | 573 | 50 | 0.99 | 0.000 | 0.263 | Panmixia | Panmixia | 0.047 | 4 | 1 | 1 |
| *Caranx melampygus* | Bluefin Trevally | ATP-S | 57 | 659 | 57.6 | 0.71 | 0.000 | 0.654 | Chaos | Stepping-Stone | 0.047 | 4 | 2 | 4 |
| *Cellana exarata* | Black-foot Opihi Limpet | CO1 | 170 | 612 | 6 | 0.70 | 0.008 | 0.001 | Regional: Other | Stepping-Stone | 0.047 | 7 | 3 | 7 |
| *Cellana sandwicensis* | Yellow-foot Opihi Limpet | CO1 | 109 | 612 | 6 | 0.96 | 0.007 | 0.005 | Regional: High-Low | Ambiguous | 0.25 | 5 | 2 | NA |
| *Cellana talcosa* | Talc Opihi Limpet | CO1 | 105 | 629 | 6 | 0.76 | 0.011 | 0.001 | Stepping-Stone | Stepping-Stone | 0.047 | 3 | 3 | 3 |
| *Chaetodon fremblii* | Blue-striped Butterflyfish | CytB | 393 | 665 | 40 | 0.71 | 0.000 | 0.554 | Panmixia | Panmixia | 0.047 | 10 | 1 | 1 |
| *Chaetodon lunulatus* | Oval Butterflyfish | CytB | 225 | 605 | 40 | 0.59 | 0.318 | 0.001 | Regional: Other | Stepping-Stone | 0.047 | 9 | 5 | 9 |
| *Chaetodon miliaris* | Lemon Butterflyfish | CytB | 401 | 655 | 60 | 0.66 | 0.000 | 0.545 | Panmixia | Stepping-Stone | 0.047 | 14 | 1 | 14 |
| *Chaetodon multicinctus* | Pebbled Butterflyfish | CytB | 278 | 657 | 40 | 0.56 | 0.000 | 0.465 | Panmixia | Stepping-Stone | 0.047 | 11 | 1 | 11 |
| *Ctenochaetus strigosus* | Yellow-eyed Tang | CytB | 531 | 632 | 58 | 0.65 | 0.000 | 0.346 | Regional: Other | Ambiguous | 0.139 | 11 | 3 | NA |
| *Dascyllus albisella* | White-spotted Damsel | CR | 102 | 378 | 27 | 0.99 | 0.006 | 0.001 | Regional: Other | Ambiguous | 0.267 | 5 | 2 | NA |
| *Epinephelus quernus* | Hawaiian Grouper | CR | 301 | 398 | 40 | 0.73 | 0.000 | 0.214 | Chaos | Panmixia | 0.047 | 10 | 2 | 1 |
| *Etelis marshi* | Pygmy Ruby Snapper | CytB | 768 | 436 | 40 | 0.55 | 0.000 | 0.506 | Panmixia | Ambiguous | 0.102 | 11 | 1 | NA |
| *Gymnothorax flavimarginatus* | Yellow-edged Moray | CytB, CO1 RAG2 | 80 | 570,  658,  1167 | 365 | 1.00 | 0.000 | 0.167 | Panmixia | Stepping-Stone 2 Parameter | 0.047 | 4 | 1 | 4 |
| *Gymnothorax undulatus* | Undulated Moray | CytB CO1RAG2 | 112 | 570,  658,  1167 | 365 | 0.97 | 0.004 | 0.020 | Panmixia | Ambiguous | 0.102 | 5 | 1 | NA |
| *Halichoeres ornatissimus* | Ornate Wrasse | CO1 | 311 | 526 | 40 | 0.81 | -0.007 | 0.904 | Panmixia | Stepping-Stone | 0.047 | 14 | 1 | 14 |
| *Heterocentrotus mammillatus* | Slate Pencil Urchin | CO1 | 174 | 410 | 8 | 0.69 | 0.001 | 0.160 | Panmixia | Stepping-Stone | 0.047 | 6 | 1 | 6 |
| *Holothuria atra* | Black Sea Cucumber | CO1 | 253 | 423 | 15 | 0.67 | 0.020 | 0.001 | Chaos | Stepping-Stone | 0.047 | 9 | 1 | 9 |
| *Holothuria whitmaei* | Teated Sea Cucumber | CO1 | 261 | 446 | 15 | 0.69 | -0.001 | 0.758 | Regional: 2 Currents | Stepping-Stone | 0.047 | 7 | 2 | 7 |
| *Lutjanus kasmira* | Blue-striped Snapper | CytB | 385 | 519 | 31 | 0.86 | 0.000 | 0.266 | Panmixia | Panmixia | 0.047 | 9 | 1 | 1 |
| *Mulloidichthys flavolineatus* | Yellowstriped Goatfish | CytB | 412 | 715 | 60 | 0.63 | 0.001 | 0.062 | Chaos | Chaos | 0.047 | 11 | 3 | 3 |
| *Mulloidichthys vanicolensis* | Yellowfin Goatfish | CytB | 235 | 716 | 36 | 0.69 | 0.000 | 0.406 | Chaos | Stepping-Stone | 0.047 | 12 | 2 | 12 |
| *Myripristis berndtii* | Blotcheye Solderfish | CytB | 147 | 644 | 55 | 0.66 | 0.000 | 0.547 | Regional: Other | Ambiguous | 0.511 | 7 | 2 | NA |
| *Ophiocoma erinaceus* | Spiny Brittle Star | 16S | 326 | 482 | 50 | 0.70 | 0.194 | 0.001 | Chaos | Ambiguous | 0.472 | 9 | 2 | NA |
| *Ophiocoma pica* | Yellow-spotted Brittle Star | 16S | 419 | 488 | 50 | 0.71 | 0.023 | 0.001 | Stepping-Stone | Stepping-Stone | 0.047 | 10 | 10 | 10 |
| *Panulirus marginatus* | Banded Spiny Lobster | CO1 | 464 | 662 | 365 | 0.90 | 0.001 | 0.011 | Regional: Other | N-island | 0.047 | 12 | 3 | 12 |
| *Panulirus penicillatus* | Pronghorn Spiny Lobster | CO1 | 268 | 460 | 270 | 0.72 | 0.011 | 0.001 | Regional: High-Low | Stepping-Stone | 0.047 | 8 | 2 | 8 |
| *Parupeneus multifasciatus* | Manybar Goatfish | CytB | 473 | 618 | 44 | 0.70 | 0.000 | 0.529 | Regional: Other | Ambiguous | 0.131 | 13 | 2 | NA |
| *Pristipomoides filamentosus* | Hawaiian Pink Snapper | CytB | 644 | 504 | 45 | 0.55 | -0.006 | 0.957 | Regional: Other | Ambiguous | 0.225 | 9 | 3 | NA |
| *Squalus mitsukurii* | Shortspine Spurdog Shark | CR | 110 | 670 | 0 | 0.56 | -0.048 | 0.774 | Panmixia | Stepping-Stone 2 Parameter | 0.047 | 4 | 1 | 4 |
| *Stegastes fasciolatus* | Pacific Gregory Damsel | CR | 219 | 477 | 30 | 0.79 | 0.001 | 0.026 | Regional: Other | Ambiguous | 0.212 | 7 | 4 | NA |
| *Stenella longirostris* | Spinner Dolphin | CR | 501 | 417 | 0 | 0.60 | 0.016 | 0.001 | Regional: Other | Regional: Other | 0.047 | 8 | 3 | 3 |
| *Triaenodon obesus* | Whitetip Reef Shark | CR | 154 | 1026 | 0 | 0.62 | 0.095 | 0.010 | Panmixia | Ambiguous | 0.194 | 9 | 1 | NA |
| *Zebrasoma flavescens* | Yellow Tang | CytB | 558 | 614 | 54 | 0.69 | 0.000 | 0.534 | Chaos | Stepping-Stone | 0.047 | 10 | 4 | 10 |
