# Supplementary material for: A coalescent sampler successfully detects biologically meaningful population structure overlooked by F‐statistics
Source: Evol Appl. 2018 Oct 15;12(2):255–65. doi: 10.1111/eva.12712 (PMC6346657; doi:10.1111/eva.12712)
Supplement: Supplementary file 6 [file EVA-12-255-s006.docx]

**ToBo Lab Authors**

Kim Andrews

Illiana Baums

Moises Bernal

Chris Bird

Holly Bollick

Richard Coleman

Matt Craig

Toby Daly-Engel

Joseph DiBattista^1,2^

Jeff Eble

Iria Fernandez-Silva

Michelle Gaither

Matthew Iacchei

Joshua S. Reece

Derek Skillings

Molly Timmers

Ellen Waldrop

Jonathon Whitney

*1 School of Molecular and Life Sciences, Curtin University, PO Box U1987, Perth, WA 6845, Australia
2 Australian Museum Research Institute, Australian Museum,*[*1 William St, Sydney, NSW 2010, Australia*](https://maps.google.com/?q=1+William+St,+Sydney,+NSW+2010,+Australia&entry=gmail&source=g)
