# Supplementary material for: A coalescent sampler successfully detects biologically meaningful population structure overlooked by F‐statistics
Source: Evol Appl. 2018 Oct 15;12(2):255–65. doi: 10.1111/eva.12712 (PMC6346657; doi:10.1111/eva.12712)
Supplement: Supplementary file 7 [file EVA-12-255-s007.docx]

**Supplemental Figure Legends:**

Figure S1. Thermodynamic marginal likelihoods from each of 3 replicates performed on 8 or 9 migrate models for each species. These are the likelihoods from which model selection was performed. Blue dots depict marginal likelihood of each replicate, while black dots label the mean of these three values. Black lines give the 95% confidence interval for small sample sizes which was calculated as $\frac{\sigma}{\sqrt{3}}*4.303$.

Figure S2. Comparison of values for effective population size (N_e_; black) and effective female migrants per generation (N_e_m; white) that were simulated by IBDsim with the posterior distributions estimated by Migrate. Known simulated values are depicted as circles, while posterior distributions are given as violin plots.

Figure S3. Violin plots depicting posterior distributions of Θ and N_e_m estimated from empirical data for all species that selected non-ambiguous models other than panmixia. Grey plots depict northwestward gene flow and white plots depict southeastward gene flow.

Figure S4. Histograms of the slope calculated for 10,000 linear models of the relationship between ln(Θ) and the area of shallow ocean habitat less than 10 fathoms deep, where values of Θ for each species were randomly sampled from the posterior for Θ for each island population.
